# An integrated analysis of genes and functional pathways for aggression in human and rodent models

**DOI:** 10.1038/s41380-018-0068-7

**Published:** 2018-06-01

**Authors:** Yanli Zhang-James, Noèlia Fernàndez-Castillo, Jonathan L Hess, Karim Malki, Stephen J Glatt, Bru Cormand, Stephen V Faraone

**Affiliations:** 1 Department of Psychiatry and Behavioral Sciences, SUNY Upstate Medical University, Syracuse, New York, NY USA; 2 Departament de Genètica, Microbiologia i Estadística, Facultat de Biologia, Universitat de Barcelona, Catalonia, Spain; 3 Centro de Investigación Biomédica en Red de Enfermedades Raras (CIBERER), Madrid, Spain; 4 Institut de Biomedicina de la Universitat de Barcelona (IBUB), Catalonia, Spain; 5 Institut de Recerca Sant Joan de Déu (IR-SJD), Esplugues de Llobregat, Spain; 6 King’s College London, MRC Social, Genetic and Developmental Psychiatry Centre at the Institute of Psychiatry, Psychology and Neuroscience (IOPPN), London, UK; 7 Department of Neuroscience and Physiology, SUNY Upstate Medical University, Syracuse, New York, NY USA; 8 K.G. Jebsen Centre for Research on Neuropsychiatric Disorders, University of Bergen, Bergen, Norway

**Keywords:** Psychiatric disorders, Genetics

## Abstract

Human genome-wide association studies (GWAS), transcriptome analyses of animal models, and candidate gene studies have advanced our understanding of the genetic architecture of aggressive behaviors. However, each of these methods presents unique limitations. To generate a more confident and comprehensive view of the complex genetics underlying aggression, we undertook an integrated, cross-species approach. We focused on human and rodent models to derive eight gene lists from three main categories of genetic evidence: two sets of genes identified in GWAS studies, four sets implicated by transcriptome-wide studies of rodent models, and two sets of genes with causal evidence from online Mendelian inheritance in man (OMIM) and knockout (KO) mice reports. These gene sets were evaluated for overlap and pathway enrichment to extract their similarities and differences. We identified enriched common pathways such as the G-protein coupled receptor (GPCR) signaling pathway, axon guidance, reelin signaling in neurons, and ERK/MAPK signaling. Also, individual genes were ranked based on their cumulative weights to quantify their importance as risk factors for aggressive behavior, which resulted in 40 top-ranked and highly interconnected genes. The results of our cross-species and integrated approach provide insights into the genetic etiology of aggression.

## Introduction

Aggressive behavior is an evolutionarily conserved, heritable trait that is essential for survival and fitness. In humans, aggressive behavior is also shaped by societal and cultural constraints. Context-inappropriate aggression can cause great harm to society, families, and individuals, and has been associated with neuropsychiatric disorders, such as attention-deficit/hyperactivity disorder (ADHD) [1, 2], schizophrenia (SCZ), and bipolar disorder (BIP) [3–5].

The heritability of human aggression has been estimated around 50% [6]. Its complex genetic architecture interacts with environmental factors [7–9]. Decades of animal studies have yielded strong neurochemical and physiological evidence that points to conserved common pathways across species such as serotoninergic and dopaminergic neurotransmission and hormonal signaling [10]. These data helped to inform candidate gene selection for human studies on serotonergic and dopaminergic genes (*MAOA*, *5HTT*, *HTR1B*, *HTR2A*, *DAT*, *DRD2*, *DRD4*), as well as hormone-related genes (*AR*, *ESR1*, *AVP*, *OXTR*) [11]. However, the largest meta-analysis of candidate genes performed to date [12] did not find any significant association. Genome-wide association studies (GWAS) of aggression have been underpowered to detect common variants of small penetrance associated with complex phenotypes [11, 12]. The largest GWAS, which was performed by the Early Genetics and Lifecourse Epidemiology (EAGLE) consortium (http://research.lunenfeld.ca/eagle/), reported association with one SNP, rs11126630, at a suggestive significance level (*p* = 5.3*e*−08). Among the “classical” candidates evaluated in the EAGLE dataset, only one gene, *AVPR1A*, encoding the arginin vasopressin receptor 1A, showed a nominal association with aggression (*p* = 1.6*e*−03) [13].

Studies of rare human genetic conditions [14] and gene knockouts (KOs) in mice [15, 16] show that many genetic determinants play critical roles in shaping the emotional circuitry of the brain and modulating aggressive behavior [17, 18]. For example, rare mutations in the gene encoding methyl-CpG-binding protein 2 (MeCP2), a chromatin-associated protein involved in transcription regulation, cause Rett syndrome, mental retardation, and increased aggression [19]. *Mecp2* KO mice also show increased aggression [20]. Another example is the gene for prion protein, the mutation of which causes inherited prion diseases and aggressive behavior in humans [21]. Mice with depleted prion protein showed increased aggressiveness [22], possibly related to the role of the prion protein in regulating cytoskeleton and associated proteins [23]. Despite convincing evidence supporting these high-risk genes from human single-gene disorders and KO mice studies, none have reached genome-wide significance in GWAS of aggression. The best *p*-value was for gene *LRRC7* in a study of children (*p* = 4*e*−06) [24]. *LRRC7* interacts with cytoskeleton molecules and is involved in synaptic spine structure and patterning [25]. *Lrrc7* KO mice show significantly increased fighting among littermates [26]. Nevertheless, similar pathways such as synaptic development, axon guidance, and MAPK signaling emerge when examining genes from the top GWAS findings (*p* ≤ 5*e*−05, ref. [11]). These pathways were also enriched in genes identified through transcriptomic studies of animal models of aggression in mice [27, 28], rats [29], zebrafish [30], and *Drosophila* [31]. With increasing sample sizes, some of these genes and, perhaps, additional risk loci may emerge in future GWAS.

Meanwhile, we postulate that a cross-species and integrated approach combining different modalities of genetic data can yield a clearer understanding of the genetics of aggression. Our study focuses on several categories of available genome-wide data: (1) genes derived from human GWAS studies: we updated and expanded the GWAS genes catalog from [11]. (2) Genes found in transcriptome studies of rodent models (brain tissue): we obtained the raw expression data from four unique selective-bred rodent models and re-analyzed them to identify strain-specific genes differentially expressed in high- versus low-aggressive lines. (3) Previously published sets of human genes implicated in aggression phenotypes in human single-gene disorders cataloged in online Mendelian inheritance in man (OMIM) [14] and mouse genes implicated in KO studies [15]. This latter category comprises high risk and possibly causal genes because single-gene changes results in (or modifies) the individual’s aggressive behavior. Although each of these studies have intrinsic limitations, the convergence of evidence by cross-referencing and integrating the available data may lead to a more comprehensive and confident understanding of the genetic basis of aggression in humans and mammals.

## Material and methods

### Aggression gene lists

#### GWAS gene sets

We updated previous reported GWAS genes for aggression [11] with studies published until August 2016, discarding those that were performed in samples of individuals with other psychiatric disorders (such as drug dependence or BIP). Selected GWAS included four studies for the adult GWAS gene set [32–35] and five for the child gene set [13, 36–39]. Two studies were GWAS meta-analyses [13, 32]. For detailed procedures, see Supplementary Figure 1. Eligible SNPs and retrieved genes are in Supplementary Table 1. As a negative control, we generated a gene list from the GWAS catalog (https://www.ebi.ac.uk/gwas/) by combining signals from 14 phenotypes not related to the nervous system in samples of individuals with European ancestry and sample sizes that are similar to those of the nine GWAS of aggressive behaviors. We used the same procedures to retrieve associated signals and nearby genes (Supplementary Table 2).

#### Genes from rodent model transcriptomes

Genome-wide transcriptome data were available for four genetic rodent models of aggression: three inbred mouse strains [28] and one rat strain [29] along with their comparable low-aggression strains. Data had been generated using Affymetrix Mouse Genome 430 2.0 or Rat Genome U34 microarrays, and acquired from the Gene Expression Omnibus (dataset series GSE29552) [29] or the author [28]. The mouse strains from the study by Malki et al. were (1) Turku aggressive and Turku non-aggressive mice selected from a colony of Swiss albino mice in Turku (Finland) based on high male–male aggression in a dyadic test against non-aggressive mice [40]; (2) short attack latency and long attack latency mice bred from a wild-type *Mus musculus domesticus* population in Groningen, Holland by selecting on average attack latency in a resident-intruder test [41]; (3) North Carolina aggressive (NC900) and non-aggressive (NC100) mice selected from out-bred NCR mice in North Carolina (USA) showing increased aggression and reactivity to stimulation [42]. For convenience, we use country of origin to denote the strains: Finland, Holland, and USA, respectively. The rat model data were derived from selectively bred high responder (bHR) and low responder (bLR) Sprague-Dawley rats. These groups showed differences in emotional reactivity and exploratory behavior, aggression, impulsivity, and proclivity to psychostimulant abuse [43, 44]. We used weighted gene co-expression network analysis [45] to identify strain-specific genes in co-expression modules significantly associated with aggression (for details of methods, see Supplementary File 1; gene sets were listed in Supplementary Table 3).

#### OMIM and KO mice genes

We used previously published sets of human genes implicated in aggression phenotypes in human single-gene disorders cataloged in OMIM (*N* = 85) [14] and mouse genes implicated in KO studies (*N* = 89) [15]. Genes in these two sets were included in Supplementary Table 4.

### Genetic correlation (LD score) analyses between aggression and psychiatric disorders

We estimated the genetic correlation of aggression with six other psychiatric disorders (ADHD, SCZ, BIP, autism spectrum disorders (ASDs), major depression (MDD) and post-traumatic stress disorders (PTSDs), by LD score (LDSC) regression analysis [46]. We used the largest aggression GWAS meta-analysis of children samples, the EAGLE (Early Genetics and Lifecourse Epidemiology Consortium) study [13] and a recently published GWAS of antisocial behavior by Tielbeek et al. [47], which included 64% adult and 36% child samples. None of the four adult aggression samples reported so far have either sufficient sample sizes or summary statistics available. For ADHD, SCZ, BIP, ASD, MDD, and PTSD, the sources of summary statistics are in Supplementary Table 5.

### Gene set overlap analysis and gene ranking

Rodent transcriptome genes were converted to human orthologues using biomaRt [48, 49]. Gene overlap among the sets was evaluated using one-tailed Fisher’s exact tests. We ranked individual genes by their total numbers of occurrences in these lists. We also ranked them using a simple weighted sum method: aggression gene rank = 1 × (total occurrence in human GWAS studies) + 0.5 × (total occurrence in four rodent model transcriptome lists) + 1.5 × (total occurrence in OMIM and KO mice genes). The rationale was to add 50% more weight to the genes in OMIM or in KO mice lists compared with the GWAS genes lists because alterations in the first set of genes are more firmly linked to aggression. We discounted the weight to 0.5 for the rodent transcriptome genes given the limitations of these studies, including the limited phenotypes used in selective breeding, small sample sizes, the limited brain regions and age ranges studied, and the potential for confounding cause and effect in such studies. This weighting scheme also ensured that the maximum possible ranking scores from all four rodent models would be 2, equal to the maximum possible score of the two GWAS lists. The three main categories of studies (human GWAS, rodent transcriptome studies, and the high-risk gene set combining KO mice and OMIM genes) were evaluated for overlap using Fisher’s exact test.

### Ingenuity pathway analysis: pathway and network analysis

Individual gene sets were imported to ingenuity pathway analysis (IPA) to assess canonical pathway enrichment. The negative log of Fisher’s exact test *p*-values are reported. Using a *p* < 0.05 cutoff, we coded the pathway enrichment as a binary variable for the subsequent analysis, with 1 indicating significant enrichment and 0 no enrichment. We examined the pathway enrichment similarities among the gene sets with classical metric multidimensional scaling (MDS) using the Rogers-Tanmoto correlation for binary data [50] in STATA 14. The configuration for the first four-dimensional Euclidean space was visualized in a 4D plot (a, b, c, and node size) created in R. We explored the activation/inhibition states of the top enriched canonical pathways using IPA’s activation *Z*-score tool. Because gene expression changes are needed to calculate activation *Z*-scores and because shared pathway enrichment is needed for the activation comparison, this analysis was only performed on the USA mice and the rat models. Finally, we used IPA’s network generation algorithm to identify the highly interconnected networks of the top 40 ranked genes according to the weighted method described in the previous section. These networks were visualized using IPA’s Path Designer tool. We imported the networks into Cytoscape to measure the number of interactions (degree) of the top-ranked 40 genes with other genes in the network. Logarithms of the degree estimates were compared for rodent versus human aggression genes using quantile regression.

## Results

### Aggression gene sets

#### Human GWAS genes

A total of 175 and 281 genes were selected from four adult and five children GWAS gene sets, respectively (Supplementary Figure 1 and Supplementary Table 1). Six genes were present in both sets: *ALK*, *LAMA2*, *NFKB1*, *OSMR*, *RBFOX1*, and *WDR62* (significant overlap by Fisher’s exact test, *p* = 0.038, Table 1). The control GWAS gene list comprises 172 genes (Supplementary Table 2); only one gene was shared with either the adult (*ARHGEF3*) or the child (*LY86*) datasets (non-significant). LDSC regression found a significant positive correlation between the EAGLE GWAS meta-analysis of aggression in children [13] and ADHD (*p* = 9.75*e*−05), and positive correlations between the recently published GWAS meta-analysis of antisocial behavior [47] with ADHD (*p* = 4.4*e*−03) and MDD (*p* = 3.53*e*−03). No significant genetic correlations were found with any other disorders examined (SCZ, ASD, BIP, and PTSD), or between the aggression GWAS meta-analyses (Supplementary Figure 2).

**Table 1:** Gene list overlaps

Gene overlap	GWAS_Adult	GWAS_Child	KO_Mice	Mice_Finland	Mice_Holland	Mice_USA	OMIM	Rat
(*N*/*p-*values)	(*N* = 175)	(*N* = 281)	(*N* = 89)	(*N* = 381)	(*N* = 271)	(*N* = 397)	(*N* = 85)	(*N* = 211)
GWAS_Child	**6**							
(*N* = 281)	***p*** **=** **0.038**							
KO_Mice	1	**4**						
(*N* = 89)	*p* = 0.544	***p*** **=** **0.037**						
Mice_Finland	1	6	**6**					
(*N* = 381)	*p* = 0.269	*p* = 0.662	***p*** **=** **0.007**					
Mice_Holland	2	2	1	**13**				
(*N* = 271)	*p* = 1	*p* = 0.597	*p* = 1	***p*** **=** **0.002**				
Mice_USA	3	**11**	4	11	**12**			
(*N* = 397)	*p* = 1	***p*** **=** **0.029**	*p* = 0.102	*p* = 0.193	***p*** **=** **0.013**			
OMIM	1	3	**4**	3	1	1		
(*N* = 85)	*p* = 0.528	*p* = 0.118	***p*** **=** **0.001**	*p* = 0.221	*p* = 1	*p* = 1		
Rat	1	4	**6**	5	5	**10**	3	
(*N* = 211)	*p* = 1	*p* = 0.546	***p*** **<** **0.0001**	*p* = 0.606	*p* = 0.215	***p*** **=** **0.010**	*p* = 0.061	
GWAS_Contrl	1	1	2	1	4	2	1	2
(*N* = 172)	*p* = 1	*p* = 0.737	*p* = 0.179	*p* = 0.389	*p* = 0.301	*p* = 0.779	*p* = 0.522	*p* = 0.704

The total number of genes in each lists and shared numbers of genes across different list is tabulated. Fisher’s exact test was used to evaluate the lists overlap based on ~22,000 total known genes for rodents and human. Significant overlaps (*p* < 0.05, uncorrected) are highlighted as bold

#### Rodent transcriptome genes

One gene module was significantly downregulated in bHR rats compared to the bLR rats (ME6, Benjamini-Hochberg (BH)-corrected *p* < 0.05). For the mouse models, one module for each line was significantly downregulated in high-aggression versus low-aggression lines (Finland: ME12, uncorrected *p*-value <0.005, BH *p*-value <0.2; Holland: ME29, BH *p* < 0.05; USA: ME11, BH *p* < 0.05). One module was significantly upregulated for the Holland aggressive versus non-aggressive line (ME22, *p*-value = 8.4*e*−04, BH *p*-value = 0.028). Supplementary Figure 3 plots the eigengene expression for all five significant rodent modules.

We combined the two modules for the Holland lines for downstream analyses. This yielded one significantly associated gene set for each single rodent model for a total of four gene sets. The gene sets and eigengene expression for individual genes in each module are shown in Supplementary Table 3. The Holland mice shared 12 genes with the USA lines (*p* = 0.013) and 13 with the Finland lines (*p* = 0.002) (Table 1). The Finland and USA lines shared 11 genes (non-significant). The rat model shared five genes with the Finland and Holland lines (non-significant) and 10 with the USA lines (*p* = 0.01).

### Comparison of gene lists and gene ranking

The human GWAS and rodent transcriptome gene sets were cross-referenced with previously published OMIM and KO mice gene sets [15, 51] (Supplementary Table 4). Table 1 summarizes the overlap among all possible pairs and the Fisher’s exact test *p*-values. Although the number of overlapping genes was small, some reached statistical significance. Notably, the USA mouse shared 11 genes with the child GWAS gene list (*p* = 0.029), and the KO mice set shared four genes with the child GWAS genes (*p* = 0.037), four with the human OMIM genes (*p* = 0.001), six with the Finland mouse (*p* = 0.007), and six with the rat model (*p* < 0.001). None of the eight aggression gene lists showed any significant overlap with the GWAS control gene set.

Supplementary Table 4 lists all the 1767 genes from the eight aggression gene sets (adult and children GWAS, transcriptomics in four rodent models, KO mice and OMIM) and ranks them based on their number of occurrences and weighted ranks for aggression (only human orthologs were included from the rodent genes). *MAOA* was ranked highest with both methods. One hundred and nineteen genes appeared in at least two lists. Forty of them have a weighted ranking score ≥2 (Table 2) and almost all are involved in neuronal functions: synaptic transmission (*n* = 13, GO: 0007268, *p* = 5*e*−09), nervous system development (*n* = 18, GO: 0007399, *p* = 7*e*−08), synapse (*n* = 11, GO: 0045202, *p* = 1*e*−08), neuron projection (*n* = 9, GO: 0043005, *p* = 2*e*−05), and neuroactive ligand–receptor interaction (*n* = 5, KEGG: 04080, *p* = 2*e*−04). Detailed information on individual genes and references to the original studies are shown in Supplementary Table 6.

**Table 2:** Top 40 ranked genes

Gene symbol	Weighted ranking	Gene name	Gene symbol	Weighted ranking	Gene name
*MAOA*	4	Monoamine oxidase A	*ECM1*	2	Extracellular matrix protein 1
*ERBB4*	3	Erb-b2 receptor tyrosine kinase 4	*EEF1A2*	2	Eukaryotic translation elongation factor 1 alpha 2
*GRIA3*	3	Glutamate ionotropic receptor AMPA type subunit 3	*EHMT1*	2	Euchromatic histone lysine methyltransferase 1
*MECP2*	3	Methyl-CpG-binding protein 2	*GAD2*	2	Glutamate decarboxylase 2
*PRNP*	3	Prion protein	*GDI1*	2	GDP dissociation inhibitor 1
*AVPR1A*	2.5	Arginine vasopressin receptor 1A	*GRID1*	2	Glutamate ionotropic receptor delta type subunit 1
*CHMP2B*	2.5	Charged multivesicular body protein 2B	*GRN*	2	Granulin
*EN2*	2.5	Engrailed homeobox 2	*GSK3A*	2	Glycogen synthase kinase 3 alpha
*FGF14*	2.5	Fibroblast growth factor 14	*HSF1*	2	Heat shock transcription factor 1
*HDAC4*	2.5	Histone deacetylase 4	*LAMA2*	2	Laminin subunit alpha 2
*KCNJ18*	2.5	Potassium voltage-gated channel subfamily J member 18	*MAPK15*	2	Mitogen-activated protein kinase 15
*LRRC7*	2.5	Leucine rich repeat containing 7	*MME*	2	Membrane metalloendopeptidase
*SERPINI1*	2.5	Serpin family I member 1	*NFKB1*	2	Nuclear factor kappa B subunit 1
*ACHE*	2	Acetylcholinesterase (Cartwright blood group)	*NPY1R*	2	Neuropeptide Y receptor Y1
*ALDH5A1*	2	Aldehyde dehydrogenase 5 family member A1	*OSMR*	2	Oncostatin M receptor
*ALK*	2	Anaplastic lymphoma receptor tyrosine kinase	*PNOC*	2	Prepronociceptin
*CACNB3*	2	Calcium voltage-gated channel auxiliary subunit beta 3	*RBFOX1*	2	RNA-binding protein, fox-1 homolog 1
*CADM1*	2	Cell adhesion molecule 1	*SPAST*	2	Spastin
*CRHR1*	2	Corticotropin releasing hormone receptor 1	*SYN1*	2	Synapsin I
*DNAJB5*	2	DnaJ heat shock protein family (Hsp40) member B5	*WDR62*	2	WD repeat domain 62

**Fig. 1:**
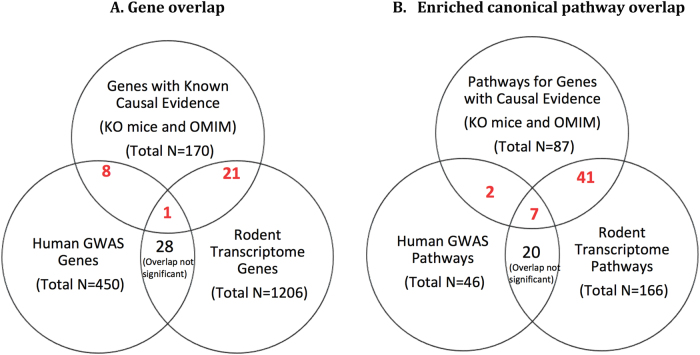
Venn diagram of gene and pathway overlaps from three categories of genetic evidence. **a** Number of gene overlaps. One gene, *ERBB4*, was shared in all three categories. A total of nine GWAS genes were also high-risk genes (*p* = 0.007), and 22 rodent transcriptome genes were high-risk genes (*p* < 0.0001). In contrast, the overlap between the human GWAS and the rodent transcriptome genes (*n* = 29) was not statistically significant (*p* = 0.29). **b** Shared canonical pathway enrichments. The total pathway overlap between the human GWAS and high-risk genes (*N* = 9, *p* = 0.015) and the overlap between the rodent transcriptome and high-risk genes (*N* = 48, *p* = 0.005) were significant. In contrast, the pathway overlap between the human GWAS and rodent transcriptome genes was not significant (*N* = 27, *p* = 0.20). Red numbers indicate significant overlaps.

Analysis of overlap among the three main categories human GWAS, rodent transcriptome, and high-risk genes (i.e., KO mice and OMIM genes) revealed one gene, *ERBB4*, with supporting evidence from all three categories. A total of nine GWAS genes were also high-risk genes (*p* = 0.007), and 22 rodent transcriptome genes were high-risk genes (*p* < 0.0001). However, the overlap between GWAS and transcriptome genes (*n* = 29) was not statistically significant (*p* = 0.29). The Venn diagram in Fig. 1a shows these overlaps.

### Pathway analysis

We performed canonical pathway enrichment analysis for each aggression gene set. Eleven pathways were significant in at least three aggression gene sets (highlighted in red in Supplementary Table 7) and included axonal guidance signaling, CREB signaling in neurons, ERK/MAPK signaling, G-protein coupled receptor (GPCR) signaling, GABA, and serotonin receptor signaling and reelin signaling. The most shared pathways between any two sets were 11 pathways in common in the OMIM and KO mice gene sets, followed by eight shared pathways between the child GWAS and USA mice and seven between the child GWAS and the rat model. The adult GWAS only shared four pathways with the USA mouse and one with the rat model, although it did share five pathways with the OMIM list. There were no common significantly enriched pathways among all three mouse strains. The Finland and Holland mouse strains had almost no overlap with other gene lists. The child and adult GWAS sets shared only one pathway, phospholipase C signaling. The percentage of enriched pathways shared with any other gene list out of the total enriched canonical pathways for each list were ranked as follows: OMIM 59%, USA mouse 53%, KO mice 48%, Child GWAS and Rat both 44%, adult GWAS 43%, Finland mouse 20% and Holland 11%.

MDS was used to analyze the pathway enrichment similarities shared by the different gene sets. The 4D plot (Supplementary Figure 4 and Supplementary File 2) shows that six sets of aggression genes (two human GWAS, OMIM genes, and all three mouse models) were closely clustered in the first three dimensions, which explained 71.6% of the total variance. The rat model, the KO mice genes, and GWAS control genes were distinctly separable in the first three dimensions. The child GWAS genes mainly loaded on the fourth dimension, which accounted for an additional 12.4% of the variance. The USA mouse model mainly loaded in the fifth dimension, which accounted for an additional 8.6% of the variance (not shown).

Examining the common pathways shared by the three main categories of genetic evidence returned seven canonical pathways (Table 3). Among them, five contained top-ranked genes and the G-protein-coupled receptor signaling pathway was also significantly enriched with the top-ranked genes (*p* = 0.002). As seen for the analysis of gene overlap, the total pathway overlap between the human GWAS and high-risk genes (*N* = 9, *p* = 0.015) and the overlap between the rodent transcriptome and high-risk genes were significant (*N* = 48, *p* = 0.005). In contrast, the pathway overlap between the human GWAS and rodent transcriptome genes was not significant (*N* = 27, *p* = 0.20). The Venn diagram in Fig. 1b shows this overlap.

**Table 3:** Canonical pathways shared by all three categories of genes

Canonical pathways	Human GWAS genes	Rodent transcriptome genes	Causal genes
Signaling by Rho family GTPases	2.294	1.594	1.847
ERK/MAPK signaling	2.093	2.238	2.965
G-protein-coupled receptor signaling	1.999	3.971	9.286
Axonal guidance signaling	1.947	1.714	2.499
Reelin signaling in neurons	1.939	1.759	1.432
Molecular mechanisms of cancer	1.647	2.591	3.075
Gαs signaling	1.609	2.054	5.589

The negative log *p*-values of enrichment assessed by Fisher’s exact test were reported in the table

The IPA activation *Z*-score analysis performed on the shared canonical pathways between the USA mice and rat gene sets predicted mostly opposite activities except for one: dopamine DARPP32 feedback in cAMP signaling. This pathway was inhibited in both models (Supplementary Figure 5).

### Network analysis of the top genes

**Fig. 2:**
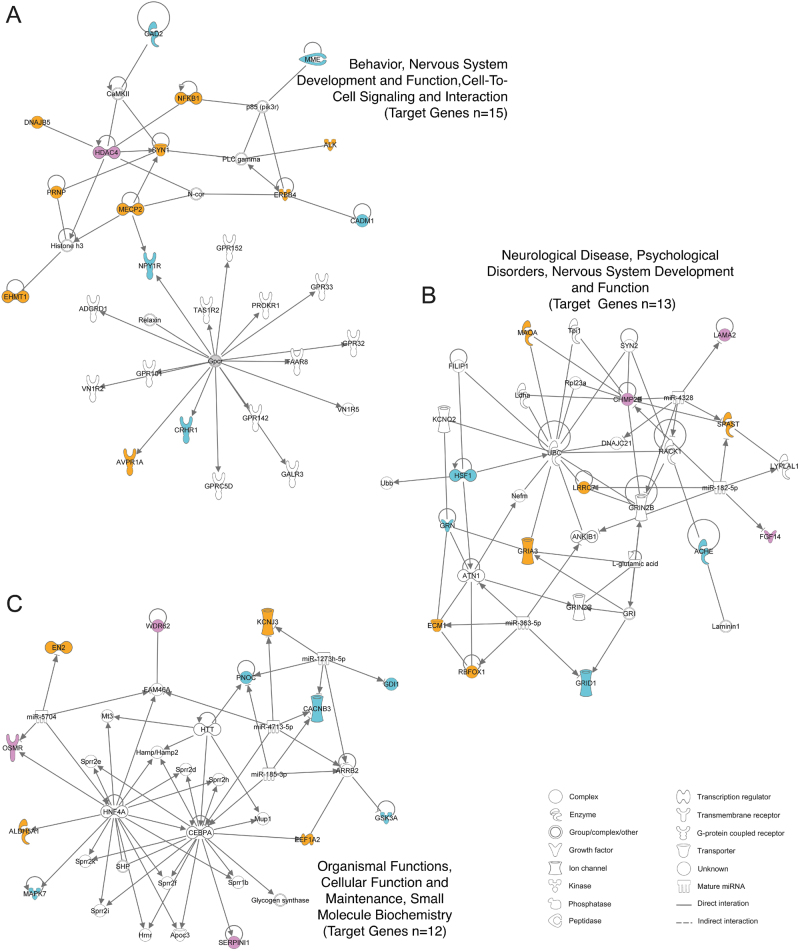
IPA network analysis of the 40 top-ranked genes. Network A is involved in behavior, nervous system development and function, and cell-to-cell signaling and interaction (score 35, 15 genes). Network B is involved in neurological disease, psychological disorders, nervous system development and function (score 29, 13 genes). Network C is involved in organismal functions, cellular function and maintenance, small molecule biochemistry (score 26, 12 genes). Genes highlighted in color correspond to the 40 top-ranked genes. In purple, human-only aggression genes (from GWAS and OMIM studies), in blue, rodent-only aggression genes (rodent model transcriptomic genes and KO mice genes), and in orange, genes from both human and rodent studies.

The 40 top-ranked genes were highly interconnected in three tightly clustered networks identified by IPA’s network generation algorithm using direct relationships from the Ingenuity^®^ Knowledge Base. These networks were related to nervous system development and function, neurological disease and psychological disorders, and cellular function and maintenance (Fig. 2). The total number of interactions with other genes, i.e, degree, was significantly higher for the human compared with the rodent aggression genes (*F*_(1, 22)_ = 10.59, *p* = 0.004). Genes from both human and rodent studies also had a significantly higher degree than the rodent-only genes (*F*_(1, 28)_ = 4.97, *p* = 0.034), but this degree is not different from that of the human-only genes (Supplementary Figure 6).

## Discussion

Our study integrates all prior human and rodent genetic studies of aggression to overcome their individual limitations and to gain a more robust insight into the genetic etiology of aggression. We found that genes from these different types of studies share some significant overlap at both the gene and biological pathway levels. Their lack of overlap with a control GWAS set confirms that they harbor genetic information relevant to aggression. The two main categories, human GWAS genes and rodent transcriptome genes, however, did not overlap significantly, although they both shared significant overlap with distinct subsets of the high-risk genes (those obtained from human OMIM phenotypes and KO mice). This pattern was also true for the pathway enrichments. This suggests differences in the genetic etiology of human and rodent aggression or the possibility that human GWAS and transcriptomic studies of rodent brains capture different sets of genes involved in the etiology of aggression. Finally, using a weighted ranking method, we provided a hierarchical list of genes associated with aggression.

The adult and child GWAS sets had only six genes in common: *ALK*, *LAMA2*, *NFKB1*, *OSMR*, *RBFOX1*, and *WDR62*. Albeit small, this overlap was statistically significant. All six genes are essential for neurodevelopment. *ALK* encodes a tyrosine kinase receptor linked to neuroblastoma [52]. *LAMA2* encodes an extracellular matrix protein. Its mutation causes denervation atrophy of the muscle [53]. *OSMR* is a member of the type-I cytokine receptor family and is essential for the development of a subtype of nociceptive neurons in the dorsal root ganglia [54]. *NFKB1* is a transcription factor involved in regulating responses of neurons to activation of different signaling pathways in a variety of physiological and pathological conditions [55]. *RBFOX1* is a splicing factor implicated in many neurodevelopmental and psychiatric disorders and several evidences have highlighted this gene as a candidate for aggression [56, 57]. *WDR62* is a centrosomal and nuclear protein linked to autosomal recessive microcephaly [58, 59].

Most genes in the adult and child GWAS sets did not overlap. The two sets only shared one canonical pathway. Furthermore, the EAGLE GWAS meta-analysis of children’s aggression [13] and the Tielbeek’s GWAS of antisocial behavior (64% adult) [47] do not show genetic correlation. The sample size of children in Tielbeek’s GWAS was only half of the EAGLE’s, likely too small for LDSC regression and explaining their lack of correlation despite the presence of children samples in both datasets. More importantly, the results highlight the possible genetic differences, although it may be due to phenotype differences between the two samples. Indeed, aggressive behaviors often manifest in different forms and are triggered by different risk factors across the lifespan [60]. Some aggressive behaviors in childhood predict aggression in later life; others are temporary and disappear at later ages, such as temper tantrums in toddlers [61] and adolescence-limited antisocial behavior [62]. Adult onset aggression is often linked to physical or emotional trauma, substance use, medical illnesses, or brain injuries [60]. Our LDSC regression found significant correlations between the EAGLE’s child sample and ADHD, and between Tielbeek’s sample (64% adults) with both ADHD and MDD. The results support pleiotropic effects of shared common DNA variants on the comorbidity of aggression with ADHD in children, or with MDD in adults. The lack of genetic correlations of either dataset with SCZ, BIP, autism, or PTSD suggests that for these disorders, aggression may arise from different causal factors. Future studies are needed to fully address the genetic bases of the comorbidity between aggression and psychiatric disorders.

Among the rodent models, few biological pathways were shared, although there was some significant gene overlap. Differences in selective breeding may have fixed different genes into these models. Considering that the Finland and Holland lines were selectively bred based on a single behavioral criterion, either increased aggression toward non-aggressive mice [40] or decreased attack latency toward intruder mice [41], it is not surprising to see that they had the lowest numbers of enriched pathways and overlap with other lists. Our results suggest that their utility in modeling human aggression may be limited because the underlying genetic risk factors may be different from one another and more importantly from the genetic predisposition to aggression in humans. This notion was further supported by our network analysis of the 40 top-ranked genes, which showed that genes with evidence only from rodent studies have a fewer number of interactions in the network than those genes derived from human studies, suggesting more restricted functional impact of rodent genes on the network.

In contrast, the USA mice and the rat lines showed many overlapping pathways with the human GWAS genes, most notably with the child set. The USA mice and the rat model were bred for varying phenotypes: increased reactivity toward stimulation [42], novelty exploration, impulsivity, and vulnerability to psychostimulant abuse [29, 44]. Considering that human aggression is often accompanied by these traits, the USA mouse (NC900/NC100) and rat (bHR/bLR) lines may be better suited to model human aggression, particularly in the context of psychiatric comorbidities. However, their shared biological pathways often showed opposite inhibition/activation activities and, although statistically significant, the two lines shared <5% of genes (Table 1). Thus, it seems likely that different mechanisms, having some shared components, regulate aggression in these two models. The only consistent directional change of these shared pathways between the two rodent models, the downregulation of the dopamine-DARPP32 feedback in cAMP signaling, was also found altered in the ventral striatum and the frontal cortex of an operant mouse model of frustration showing aggressive behavior [63].

The most notable overlap observed in our analyses was for the 11 pathways shared between the human OMIM and the KO mice genes. Because the OMIM phenotypes are typically multidimensional phenotypes including medical, psychiatric, and aggressive features, it has not heretofore been clear whether the aggression observed in affected individuals was a direct genetic effect or if it was mediated via another phenotype. The substantial overlap with the KO mouse gene set provides strong evidence that the aggression in these OMIM disorders has a genetic etiology.

Among the top enriched pathways, several were previously well-known pathways for aggression: the dopamine, serotonin, glutamate, and GABA signaling pathways (Supplementary Table 7). Some of the pathways we found were not previously linked to aggression directly. However, there are several reasons to view them as functionally associated with aggression. For example, the GPCR signaling pathway, which was significantly enriched in our data (Table 3), mediates much receptor signaling including serotonin, dopamine, metabolic glutamate receptors, oxytocin and vasopressin receptors. ERK/MAPK and Rho-GTPase signaling form intracellular signaling cascades that orchestrate cellular responses of GPCR signaling. Axonal guidance and reelin signaling are important pathways for nervous system development and have been implicated in neuropsychiatric disorders such as bipolar, SCZ, and ADHD [64–68], which are often associated with aggression. Indeed, several recent reviews in human and animals have consistently identified these pathways [11, 15, 51]. Novel pathways that have never been linked to aggression offer us new perspectives on the pathophysiology of aggression. One interesting example is cancer signaling. Although it has never been implicated in aggression, it is not uncommon for cancer patients to display changed personalities and even violent behaviors [69]. The close relationship of cancer signaling with immune system offers a plausible mechanism linking cancer with many neuropsychiatric conditions including aggression.

Finally, one notable finding is that our ranked gene list highlights 40 top genes (Table 2 and Supplementary Table 6), all of which are involved in neurotransmission, axon guidance, synaptic plasticity, learning and memory, neuronal development, or hormone signaling. Twenty-three of the top genes had reports of KO mice studies, strongly supporting their role in aggression. One particular gene of our interest is *RBFOX1*, a splicing factor important for neuronal development. Interestingly, the protein encoded by *RBFOX1* regulates the expression of 15 of the top 40 ranked genes (the probability of this event is *p* = 3.4*e*−05) [70]. Convergent data from GWAS, neuroimaging genetics, epigenetics, gene expression, and animal models supports *RBFOX1* as a strong candidate for aggression [57]. Furthermore, all 40 genes are highly connected in three functional networks (Fig. 2). Human disease genes tend to interact with each other with higher network connectivity than non-disease genes [71]. Many studies of complex neuropsychiatric disorders have also concluded that disease-causing variants are often clustered in protein-interaction networks with a high degree of connectivity among themselves and that these clustered networks are often enriched with functional pathways relevant to brain functions [72–80]. We observed both characteristics for our top-ranked genes, which strongly supports a multifactorial genetic landscape for aggression and the roles of these top genes in aggression. The clusters formed by the top-ranked genes are not only crucial keys for deciphering molecular mechanisms underlying the pathophysiology of aggression; they may also harbor useful therapeutic targets.

We noted that non-genetic models, such as stress-induced aggression [27, 81] and other organisms [30, 31, 82], were not included in this study. However, some correspondences with our findings are worth mentioning (details in Supplementary File 1). These consistencies support the utility of a cross-species approach like ours for identifying genetic mechanisms that are evolutionarily conserved and that may underlie gene-by-environment interactions.

Our approach inherits the limitations of the original studies. GWAS were underpowered and the resulting gene sets may include many false positives. KO mice studies are biased by authors’ choices. For example, genes reported to cause aggression in OMIM disorders may be more likely selected for gene KO in animal models. Although these KO models validate the role of these genes in aggression, it limits our ability to understand the true degree of overlap. The rodent genetic models had been defined by simple behavioral criteria, which may not be generalizable to other species. Improving aggression studies, for example by increasing GWAS sample sizes or by building a repository of behavioral phenotypes for gene KOs in mice, could certainly improve accuracy and decrease the noise in integrative studies like ours. Including other species and model organisms may also provide additional insights; however, difficulty remains regarding gene orthology and generalization of behaviors across distant species.

In summary, we integrated genomic and transcriptomic studies from different species and provided valuable insights into the complex genetic signatures that underlie aggression in both humans and rodent models. Our ranked lists of genes and pathways provide guidance for functional studies in the future.

## Electronic supplementary material

Supplementary Figure Legends and File Lists

Supplementary Table 1

Supplementary Table 2

Supplementary Table 3

Supplementary Table 4

Supplementary Table 5

Supplementary Table 6

Supplementary Table 7

Supplementary Figure 1

Supplementary Figure 2

Supplementary Figure 3

Supplementary Figure 4

Supplementary Figure 5

Supplementary Figure 6

Supplementary File 1

Supplementary Figure 2
