# Supplementary material for: An integrated analysis of genes and functional pathways for aggression in human and rodent models
Source: Mol Psychiatry. 2018 Jun 1;24(11):1655–67. doi: 10.1038/s41380-018-0068-7 (PMC6274606; doi:10.1038/s41380-018-0068-7)
Supplement: Supplementary file 1 — Supplementary Figure Legends and File Lists [file 41380_2018_68_MOESM1_ESM.docx]

# Supplementary Figure Legends

**Supplementary Figure 1. GWAS gene selection.** . We included four studies for the adult GWAS gene set and five for the child gene set. We requested summary statistics for SNPs associated at P ≤ 5e-05, except for one study by Pappa et al. (2016), which was a meta-analysis of 9 different cohorts and had a large sample size (~19,000 samples). We considered a lower threshold for suggestive associations of p ≤ 5e-04 for this study. For Brevik et al. (2016), we only considered associations identified in the meta-analyses of adult samples, since their child samples had already been included in the study by Aebi el al. (2016). For Merjonen et al. (2011), we only considered signals associated with anger (mean values across the four measures), leaving associations with cynicism and paranoia out. For Mick et al. (2014), we included associations with angry temperament and reaction measures. For Anney et al. (2008), we only used the additive model. For Viding et al. (2010), a list of top association signals was not available, but since the study included the results of a follow-up sample, we considered the top 30 replicated SNPs. Using these criteria, a total of 2,143 signals were selected across the nine GWAS and information about nearby genes for each of those SNPs was retrieved using PLINK software with a window of 100 kb. When the window included more than one gene, only the one closest to the SNP was selected.

**Supplementary Figure 2.** **LD score regression analysis to identify genetic correlations between aggression and six psychiatric disorders.** The two largest GWAS of aggression were used to calculate genetic correlation with the six psychiatric disorders: ADHD, MDD, SCZ, ASD, BIP and PTSD. The results for the largest children GWAS sample of aggression, the EAGLE (EArly Genetics and Lifecourse Epidemiology Consortium) study (Pappa et al. 2016) versus the psychiatric disorders are plotted in (**A**). We found a significant correlation with ADHD. The GWAS of broad spectrum of antisocial behavior (Tielbeek et al. 2017) versus the psychiatric dirsorders are plotted in (**B**). This study has both adult (64%) and children (36%) samples. We found a significant genetic correlation with ADHD and MDD.

**Supplementary Figure 3.** **Eigengene expression plotted for modules that were significant for aggression status.** **A.** Eigengene expression for rat module ME6. Benjamini-Hochberg p < .05, total of 18 tests. **B.** ME11 for USA mouse lines. Benjamini-Hochberg adjusted p < .05; total of 30 tests. **C.** ME12 for Finland mouse lines was nominally significantly associated with aggressiveness, but did not survive multiple testing correction (uncorrected p < .005). **D.** Two gene modules for Holland mouse lines, ME22 and ME29, both showed a significant association with aggressiveness (Benjamini-Hochberg adjusted p < .05; total of 35 tests.

**Supplementary Figure 4.** **Canonical pathway enrichment similarities for gene sets were visualized in a 4D MDS configuration plot.** The axes correspond to the first three dimensions. The fourth dimension corresponds to the size of the dots. We observe that both human GWAS and OMIM genes and all three mice models were closely clustered. The rat model, the KO mice genes and GWAS control genes were distinctly apart.

(Note: this image will be linked to a .gif file (Supplementary File 2) to show a rotating 3D plot, which better differentiates the spatial relationships.)

**Supplementary Figure 5.** Canonical pathway activation/inhibition Z-scores were shown as a heatmap to comtrast the USA mice and the rat models. Orange colors are positive Z-scores indicating the overal activation of the pathways; and blues are negative Z-scores indicating the inhibition of the pathways.

**Supplementary Figure 6.** **Violin plots of the network degree (i.e., the numbers of connections) of the top 40 genes in logarithm scales.** Rodent genes are those from the mice/rat transcriptomic and KO mice studies. Human genes include those from the OMIM list and GWAS studies. 14 genes were only from rodent studies, 10 genes were only from human studies, and 16 were from studies of both species. Network degree, i.e. the total numbers of interactions with other genes/molecules in the top 3 IPA networks were quantified. Aggression genes from human studies have higher degree than those rodent_only genes. The differences were statistically significant for human_only genes (F_(1, 22)_ = 10.59, p = 0.004) and genes from both human and rodent studies(F_(1, 28)_ = 4.97, p = 0.034). There was no difference between human genes with or without animal evidence.

**Supplementary file 1:** 1) Details of WGCNA methods; 2) Additional information: correspondence of our gene sets with other models and organisms.

**Supplementary file 2:** Rotating 4D plot of MDS configuration of canonical pathway enrichments.
