# Supplementary material for: An integrated analysis of genes and functional pathways for aggression in human and rodent models
Source: Mol Psychiatry. 2018 Jun 1;24(11):1655–67. doi: 10.1038/s41380-018-0068-7 (PMC6274606; doi:10.1038/s41380-018-0068-7)
Supplement: Supplementary file 2 — Supplementary Table 1 [file 41380_2018_68_MOESM2_ESM.doc]

**Supplementary Table 1:** SNPs associated with aggressive behaviours in GWAS. Selected 2,143 signals by significance.

| SNP | Chr | bp | P-value | P-value Replica | Closest gene* | Distance (kb) | Phenotype | GWAS study |
| --- | --- | --- | --- | --- | --- | --- | --- | --- |
|  |  |  |  |  |  |  |  |  |
| rs140943213 | 1 | 3005246 | 4,81E-05 | - | *PRDM16* | intragenic | Aggressive behavior | Brevik et al., 2016 |
| rs150014345 | 1 | 3008795 | 2,93E-05 | - | *PRDM16* | intragenic | Aggressive behavior | Brevik et al., 2016 |
| rs144697046 | 1 | 3524435 | 1,84E-05 | - | *MEGF6* | 6,5 | Aggressive behavior | Brevik et al., 2016 |
| rs3789562 | 1 | 6026616 | 4.5E-05 | - | *KCNAB2* | intragenic | Anger | Merjonen et al., 2011 |
| rs6694431 | 1 | 14767165 | 3,44E-05 | - | *KIAA1026* | 30,6 | Aggressive behavior | Brevik et al., 2016 |
| rs55633715 | 1 | 14767530 | 3,80E-05 | - | *KIAA1026* | 30,3 | Aggressive behavior | Brevik et al., 2016 |
| rs140424004 | 1 | 14768544 | 3,88E-05 | - | *KIAA1026* | 29,3 | Aggressive behavior | Brevik et al., 2016 |
| rs56370828 | 1 | 14772332 | 4,90E-05 | - | *KIAA1026* | 25,5 | Aggressive behavior | Brevik et al., 2016 |
| rs6666337 | 1 | 14772941 | 4,27E-05 | - | *KIAA1026* | 24,9 | Aggressive behavior | Brevik et al., 2016 |
| rs12040354 | 1 | 15300388 | 3.0E-05 | - | *KIAA1026* | intragenic | CBCL-DP | Mick et al., 2011 |
| rs1567102 | 1 | 20317060 | 4,87E-04 | *-* | *PLA2G2D* | intragenic | Aggressive behavior | Pappa et al., 2016 |
| rs6696025 | 1 | 26490361 | 9,79E-05 | *-* | *UBXD5* | intragenic | Aggressive behavior | Pappa et al., 2016 |
| rs12404062 | 1 | 26540983 | 1,87E-04 | *-* | *AIM1L* | intragenic | Aggressive behavior | Pappa et al., 2016 |
| rs10799071 | 1 | 34821839 | 4.3E-05 | - | *-* | - | Antisocial behavior | Tielbeek et al., 2012 |
| rs728449 | 1 | 34830586 | 3.3E-05 | - | *-* | - | Antisocial behavior | Tielbeek et al., 2012 |
| rs728447 | 1 | 34830735 | 3.3E-05 | - | *-* | - | Antisocial behavior | Tielbeek et al., 2012 |
| rs6661888 | 1 | 41054182 | 3.4E-05 | - | *KCNQ4* | intragenic | Anger (temper) | Mick et al., 2014 |
| rs3889128 | 1 | 53781821 | 3.7E-05 | - | *GLIS1* | intragenic | Anger (temper) | Mick et al., 2014 |
| rs72664414 | 1 | 55512672 | 5,89E-06 | - | *USP24* | 59,4 | Aggressive behavior | Brevik et al., 2016 |
| rs1337740 | 1 | 60317006 | 2,60E-04 | *-* | *C1orf87* | 5,0 | Aggressive behavior | Pappa et al., 2016 |
| rs1337741 | 1 | 60317024 | 4,34E-04 | *-* | *C1orf87* | 5,0 | Aggressive behavior | Pappa et al., 2016 |
| rs10889169 | 1 | 60322765 | 3,58E-04 | *-* | *C1orf87* | 10,7 | Aggressive behavior | Pappa et al., 2016 |
| rs2376016 | 1 | 65806584 | 3.4E-03 | 2.5E-03 | *LEPR* | intragenic | AB+/CU+ | Viding et al., 2010 |
| rs12035080 | 1 | 70098795 | 1.8E-05 | - | *LRRC7* | intragenic | CBCL-DP | Mick et al., 2011 |
| rs17131060 | 1 | 70102187 | 1.4E-05 | - | *LRRC7* | intragenic | CBCL-DP | Mick et al., 2011 |
| rs12037173 | 1 | 70128569 | 4.0E-06 | - | *LRRC7* | intragenic | CBCL-DP | Mick et al., 2011 |
| rs17097465 | 1 | 70171770 | 1.2E-05 | - | *LRRC7* | intragenic | CBCL-DP | Mick et al., 2011 |
| rs11210395 | 1 | 74250927 | 2,58E-05 | - | *LRRIQ3* | 13,4 | Aggressive behavior | Brevik et al., 2016 |
| rs4649996 | 1 | 74292867 | 4,80E-05 | - | *LRRIQ3* | intragenic | Aggressive behavior | Brevik et al., 2016 |
| rs1483793 | 1 | 74307718 | 4,27E-05 | - | *LRRIQ3* | intragenic | Aggressive behavior | Brevik et al., 2016 |
| rs7550195 | 1 | 74334893 | 2,66E-05 | - | *LRRIQ3* | intragenic | Aggressive behavior | Brevik et al., 2016 |
| rs186136551 | 1 | 74338488 | 3,87E-05 | - | *LRRIQ3* | intragenic | Aggressive behavior | Brevik et al., 2016 |
| rs10489950 | 1 | 78614853 | 4,07E-04 | *-* | *-* | - | Aggressive behavior | Pappa et al., 2016 |
| rs10782764 | 1 | 81767466 | 3,57E-05 | - | *-* | - | ODD | Aebi et al., 2016 |
| rs11806028 | 1 | 81768458 | 3,57E-05 | - | *-* | - | ODD | Aebi et al., 2016 |
| rs4650372 | 1 | 81772886 | 4,99E-05 | - | *-* | - | ODD | Aebi et al., 2016 |
| rs113396462 | 1 | 82080292 | 3,07E-05 | - | *LPHN2* | intragenic | Aggressive behavior | Brevik et al., 2016 |
| rs17108253 | 1 | 82602056 | 4,49E-04 | *-* | *-* | - | Aggressive behavior | Pappa et al., 2016 |
| rs7531603 | 1 | 83018311 | 5.0E-03 | 1.3E-03 | *-* | - | AB+/CU+ | Viding et al., 2010 |
| rs4658331 | 1 | 90069372 | 3,40E-04 | *-* | *LRRC8D* | intragenic | Aggressive behavior | Pappa et al., 2016 |
| rs6604060 | 1 | 92101183 | 3,30E-04 | *-* | *TGFBR3* | intragenic | Aggressive behavior | Pappa et al., 2016 |
| rs6604061 | 1 | 92101201 | 3,85E-04 | *-* | *TGFBR3* | intragenic | Aggressive behavior | Pappa et al., 2016 |
| rs6684753 | 1 | 92102604 | 2,31E-04 | *-* | *TGFBR3* | intragenic | Aggressive behavior | Pappa et al., 2016 |
| rs12124897 | 1 | 92104488 | 2,27E-04 | *-* | *TGFBR3* | intragenic | Aggressive behavior | Pappa et al., 2016 |
| rs2039615 | 1 | 95517923 | 2,05E-04 | *-* | *RWDD3* | 32,5 | Aggressive behavior | Pappa et al., 2016 |
| rs12067010 | 1 | 101047521 | 3.5E-05 | - | *EXTL2* | 63.0 | Anger | Merjonen et al., 2011 |
| rs9440550 | 1 | 109194424 | 4,90E-04 | *-* | *C1orf62* | intragenic | Aggressive behavior | Pappa et al., 2016 |
| rs12411132 | 1 | 112279235 | 3.4E-03 | 9.3E-04 | *KCND3* | intragenic | AB+/CU+ | Viding et al., 2010 |
| rs75693843 | 1 | 116834581 | 1,65E-05 | - | *CD58* | 24,1 | Aggressive behavior | Brevik et al., 2016 |
| rs12033744 | 1 | 149227552 | 1,12E-04 | *-* | *ANXA9* | intragenic | Aggressive behavior | Pappa et al., 2016 |
| rs924088 | 1 | 150408711 | 4,63E-04 | *-* | *RPTN* | 10,4 | Aggressive behavior | Pappa et al., 2016 |
| rs11265622 | 1 | 152718044 | 3,31E-04 | *-* | *SHE* | 0,5 | Aggressive behavior | Pappa et al., 2016 |
| rs12568083 | 1 | 152722573 | 3,05E-04 | *-* | *SHE* | intragenic | Aggressive behavior | Pappa et al., 2016 |
| rs4478801 | 1 | 152731196 | 3,02E-04 | *-* | *SHE* | intragenic | Aggressive behavior | Pappa et al., 2016 |
| rs6684921 | 1 | 152731569 | 3,01E-04 | *-* | *SHE* | intragenic | Aggressive behavior | Pappa et al., 2016 |
| rs6700296 | 1 | 152740284 | 2,69E-04 | *-* | *SHE* | intragenic | Aggressive behavior | Pappa et al., 2016 |
| rs5018567 | 1 | 152746942 | 2,89E-04 | *-* | *TDRD10* | intragenic | Aggressive behavior | Pappa et al., 2016 |
| rs7518694 | 1 | 152751412 | 2,30E-04 | *-* | *TDRD10* | intragenic | Aggressive behavior | Pappa et al., 2016 |
| rs12128408 | 1 | 152755157 | 2,81E-04 | *-* | *TDRD10* | intragenic | Aggressive behavior | Pappa et al., 2016 |
| rs4845638 | 1 | 152756893 | 2,78E-04 | *-* | *TDRD10* | intragenic | Aggressive behavior | Pappa et al., 2016 |
| rs4845639 | 1 | 152756976 | 2,75E-04 | *-* | *TDRD10* | intragenic | Aggressive behavior | Pappa et al., 2016 |
| rs10908843 | 1 | 152761104 | 2,40E-04 | *-* | *TDRD10* | intragenic | Aggressive behavior | Pappa et al., 2016 |
| rs4584384 | 1 | 152762321 | 2,30E-04 | *-* | *TDRD10* | intragenic | Aggressive behavior | Pappa et al., 2016 |
| rs4845642 | 1 | 152764652 | 2,54E-04 | *-* | *TDRD10* | intragenic | Aggressive behavior | Pappa et al., 2016 |
| rs12037271 | 1 | 152774512 | 2,12E-04 | *-* | *TDRD10* | intragenic | Aggressive behavior | Pappa et al., 2016 |
| rs12136771 | 1 | 152778126 | 2,64E-04 | *-* | *TDRD10* | intragenic | Aggressive behavior | Pappa et al., 2016 |
| rs883425 | 1 | 155235682 | 1,31E-04 | *-* | *ARHGEF11* | intragenic | Aggressive behavior | Pappa et al., 2016 |
| rs967072 | 1 | 155239109 | 1,47E-04 | *-* | *ARHGEF11* | intragenic | Aggressive behavior | Pappa et al., 2016 |
| rs1359647 | 1 | 155303679 | 4,47E-05 | *-* | *ARHGEF11* | 21,9 | Aggressive behavior | Pappa et al., 2016 |
| rs6678555 | 1 | 155316017 | 1,08E-04 | *-* | *ETV3L* | 12,4 | Aggressive behavior | Pappa et al., 2016 |
| rs17412946 | 1 | 155318425 | 1,44E-04 | *-* | *ETV3L* | 10,0 | Aggressive behavior | Pappa et al., 2016 |
| rs6427356 | 1 | 155397190 | 4.7E-05 | - | *ETV3* | 22.4 | CD | Anney et al., 2008 |
| rs12118751 | 1 | 165361532 | 1,17E-05 | - | *DUSP27* | intragenic | Aggressive behavior | Brevik et al., 2016 |
| rs10798263 | 1 | 169436833 | 3.9E-05 | - | *FMO2* | intragenic | Anger (temper) | Mick et al., 2014 |
| rs10912593 | 1 | 169454681 | 4.8E-05 | - | *FMO2* | 6.2 | Anger (temper) | Mick et al., 2014 |
| rs3753737 | 1 | 179911850 | 2,41E-05 | - | *CACNA1E* | intragenic | Aggressive behavior | Brevik et al., 2016 |
| rs4652673 | 1 | 179931792 | 2,36E-05 | - | *CACNA1E* | intragenic | Aggressive behavior | Brevik et al., 2016 |
| rs4652675 | 1 | 179939026 | 2,73E-05 | - | *CACNA1E* | intragenic | Aggressive behavior | Brevik et al., 2016 |
| rs3845444 | 1 | 179941514 | 1,81E-05 | - | *CACNA1E* | intragenic | Aggressive behavior | Brevik et al., 2016 |
| rs16852155 | 1 | 201926973 | 4,59E-05 | - | *ATP2B4* | intragenic | Aggressive behavior | Brevik et al., 2016 |
| rs1890334 | 1 | 201932484 | 3,81E-05 | - | *ATP2B4* | intragenic | Aggressive behavior | Brevik et al., 2016 |
| rs12405255 | 1 | 201934420 | 3,39E-05 | - | *ATP2B4* | intragenic | Aggressive behavior | Brevik et al., 2016 |
| rs116785145 | 1 | 214097851 | 3,55E-05 | - | *USH2A* | intragenic | Aggressive behavior | Brevik et al., 2016 |
| rs6666194 | 1 | 214739428 | 4,49E-04 | *-* | *ESRRG* | 3,8 | Aggressive behavior | Pappa et al., 2016 |
| rs17659699 | 1 | 214739949 | 4,44E-04 | *-* | *ESRRG* | 3,3 | Aggressive behavior | Pappa et al., 2016 |
| rs12029460 | 1 | 215131484 | 4,91E-04 | *-* | *ESRRG* | intragenic | Aggressive behavior | Pappa et al., 2016 |
| rs12035921 | 1 | 218447442 | 4.3E-05 | - | *RAB3GAP2* | intragenic | Anger (temper) | Mick et al., 2014 |
| rs2456337 | 1 | 223296489 | 2.8E-05 | - | *C1orf67* | 74.2 | Anger | Merjonen et al., 2011 |
| rs12039184 | 1 | 224028565 | 3.8E-05 | - | *SRP9* | 3.6 | Anger (temper) | Mick et al., 2014 |
| rs2234698 | 1 | 224086123 | 3.0E-05 | - | *EPHX1* | intragenic | Antisocial behavior | Tielbeek et al., 2012 |
| rs12737863 | 1 | 224299061 | 6,38E-06 | - | *H3F3A* | 18,0 | Aggressive behavior | Brevik et al., 2016 |
| rs12752329 | 1 | 224384687 | 7,16E-06 | - | *ACBD3* | 14,3 | Aggressive behavior | Brevik et al., 2016 |
| rs9659351 | 1 | 224423563 | 4,06E-05 | - | *ACBD3* | intragenic | Aggressive behavior | Brevik et al., 2016 |
| rs4376699 | 1 | 224424733 | 1,72E-05 | - | *ACBD3* | intragenic | Aggressive behavior | Brevik et al., 2016 |
| rs853017 | 1 | 228706357 | 4,52E-04 | *-* | *-* | - | Aggressive behavior | Pappa et al., 2016 |
| rs12036759 | 1 | 230992064 | 4,19E-05 | - | *KIAA1383* | 15,2 | ODD | Aebi et al., 2016 |
| rs12042052 | 1 | 231002342 | 4,29E-05 | - | *KIAA1383* | 4,9 | ODD | Aebi et al., 2016 |
| rs10910623 | 1 | 231005322 | 3,88E-05 | - | *KIAA1383* | 1,9 | ODD | Aebi et al., 2016 |
| rs72752670 | 1 | 237419616 | 3,06E-05 | - | *-* | - | Aggressive behavior | Brevik et al., 2016 |
| rs111430144 | 1 | 237434785 | 4,21E-05 | - | *-* | - | Aggressive behavior | Brevik et al., 2016 |
| rs72752679 | 1 | 237474631 | 3,34E-05 | - | *-* | - | Aggressive behavior | Brevik et al., 2016 |
| rs72752681 | 1 | 237500495 | 7,78E-06 | - | *-* | - | Aggressive behavior | Brevik et al., 2016 |
| rs2802723 | 1 | 241564935 | 4,58E-04 | *-* | *SDCCAG8* | intragenic | Aggressive behavior | Pappa et al., 2016 |
| rs2802722 | 1 | 241565156 | 3,95E-04 | *-* | *SDCCAG8* | intragenic | Aggressive behavior | Pappa et al., 2016 |
| rs2783964 | 1 | 241567287 | 4,52E-04 | *-* | *SDCCAG8* | intragenic | Aggressive behavior | Pappa et al., 2016 |
| rs2636319 | 1 | 241567670 | 4,45E-04 | *-* | *SDCCAG8* | intragenic | Aggressive behavior | Pappa et al., 2016 |
| rs2636320 | 1 | 241567679 | 4,65E-04 | *-* | *SDCCAG8* | intragenic | Aggressive behavior | Pappa et al., 2016 |
| rs2941874 | 2 | 323665 | 4,46E-04 | *-* | *FAM150B* | 45,4 | Aggressive behavior | Pappa et al., 2016 |
| rs11687356 | 2 | 5719168 | 2.6E-05 | - | *SOX11* | 31.1 | Anger | Merjonen et al., 2011 |
| rs4668497 | 2 | 6517422 | 4.4E-06 | - | *-* | - | Anger | Merjonen et al., 2011 |
| rs2882650 | 2 | 6517472 | 4.3E-06 | - | *-* | - | Anger | Merjonen et al., 2011 |
| rs931007 | 2 | 6517628 | 5.3E-06 | - | *-* | - | Anger | Merjonen et al., 2011 |
| rs10929436 | 2 | 6518881 | 4.3E-06 | - | *-* | - | Anger | Merjonen et al., 2011 |
| rs7593230 | 2 | 6519359 | 4.4E-06 | - | *-* | - | Anger | Merjonen et al., 2011 |
| rs2045722 | 2 | 6519738 | 1.2E-05 | - | *-* | - | Anger | Merjonen et al., 2011 |
| rs10929438 | 2 | 6522878 | 4.7E-06 | - | *-* | - | Anger | Merjonen et al., 2011 |
| rs1462061 | 2 | 6524238 | 5.1E-06 | - | *-* | - | Anger | Merjonen et al., 2011 |
| rs306169 | 2 | 6528852 | 5.8E-06 | - | *-* | - | Anger | Merjonen et al., 2011 |
| rs4669073 | 2 | 6533229 | 5.6E-06 | - | *-* | - | Anger | Merjonen et al., 2011 |
| rs9678581 | 2 | 6533590 | 5.6E-06 | - | *-* | - | Anger | Merjonen et al., 2011 |
| rs12475855 | 2 | 6539922 | 3.4E-05 | - | *-* | - | Anger | Merjonen et al., 2011 |
| rs413642 | 2 | 6561357 | 3.5E-05 | - | *-* | - | Anger | Merjonen et al., 2011 |
| rs307992 | 2 | 6769427 | 3.4E-05 | - | *-* | - | CBCL-DP | Mick et al., 2011 |
| rs382759 | 2 | 6778735 | 4.1E-05 | - | *-* | - | CBCL-DP | Mick et al., 2011 |
| rs308022 | 2 | 6790807 | 3.9E-05 | - | *-* | - | CBCL-DP | Mick et al., 2011 |
| rs308027 | 2 | 6791903 | 4.2E-05 | - | *-* | - | CBCL-DP | Mick et al., 2011 |
| rs62129892 | 2 | 9878424 | 4,64E-05 | - | *TAF1B* | 22,6 | Aggressive behavior | Brevik et al., 2016 |
| rs72782699 | 2 | 9978844 | 2,45E-05 | - | *TAF1B* | intragenic | Aggressive behavior | Brevik et al., 2016 |
| rs4669507 | 2 | 10000460 | 4,06E-05 | - | *TAF1B* | 8,5 | Aggressive behavior | Brevik et al., 2016 |
| rs973436 | 2 | 17074205 | 4,14E-04 | *-* | *-* | - | Aggressive behavior | Pappa et al., 2016 |
| rs16983088 | 2 | 17079959 | 4,06E-04 | *-* | *-* | - | Aggressive behavior | Pappa et al., 2016 |
| rs1878370 | 2 | 18322563 | 4,22E-04 | *-* | *-* | - | Aggressive behavior | Pappa et al., 2016 |
| rs7600488 | 2 | 18323642 | 4,28E-04 | *-* | *-* | - | Aggressive behavior | Pappa et al., 2016 |
| rs7584266 | 2 | 18323871 | 4,33E-04 | *-* | *-* | - | Aggressive behavior | Pappa et al., 2016 |
| rs7557320 | 2 | 18324087 | 4,35E-04 | *-* | *-* | - | Aggressive behavior | Pappa et al., 2016 |
| rs7598323 | 2 | 18324225 | 4,37E-04 | *-* | *-* | - | Aggressive behavior | Pappa et al., 2016 |
| rs7560142 | 2 | 18328147 | 4,51E-04 | *-* | *-* | - | Aggressive behavior | Pappa et al., 2016 |
| rs4036260 | 2 | 18332319 | 4,56E-04 | *-* | *-* | - | Aggressive behavior | Pappa et al., 2016 |
| rs16984640 | 2 | 18345717 | 4,15E-04 | *-* | *-* | - | Aggressive behavior | Pappa et al., 2016 |
| rs16985192 | 2 | 18568624 | 4.2E-05 | - | *RDH14* | 30.8 | CBCL-DP | Mick et al., 2011 |
| rs4832606 | 2 | 18586118 | 4.9E-05 | - | *RDH14* | 13.4 | CBCL-DP | Mick et al., 2011 |
| rs12996631 | 2 | 29425803 | 1.9E-05 | - | *ALK* | intragenic | CBCL-DP | Mick et al., 2011 |
| rs13029602 | 2 | 29428379 | 2.8E-05 | - | *ALK* | intragenic | CBCL-DP | Mick et al., 2011 |
| rs12993319 | 2 | 29449863 | 4.5E-05 | - | *ALK* | intragenic | CBCL-DP | Mick et al., 2011 |
| rs4666212 | 2 | 29466718 | 4.7E-05 | - | *ALK* | intragenic | CBCL-DP | Mick et al., 2011 |
| rs6725521 | 2 | 29576103 | 4,75E-05 | - | *ALK* | intragenic | Aggressive behavior | Brevik et al., 2016 |
| rs11677924 | 2 | 29576696 | 4,26E-05 | - | *ALK* | intragenic | Aggressive behavior | Brevik et al., 2016 |
| rs4550626 | 2 | 29577912 | 1,48E-05 | - | *ALK* | intragenic | Aggressive behavior | Brevik et al., 2016 |
| rs12475058 | 2 | 29578628 | 3,73E-05 | - | *ALK* | intragenic | Aggressive behavior | Brevik et al., 2016 |
| rs6734413 | 2 | 29579582 | 3,59E-05 | - | *ALK* | intragenic | Aggressive behavior | Brevik et al., 2016 |
| rs13389641 | 2 | 35470131 | 4,10E-04 | *-* | *-* | - | Aggressive behavior | Pappa et al., 2016 |
| rs13429480 | 2 | 35495036 | 3,42E-04 | *-* | *-* | - | Aggressive behavior | Pappa et al., 2016 |
| rs11694690 | 2 | 35504532 | 4,70E-04 | *-* | *-* | - | Aggressive behavior | Pappa et al., 2016 |
| rs11695945 | 2 | 35504831 | 4,74E-04 | *-* | *-* | - | Aggressive behavior | Pappa et al., 2016 |
| rs6543943 | 2 | 35505949 | 4,43E-04 | *-* | *-* | - | Aggressive behavior | Pappa et al., 2016 |
| rs873699 | 2 | 46981538 | 4,46E-05 | - | *MCFD2* | 1,0 | Aggressive behavior | Brevik et al., 2016 |
| rs35938067 | 2 | 47002414 | 3,10E-05 | - | *MCFD2* | 6,0 | Aggressive behavior | Brevik et al., 2016 |
| rs138155754 | 2 | 47032907 | 4,69E-05 | - | *TTC7A* | intragenic | Aggressive behavior | Brevik et al., 2016 |
| rs151107270 | 2 | 47035414 | 3,27E-05 | - | *TTC7A* | intragenic | Aggressive behavior | Brevik et al., 2016 |
| rs149824053 | 2 | 47035629 | 2,80E-05 | - | *TTC7A* | intragenic | Aggressive behavior | Brevik et al., 2016 |
| rs114700821 | 2 | 47039218 | 2,66E-05 | - | *TTC7A* | intragenic | Aggressive behavior | Brevik et al., 2016 |
| rs114977142 | 2 | 47049608 | 3,85E-05 | - | *TTC7A* | intragenic | Aggressive behavior | Brevik et al., 2016 |
| rs116777406 | 2 | 47049756 | 3,98E-05 | - | *TTC7A* | intragenic | Aggressive behavior | Brevik et al., 2016 |
| rs116131523 | 2 | 47052020 | 3,88E-05 | - | *TTC7A* | intragenic | Aggressive behavior | Brevik et al., 2016 |
| rs79390512 | 2 | 47052550 | 3,87E-05 | - | *TTC7A* | intragenic | Aggressive behavior | Brevik et al., 2016 |
| rs71423932 | 2 | 47056117 | 1,42E-05 | - | *TTC7A* | intragenic | Aggressive behavior | Brevik et al., 2016 |
| rs114356926 | 2 | 47075740 | 2,92E-05 | - | *TTC7A* | intragenic | Aggressive behavior | Brevik et al., 2016 |
| rs17481147 | 2 | 47076941 | 4,94E-05 | - | *TTC7A* | intragenic | Aggressive behavior | Brevik et al., 2016 |
| rs17540621 | 2 | 47083343 | 4,49E-05 | - | *TTC7A* | intragenic | Aggressive behavior | Brevik et al., 2016 |
| rs116079162 | 2 | 47084312 | 4,47E-05 | - | *TTC7A* | intragenic | Aggressive behavior | Brevik et al., 2016 |
| rs115086675 | 2 | 47085751 | 4,42E-05 | - | *TTC7A* | intragenic | Aggressive behavior | Brevik et al., 2016 |
| rs115360269 | 2 | 47086031 | 4,36E-05 | - | *TTC7A* | intragenic | Aggressive behavior | Brevik et al., 2016 |
| rs79562419 | 2 | 47086137 | 4,39E-05 | - | *TTC7A* | intragenic | Aggressive behavior | Brevik et al., 2016 |
| rs148834702 | 2 | 47087224 | 4,34E-05 | - | *TTC7A* | intragenic | Aggressive behavior | Brevik et al., 2016 |
| rs145220423 | 2 | 47087226 | 4,34E-05 | - | *TTC7A* | intragenic | Aggressive behavior | Brevik et al., 2016 |
| rs10193261 | 2 | 47092646 | 4,22E-05 | - | *TTC7A* | intragenic | Aggressive behavior | Brevik et al., 2016 |
| rs143134854 | 2 | 47092996 | 4,22E-05 | - | *TTC7A* | intragenic | Aggressive behavior | Brevik et al., 2016 |
| rs10196993 | 2 | 47093989 | 4,24E-05 | - | *TTC7A* | intragenic | Aggressive behavior | Brevik et al., 2016 |
| rs140684405 | 2 | 54465135 | 3,32E-05 | - | *C2orf73* | 22,9 | Aggressive behavior | Brevik et al., 2016 |
| rs13030887 | 2 | 67939549 | 1,58E-04 | *-* | *-* | - | Aggressive behavior | Pappa et al., 2016 |
| rs1393821 | 2 | 67940460 | 1,29E-04 | *-* | *-* | - | Aggressive behavior | Pappa et al., 2016 |
| rs1393822 | 2 | 67940503 | 1,26E-04 | *-* | *-* | - | Aggressive behavior | Pappa et al., 2016 |
| rs7578047 | 2 | 68433435 | 5.8E-06 | - | *PLEK* | 12.4 | Anger (reaction) | Mick et al., 2014 |
| rs2271836 | 2 | 68913662 | 1,72E-04 | *-* | *ARHGAP25* | 6,2 | Aggressive behavior | Pappa et al., 2016 |
| rs3749133 | 2 | 68915842 | 1,70E-04 | *-* | *ARHGAP25* | 8,4 | Aggressive behavior | Pappa et al., 2016 |
| rs10496166 | 2 | 68917413 | 1,65E-04 | *-* | *ARHGAP25* | 9,9 | Aggressive behavior | Pappa et al., 2016 |
| rs2128 | 2 | 70804528 | 1,03E-04 | *-* | *ADD2* | intragenic | Aggressive behavior | Pappa et al., 2016 |
| rs2129 | 2 | 70804573 | 9,86E-05 | *-* | *ADD2* | intragenic | Aggressive behavior | Pappa et al., 2016 |
| rs3771436 | 2 | 70805083 | 9,53E-05 | *-* | *ADD2* | intragenic | Aggressive behavior | Pappa et al., 2016 |
| rs1030043 | 2 | 70807171 | 8,24E-05 | *-* | *ADD2* | intragenic | Aggressive behavior | Pappa et al., 2016 |
| rs1030044 | 2 | 70807253 | 1,12E-04 | *-* | *ADD2* | intragenic | Aggressive behavior | Pappa et al., 2016 |
| rs7576498 | 2 | 70809136 | 1,16E-04 | *-* | *ADD2* | intragenic | Aggressive behavior | Pappa et al., 2016 |
| rs6744785 | 2 | 77783686 | 5,73E-06 | *-* | *-* | - | Aggressive behavior | Pappa et al., 2016 |
| rs13415782 | 2 | 77784391 | 5,92E-06 | *-* | *-* | - | Aggressive behavior | Pappa et al., 2016 |
| rs13426167 | 2 | 77787031 | 5,88E-06 | *-* | *-* | - | Aggressive behavior | Pappa et al., 2016 |
| rs6757874 | 2 | 77787372 | 5,14E-05 | *-* | *-* | - | Aggressive behavior | Pappa et al., 2016 |
| rs1487035 | 2 | 77789803 | 5,89E-05 | *-* | *-* | - | Aggressive behavior | Pappa et al., 2016 |
| rs1386379 | 2 | 77790090 | 4,45E-04 | *-* | *-* | - | Aggressive behavior | Pappa et al., 2016 |
| rs1487033 | 2 | 77790113 | 4,68E-04 | *-* | *-* | - | Aggressive behavior | Pappa et al., 2016 |
| rs12622166 | 2 | 77791620 | 4,02E-04 | *-* | *-* | - | Aggressive behavior | Pappa et al., 2016 |
| rs12622168 | 2 | 77791633 | 3,99E-04 | *-* | *-* | - | Aggressive behavior | Pappa et al., 2016 |
| rs729756 | 2 | 77793781 | 8,31E-06 | *-* | *-* | - | Aggressive behavior | Pappa et al., 2016 |
| rs17014427 | 2 | 77794783 | 4,32E-06 | *-* | *-* | - | Aggressive behavior | Pappa et al., 2016 |
| rs12613157 | 2 | 77796327 | 9,84E-07 | *-* | *-* | - | Aggressive behavior | Pappa et al., 2016 |
| rs11126630 | 2 | 77796352 | 5,30E-08 | *-* | *-* | - | Aggressive behavior | Pappa et al., 2016 |
| rs1542677 | 2 | 77796867 | 1,01E-06 | *-* | *-* | - | Aggressive behavior | Pappa et al., 2016 |
| rs1542678 | 2 | 77796893 | 1,02E-06 | *-* | *-* | - | Aggressive behavior | Pappa et al., 2016 |
| rs11126631 | 2 | 77798089 | 4,63E-06 | *-* | *-* | - | Aggressive behavior | Pappa et al., 2016 |
| rs994214 | 2 | 77798750 | 4,65E-06 | *-* | *-* | - | Aggressive behavior | Pappa et al., 2016 |
| rs10169036 | 2 | 77800630 | 1,86E-07 | *-* | *-* | - | Aggressive behavior | Pappa et al., 2016 |
| rs10192926 | 2 | 77800959 | 1,48E-06 | *-* | *-* | - | Aggressive behavior | Pappa et al., 2016 |
| rs11676317 | 2 | 77801496 | 3,05E-07 | *-* | *-* | - | Aggressive behavior | Pappa et al., 2016 |
| rs2861212 | 2 | 77859654 | 1,14E-04 | *-* | *-* | - | Aggressive behavior | Pappa et al., 2016 |
| rs7595440 | 2 | 77859858 | 1,39E-04 | *-* | *-* | - | Aggressive behavior | Pappa et al., 2016 |
| rs17015724 | 2 | 78917688 | 2.3E-05 | - | *-* | - | Antisocial behavior | Tielbeek et al., 2012 |
| rs283823 | 2 | 79083507 | 2.3E-05 | - | *REG3G* | 22.8 | Anger | Merjonen et al., 2011 |
| rs283826 | 2 | 79086587 | 3.5E-05 | - | *REG3G* | 19.8 | Anger | Merjonen et al., 2011 |
| rs283828 | 2 | 79087585 | 3.4E-05 | - | *REG3G* | 18.8 | Anger | Merjonen et al., 2011 |
| rs283830 | 2 | 79088155 | 3.4E-05 | - | *REG3G* | 18.2 | Anger | Merjonen et al., 2011 |
| rs283832 | 2 | 79090597 | 3.5E-05 | - | *REG3G* | 15.7 | Anger | Merjonen et al., 2011 |
| rs374425 | 2 | 79091248 | 1.1E-05 | - | *REG3G* | 15.1 | Anger | Merjonen et al., 2011 |
| rs688806 | 2 | 79092724 | 1.0E-05 | - | *REG3G* | 13.6 | Anger | Merjonen et al., 2011 |
| rs405595 | 2 | 79092786 | 9.8E-06 | - | *REG3G* | 13.6 | Anger | Merjonen et al., 2011 |
| rs436569 | 2 | 79092808 | 9.6E-06 | - | *REG3G* | 13.5 | Anger | Merjonen et al., 2011 |
| rs396909 | 2 | 79092910 | 9.4E-06 | - | *REG3G* | 13.4 | Anger | Merjonen et al., 2011 |
| rs397749 | 2 | 79093567 | 9.0E-06 | - | *REG3G* | 12.8 | Anger | Merjonen et al., 2011 |
| rs396619 | 2 | 79093584 | 8.8E-06 | - | *REG3G* | 12.8 | Anger | Merjonen et al., 2011 |
| rs395701 | 2 | 79093857 | 8.7E-06 | - | *REG3G* | 12.5 | Anger | Merjonen et al., 2011 |
| rs417898 | 2 | 79093962 | 8.5E-06 | - | *REG3G* | 12.4 | Anger | Merjonen et al., 2011 |
| rs422782 | 2 | 79094203 | 8.4E-06 | - | *REG3G* | 12.1 | Anger | Merjonen et al., 2011 |
| rs437049 | 2 | 79094364 | 8.1E-06 | - | *REG3G* | 12.0 | Anger | Merjonen et al., 2011 |
| rs561259 | 2 | 79094959 | 8.0E-06 | - | *REG3G* | 11.4 | Anger | Merjonen et al., 2011 |
| rs402329 | 2 | 79095264 | 7.9E-06 | - | *REG3G* | 11.1 | Anger | Merjonen et al., 2011 |
| rs374604 | 2 | 79095618 | 7.7E-06 | - | *REG3G* | 10.7 | Anger | Merjonen et al., 2011 |
| rs374242 | 2 | 79095738 | 7.7E-06 | - | *REG3G* | 10.6 | Anger | Merjonen et al., 2011 |
| rs400861 | 2 | 79095766 | 7.7E-06 | - | *REG3G* | 10.6 | Anger | Merjonen et al., 2011 |
| rs432420 | 2 | 79096077 | 7.7E-06 | - | *REG3G* | 10.3 | Anger | Merjonen et al., 2011 |
| rs425048 | 2 | 79096110 | 7.7E-06 | - | *REG3G* | 10.2 | Anger | Merjonen et al., 2011 |
| rs422871 | 2 | 79096294 | 7.7E-06 | - | *REG3G* | 10.0 | Anger | Merjonen et al., 2011 |
| rs423313 | 2 | 79096462 | 7.1E-06 | - | *REG3G* | 9.9 | Anger | Merjonen et al., 2011 |
| rs434922 | 2 | 79097105 | 7.7E-06 | - | *REG3G* | 9.2 | Anger | Merjonen et al., 2011 |
| rs434372 | 2 | 79097425 | 7.7E-06 | - | *REG3G* | 8.9 | Anger | Merjonen et al., 2011 |
| rs424662 | 2 | 79097559 | 7.7E-06 | - | *REG3G* | 8.8 | Anger | Merjonen et al., 2011 |
| rs430406 | 2 | 79097608 | 7.7E-06 | - | *REG3G* | 8.7 | Anger | Merjonen et al., 2011 |
| rs448043 | 2 | 79097653 | 7.7E-06 | - | *REG3G* | 8.7 | Anger | Merjonen et al., 2011 |
| rs393149 | 2 | 79097715 | 9.4E-06 | - | *REG3G* | 8.6 | Anger | Merjonen et al., 2011 |
| rs409899 | 2 | 79097734 | 7.7E-06 | - | *REG3G* | 8.6 | Anger | Merjonen et al., 2011 |
| rs428676 | 2 | 79098840 | 7.7E-06 | - | *REG3G* | 7.5 | Anger | Merjonen et al., 2011 |
| rs412656 | 2 | 79099070 | 7.7E-06 | - | *REG3G* | 7.3 | Anger | Merjonen et al., 2011 |
| rs435133 | 2 | 79099894 | 7.7E-06 | - | *REG3G* | 6.4 | Anger | Merjonen et al., 2011 |
| rs436049 | 2 | 79100386 | 7.7E-06 | - | *REG3G* | 5.9 | Anger | Merjonen et al., 2011 |
| rs364452 | 2 | 79100901 | 7.6E-06 | - | *REG3G* | 5.4 | Anger | Merjonen et al., 2011 |
| rs436758 | 2 | 79101018 | 7.6E-06 | - | *REG3G* | 5.3 | Anger | Merjonen et al., 2011 |
| rs446160 | 2 | 79101453 | 7.6E-06 | - | *REG3G* | 4.9 | Anger | Merjonen et al., 2011 |
| rs375700 | 2 | 79101757 | 7.5E-06 | - | *REG3G* | 4.6 | Anger | Merjonen et al., 2011 |
| rs426233 | 2 | 79103154 | 7.5E-06 | - | *REG3G* | 3.2 | Anger | Merjonen et al., 2011 |
| rs430298 | 2 | 79103280 | 7.5E-06 | - | *REG3G* | 3.1 | Anger | Merjonen et al., 2011 |
| rs368506 | 2 | 79106269 | 7.5E-06 | - | *REG3G* | 0.1 | Anger | Merjonen et al., 2011 |
| rs412912 | 2 | 79107326 | 7.4E-06 | - | *REG3G* | intragenic | Anger | Merjonen et al., 2011 |
| rs429210 | 2 | 79109883 | 7.4E-06 | - | *REG3G* | 0.7 | Anger | Merjonen et al., 2011 |
| rs993736 | 2 | 83617449 | 2,49E-04 | *-* | *-* | - | Aggressive behavior | Pappa et al., 2016 |
| rs7565040 | 2 | 83637635 | 2,86E-04 | *-* | *-* | - | Aggressive behavior | Pappa et al., 2016 |
| rs13013328 | 2 | 83652487 | 1,50E-04 | *-* | *-* | - | Aggressive behavior | Pappa et al., 2016 |
| rs1386306 | 2 | 83662816 | 1,78E-04 | *-* | *-* | - | Aggressive behavior | Pappa et al., 2016 |
| rs7561888 | 2 | 86246279 | 5.5E-06 | - | *IMMT* | intragenic | Antisocial behavior | Tielbeek et al., 2012 |
| rs17510310 | 2 | 86296719 | 6.6E-06 | - | *REEP1* | intragenic | Antisocial behavior | Tielbeek et al., 2012 |
| rs2970895 | 2 | 88198502 | 3,49E-04 | *-* | *SMYD1* | 4,5 | Aggressive behavior | Pappa et al., 2016 |
| rs1800649 | 2 | 97631289 | 2.9E-05 | - | *COX5B* | 0.2 | CBCL-DP | Mick et al., 2011 |
| rs11123861 | 2 | 97676314 | 2.8E-05 | - | *ZAP70* | 20.2 | CBCL-DP | Mick et al., 2011 |
| rs1192815 | 2 | 101179889 | 1,60E-04 | *-* | *TBC1D8* | 45,6 | Aggressive behavior | Pappa et al., 2016 |
| rs2587695 | 2 | 120038287 | 4.1E-05 | - | *PCDP1* | intragenic | CD | Anney et al., 2008 |
| rs11680911 | 2 | 127605227 | 4,18E-04 | *-* | *BIN1* | 23,9 | Aggressive behavior | Pappa et al., 2016 |
| rs7561528 | 2 | 127606107 | 4,27E-04 | *-* | *BIN1* | 24,8 | Aggressive behavior | Pappa et al., 2016 |
| rs1464297 | 2 | 140370219 | 1.7E-05 | - | *-* | - | Antisocial behavior | Tielbeek et al., 2012 |
| rs9287528 | 2 | 140371591 | 1.3E-05 | - | *-* | - | Antisocial behavior | Tielbeek et al., 2012 |
| rs11901615 | 2 | 140373840 | 1.6E-05 | - | *-* | - | Antisocial behavior | Tielbeek et al., 2012 |
| rs11682518 | 2 | 153932192 | 5.6E-03 | 8.6E-04 | *-* | - | AB+/CU+ | Viding et al., 2010 |
| rs707087 | 2 | 155019555 | 1,62E-05 | - | *GALNT13* | 0,8 | Aggressive behavior | Brevik et al., 2016 |
| rs16837898 | 2 | 155213067 | 4,78E-04 | *-* | *KCNJ3* | 50,3 | Aggressive behavior | Pappa et al., 2016 |
| rs16837918 | 2 | 155223672 | 4,62E-04 | *-* | *KCNJ3* | 39,7 | Aggressive behavior | Pappa et al., 2016 |
| rs16837930 | 2 | 155233617 | 1,22E-04 | *-* | *KCNJ3* | 29,7 | Aggressive behavior | Pappa et al., 2016 |
| rs4571015 | 2 | 155253916 | 1,44E-04 | *-* | *KCNJ3* | 9,4 | Aggressive behavior | Pappa et al., 2016 |
| rs16838005 | 2 | 155255073 | 1,97E-04 | *-* | *KCNJ3* | 8,3 | Aggressive behavior | Pappa et al., 2016 |
| rs11884022 | 2 | 159910201 | 2.0E-05 | - | *BAZ2B* | intragenic | Antisocial behavior | Tielbeek et al., 2012 |
| rs3771684 | 2 | 159910514 | 8.7E-06 | - | *BAZ2B* | intragenic | Antisocial behavior | Tielbeek et al., 2012 |
| rs1267043 | 2 | 161833540 | 1,52E-04 | *-* | *TANK* | 32,6 | Aggressive behavior | Pappa et al., 2016 |
| rs2013879 | 2 | 161884290 | 1,93E-04 | *-* | *PSMD14* | intragenic | Aggressive behavior | Pappa et al., 2016 |
| rs2113480 | 2 | 161894856 | 4,66E-04 | *-* | *PSMD14* | intragenic | Aggressive behavior | Pappa et al., 2016 |
| rs12988044 | 2 | 161901155 | 1,26E-04 | *-* | *PSMD14* | intragenic | Aggressive behavior | Pappa et al., 2016 |
| rs1110587 | 2 | 161972523 | 4,05E-04 | *-* | *PSMD14* | intragenic | Aggressive behavior | Pappa et al., 2016 |
| rs1345959 | 2 | 163536361 | 3.5E-03 | 4.6E-03 | *-* | - | AB+/CU+ | Viding et al., 2010 |
| rs6719629 | 2 | 168527483 | 1,21E-05 | - | *STK39* | intragenic | ODD | Aebi et al., 2016 |
| rs7594207 | 2 | 168544415 | 1,59E-05 | - | *STK39* | intragenic | ODD | Aebi et al., 2016 |
| rs10497332 | 2 | 168548561 | 1,67E-05 | - | *STK39* | intragenic | ODD | Aebi et al., 2016 |
| rs17778577 | 2 | 168549891 | 1,67E-05 | - | *STK39* | intragenic | ODD | Aebi et al., 2016 |
| rs4667545 | 2 | 168556221 | 1,29E-05 | - | *STK39* | intragenic | ODD | Aebi et al., 2016 |
| rs2893070 | 2 | 168557593 | 1,24E-05 | - | *STK39* | intragenic | ODD | Aebi et al., 2016 |
| rs6712239 | 2 | 168557693 | 1,07E-05 | - | *STK39* | intragenic | ODD | Aebi et al., 2016 |
| rs4667995 | 2 | 168560465 | 3,47E-05 | - | *STK39* | intragenic | ODD | Aebi et al., 2016 |
| rs13013931 | 2 | 169840478 | 3,43E-04 | *-* | *LRP2* | intragenic | Aggressive behavior | Pappa et al., 2016 |
| rs12619395 | 2 | 173614287 | 7,41E-05 | *-* | *RAPGEF4* | intragenic | Aggressive behavior | Pappa et al., 2016 |
| rs16861227 | 2 | 173614711 | 7,86E-05 | *-* | *RAPGEF4* | intragenic | Aggressive behavior | Pappa et al., 2016 |
| rs16824241 | 2 | 183849991 | 3,44E-04 | *-* | *-* | - | Aggressive behavior | Pappa et al., 2016 |
| rs3749022 | 2 | 190892769 | 4.2E-05 | - | *HIBCH* | intragenic | Anger | Merjonen et al., 2011 |
| rs16832572 | 2 | 190899093 | 4.9E-05 | - | *HIBCH* | 6.3 | Anger | Merjonen et al., 2011 |
| rs12613768 | 2 | 191101691 | 4.8E-05 | - | *FLJ20160* | 26.4 | Anger | Merjonen et al., 2011 |
| rs12694000 | 2 | 204738545 | 1.8E-05 | - | *-* | - | Antisocial behavior | Tielbeek et al., 2012 |
| rs4419220 | 2 | 204740302 | 2.3E-05 | - | *-* | - | Antisocial behavior | Tielbeek et al., 2012 |
| rs16836750 | 2 | 205509376 | 3.7E-05 | - | *PARD3B* | intragenic | Anger | Merjonen et al., 2011 |
| rs16836754 | 2 | 205509485 | 3.7E-05 | - | *PARD3B* | intragenic | Anger | Merjonen et al., 2011 |
| rs16836757 | 2 | 205509856 | 3.7E-05 | - | *PARD3B* | intragenic | Anger | Merjonen et al., 2011 |
| rs1862995 | 2 | 205510283 | 3.7E-05 | - | *PARD3B* | intragenic | Anger | Merjonen et al., 2011 |
| rs16836762 | 2 | 205510694 | 3.7E-05 | - | *PARD3B* | intragenic | Anger | Merjonen et al., 2011 |
| rs16824690 | 2 | 205511163 | 3.8E-05 | - | *PARD3B* | intragenic | Anger | Merjonen et al., 2011 |
| rs7595624 | 2 | 205512157 | 3.9E-05 | - | *PARD3B* | intragenic | Anger | Merjonen et al., 2011 |
| rs13407207 | 2 | 205512820 | 3.9E-05 | - | *PARD3B* | intragenic | Anger | Merjonen et al., 2011 |
| rs13394155 | 2 | 205520948 | 4.1E-05 | - | *PARD3B* | intragenic | Anger | Merjonen et al., 2011 |
| rs13394306 | 2 | 205521123 | 4.1E-05 | - | *PARD3B* | intragenic | Anger | Merjonen et al., 2011 |
| rs6718456 | 2 | 205521403 | 4.1E-05 | - | *PARD3B* | intragenic | Anger | Merjonen et al., 2011 |
| rs6718708 | 2 | 205521602 | 4.1E-05 | - | *PARD3B* | intragenic | Anger | Merjonen et al., 2011 |
| rs10197984 | 2 | 205532832 | 4.0E-05 | - | *PARD3B* | intragenic | Anger | Merjonen et al., 2011 |
| rs13397257 | 2 | 205534644 | 4.0E-05 | - | *PARD3B* | intragenic | Anger | Merjonen et al., 2011 |
| rs10183528 | 2 | 211860758 | 1,14E-04 | *-* | *ERBB4* | 87,9 | Aggressive behavior | Pappa et al., 2016 |
| rs6727011 | 2 | 215247415 | 2,46E-04 | *-* | *BARD1* | 54,1 | Aggressive behavior | Pappa et al., 2016 |
| rs2568164 | 2 | 218036983 | 4,78E-04 | *-* | *-* | - | Aggressive behavior | Pappa et al., 2016 |
| rs2618141 | 2 | 218044863 | 1,73E-05 | *-* | *-* | - | Aggressive behavior | Pappa et al., 2016 |
| rs1871230 | 2 | 218049053 | 3,22E-04 | *-* | *-* | - | Aggressive behavior | Pappa et al., 2016 |
| rs12472818 | 2 | 218066557 | 1,25E-04 | *-* | *-* | - | Aggressive behavior | Pappa et al., 2016 |
| rs10198000 | 2 | 218073582 | 4,78E-04 | *-* | *-* | - | Aggressive behavior | Pappa et al., 2016 |
| rs10191686 | 2 | 218074976 | 4,83E-04 | *-* | *-* | - | Aggressive behavior | Pappa et al., 2016 |
| rs10191778 | 2 | 218075039 | 4,10E-04 | *-* | *-* | - | Aggressive behavior | Pappa et al., 2016 |
| rs13008340 | 2 | 218098259 | 1,88E-04 | *-* | *-* | - | Aggressive behavior | Pappa et al., 2016 |
| rs12694419 | 2 | 218099262 | 2,46E-05 | *-* | *-* | - | Aggressive behavior | Pappa et al., 2016 |
| rs750365 | 2 | 218099708 | 1,07E-04 | *-* | *-* | - | Aggressive behavior | Pappa et al., 2016 |
| rs11889534 | 2 | 218102220 | 9,13E-05 | *-* | *-* | - | Aggressive behavior | Pappa et al., 2016 |
| rs7582879 | 2 | 218102574 | 1,38E-04 | *-* | *-* | - | Aggressive behavior | Pappa et al., 2016 |
| rs10932846 | 2 | 220819539 | 4.1E-05 | - | *-* |  | Anger (temper) | Mick et al., 2014 |
| rs2396012 | 2 | 224274839 | 4.6E-05 | - | *AP1S3* | 53.5 | Antisocial behavior | Tielbeek et al., 2012 |
| rs7577145 | 2 | 224275527 | 4.7E-05 | - | *AP1S3* | 52.8 | Antisocial behavior | Tielbeek et al., 2012 |
| rs12992947 | 2 | 224276333 | 4.7E-05 | - | *AP1S3* | 52.0 | Antisocial behavior | Tielbeek et al., 2012 |
| rs893451 | 2 | 224278593 | 3.5E-05 | - | *AP1S3* | 49.7 | Antisocial behavior | Tielbeek et al., 2012 |
| rs744396 | 2 | 224279025 | 1.7E-05 | - | *AP1S3* | 49.3 | Antisocial behavior | Tielbeek et al., 2012 |
| rs3754945 | 2 | 230989945 | 1,46E-04 | *-* | *SP100* | intragenic | Aggressive behavior | Pappa et al., 2016 |
| rs1427283 | 2 | 230991035 | 8,94E-05 | *-* | *SP100* | intragenic | Aggressive behavior | Pappa et al., 2016 |
| rs7604350 | 2 | 238754423 | 2,71E-04 | *-* | *ILKAP* | intragenic | Aggressive behavior | Pappa et al., 2016 |
| rs6431587 | 2 | 238754781 | 2,77E-04 | *-* | *ILKAP* | intragenic | Aggressive behavior | Pappa et al., 2016 |
| rs3791556 | 2 | 239777909 | 1,97E-04 | *-* | *HDAC4* | intragenic | Aggressive behavior | Pappa et al., 2016 |
| rs3791568 | 2 | 239780638 | 1,50E-04 | *-* | *HDAC4* | intragenic | Aggressive behavior | Pappa et al., 2016 |
| rs13031713 | 2 | 239783805 | 1,11E-04 | *-* | *HDAC4* | intragenic | Aggressive behavior | Pappa et al., 2016 |
| rs9843857 | 3 | 733920 | 4,65E-04 | *-* | *-* | - | Aggressive behavior | Pappa et al., 2016 |
| rs9883263 | 3 | 738586 | 2,01E-04 | *-* | *-* | - | Aggressive behavior | Pappa et al., 2016 |
| rs4686104 | 3 | 742461 | 1,00E-04 | *-* | *-* | - | Aggressive behavior | Pappa et al., 2016 |
| rs9829368 | 3 | 742659 | 2,33E-04 | *-* | *-* | - | Aggressive behavior | Pappa et al., 2016 |
| rs13095275 | 3 | 2214184 | 2,65E-04 | *-* | *CNTN4* | intragenic | Aggressive behavior | Pappa et al., 2016 |
| rs4685500 | 3 | 2239609 | 2,70E-04 | *-* | *CNTN4* | intragenic | Aggressive behavior | Pappa et al., 2016 |
| rs6808972 | 3 | 2748738 | 2,84E-04 | *-* | *CNTN4* | intragenic | Aggressive behavior | Pappa et al., 2016 |
| rs12635996 | 3 | 2752017 | 4,85E-04 | *-* | *CNTN4* | intragenic | Aggressive behavior | Pappa et al., 2016 |
| rs4685543 | 3 | 2754566 | 4,55E-04 | *-* | *CNTN4* | intragenic | Aggressive behavior | Pappa et al., 2016 |
| rs7648792 | 3 | 2755113 | 2,71E-04 | *-* | *CNTN4* | intragenic | Aggressive behavior | Pappa et al., 2016 |
| rs9823500 | 3 | 2758344 | 4,68E-04 | *-* | *CNTN4* | intragenic | Aggressive behavior | Pappa et al., 2016 |
| rs7648378 | 3 | 3339182 | 1.1E-05 | - | *-* | - | CD | Anney et al., 2008 |
| rs9853191 | 3 | 9145534 | 4,52E-04 | *-* | *SRGAP3* | intragenic | Aggressive behavior | Pappa et al., 2016 |
| rs7640807 | 3 | 15660141 | 4.7E-03 | 3.1E-03 | *BTD* | intragenic | AB+/CU+ | Viding et al., 2010 |
| rs2366681 | 3 | 20972617 | 2,99E-04 | *-* | *-* | - | Aggressive behavior | Pappa et al., 2016 |
| rs1878012 | 3 | 21822622 | 3.8E-05 | - | *ZNF385D* | 54.8 | Anger (reaction) | Mick et al., 2014 |
| rs9867006 | 3 | 25336856 | 1,06E-05 | - | *-* | - | ODD | Aebi et al., 2016 |
| rs9877461 | 3 | 25338624 | 1,69E-05 | - | *-* | - | ODD | Aebi et al., 2016 |
| rs9822411 | 3 | 25338947 | 5,29E-06 | - | *-* | - | ODD | Aebi et al., 2016 |
| rs17016187 | 3 | 25339293 | 1,23E-05 | - | *-* | - | ODD | Aebi et al., 2016 |
| rs9833653 | 3 | 25344352 | 5,29E-06 | - | *-* | - | ODD | Aebi et al., 2016 |
| rs7649070 | 3 | 25352790 | 2,25E-05 | - | *RARB* | 92,0 | ODD | Aebi et al., 2016 |
| rs4955203 | 3 | 31821255 | 4.2E-05 | - | *OSBPL10* | intragenic | Anger | Merjonen et al., 2011 |
| rs4955113 | 3 | 31821481 | 4.9E-05 | - | *OSBPL10* | intragenic | Anger | Merjonen et al., 2011 |
| rs4611855 | 3 | 31822364 | 4.2E-05 | - | *OSBPL10* | intragenic | Anger | Merjonen et al., 2011 |
| rs7632391 | 3 | 40055548 | 4,18E-04 | *-* | *MYRIP* | intragenic | Aggressive behavior | Pappa et al., 2016 |
| rs13098603 | 3 | 47509113 | 4,10E-04 | *-* | *TMEM103* | 3,0 | Aggressive behavior | Pappa et al., 2016 |
| rs12639503 | 3 | 51835857 | 2.6E-05 | - | *IQCF3* | 1.8 | Anger (reaction) | Mick et al., 2014 |
| rs9866552 | 3 | 51839889 | 4.2E-05 | - | *IQCF3* | intragenic | Anger (reaction) | Mick et al., 2014 |
| rs7644325 | 3 | 52056176 | 3,83E-05 | *-* | *DUSP7* | 3,6 | Aggressive behavior | Pappa et al., 2016 |
| rs4687607 | 3 | 52063691 | 3,57E-05 | *-* | *DUSP7* | intragenic | Aggressive behavior | Pappa et al., 2016 |
| rs9851576 | 3 | 52066294 | 4,47E-05 | *-* | *DUSP7* | 1,0 | Aggressive behavior | Pappa et al., 2016 |
| rs13319566 | 3 | 52070595 | 2,26E-04 | *-* | *LOC100128378* | 0,6 | Aggressive behavior | Pappa et al., 2016 |
| rs17051957 | 3 | 52074481 | 2,32E-04 | *-* | *LOC100128378* | 0,3 | Aggressive behavior | Pappa et al., 2016 |
| rs12496447 | 3 | 52078827 | 5,88E-05 | *-* | *LOC100128378* | 4,7 | Aggressive behavior | Pappa et al., 2016 |
| rs9865330 | 3 | 52080965 | 2,00E-04 | *-* | *WDR51A* | 3,3 | Aggressive behavior | Pappa et al., 2016 |
| rs876782 | 3 | 52082949 | 3,63E-04 | *-* | *WDR51A* | 1,4 | Aggressive behavior | Pappa et al., 2016 |
| rs747343 | 3 | 52084447 | 4,63E-05 | *-* | *WDR51A* | intragenic | Aggressive behavior | Pappa et al., 2016 |
| rs893056 | 3 | 52084604 | 3,21E-04 | *-* | *WDR51A* | intragenic | Aggressive behavior | Pappa et al., 2016 |
| rs9857878 | 3 | 52084708 | 2,51E-04 | *-* | *WDR51A* | intragenic | Aggressive behavior | Pappa et al., 2016 |
| rs9809254 | 3 | 52086514 | 3,36E-04 | *-* | *WDR51A* | intragenic | Aggressive behavior | Pappa et al., 2016 |
| rs13314125 | 3 | 52090438 | 3,02E-04 | *-* | *WDR51A* | intragenic | Aggressive behavior | Pappa et al., 2016 |
| rs9828327 | 3 | 52095249 | 6,23E-05 | *-* | *WDR51A* | intragenic | Aggressive behavior | Pappa et al., 2016 |
| rs9846049 | 3 | 52095254 | 3,34E-04 | *-* | *WDR51A* | intragenic | Aggressive behavior | Pappa et al., 2016 |
| rs4687805 | 3 | 52098353 | 6,87E-05 | *-* | *WDR51A* | intragenic | Aggressive behavior | Pappa et al., 2016 |
| rs9843852 | 3 | 52107069 | 3,44E-04 | *-* | *WDR51A* | intragenic | Aggressive behavior | Pappa et al., 2016 |
| rs9868101 | 3 | 52113417 | 3,46E-04 | *-* | *WDR51A* | intragenic | Aggressive behavior | Pappa et al., 2016 |
| rs9847073 | 3 | 52119987 | 3,65E-04 | *-* | *WDR51A* | intragenic | Aggressive behavior | Pappa et al., 2016 |
| rs9817653 | 3 | 52123273 | 3,05E-04 | *-* | *WDR51A* | intragenic | Aggressive behavior | Pappa et al., 2016 |
| rs6778442 | 3 | 52123523 | 1,43E-04 | *-* | *WDR51A* | intragenic | Aggressive behavior | Pappa et al., 2016 |
| rs9882775 | 3 | 52124689 | 3,49E-04 | *-* | *WDR51A* | intragenic | Aggressive behavior | Pappa et al., 2016 |
| rs9809977 | 3 | 52141187 | 3,09E-04 | *-* | *WDR51A* | intragenic | Aggressive behavior | Pappa et al., 2016 |
| rs9841469 | 3 | 52141636 | 3,44E-04 | *-* | *WDR51A* | intragenic | Aggressive behavior | Pappa et al., 2016 |
| rs4687810 | 3 | 52144135 | 7,58E-05 | *-* | *WDR51A* | intragenic | Aggressive behavior | Pappa et al., 2016 |
| rs2334961 | 3 | 52148665 | 7,54E-05 | *-* | *WDR51A* | intragenic | Aggressive behavior | Pappa et al., 2016 |
| rs735768 | 3 | 52150010 | 7,91E-05 | *-* | *WDR51A* | intragenic | Aggressive behavior | Pappa et al., 2016 |
| rs893055 | 3 | 52152388 | 7,61E-05 | *-* | *WDR51A* | intragenic | Aggressive behavior | Pappa et al., 2016 |
| rs17051977 | 3 | 52157984 | 2,99E-04 | *-* | *WDR51A* | intragenic | Aggressive behavior | Pappa et al., 2016 |
| rs2304505 | 3 | 52159800 | 3,17E-04 | *-* | *WDR51A* | intragenic | Aggressive behavior | Pappa et al., 2016 |
| rs6786592 | 3 | 52171281 | 7,60E-05 | *-* | *WDR51A* | 7,8 | Aggressive behavior | Pappa et al., 2016 |
| rs171294 | 3 | 52178639 | 3,27E-04 | *-* | *WDR51A* | 15,2 | Aggressive behavior | Pappa et al., 2016 |
| rs352148 | 3 | 52183938 | 3,58E-04 | *-* | *WDR51A* | 20,5 | Aggressive behavior | Pappa et al., 2016 |
| rs352149 | 3 | 52184570 | 3,53E-04 | *-* | *WDR51A* | 21,1 | Aggressive behavior | Pappa et al., 2016 |
| rs352151 | 3 | 52185312 | 3,55E-04 | *-* | *ALAS1* | 21,8 | Aggressive behavior | Pappa et al., 2016 |
| rs352152 | 3 | 52185740 | 2,80E-05 | *-* | *ALAS1* | 21,4 | Aggressive behavior | Pappa et al., 2016 |
| rs352140 | 3 | 52231737 | 4,83E-04 | *-* | *TLR9* | intragenic | Aggressive behavior | Pappa et al., 2016 |
| rs146735513 | 3 | 56724085 | 4,77E-05 | - | *ARHGEF3* | 12,4 | Aggressive behavior | Brevik et al., 2016 |
| rs1490000 | 3 | 68996086 | 4,75E-04 | *-* | *FAM19A4* | intragenic | Aggressive behavior | Pappa et al., 2016 |
| rs116644287 | 3 | 69822366 | 4,66E-05 | - | *MITF* | 49,0 | Aggressive behavior | Brevik et al., 2016 |
| rs13064369 | 3 | 77621908 | 1.6E-03 | 4.6E-03 | *ROBO2* | intragenic | AB+/CU+ | Viding et al., 2010 |
| rs35456279 | 3 | 78767714 | 4,84E-05 | - | *ROBO1* | intragenic | Aggressive behavior | Brevik et al., 2016 |
| rs149420276 | 3 | 78770461 | 3,44E-05 | - | *ROBO1* | intragenic | Aggressive behavior | Brevik et al., 2016 |
| rs12638524 | 3 | 87307837 | 1,75E-05 | - | *CHMP2B* | 51,3 | Aggressive behavior | Brevik et al., 2016 |
| rs6805837 | 3 | 100682218 | 2,03E-05 | - | *-* | - | Aggressive behavior | Brevik et al., 2016 |
| rs6797397 | 3 | 101653515 | 4.4E-05 | - | *LNP1* | intragenic | Antisocial behavior | Tielbeek et al., 2012 |
| rs9840370 | 3 | 101656215 | 4.7E-05 | - | *LNP1* | intragenic | Antisocial behavior | Tielbeek et al., 2012 |
| rs1132022 | 3 | 101657412 | 4.2E-05 | - | *LNP1* | intragenic | Antisocial behavior | Tielbeek et al., 2012 |
| rs4928050 | 3 | 101661149 | 3.3E-05 | - | *LNP1* | 3.3 | Antisocial behavior | Tielbeek et al., 2012 |
| rs17535407 | 3 | 105381763 | 1.0E-05 | - | *-* | - | Anger (reaction) | Mick et al., 2014 |
| rs6787984 | 3 | 107177546 | 1,32E-04 | *-* | *-* | - | Aggressive behavior | Pappa et al., 2016 |
| rs605795 | 3 | 111118349 | 4.5E-05 | - | *-* | - | Antisocial behavior | Tielbeek et al., 2012 |
| rs17722374 | 3 | 111221952 | 7.1E-06 | - | *-* | - | Antisocial behavior | Tielbeek et al., 2012 |
| rs1387024 | 3 | 114030291 | 4.1E-05 | - | *CD200R2* | intragenic | Anger (reaction) | Mick et al., 2014 |
| rs11917714 | 3 | 121016036 | 4.1E-05 | - | *NR1I2* | intragenic | Anger | Merjonen et al., 2011 |
| rs2276707 | 3 | 121016843 | 4.1E-05 | - | *NR1I2* | intragenic | Anger | Merjonen et al., 2011 |
| rs3814057 | 3 | 121019944 | 4.2E-05 | - | *NR1I2* | intragenic | Anger | Merjonen et al., 2011 |
| rs3814058 | 3 | 121019981 | 4.2E-05 | - | *NR1I2* | intragenic | Anger | Merjonen et al., 2011 |
| rs2873950 | 3 | 121029752 | 2.2E-05 | - | *GSK3B* | intragenic | Anger | Merjonen et al., 2011 |
| rs6808874 | 3 | 121040541 | 4.3E-05 | - | *GSK3B* | intragenic | Anger | Merjonen et al., 2011 |
| rs4624596 | 3 | 121054231 | 4.3E-05 | - | *GSK3B* | intragenic | Anger | Merjonen et al., 2011 |
| rs12634889 | 3 | 121079204 | 4.4E-05 | - | *GSK3B* | intragenic | Anger | Merjonen et al., 2011 |
| rs7639388 | 3 | 121092643 | 4.4E-05 | - | *GSK3B* | intragenic | Anger | Merjonen et al., 2011 |
| rs12629015 | 3 | 121100743 | 4.4E-05 | - | *GSK3B* | intragenic | Anger | Merjonen et al., 2011 |
| rs4340737 | 3 | 121104704 | 4.4E-05 | - | *GSK3B* | intragenic | Anger | Merjonen et al., 2011 |
| rs1574154 | 3 | 121109937 | 4.4E-05 | - | *GSK3B* | intragenic | Anger | Merjonen et al., 2011 |
| rs4688046 | 3 | 121146560 | 4.4E-05 | - | *GSK3B* | intragenic | Anger | Merjonen et al., 2011 |
| rs10934502 | 3 | 121151826 | 4.4E-05 | - | *GSK3B* | intragenic | Anger | Merjonen et al., 2011 |
| rs12634476 | 3 | 121161515 | 4.4E-05 | - | *GSK3B* | intragenic | Anger | Merjonen et al., 2011 |
| rs12638973 | 3 | 121167517 | 4.4E-05 | - | *GSK3B* | intragenic | Anger | Merjonen et al., 2011 |
| rs10934503 | 3 | 121173857 | 4.4E-05 | - | *GSK3B* | intragenic | Anger | Merjonen et al., 2011 |
| rs10433404 | 3 | 121175930 | 4.4E-05 | - | *GSK3B* | intragenic | Anger | Merjonen et al., 2011 |
| rs4688054 | 3 | 121216400 | 4.4E-05 | - | *GSK3B* | intragenic | Anger | Merjonen et al., 2011 |
| rs1870931 | 3 | 121239703 | 4.4E-05 | - | *GSK3B* | intragenic | Anger | Merjonen et al., 2011 |
| rs10934505 | 3 | 121250012 | 4.4E-05 | - | *GSK3B* | intragenic | Anger | Merjonen et al., 2011 |
| rs10934506 | 3 | 121253342 | 4.4E-05 | - | *GSK3B* | intragenic | Anger | Merjonen et al., 2011 |
| rs10934507 | 3 | 121253350 | 4.4E-05 | - | *GSK3B* | intragenic | Anger | Merjonen et al., 2011 |
| rs4688059 | 3 | 121294258 | 3.8E-05 | - | *GSK3B* | intragenic | Anger | Merjonen et al., 2011 |
| rs4646700 | 3 | 127369013 | 1,88E-04 | *-* | *ALDH1L1* | intragenic | Aggressive behavior | Pappa et al., 2016 |
| rs3893429 | 3 | 133361836 | 4,26E-06 | - | *-* | - | Aggressive behavior | Brevik et al., 2016 |
| rs4974486 | 3 | 135562218 | 2.0E-05 | - | *AMOTL2* | intragenic | Anger (temper) | Mick et al., 2014 |
| rs9847623 | 3 | 154851140 | 4,30E-04 | *-* | *-* | - | Aggressive behavior | Pappa et al., 2016 |
| rs4324501 | 3 | 159658683 | 3,71E-04 | *-* | *RSRC1* | intragenic | Aggressive behavior | Pappa et al., 2016 |
| rs12631628 | 3 | 159660117 | 3,49E-04 | *-* | *RSRC1* | intragenic | Aggressive behavior | Pappa et al., 2016 |
| rs12631675 | 3 | 159660276 | 3,39E-04 | *-* | *RSRC1* | intragenic | Aggressive behavior | Pappa et al., 2016 |
| rs2885663 | 3 | 159666957 | 2,80E-04 | *-* | *RSRC1* | intragenic | Aggressive behavior | Pappa et al., 2016 |
| rs6787172 | 3 | 159670505 | 2,26E-04 | *-* | *RSRC1* | intragenic | Aggressive behavior | Pappa et al., 2016 |
| rs7627374 | 3 | 159672758 | 1,80E-04 | *-* | *RSRC1* | intragenic | Aggressive behavior | Pappa et al., 2016 |
| rs1714507 | 3 | 159734126 | 4,01E-04 | *-* | *RSRC1* | intragenic | Aggressive behavior | Pappa et al., 2016 |
| rs1714509 | 3 | 159735652 | 7,52E-05 | *-* | *RSRC1* | intragenic | Aggressive behavior | Pappa et al., 2016 |
| rs1656366 | 3 | 159743493 | 4,42E-04 | *-* | *RSRC1* | intragenic | Aggressive behavior | Pappa et al., 2016 |
| rs1656364 | 3 | 159748915 | 6,77E-05 | *-* | *RSRC1* | 3,6 | Aggressive behavior | Pappa et al., 2016 |
| rs1630524 | 3 | 159752993 | 6,29E-05 | *-* | *RSRC1* | 7,7 | Aggressive behavior | Pappa et al., 2016 |
| rs1714524 | 3 | 159755790 | 4,29E-04 | *-* | *RSRC1* | 10,5 | Aggressive behavior | Pappa et al., 2016 |
| rs1656370 | 3 | 159758744 | 6,20E-05 | *-* | *MLF1* | 12,9 | Aggressive behavior | Pappa et al., 2016 |
| rs10276 | 3 | 159897860 | 4,72E-04 | *-* | *RARRES1* | intragenic | Aggressive behavior | Pappa et al., 2016 |
| rs12488447 | 3 | 159904518 | 4,76E-04 | *-* | *RARRES1* | intragenic | Aggressive behavior | Pappa et al., 2016 |
| rs9825140 | 3 | 159918068 | 3,61E-04 | *-* | *RARRES1* | intragenic | Aggressive behavior | Pappa et al., 2016 |
| rs4680461 | 3 | 159936854 | 3,39E-04 | *-* | *RARRES1* | 3,9 | Aggressive behavior | Pappa et al., 2016 |
| rs9858935 | 3 | 169004035 | 2,20E-04 | *-* | *SERPINI1* | intragenic | Aggressive behavior | Pappa et al., 2016 |
| rs13071510 | 3 | 169009349 | 1,24E-04 | *-* | *SERPINI1* | intragenic | Aggressive behavior | Pappa et al., 2016 |
| rs3792297 | 3 | 169013284 | 4,28E-04 | *-* | *SERPINI1* | intragenic | Aggressive behavior | Pappa et al., 2016 |
| rs1522278 | 3 | 170557772 | 2,77E-05 | *-* | *MDS1* | intragenic | Aggressive behavior | Pappa et al., 2016 |
| rs6774494 | 3 | 170565327 | 2,65E-05 | *-* | *MDS1* | intragenic | Aggressive behavior | Pappa et al., 2016 |
| rs16853609 | 3 | 170581369 | 4,01E-04 | *-* | *MDS1* | intragenic | Aggressive behavior | Pappa et al., 2016 |
| rs7622799 | 3 | 170581686 | 3,63E-04 | *-* | *MDS1* | intragenic | Aggressive behavior | Pappa et al., 2016 |
| rs16853620 | 3 | 170582811 | 1,29E-04 | *-* | *MDS1* | intragenic | Aggressive behavior | Pappa et al., 2016 |
| rs1835374 | 3 | 170585879 | 1,10E-04 | *-* | *MDS1* | intragenic | Aggressive behavior | Pappa et al., 2016 |
| rs1879795 | 3 | 170587236 | 1,04E-04 | *-* | *MDS1* | intragenic | Aggressive behavior | Pappa et al., 2016 |
| rs4955646 | 3 | 170600874 | 2,39E-04 | *-* | *MDS1* | intragenic | Aggressive behavior | Pappa et al., 2016 |
| rs16853663 | 3 | 170603970 | 2,86E-04 | *-* | *MDS1* | intragenic | Aggressive behavior | Pappa et al., 2016 |
| rs11923179 | 3 | 170631164 | 4,08E-04 | *-* | *MDS1* | intragenic | Aggressive behavior | Pappa et al., 2016 |
| rs10936582 | 3 | 170696594 | 4,89E-05 | *-* | *MDS1* | intragenic | Aggressive behavior | Pappa et al., 2016 |
| rs9856695 | 3 | 170696607 | 1,70E-04 | *-* | *MDS1* | intragenic | Aggressive behavior | Pappa et al., 2016 |
| rs12495069 | 3 | 170703798 | 1,16E-04 | *-* | *MDS1* | intragenic | Aggressive behavior | Pappa et al., 2016 |
| rs7631580 | 3 | 170709305 | 1,42E-04 | *-* | *MDS1* | intragenic | Aggressive behavior | Pappa et al., 2016 |
| rs3905410 | 3 | 170714407 | 1,85E-04 | *-* | *MDS1* | intragenic | Aggressive behavior | Pappa et al., 2016 |
| rs12630817 | 3 | 170715216 | 2,02E-04 | *-* | *MDS1* | intragenic | Aggressive behavior | Pappa et al., 2016 |
| rs7624529 | 3 | 170716095 | 1,95E-04 | *-* | *MDS1* | intragenic | Aggressive behavior | Pappa et al., 2016 |
| rs6444857 | 3 | 170721406 | 1,90E-04 | *-* | *MDS1* | intragenic | Aggressive behavior | Pappa et al., 2016 |
| rs1343036 | 3 | 170724940 | 2,28E-04 | *-* | *MDS1* | intragenic | Aggressive behavior | Pappa et al., 2016 |
| rs12492499 | 3 | 170731457 | 2,77E-04 | *-* | *MDS1* | intragenic | Aggressive behavior | Pappa et al., 2016 |
| rs3863104 | 3 | 170732170 | 2,78E-04 | *-* | *MDS1* | intragenic | Aggressive behavior | Pappa et al., 2016 |
| rs3909247 | 3 | 170732439 | 2,81E-04 | *-* | *MDS1* | intragenic | Aggressive behavior | Pappa et al., 2016 |
| rs1421007 | 3 | 170732484 | 4,14E-05 | *-* | *MDS1* | intragenic | Aggressive behavior | Pappa et al., 2016 |
| rs1833340 | 3 | 170732566 | 4,13E-05 | *-* | *MDS1* | intragenic | Aggressive behavior | Pappa et al., 2016 |
| rs1016444 | 3 | 170732778 | 1,13E-04 | *-* | *MDS1* | intragenic | Aggressive behavior | Pappa et al., 2016 |
| rs2901526 | 3 | 170734436 | 2,13E-04 | *-* | *MDS1* | intragenic | Aggressive behavior | Pappa et al., 2016 |
| rs733507 | 3 | 170735612 | 1,94E-04 | *-* | *MDS1* | intragenic | Aggressive behavior | Pappa et al., 2016 |
| rs733508 | 3 | 170735676 | 1,80E-04 | *-* | *MDS1* | intragenic | Aggressive behavior | Pappa et al., 2016 |
| rs6767836 | 3 | 170736687 | 1,87E-04 | *-* | *MDS1* | intragenic | Aggressive behavior | Pappa et al., 2016 |
| rs6776319 | 3 | 170736795 | 1,87E-04 | *-* | *MDS1* | intragenic | Aggressive behavior | Pappa et al., 2016 |
| rs12631412 | 3 | 170745692 | 3,22E-05 | *-* | *MDS1* | intragenic | Aggressive behavior | Pappa et al., 2016 |
| rs1421015 | 3 | 170761051 | 2,31E-05 | *-* | *MDS1* | intragenic | Aggressive behavior | Pappa et al., 2016 |
| rs6809237 | 3 | 170764656 | 2,19E-05 | *-* | *MDS1* | intragenic | Aggressive behavior | Pappa et al., 2016 |
| rs4955663 | 3 | 170773718 | 2,03E-05 | *-* | *MDS1* | intragenic | Aggressive behavior | Pappa et al., 2016 |
| rs6763160 | 3 | 170805443 | 2,61E-04 | *-* | *MDS1* | intragenic | Aggressive behavior | Pappa et al., 2016 |
| rs933604 | 3 | 170809139 | 1,27E-04 | *-* | *MDS1* | intragenic | Aggressive behavior | Pappa et al., 2016 |
| rs6804374 | 3 | 170825917 | 4,97E-05 | *-* | *MDS1* | intragenic | Aggressive behavior | Pappa et al., 2016 |
| rs1060462 | 3 | 170828312 | 1,19E-04 | *-* | *MDS1* | intragenic | Aggressive behavior | Pappa et al., 2016 |
| rs7625357 | 3 | 170830526 | 4,08E-06 | *-* | *MDS1* | intragenic | Aggressive behavior | Pappa et al., 2016 |
| rs1421005 | 3 | 170834471 | 5,81E-06 | *-* | *MDS1* | intragenic | Aggressive behavior | Pappa et al., 2016 |
| rs7652404 | 3 | 170835152 | 5,12E-06 | *-* | *MDS1* | intragenic | Aggressive behavior | Pappa et al., 2016 |
| rs10513679 | 3 | 170835878 | 4,46E-06 | *-* | *MDS1* | intragenic | Aggressive behavior | Pappa et al., 2016 |
| rs9818899 | 3 | 179611057 | 3,91E-04 | *-* | *-* | - | Aggressive behavior | Pappa et al., 2016 |
| rs6805982 | 3 | 179615058 | 3,87E-04 | *-* | *-* | - | Aggressive behavior | Pappa et al., 2016 |
| rs9840540 | 3 | 179615315 | 3,86E-04 | *-* | *-* | - | Aggressive behavior | Pappa et al., 2016 |
| rs9850747 | 3 | 179616954 | 3,85E-04 | *-* | *-* | - | Aggressive behavior | Pappa et al., 2016 |
| rs7648263 | 3 | 179617538 | 3,83E-04 | *-* | *-* | - | Aggressive behavior | Pappa et al., 2016 |
| rs9828420 | 3 | 179618159 | 3,82E-04 | *-* | *-* | - | Aggressive behavior | Pappa et al., 2016 |
| rs9290644 | 3 | 179622176 | 3,82E-04 | *-* | *-* | - | Aggressive behavior | Pappa et al., 2016 |
| rs9869753 | 3 | 179625593 | 3,63E-04 | *-* | *-* | - | Aggressive behavior | Pappa et al., 2016 |
| rs9863158 | 3 | 179629669 | 3,92E-04 | *-* | *-* | - | Aggressive behavior | Pappa et al., 2016 |
| rs9868767 | 3 | 179630781 | 3,71E-04 | *-* | *-* | - | Aggressive behavior | Pappa et al., 2016 |
| rs9868938 | 3 | 179630927 | 3,64E-04 | *-* | *-* | - | Aggressive behavior | Pappa et al., 2016 |
| rs9815296 | 3 | 179635045 | 4,32E-04 | *-* | *-* | - | Aggressive behavior | Pappa et al., 2016 |
| rs1968559 | 3 | 179635566 | 4,43E-04 | *-* | *-* | - | Aggressive behavior | Pappa et al., 2016 |
| rs11918971 | 3 | 181099454 | 4,62E-04 | *-* | *PEX5L* | intragenic | Aggressive behavior | Pappa et al., 2016 |
| rs12635173 | 3 | 181103472 | 2,93E-04 | *-* | *PEX5L* | intragenic | Aggressive behavior | Pappa et al., 2016 |
| rs1056103 | 3 | 181122086 | 5,75E-05 | *-* | *PEX5L* | intragenic | Aggressive behavior | Pappa et al., 2016 |
| rs10937013 | 3 | 181124545 | 6,50E-05 | *-* | *PEX5L* | intragenic | Aggressive behavior | Pappa et al., 2016 |
| rs1524517 | 3 | 181130059 | 4,93E-05 | *-* | *PEX5L* | intragenic | Aggressive behavior | Pappa et al., 2016 |
| rs7652323 | 3 | 181143334 | 5,89E-05 | *-* | *PEX5L* | intragenic | Aggressive behavior | Pappa et al., 2016 |
| rs7623941 | 3 | 181148712 | 4,16E-04 | *-* | *PEX5L* | intragenic | Aggressive behavior | Pappa et al., 2016 |
| rs1524515 | 3 | 181148926 | 4,31E-04 | *-* | *PEX5L* | intragenic | Aggressive behavior | Pappa et al., 2016 |
| rs1524516 | 3 | 181148992 | 4,07E-04 | *-* | *PEX5L* | intragenic | Aggressive behavior | Pappa et al., 2016 |
| rs6443681 | 3 | 181149751 | 4,00E-04 | *-* | *PEX5L* | intragenic | Aggressive behavior | Pappa et al., 2016 |
| rs6443682 | 3 | 181149904 | 4,14E-04 | *-* | *PEX5L* | intragenic | Aggressive behavior | Pappa et al., 2016 |
| rs7613963 | 3 | 181151022 | 3,78E-04 | *-* | *PEX5L* | intragenic | Aggressive behavior | Pappa et al., 2016 |
| rs2031043 | 3 | 181171298 | 3,03E-04 | *-* | *PEX5L* | intragenic | Aggressive behavior | Pappa et al., 2016 |
| rs2031044 | 3 | 181171365 | 3,62E-04 | *-* | *PEX5L* | intragenic | Aggressive behavior | Pappa et al., 2016 |
| rs3774256 | 3 | 181171958 | 4,19E-04 | *-* | *PEX5L* | intragenic | Aggressive behavior | Pappa et al., 2016 |
| rs9837660 | 3 | 181176506 | 7,35E-05 | *-* | *PEX5L* | intragenic | Aggressive behavior | Pappa et al., 2016 |
| rs4855131 | 3 | 181179413 | 1,17E-04 | *-* | *PEX5L* | intragenic | Aggressive behavior | Pappa et al., 2016 |
| rs956396 | 3 | 181180766 | 6,29E-05 | *-* | *PEX5L* | intragenic | Aggressive behavior | Pappa et al., 2016 |
| rs956395 | 3 | 181180935 | 6,23E-05 | *-* | *PEX5L* | intragenic | Aggressive behavior | Pappa et al., 2016 |
| rs9290781 | 3 | 185312427 | 5.3E-06 | - | *HTR3E* | 5.0 | Anger | Merjonen et al., 2011 |
| rs11718245 | 3 | 185313087 | 9.6E-06 | - | *HTR3E* | 5.6 | Anger | Merjonen et al., 2011 |
| rs7427596 | 3 | 185316433 | 9.7E-06 | - | *HTR3E* | 9.0 | Anger | Merjonen et al., 2011 |
| rs9815292 | 3 | 185317184 | 1.5E-05 | - | *HTR3E* | 9.7 | Anger | Merjonen et al., 2011 |
| rs9868117 | 3 | 185318264 | 5.2E-06 | - | *HTR3E* | 10.8 | Anger | Merjonen et al., 2011 |
| rs9817497 | 3 | 185319125 | 9.5E-06 | - | *HTR3E* | 11.6 | Anger | Merjonen et al., 2011 |
| rs11924174 | 3 | 185323323 | 9.3E-06 | - | *EIF2B5* | 12.2 | Anger | Merjonen et al., 2011 |
| rs11916456 | 3 | 185323360 | 9.3E-06 | - | *EIF2B5* | 12.1 | Anger | Merjonen et al., 2011 |
| rs11708998 | 3 | 185324408 | 9.0E-06 | - | *EIF2B5* | 11.1 | Anger | Merjonen et al., 2011 |
| rs11924144 | 3 | 187328277 | 4,96E-04 | *-* | *ETV5* | 18,7 | Aggressive behavior | Pappa et al., 2016 |
| rs9830802 | 3 | 187378139 | 3,45E-04 | *-* | *DGKG* | intragenic | Aggressive behavior | Pappa et al., 2016 |
| rs759679 | 3 | 187378812 | 2,43E-04 | *-* | *DGKG* | intragenic | Aggressive behavior | Pappa et al., 2016 |
| rs4686742 | 3 | 187379481 | 1,49E-04 | *-* | *DGKG* | intragenic | Aggressive behavior | Pappa et al., 2016 |
| rs9851948 | 3 | 187381934 | 1,82E-04 | *-* | *DGKG* | intragenic | Aggressive behavior | Pappa et al., 2016 |
| rs293844 | 3 | 192614640 | 9.9E-04 | 3.3E-03 | *CCDC50* | 22.0 | AB+/CU+ | Viding et al., 2010 |
| rs3846069 | 3 | 198248468 | 3,89E-05 | - | *DLG1* | 5,4 | Aggressive behavior | Brevik et al., 2016 |
| rs4527428 | 4 | 5704931 | 3,28E-05 | *-* | *EVC2* | intragenic | Aggressive behavior | Pappa et al., 2016 |
| rs6842115 | 4 | 5709455 | 2,19E-05 | *-* | *EVC2* | intragenic | Aggressive behavior | Pappa et al., 2016 |
| rs41516949 | 4 | 6975910 | 3.8E-03 | 3.1E-03 | *TBC1D14* | intragenic | AB+/CU+ | Viding et al., 2010 |
| rs6446569 | 4 | 7177419 | 1.1E-03 | 3.3E-03 | *GRPEL1* | 56.7 | AB+/CU+ | Viding et al., 2010 |
| rs4696814 | 4 | 8378011 | 1,36E-04 | *-* | *HTRA3* | 18,3 | Aggressive behavior | Pappa et al., 2016 |
| rs1030000 | 4 | 13481192 | 4,11E-04 | *-* | *-* | - | Aggressive behavior | Pappa et al., 2016 |
| rs6837229 | 4 | 16289063 | 4.9E-05 | - | *LDB2* | intragenic | Antisocial behavior | Tielbeek et al., 2012 |
| rs77868629 | 4 | 22467027 | 3,55E-05 | - | *GBA3* | 36,8 | Aggressive behavior | Brevik et al., 2016 |
| rs6831024 | 4 | 25472473 | 7.3E-06 | - | *KIAA0746* | intragenic | Anger (reaction) | Mick et al., 2014 |
| rs12163915 | 4 | 30320328 | 3,16E-04 | *-* | *PCDH7* | 10,8 | Aggressive behavior | Pappa et al., 2016 |
| rs9291546 | 4 | 30327431 | 3,57E-04 | *-* | *PCDH7* | 3,7 | Aggressive behavior | Pappa et al., 2016 |
| rs4288052 | 4 | 32418518 | 3.6E-05 | - | *-* | - | Anger | Merjonen et al., 2011 |
| rs4583806 | 4 | 32421365 | 3.6E-05 | - | *-* | - | Anger | Merjonen et al., 2011 |
| rs6832273 | 4 | 32431499 | 3.6E-05 | - | *-* | - | Anger | Merjonen et al., 2011 |
| rs10007711 | 4 | 32457121 | 3.5E-05 | - | *-* | - | Anger | Merjonen et al., 2011 |
| rs10006761 | 4 | 32459036 | 3.5E-05 | - | *-* | - | Anger | Merjonen et al., 2011 |
| rs10005246 | 4 | 32463396 | 3.5E-05 | - | *-* | - | Anger | Merjonen et al., 2011 |
| rs17340387 | 4 | 32757647 | 1.2E-05 | - | *-* | - | Anger | Merjonen et al., 2011 |
| rs4859315 | 4 | 32867764 | 4.6E-06 | - | *-* | - | Anger | Merjonen et al., 2011 |
| rs1319729 | 4 | 36562852 | 7,44E-05 | *-* | *-* | - | Aggressive behavior | Pappa et al., 2016 |
| rs2376777 | 4 | 37065032 | 3,46E-04 | *-* | *C4orf19* | 66,9 | Aggressive behavior | Pappa et al., 2016 |
| rs10856830 | 4 | 37074655 | 3,33E-04 | *-* | *C4orf19* | 57,3 | Aggressive behavior | Pappa et al., 2016 |
| rs10028027 | 4 | 37076701 | 3,43E-04 | *-* | *C4orf19* | 55,2 | Aggressive behavior | Pappa et al., 2016 |
| rs6816721 | 4 | 37077170 | 3,96E-04 | *-* | *C4orf19* | 54,8 | Aggressive behavior | Pappa et al., 2016 |
| rs17595063 | 4 | 45185804 | 2.0E-05 | - | *-* | - | Antisocial behavior | Tielbeek et al., 2012 |
| rs1442097 | 4 | 46845754 | 3.2E-05 | - | *GABRB1* | intragenic | Anger | Merjonen et al., 2011 |
| rs13125961 | 4 | 46848066 | 3.0E-05 | - | *GABRB1* | intragenic | Anger | Merjonen et al., 2011 |
| rs11727585 | 4 | 46850113 | 2.9E-05 | - | *GABRB1* | intragenic | Anger | Merjonen et al., 2011 |
| rs2165611 | 4 | 46859245 | 4.9E-05 | - | *GABRB1* | intragenic | Anger | Merjonen et al., 2011 |
| rs10015561 | 4 | 46863465 | 1.4E-05 | - | *GABRB1* | intragenic | Anger | Merjonen et al., 2011 |
| rs114439515 | 4 | 57371160 | 8,50E-06 | - | *SPINK2* | intragenic | Aggressive behavior | Brevik et al., 2016 |
| rs147046559 | 4 | 57899375 | 2,82E-05 | - | *-* | - | Aggressive behavior | Brevik et al., 2016 |
| rs78030545 | 4 | 58066680 | 1,91E-06 | - | *-* | - | Aggressive behavior | Brevik et al., 2016 |
| rs113904903 | 4 | 58179948 | 4,29E-05 | - | *-* | - | Aggressive behavior | Brevik et al., 2016 |
| rs10024718 | 4 | 65105638 | 3.1E-05 | - | *-* | - | Antisocial behavior | Tielbeek et al., 2012 |
| rs2218192 | 4 | 67806116 | 4,55E-04 | *-* | *-* | - | Aggressive behavior | Pappa et al., 2016 |
| rs17818523 | 4 | 67813208 | 2,95E-04 | *-* | *-* | - | Aggressive behavior | Pappa et al., 2016 |
| rs17767421 | 4 | 67814893 | 2,73E-04 | *-* | *-* | - | Aggressive behavior | Pappa et al., 2016 |
| rs1595908 | 4 | 67836697 | 2,50E-04 | *-* | *-* | - | Aggressive behavior | Pappa et al., 2016 |
| rs17818614 | 4 | 67837959 | 2,42E-04 | *-* | *-* | - | Aggressive behavior | Pappa et al., 2016 |
| rs17818679 | 4 | 67839653 | 2,72E-04 | *-* | *-* | - | Aggressive behavior | Pappa et al., 2016 |
| rs1348059 | 4 | 67875579 | 1,88E-04 | *-* | *-* | - | Aggressive behavior | Pappa et al., 2016 |
| rs1348058 | 4 | 67875720 | 1,89E-04 | *-* | *-* | - | Aggressive behavior | Pappa et al., 2016 |
| rs10002035 | 4 | 67879431 | 3,47E-05 | *-* | *-* | - | Aggressive behavior | Pappa et al., 2016 |
| rs11131694 | 4 | 67896689 | 2,62E-04 | *-* | *-* | - | Aggressive behavior | Pappa et al., 2016 |
| rs1497431 | 4 | 67897598 | 4,02E-04 | *-* | *-* | - | Aggressive behavior | Pappa et al., 2016 |
| rs17624734 | 4 | 68059721 | 2,90E-04 | *-* | *CENPC1* | intragenic | Aggressive behavior | Pappa et al., 2016 |
| rs6552104 | 4 | 68068415 | 4,25E-04 | *-* | *CENPC1* | intragenic | Aggressive behavior | Pappa et al., 2016 |
| rs17625547 | 4 | 68092604 | 4,49E-04 | *-* | *CENPC1* | intragenic | Aggressive behavior | Pappa et al., 2016 |
| rs1534841 | 4 | 70344332 | 4,73E-04 | *-* | *UGT2B4* | 36,1 | Aggressive behavior | Pappa et al., 2016 |
| rs6600933 | 4 | 70345280 | 4,51E-04 | *-* | *UGT2B4* | 35,2 | Aggressive behavior | Pappa et al., 2016 |
| rs1032932 | 4 | 76503789 | 1,10E-04 | *-* | *-* | - | Aggressive behavior | Pappa et al., 2016 |
| rs3853186 | 4 | 76509785 | 1,80E-04 | *-* | *-* | - | Aggressive behavior | Pappa et al., 2016 |
| rs976368 | 4 | 76511430 | 1,48E-04 | *-* | *-* | - | Aggressive behavior | Pappa et al., 2016 |
| rs11936139 | 4 | 76513590 | 1,89E-04 | *-* | *-* | - | Aggressive behavior | Pappa et al., 2016 |
| rs2201331 | 4 | 76515445 | 2,11E-04 | *-* | *-* | - | Aggressive behavior | Pappa et al., 2016 |
| rs4241597 | 4 | 78121149 | 1.3E-03 | 3.3E-03 | *sep-11* | intragenic | AB+/CU+ | Viding et al., 2010 |
| rs6828528 | 4 | 81214905 | 3,57E-04 | *-* | *ANTXR2* | 1,7 | Aggressive behavior | Pappa et al., 2016 |
| rs2867698 | 4 | 81220964 | 4,27E-04 | *-* | *ANTXR2* | 7,7 | Aggressive behavior | Pappa et al., 2016 |
| rs150333105 | 4 | 86625858 | 1,87E-05 | - | *ARHGAP24* | intragenic | Aggressive behavior | Brevik et al., 2016 |
| rs10050093 | 4 | 94942802 | 6.6E-03 | 4.4E-03 | *ATOH1* | 26.3 | AB+/CU+ | Viding et al., 2010 |
| rs2045797 | 4 | 103572097 | 1.0E-05 | - | *NFKB1* | 69.4 | Anger (reaction) | Mick et al., 2014 |
| rs6533015 | 4 | 103574556 | 1.9E-05 | - | *NFKB1* | 67.0 | Anger (reaction) | Mick et al., 2014 |
| rs17032641 | 4 | 103589914 | 2.0E-05 | - | *NFKB1* | 51.6 | Antisocial behavior | Tielbeek et al., 2012 |
| rs1313925 | 4 | 103596601 | 2.6E-05 | - | *NFKB1* | 44.9 | Anger (reaction) | Mick et al., 2014 |
| rs17008528 | 4 | 103602131 | 1.6E-05 | - | *NFKB1* | 39.4 | Antisocial behavior | Tielbeek et al., 2012 |
| rs13117745 | 4 | 103697738 | 2,86E-04 | *-* | *NFKB1* | intragenic | Aggressive behavior | Pappa et al., 2016 |
| rs4383690 | 4 | 107586296 | 1.8E-03 | 1.6E-03 | *SCYE1* | 97.3 | AB+/CU+ | Viding et al., 2010 |
| rs78451261 | 4 | 115444634 | 4,99E-05 | - | *-* | - | Aggressive behavior | Brevik et al., 2016 |
| rs12647756 | 4 | 119550717 | 1.2E-03 | 4.5E-03 | *PRSS12* | 57.4 | AB+/CU+ | Viding et al., 2010 |
| rs142453940 | 4 | 134696528 | 1,30E-05 | - | *-* | - | Aggressive behavior | Brevik et al., 2016 |
| rs6837219 | 4 | 139520188 | 2,00E-04 | *-* | *-* | - | Aggressive behavior | Pappa et al., 2016 |
| rs11935389 | 4 | 139523728 | 2,05E-04 | *-* | *-* | - | Aggressive behavior | Pappa et al., 2016 |
| rs2052750 | 4 | 139524740 | 1,90E-04 | *-* | *-* | - | Aggressive behavior | Pappa et al., 2016 |
| rs1872296 | 4 | 143451107 | 3,97E-04 | *-* | *INPP4B* | intragenic | Aggressive behavior | Pappa et al., 2016 |
| rs10013303 | 4 | 143453992 | 3,43E-04 | *-* | *INPP4B* | intragenic | Aggressive behavior | Pappa et al., 2016 |
| rs10034842 | 4 | 143454059 | 3,36E-04 | *-* | *INPP4B* | intragenic | Aggressive behavior | Pappa et al., 2016 |
| rs10024947 | 4 | 143454113 | 3,39E-04 | *-* | *INPP4B* | intragenic | Aggressive behavior | Pappa et al., 2016 |
| rs16998530 | 4 | 143454708 | 3,29E-04 | *-* | *INPP4B* | intragenic | Aggressive behavior | Pappa et al., 2016 |
| rs3756121 | 4 | 143454794 | 2,85E-04 | *-* | *INPP4B* | intragenic | Aggressive behavior | Pappa et al., 2016 |
| rs11945996 | 4 | 143455171 | 3,29E-04 | *-* | *INPP4B* | intragenic | Aggressive behavior | Pappa et al., 2016 |
| rs6811427 | 4 | 143456200 | 2,93E-04 | *-* | *INPP4B* | intragenic | Aggressive behavior | Pappa et al., 2016 |
| rs6858661 | 4 | 143456347 | 2,87E-04 | *-* | *INPP4B* | intragenic | Aggressive behavior | Pappa et al., 2016 |
| rs6812034 | 4 | 143456493 | 2,84E-04 | *-* | *INPP4B* | intragenic | Aggressive behavior | Pappa et al., 2016 |
| rs6850050 | 4 | 143458214 | 2,76E-04 | *-* | *INPP4B* | intragenic | Aggressive behavior | Pappa et al., 2016 |
| rs17015920 | 4 | 143460101 | 3,17E-04 | *-* | *INPP4B* | intragenic | Aggressive behavior | Pappa et al., 2016 |
| rs10519642 | 4 | 143462600 | 2,56E-04 | *-* | *INPP4B* | intragenic | Aggressive behavior | Pappa et al., 2016 |
| rs13117885 | 4 | 146706090 | 3,34E-04 | *-* | *SMAD1* | 6,3 | Aggressive behavior | Pappa et al., 2016 |
| rs11944100 | 4 | 146707667 | 3,11E-04 | *-* | *SMAD1* | 7,9 | Aggressive behavior | Pappa et al., 2016 |
| rs13140532 | 4 | 146708694 | 3,29E-04 | *-* | *SMAD1* | 8,9 | Aggressive behavior | Pappa et al., 2016 |
| rs13123559 | 4 | 146710052 | 3,27E-04 | *-* | *SMAD1* | 10,3 | Aggressive behavior | Pappa et al., 2016 |
| rs12649100 | 4 | 146710977 | 3,23E-04 | *-* | *SMAD1* | 11,2 | Aggressive behavior | Pappa et al., 2016 |
| rs148603251 | 4 | 149731592 | 1,46E-05 | - | *-* | - | Aggressive behavior | Brevik et al., 2016 |
| rs114719252 | 4 | 149782416 | 2,83E-05 | - | *-* | - | Aggressive behavior | Brevik et al., 2016 |
| rs77077623 | 4 | 149825195 | 4,14E-05 | - | *-* | - | Aggressive behavior | Brevik et al., 2016 |
| rs4479686 | 4 | 152618774 | 2.6E-04 | 4.2E-03 | *FAM160A1* | intragenic | AB+/CU+ | Viding et al., 2010 |
| rs6846114 | 4 | 152824008 | 5.5E-03 | 2.0E-03 | *PET112L* | intragenic | AB+/CU+ | Viding et al., 2010 |
| rs1490666 | 4 | 155622735 | 2.6E-03 | 3.1E-03 | *PLRG1* | 54.4 | AB+/CU+ | Viding et al., 2010 |
| rs17038216 | 4 | 160575274 | 1.9E-05 | - | *RAPGEF2* | 74.5 | Antisocial behavior | Tielbeek et al., 2012 |
| rs13112097 | 4 | 161551619 | 4.4E-05 | - | *-* | - | Antisocial behavior | Tielbeek et al., 2012 |
| rs6536590 | 4 | 162539362 | 1,08E-04 | *-* | *FSTL5* | intragenic | Aggressive behavior | Pappa et al., 2016 |
| rs11725954 | 4 | 162540353 | 1,31E-04 | *-* | *FSTL5* | intragenic | Aggressive behavior | Pappa et al., 2016 |
| rs9998941 | 4 | 162544312 | 7,03E-05 | *-* | *FSTL5* | intragenic | Aggressive behavior | Pappa et al., 2016 |
| rs4547755 | 4 | 162546715 | 3,97E-05 | *-* | *FSTL5* | intragenic | Aggressive behavior | Pappa et al., 2016 |
| rs17397279 | 4 | 162546792 | 1,02E-04 | *-* | *FSTL5* | intragenic | Aggressive behavior | Pappa et al., 2016 |
| rs2706836 | 4 | 168570603 | 4,69E-04 | *-* | *-* | - | Aggressive behavior | Pappa et al., 2016 |
| rs7671943 | 4 | 168631119 | 2,21E-04 | *-* | *-* | - | Aggressive behavior | Pappa et al., 2016 |
| rs7697737 | 4 | 175827908 | 4.3E-05 | - | *GLRA3* | intragenic | CBCL-DP | Mick et al., 2011 |
| rs6834498 | 4 | 189802164 | 1.6E-05 | - | *-* |  | Anger (temper) | Mick et al., 2014 |
| rs6831779 | 4 | 189814140 | 2.5E-05 | - | *-* |  | Anger (temper) | Mick et al., 2014 |
| rs11737490 | 4 | 189819962 | 3.7E-05 | - | *-* |  | Anger (temper) | Mick et al., 2014 |
| rs12653269 | 5 | 5075661 | 2,75E-04 | *-* | *-* | - | Aggressive behavior | Pappa et al., 2016 |
| rs116335706 | 5 | 6541175 | 2,74E-05 | - | *-* | - | Aggressive behavior | Brevik et al., 2016 |
| rs77068530 | 5 | 6546680 | 2,95E-05 | - | *-* | - | Aggressive behavior | Brevik et al., 2016 |
| rs80115503 | 5 | 6559411 | 1,96E-05 | - | *NSUN2* | 92,9 | Aggressive behavior | Brevik et al., 2016 |
| rs74695265 | 5 | 6559887 | 3,16E-05 | - | *NSUN2* | 92,5 | Aggressive behavior | Brevik et al., 2016 |
| rs77951737 | 5 | 6559915 | 3,21E-05 | - | *NSUN2* | 92,4 | Aggressive behavior | Brevik et al., 2016 |
| rs116109275 | 5 | 6570955 | 4,95E-05 | - | *NSUN2* | 81,4 | Aggressive behavior | Brevik et al., 2016 |
| rs992283 | 5 | 6592186 | 2,13E-05 | - | *NSUN2* | 60,2 | Aggressive behavior | Brevik et al., 2016 |
| rs11742745 | 5 | 7095946 | 1,77E-05 | - | *-* | - | Aggressive behavior | Brevik et al., 2016 |
| rs11745335 | 5 | 7098021 | 3,33E-05 | - | *-* | - | Aggressive behavior | Brevik et al., 2016 |
| rs11741274 | 5 | 7098051 | 3,43E-05 | - | *-* | - | Aggressive behavior | Brevik et al., 2016 |
| rs11134201 | 5 | 7098934 | 3,46E-05 | - | *-* | - | Aggressive behavior | Brevik et al., 2016 |
| rs12516838 | 5 | 7099179 | 3,47E-05 | - | *-* | - | Aggressive behavior | Brevik et al., 2016 |
| rs72719015 | 5 | 7099779 | 3,57E-05 | - | *-* | - | Aggressive behavior | Brevik et al., 2016 |
| rs79632925 | 5 | 7099956 | 3,50E-05 | - | *-* | - | Aggressive behavior | Brevik et al., 2016 |
| rs11743025 | 5 | 7100375 | 3,51E-05 | - | *-* | - | Aggressive behavior | Brevik et al., 2016 |
| rs17273158 | 5 | 9748859 | 2,08E-04 | *-* | *TAS2R1* | 65,4 | Aggressive behavior | Pappa et al., 2016 |
| rs11744711 | 5 | 9752040 | 4,60E-04 | *-* | *TAS2R1* | 68,6 | Aggressive behavior | Pappa et al., 2016 |
| rs2915512 | 5 | 10546283 | 2,49E-04 | *-* | *ROPN1L* | 28,1 | Aggressive behavior | Pappa et al., 2016 |
| rs7716417 | 5 | 29503421 | 9,47E-05 | *-* | *-* | - | Aggressive behavior | Pappa et al., 2016 |
| rs6869294 | 5 | 31218170 | 1.1E-05 | - | *CDH6* | 11.4 | CD | Anney et al., 2008 |
| rs16901319 | 5 | 31645928 | 4,98E-04 | *-* | *C5orf22* | 55,0 | Aggressive behavior | Pappa et al., 2016 |
| rs11959325 | 5 | 37961275 | 3,96E-05 | - | *GDNF* | 85,8 | Aggressive behavior | Brevik et al., 2016 |
| rs67437118 | 5 | 37978090 | 4,14E-05 | - | *-* | - | Aggressive behavior | Brevik et al., 2016 |
| rs386994 | 5 | 38854038 | 1,81E-05 | - | *OSMR* | 27,9 | Aggressive behavior | Brevik et al., 2016 |
| rs375364 | 5 | 38857425 | 2,61E-05 | - | *OSMR* | 24,5 | Aggressive behavior | Brevik et al., 2016 |
| rs413278 | 5 | 38857797 | 2,61E-05 | - | *OSMR* | 24,1 | Aggressive behavior | Brevik et al., 2016 |
| rs369886 | 5 | 38859011 | 2,57E-05 | - | *OSMR* | 22,9 | Aggressive behavior | Brevik et al., 2016 |
| rs428525 | 5 | 38864383 | 4,40E-05 | - | *OSMR* | 17,5 | Aggressive behavior | Brevik et al., 2016 |
| rs655058 | 5 | 38864974 | 4,41E-05 | - | *OSMR* | 16,9 | Aggressive behavior | Brevik et al., 2016 |
| rs2921715 | 5 | 38867810 | 1,12E-05 | - | *OSMR* | 14,1 | Aggressive behavior | Brevik et al., 2016 |
| rs1239353 | 5 | 38871110 | 2,59E-05 | - | *OSMR* | 10,8 | Aggressive behavior | Brevik et al., 2016 |
| rs357285 | 5 | 38873836 | 2,03E-05 | - | *OSMR* | 8,1 | Aggressive behavior | Brevik et al., 2016 |
| rs540558 | 5 | 38880030 | 2,96E-05 | - | *OSMR* | 1,9 | Aggressive behavior | Brevik et al., 2016 |
| rs181544 | 5 | 38884652 | 3,81E-05 | - | *OSMR* | intragenic | Aggressive behavior | Brevik et al., 2016 |
| rs357288 | 5 | 38887032 | 3,64E-05 | - | *OSMR* | intragenic | Aggressive behavior | Brevik et al., 2016 |
| rs357289 | 5 | 38887682 | 3,71E-05 | - | *OSMR* | intragenic | Aggressive behavior | Brevik et al., 2016 |
| rs548287 | 5 | 38891102 | 4,76E-05 | - | *OSMR* | intragenic | Aggressive behavior | Brevik et al., 2016 |
| rs533654 | 5 | 38913723 | 4,89E-04 | *-* | *OSMR* | intragenic | Aggressive behavior | Pappa et al., 2016 |
| rs13186506 | 5 | 59147672 | 8,96E-05 | *-* | *PDE4D* | intragenic | Aggressive behavior | Pappa et al., 2016 |
| rs10514880 | 5 | 59148611 | 7,13E-05 | *-* | *PDE4D* | intragenic | Aggressive behavior | Pappa et al., 2016 |
| rs6859121 | 5 | 59157514 | 9,07E-05 | *-* | *PDE4D* | intragenic | Aggressive behavior | Pappa et al., 2016 |
| rs16890269 | 5 | 59173545 | 2,02E-04 | *-* | *PDE4D* | intragenic | Aggressive behavior | Pappa et al., 2016 |
| rs16890276 | 5 | 59173918 | 3,14E-04 | *-* | *PDE4D* | intragenic | Aggressive behavior | Pappa et al., 2016 |
| rs10514888 | 5 | 59188266 | 3,34E-04 | *-* | *PDE4D* | intragenic | Aggressive behavior | Pappa et al., 2016 |
| rs16890316 | 5 | 59195189 | 3,45E-04 | *-* | *PDE4D* | intragenic | Aggressive behavior | Pappa et al., 2016 |
| rs1003219 | 5 | 59200750 | 1,68E-04 | *-* | *PDE4D* | intragenic | Aggressive behavior | Pappa et al., 2016 |
| rs1003218 | 5 | 59200831 | 4,74E-04 | *-* | *PDE4D* | intragenic | Aggressive behavior | Pappa et al., 2016 |
| rs349499 | 5 | 62589415 | 1.9E-05 | - | *-* | - | Antisocial behavior | Tielbeek et al., 2012 |
| rs349497 | 5 | 62589803 | 3.0E-05 | - | *-* | - | Antisocial behavior | Tielbeek et al., 2012 |
| rs349496 | 5 | 62590942 | 1.9E-05 | - | *-* | - | Antisocial behavior | Tielbeek et al., 2012 |
| rs349495 | 5 | 62591273 | 2.5E-05 | - | *-* | - | Antisocial behavior | Tielbeek et al., 2012 |
| rs349494 | 5 | 62591646 | 1.9E-05 | - | *-* | - | Antisocial behavior | Tielbeek et al., 2012 |
| rs349493 | 5 | 62592250 | 1.4E-05 | - | *-* | - | Antisocial behavior | Tielbeek et al., 2012 |
| rs349492 | 5 | 62592269 | 1.9E-05 | - | *-* | - | Antisocial behavior | Tielbeek et al., 2012 |
| rs349491 | 5 | 62592729 | 1.8E-05 | - | *-* | - | Antisocial behavior | Tielbeek et al., 2012 |
| rs349488 | 5 | 62593454 | 1.8E-05 | - | *-* | - | Antisocial behavior | Tielbeek et al., 2012 |
| rs349487 | 5 | 62593521 | 1.8E-05 | - | *-* | - | Antisocial behavior | Tielbeek et al., 2012 |
| rs401859 | 5 | 62594127 | 1.1E-05 | - | *-* | - | Antisocial behavior | Tielbeek et al., 2012 |
| rs373666 | 5 | 62594238 | 1.1E-05 | - | *-* | - | Antisocial behavior | Tielbeek et al., 2012 |
| rs423216 | 5 | 62594263 | 1.8E-05 | - | *-* | - | Antisocial behavior | Tielbeek et al., 2012 |
| rs462861 | 5 | 62594850 | 1.0E-05 | - | *-* | - | Antisocial behavior | Tielbeek et al., 2012 |
| rs460325 | 5 | 62595164 | 1.0E-05 | - | *-* | - | Antisocial behavior | Tielbeek et al., 2012 |
| rs460949 | 5 | 62595222 | 1.0E-05 | - | *-* | - | Antisocial behavior | Tielbeek et al., 2012 |
| rs455575 | 5 | 62595583 | 1.5E-05 | - | *-* | - | Antisocial behavior | Tielbeek et al., 2012 |
| rs458362 | 5 | 62596288 | 9.7E-06 | - | *-* | - | Antisocial behavior | Tielbeek et al., 2012 |
| rs1676146 | 5 | 62596743 | 9.7E-06 | - | *-* | - | Antisocial behavior | Tielbeek et al., 2012 |
| rs434640 | 5 | 62597582 | 1.5E-05 | - | *-* | - | Antisocial behavior | Tielbeek et al., 2012 |
| rs443543 | 5 | 62600416 | 9.6E-06 | - | *-* | - | Antisocial behavior | Tielbeek et al., 2012 |
| rs418226 | 5 | 62602292 | 1.5E-05 | - | *-* | - | Antisocial behavior | Tielbeek et al., 2012 |
| rs392246 | 5 | 62602498 | 1.5E-05 | - | *-* | - | Antisocial behavior | Tielbeek et al., 2012 |
| rs387996 | 5 | 62602687 | 1.5E-05 | - | *-* | - | Antisocial behavior | Tielbeek et al., 2012 |
| rs427421 | 5 | 62602801 | 1.5E-05 | - | *-* | - | Antisocial behavior | Tielbeek et al., 2012 |
| rs444037 | 5 | 62606171 | 1.2E-05 | - | *-* | - | Antisocial behavior | Tielbeek et al., 2012 |
| rs369747 | 5 | 62606393 | 1.5E-05 | - | *-* | - | Antisocial behavior | Tielbeek et al., 2012 |
| rs424672 | 5 | 62612720 | 1.4E-05 | - | *-* | - | Antisocial behavior | Tielbeek et al., 2012 |
| rs409363 | 5 | 62612882 | 1.4E-05 | - | *-* | - | Antisocial behavior | Tielbeek et al., 2012 |
| rs386661 | 5 | 62618589 | 1.7E-05 | - | *-* | - | Antisocial behavior | Tielbeek et al., 2012 |
| rs450583 | 5 | 62618893 | 2.1E-05 | - | *-* | - | Antisocial behavior | Tielbeek et al., 2012 |
| rs346431 | 5 | 62620271 | 1.7E-05 | - | *-* | - | Antisocial behavior | Tielbeek et al., 2012 |
| rs346425 | 5 | 62632000 | 2.5E-07 | - | *-* | - | Antisocial behavior | Tielbeek et al., 2012 |
| rs3756668 | 5 | 67631844 | 3,28E-04 | *-* | *PIK3R1* | intragenic | Aggressive behavior | Pappa et al., 2016 |
| rs17166656 | 5 | 98456063 | 2,18E-05 | - | *-* | - | ODD | Aebi et al., 2016 |
| rs17166666 | 5 | 98463458 | 3,47E-05 | - | *-* | - | ODD | Aebi et al., 2016 |
| rs7720227 | 5 | 98468720 | 3,60E-05 | - | *-* | - | ODD | Aebi et al., 2016 |
| rs7715722 | 5 | 98468884 | 3,60E-05 | - | *-* | - | ODD | Aebi et al., 2016 |
| rs10065851 | 5 | 99366095 | 4.4E-05 | - | *-* | - | Anger | Merjonen et al., 2011 |
| rs74429604 | 5 | 101035609 | 4,51E-05 | - | *-* | - | Aggressive behavior | Brevik et al., 2016 |
| rs1438251 | 5 | 106194322 | 2,79E-05 | - | *-* | - | Aggressive behavior | Brevik et al., 2016 |
| rs11241004 | 5 | 108672117 | 3,02E-04 | *-* | *PJA2* | 26,2 | Aggressive behavior | Pappa et al., 2016 |
| rs3890423 | 5 | 108675850 | 3,88E-04 | *-* | *PJA2* | 22,5 | Aggressive behavior | Pappa et al., 2016 |
| rs1985666 | 5 | 108676130 | 4,37E-04 | *-* | *PJA2* | 22,2 | Aggressive behavior | Pappa et al., 2016 |
| rs10055107 | 5 | 108679640 | 4,17E-04 | *-* | *PJA2* | 18,7 | Aggressive behavior | Pappa et al., 2016 |
| rs868260 | 5 | 108682628 | 2,45E-04 | *-* | *PJA2* | 15,7 | Aggressive behavior | Pappa et al., 2016 |
| rs1428929 | 5 | 108713773 | 4,38E-04 | *-* | *PJA2* | intragenic | Aggressive behavior | Pappa et al., 2016 |
| rs79087387 | 5 | 108884589 | 1,34E-05 | - | *-* | - | Aggressive behavior | Brevik et al., 2016 |
| rs76593371 | 5 | 108890608 | 1,31E-05 | - | *-* | - | Aggressive behavior | Brevik et al., 2016 |
| rs138474885 | 5 | 108914912 | 1,23E-05 | - | *-* | - | Aggressive behavior | Brevik et al., 2016 |
| rs9326778 | 5 | 108957496 | 7,80E-06 | - | *MAN2A1* | 95,6 | Aggressive behavior | Brevik et al., 2016 |
| rs76037120 | 5 | 108961380 | 2,87E-06 | - | *MAN2A1* | 91,7 | Aggressive behavior | Brevik et al., 2016 |
| rs36049337 | 5 | 114408284 | 1,43E-05 | - | *TRIM36* | 80,1 | Aggressive behavior | Brevik et al., 2016 |
| rs2134181 | 5 | 123273895 | 3,35E-04 | *-* | *-* | - | Aggressive behavior | Pappa et al., 2016 |
| rs2255981 | 5 | 123275486 | 3,54E-04 | *-* | *-* | - | Aggressive behavior | Pappa et al., 2016 |
| rs10067711 | 5 | 128141781 | 2,63E-05 | *-* | *-* | - | Aggressive behavior | Pappa et al., 2016 |
| rs6874556 | 5 | 132619430 | 4.7E-05 | - | *FSTL4* | intragenic | Anger (reaction) | Mick et al., 2014 |
| rs6884385 | 5 | 136881488 | 3.3E-05 | - | *SPOCK1* | 18.6 | Anger | Merjonen et al., 2011 |
| rs4835656 | 5 | 136889390 | 9.8E-06 | - | *SPOCK1* | 26.5 | Anger | Merjonen et al., 2011 |
| rs7714307 | 5 | 136897118 | 9.8E-06 | - | *SPOCK1* | 34.2 | Anger | Merjonen et al., 2011 |
| rs723546 | 5 | 136909905 | 9.7E-06 | - | *SPOCK1* | 47.0 | Anger | Merjonen et al., 2011 |
| rs6877688 | 5 | 136910152 | 9.7E-06 | - | *SPOCK1* | 47.3 | Anger | Merjonen et al., 2011 |
| rs10491335 | 5 | 136919055 | 9.6E-06 | - | *SPOCK1* | 56.2 | Anger | Merjonen et al., 2011 |
| rs1989919 | 5 | 136925491 | 3.1E-05 | - | *KLHL3* | 55.6 | Anger | Merjonen et al., 2011 |
| rs13156862 | 5 | 136927767 | 3.0E-05 | - | *KLHL3* | 53.3 | Anger | Merjonen et al., 2011 |
| rs255997 | 5 | 137917404 | 1,14E-05 | - | *HSPA9* | 1,5 | Aggressive behavior | Brevik et al., 2016 |
| rs740477 | 5 | 144377417 | 1.2E-05 | - | *-* | - | Anger | Merjonen et al., 2011 |
| rs740476 | 5 | 144377773 | 1.2E-05 | - | *-* | - | Anger | Merjonen et al., 2011 |
| rs12153546 | 5 | 144380810 | 1.2E-05 | - | *-* | - | Anger | Merjonen et al., 2011 |
| rs11747613 | 5 | 144384972 | 5.6E-06 | - | *-* | - | Anger | Merjonen et al., 2011 |
| rs12655091 | 5 | 144392528 | 1.2E-05 | - | *-* | - | Anger | Merjonen et al., 2011 |
| rs17455649 | 5 | 144406148 | 1.2E-05 | - | *-* | - | Anger | Merjonen et al., 2011 |
| rs1469072 | 5 | 144406362 | 1.2E-05 | - | *-* | - | Anger | Merjonen et al., 2011 |
| rs11955312 | 5 | 144409101 | 1.2E-05 | - | *-* | - | Anger | Merjonen et al., 2011 |
| rs17390089 | 5 | 144409764 | 1.2E-05 | - | *-* | - | Anger | Merjonen et al., 2011 |
| rs11167889 | 5 | 144410493 | 1.2E-05 | - | *-* | - | Anger | Merjonen et al., 2011 |
| rs6860134 | 5 | 144413524 | 1.6E-05 | - | *-* | - | Anger | Merjonen et al., 2011 |
| rs10515546 | 5 | 144416820 | 2.0E-05 | - | *-* | - | Anger | Merjonen et al., 2011 |
| rs13189940 | 5 | 144421366 | 1.9E-05 | - | *-* | - | Anger | Merjonen et al., 2011 |
| rs6892136 | 5 | 144421471 | 1.9E-05 | - | *-* | - | Anger | Merjonen et al., 2011 |
| rs966180 | 5 | 144423710 | 1.9E-05 | - | *-* | - | Anger | Merjonen et al., 2011 |
| rs13187785 | 5 | 144424477 | 1.8E-05 | - | *-* | - | Anger | Merjonen et al., 2011 |
| rs11954987 | 5 | 144427509 | 1.8E-05 | - | *-* | - | Anger | Merjonen et al., 2011 |
| rs13160635 | 5 | 144428770 | 2.7E-05 | - | *-* | - | Anger | Merjonen et al., 2011 |
| rs918515 | 5 | 144429193 | 2.7E-05 | - | *-* | - | Anger | Merjonen et al., 2011 |
| rs12153468 | 5 | 144429586 | 2.8E-05 | - | *-* | - | Anger | Merjonen et al., 2011 |
| rs6421158 | 5 | 144648812 | 1,73E-04 | *-* | *-* | - | Aggressive behavior | Pappa et al., 2016 |
| rs9637879 | 5 | 145915989 | 4,37E-05 | - | *PPP2R2B* | 33,3 | Aggressive behavior | Brevik et al., 2016 |
| rs113719485 | 5 | 145917478 | 3,76E-05 | - | *PPP2R2B* | 31,8 | Aggressive behavior | Brevik et al., 2016 |
| rs113432716 | 5 | 145918766 | 3,41E-05 | - | *PPP2R2B* | 30,5 | Aggressive behavior | Brevik et al., 2016 |
| rs112896656 | 5 | 145918937 | 3,38E-05 | - | *PPP2R2B* | 30,3 | Aggressive behavior | Brevik et al., 2016 |
| rs11956931 | 5 | 146663493 | 8.8E-06 | - | *STK32A* | intragenic | Antisocial behavior | Tielbeek et al., 2012 |
| rs13164049 | 5 | 152919093 | 4,40E-04 | *-* | *GRIA1* | intragenic | Aggressive behavior | Pappa et al., 2016 |
| rs867674 | 5 | 152921284 | 2,31E-04 | *-* | *GRIA1* | intragenic | Aggressive behavior | Pappa et al., 2016 |
| rs12522802 | 5 | 152922416 | 1,42E-04 | *-* | *GRIA1* | intragenic | Aggressive behavior | Pappa et al., 2016 |
| rs12520227 | 5 | 152922945 | 1,42E-04 | *-* | *GRIA1* | intragenic | Aggressive behavior | Pappa et al., 2016 |
| rs12520252 | 5 | 152923019 | 1,42E-04 | *-* | *GRIA1* | intragenic | Aggressive behavior | Pappa et al., 2016 |
| rs12522937 | 5 | 152923029 | 1,42E-04 | *-* | *GRIA1* | intragenic | Aggressive behavior | Pappa et al., 2016 |
| rs12153160 | 5 | 152931393 | 1,32E-04 | *-* | *GRIA1* | intragenic | Aggressive behavior | Pappa et al., 2016 |
| rs12515561 | 5 | 152935804 | 1,91E-04 | *-* | *GRIA1* | intragenic | Aggressive behavior | Pappa et al., 2016 |
| rs12515520 | 5 | 152935856 | 2,06E-04 | *-* | *GRIA1* | intragenic | Aggressive behavior | Pappa et al., 2016 |
| rs12515594 | 5 | 152936060 | 2,10E-04 | *-* | *GRIA1* | intragenic | Aggressive behavior | Pappa et al., 2016 |
| rs12520732 | 5 | 152936070 | 4,87E-04 | *-* | *GRIA1* | intragenic | Aggressive behavior | Pappa et al., 2016 |
| rs12520758 | 5 | 152936210 | 1,99E-04 | *-* | *GRIA1* | intragenic | Aggressive behavior | Pappa et al., 2016 |
| rs12515622 | 5 | 152936243 | 3,82E-04 | *-* | *GRIA1* | intragenic | Aggressive behavior | Pappa et al., 2016 |
| rs1552837 | 5 | 152939862 | 4,57E-04 | *-* | *GRIA1* | intragenic | Aggressive behavior | Pappa et al., 2016 |
| rs1552835 | 5 | 152939962 | 4,41E-04 | *-* | *GRIA1* | intragenic | Aggressive behavior | Pappa et al., 2016 |
| rs2131564 | 5 | 152940097 | 4,19E-04 | *-* | *GRIA1* | intragenic | Aggressive behavior | Pappa et al., 2016 |
| rs17519558 | 5 | 152940512 | 4,07E-04 | *-* | *GRIA1* | intragenic | Aggressive behavior | Pappa et al., 2016 |
| rs17519656 | 5 | 152940721 | 3,75E-04 | *-* | *GRIA1* | intragenic | Aggressive behavior | Pappa et al., 2016 |
| rs17591636 | 5 | 152940808 | 4,13E-04 | *-* | *GRIA1* | intragenic | Aggressive behavior | Pappa et al., 2016 |
| rs17054414 | 5 | 156618618 | 3,05E-04 | *-* | *ITK* | 3,9 | Aggressive behavior | Pappa et al., 2016 |
| rs4588578 | 5 | 157548215 | 4,34E-04 | *-* | *-* | - | Aggressive behavior | Pappa et al., 2016 |
| rs17328441 | 5 | 164427451 | 3,40E-04 | *-* | *-* | - | Aggressive behavior | Pappa et al., 2016 |
| rs17067247 | 5 | 165957746 | 2,75E-05 | - | *-* | - | ODD | Aebi et al., 2016 |
| rs6879854 | 5 | 166165061 | 1.9E-05 | - | *-* |  | Anger (temper) | Mick et al., 2014 |
| rs792757 | 5 | 172097329 | 2,81E-04 | *-* | *DUSP1* | 30,4 | Aggressive behavior | Pappa et al., 2016 |
| rs17648108 | 5 | 177764162 | 2.9E-05 | - | *COL23A1* | intragenic | Anger | Merjonen et al., 2011 |
| rs13211623 | 6 | 715769 | 4,78E-04 | *-* | *EXOC2* | 77,6 | Aggressive behavior | Pappa et al., 2016 |
| rs9502559 | 6 | 716389 | 4,50E-04 | *-* | *EXOC2* | 78,3 | Aggressive behavior | Pappa et al., 2016 |
| rs7762873 | 6 | 716504 | 4,31E-04 | *-* | *EXOC2* | 78,4 | Aggressive behavior | Pappa et al., 2016 |
| rs6597261 | 6 | 722973 | 2,78E-05 | *-* | *EXOC2* | 84,8 | Aggressive behavior | Pappa et al., 2016 |
| rs13219492 | 6 | 725441 | 2,48E-05 | *-* | *EXOC2* | 87,3 | Aggressive behavior | Pappa et al., 2016 |
| rs17239807 | 6 | 729680 | 2,10E-04 | *-* | *EXOC2* | 91,5 | Aggressive behavior | Pappa et al., 2016 |
| rs12198560 | 6 | 732703 | 2,99E-04 | *-* | *EXOC2* | 94,6 | Aggressive behavior | Pappa et al., 2016 |
| rs9328417 | 6 | 733169 | 2,09E-04 | *-* | *EXOC2* | 95,0 | Aggressive behavior | Pappa et al., 2016 |
| rs4960302 | 6 | 733882 | 3,05E-04 | *-* | *EXOC2* | 95,7 | Aggressive behavior | Pappa et al., 2016 |
| rs12197967 | 6 | 734572 | 2,25E-04 | *-* | *EXOC2* | 96,4 | Aggressive behavior | Pappa et al., 2016 |
| rs2873398 | 6 | 735302 | 3,35E-04 | *-* | *EXOC2* | 97,2 | Aggressive behavior | Pappa et al., 2016 |
| rs2438068 | 6 | 1205672 | 3,93E-05 | - | *FOXQ1* | 52,0 | Aggressive behavior | Brevik et al., 2016 |
| rs2496305 | 6 | 1206007 | 3,44E-05 | - | *FOXQ1* | 51,7 | Aggressive behavior | Brevik et al., 2016 |
| rs2064310 | 6 | 3025323 | 3.5E-05 | - | *RIPK1* | intragenic | Anger (reaction) | Mick et al., 2014 |
| rs583807 | 6 | 4100791 | 2.0E-05 | - | *PECI* | 20.0 | Anger (temper) | Mick et al., 2014 |
| rs13203027 | 6 | 4434277 | 3,73E-04 | *-* | *-* | - | Aggressive behavior | Pappa et al., 2016 |
| rs2181017 | 6 | 4435629 | 3,79E-04 | *-* | *-* | - | Aggressive behavior | Pappa et al., 2016 |
| rs9328254 | 6 | 4435821 | 3,59E-04 | *-* | *-* | - | Aggressive behavior | Pappa et al., 2016 |
| rs1265248 | 6 | 4436514 | 2,70E-04 | *-* | *-* | - | Aggressive behavior | Pappa et al., 2016 |
| rs7764381 | 6 | 4438656 | 3,44E-04 | *-* | *-* | - | Aggressive behavior | Pappa et al., 2016 |
| rs7743833 | 6 | 4438768 | 3,26E-04 | *-* | *-* | - | Aggressive behavior | Pappa et al., 2016 |
| rs3804482 | 6 | 6579204 | 4,69E-04 | *-* | *LY86* | intragenic | Aggressive behavior | Pappa et al., 2016 |
| rs3804483 | 6 | 6582676 | 2,80E-04 | *-* | *LY86* | intragenic | Aggressive behavior | Pappa et al., 2016 |
| rs3804484 | 6 | 6582809 | 2,75E-04 | *-* | *LY86* | intragenic | Aggressive behavior | Pappa et al., 2016 |
| rs3804486 | 6 | 6582994 | 4,21E-04 | *-* | *LY86* | intragenic | Aggressive behavior | Pappa et al., 2016 |
| rs9392817 | 6 | 6583442 | 2,94E-04 | *-* | *LY86* | intragenic | Aggressive behavior | Pappa et al., 2016 |
| rs2064236 | 6 | 6584424 | 2,91E-04 | *-* | *LY86* | intragenic | Aggressive behavior | Pappa et al., 2016 |
| rs4246077 | 6 | 6584568 | 2,65E-04 | *-* | *LY86* | intragenic | Aggressive behavior | Pappa et al., 2016 |
| rs12206199 | 6 | 6584860 | 2,37E-04 | *-* | *LY86* | intragenic | Aggressive behavior | Pappa et al., 2016 |
| rs12213188 | 6 | 6584948 | 2,00E-04 | *-* | *LY86* | intragenic | Aggressive behavior | Pappa et al., 2016 |
| rs9392818 | 6 | 6586034 | 8,52E-05 | *-* | *LY86* | intragenic | Aggressive behavior | Pappa et al., 2016 |
| rs9502485 | 6 | 6589462 | 7,45E-05 | *-* | *LY86* | intragenic | Aggressive behavior | Pappa et al., 2016 |
| rs9328380 | 6 | 6593383 | 1,64E-04 | *-* | *LY86* | intragenic | Aggressive behavior | Pappa et al., 2016 |
| rs4959501 | 6 | 8752899 | 4.2E-05 | - | *-* | - | Antisocial behavior | Tielbeek et al., 2012 |
| rs969527 | 6 | 10094971 | 1,76E-04 | *-* | *OFCC1* | intragenic | Aggressive behavior | Pappa et al., 2016 |
| rs9465606 | 6 | 20097652 | 2,15E-04 | *-* | *-* | - | Aggressive behavior | Pappa et al., 2016 |
| rs7749480 | 6 | 20108027 | 1,58E-04 | *-* | *-* | - | Aggressive behavior | Pappa et al., 2016 |
| rs555017 | 6 | 20293030 | 8.8E-06 | - | *MBOAT1* | intragenic | Anger (reaction) | Mick et al., 2014 |
| rs17835633 | 6 | 21252098 | 3.1E-03 | 4.2E-03 | *CDKAL1* | intragenic | AB+/CU+ | Viding et al., 2010 |
| rs6927510 | 6 | 22058950 | 3,60E-04 | *-* | *-* | - | Aggressive behavior | Pappa et al., 2016 |
| rs9257148 | 6 | 28839788 | 2,29E-04 | *-* | *-* | - | Aggressive behavior | Pappa et al., 2016 |
| rs9368576 | 6 | 28880329 | 9,22E-05 | *-* | *TRIM27* | 98,4 | Aggressive behavior | Pappa et al., 2016 |
| rs10484543 | 6 | 28899181 | 6,39E-05 | *-* | *TRIM27* | 79,6 | Aggressive behavior | Pappa et al., 2016 |
| rs209177 | 6 | 28911082 | 8,06E-06 | *-* | *TRIM27* | 67,7 | Aggressive behavior | Pappa et al., 2016 |
| rs209165 | 6 | 28937465 | 2,63E-05 | *-* | *TRIM27* | 41,3 | Aggressive behavior | Pappa et al., 2016 |
| rs9257280 | 6 | 28941486 | 3,25E-04 | *-* | *TRIM27* | 37,3 | Aggressive behavior | Pappa et al., 2016 |
| rs209151 | 6 | 28954703 | 6,10E-06 | *-* | *TRIM27* | 24,1 | Aggressive behavior | Pappa et al., 2016 |
| rs2006758 | 6 | 28958361 | 3,53E-05 | *-* | *TRIM27* | 20,4 | Aggressive behavior | Pappa et al., 2016 |
| rs1016472 | 6 | 28959346 | 5,87E-06 | *-* | *TRIM27* | 19,4 | Aggressive behavior | Pappa et al., 2016 |
| rs9257319 | 6 | 28959616 | 2,31E-04 | *-* | *TRIM27* | 19,1 | Aggressive behavior | Pappa et al., 2016 |
| rs429369 | 6 | 28959887 | 5,22E-06 | *-* | *TRIM27* | 18,9 | Aggressive behavior | Pappa et al., 2016 |
| rs7750338 | 6 | 28961136 | 4,41E-06 | *-* | *TRIM27* | 17,6 | Aggressive behavior | Pappa et al., 2016 |
| rs2148007 | 6 | 28964429 | 2,96E-05 | *-* | *TRIM27* | 14,3 | Aggressive behavior | Pappa et al., 2016 |
| rs209146 | 6 | 28966829 | 2,68E-06 | *-* | *TRIM27* | 11,9 | Aggressive behavior | Pappa et al., 2016 |
| rs209140 | 6 | 28971448 | 2,69E-06 | *-* | *TRIM27* | 7,3 | Aggressive behavior | Pappa et al., 2016 |
| rs209131 | 6 | 28975734 | 2,71E-06 | *-* | *TRIM27* | 3,0 | Aggressive behavior | Pappa et al., 2016 |
| rs2269553 | 6 | 28984488 | 4,95E-05 | *-* | *TRIM27* | intragenic | Aggressive behavior | Pappa et al., 2016 |
| rs209121 | 6 | 28989639 | 2,20E-06 | *-* | *TRIM27* | intragenic | Aggressive behavior | Pappa et al., 2016 |
| rs929042 | 6 | 28998779 | 4,63E-05 | *-* | *TRIM27* | intragenic | Aggressive behavior | Pappa et al., 2016 |
| rs2015436 | 6 | 29004893 | 1,57E-06 | *-* | *TRIM27* | 5,1 | Aggressive behavior | Pappa et al., 2016 |
| rs6912843 | 6 | 29012141 | 1,61E-06 | *-* | *TRIM27* | 12,4 | Aggressive behavior | Pappa et al., 2016 |
| rs3135329 | 6 | 29013083 | 4,96E-04 | *-* | *TRIM27* | 13,3 | Aggressive behavior | Pappa et al., 2016 |
| rs4713186 | 6 | 29017444 | 2,60E-04 | *-* | *TRIM27* | 17,7 | Aggressive behavior | Pappa et al., 2016 |
| rs2071789 | 6 | 29020055 | 3,42E-04 | *-* | *TRIM27* | 20,3 | Aggressive behavior | Pappa et al., 2016 |
| rs4947339 | 6 | 29024231 | 2,83E-04 | *-* | *TRIM27* | 24,5 | Aggressive behavior | Pappa et al., 2016 |
| rs6456876 | 6 | 29026915 | 2,61E-04 | *-* | *TRIM27* | 27,2 | Aggressive behavior | Pappa et al., 2016 |
| rs6901599 | 6 | 29034724 | 2,27E-04 | *-* | *TRIM27* | 35,0 | Aggressive behavior | Pappa et al., 2016 |
| rs9257425 | 6 | 29044535 | 1,81E-06 | *-* | *ZNF311* | 26,0 | Aggressive behavior | Pappa et al., 2016 |
| rs16894533 | 6 | 29053501 | 8,12E-05 | *-* | *ZNF311* | 17,1 | Aggressive behavior | Pappa et al., 2016 |
| rs6904975 | 6 | 29060014 | 1,92E-04 | *-* | *ZNF311* | 10,6 | Aggressive behavior | Pappa et al., 2016 |
| rs9257449 | 6 | 29065524 | 3,76E-04 | *-* | *ZNF311* | 5,0 | Aggressive behavior | Pappa et al., 2016 |
| rs6456880 | 6 | 29071227 | 3,32E-04 | *-* | *ZNF311* | intragenic | Aggressive behavior | Pappa et al., 2016 |
| rs2269555 | 6 | 29072826 | 4,63E-04 | *-* | *ZNF311* | intragenic | Aggressive behavior | Pappa et al., 2016 |
| rs6920392 | 6 | 29075017 | 3,91E-04 | *-* | *ZNF311* | intragenic | Aggressive behavior | Pappa et al., 2016 |
| rs6906909 | 6 | 29076125 | 3,89E-04 | *-* | *ZNF311* | intragenic | Aggressive behavior | Pappa et al., 2016 |
| rs6456883 | 6 | 29078348 | 3,84E-04 | *-* | *ZNF311* | intragenic | Aggressive behavior | Pappa et al., 2016 |
| rs9257455 | 6 | 29078724 | 1,85E-04 | *-* | *ZNF311* | intragenic | Aggressive behavior | Pappa et al., 2016 |
| rs9257461 | 6 | 29085904 | 4,62E-04 | *-* | *ZNF311* | 4,9 | Aggressive behavior | Pappa et al., 2016 |
| rs6916161 | 6 | 29091825 | 3,83E-04 | *-* | *ZNF311* | 10,8 | Aggressive behavior | Pappa et al., 2016 |
| rs16894557 | 6 | 29107805 | 4,94E-04 | *-* | *OR2W1* | 12,2 | Aggressive behavior | Pappa et al., 2016 |
| rs999265 | 6 | 29149782 | 3,57E-04 | *-* | *OR2B3P* | 12,3 | Aggressive behavior | Pappa et al., 2016 |
| rs2064365 | 6 | 29154399 | 4,66E-04 | *-* | *OR2B3P* | 7,7 | Aggressive behavior | Pappa et al., 2016 |
| rs12665108 | 6 | 29170173 | 4,61E-04 | *-* | *OR2B3P* | 7,2 | Aggressive behavior | Pappa et al., 2016 |
| rs3131087 | 6 | 29176738 | 4,41E-04 | *-* | *OR2J3* | 10,9 | Aggressive behavior | Pappa et al., 2016 |
| rs3130762 | 6 | 29185766 | 3,63E-04 | *-* | *OR2J3* | 1,9 | Aggressive behavior | Pappa et al., 2016 |
| rs3116838 | 6 | 29186130 | 3,55E-04 | *-* | *OR2J3* | 1,5 | Aggressive behavior | Pappa et al., 2016 |
| rs3130764 | 6 | 29188328 | 2,28E-04 | *-* | *OR2J3* | intragenic | Aggressive behavior | Pappa et al., 2016 |
| rs3130714 | 6 | 29216708 | 3,46E-04 | *-* | *OR2J3* | 28,1 | Aggressive behavior | Pappa et al., 2016 |
| rs3116847 | 6 | 29226792 | 2,32E-04 | *-* | *OR2J2* | 22,5 | Aggressive behavior | Pappa et al., 2016 |
| rs2394520 | 6 | 29245199 | 4,11E-04 | *-* | *OR2J2* | 4,1 | Aggressive behavior | Pappa et al., 2016 |
| rs3129156 | 6 | 29248196 | 1,95E-04 | *-* | *OR2J2* | 1,1 | Aggressive behavior | Pappa et al., 2016 |
| rs2394546 | 6 | 29283578 | 9,95E-05 | *-* | *OR2J2* | 33,2 | Aggressive behavior | Pappa et al., 2016 |
| rs1883329 | 6 | 29285081 | 1,40E-04 | *-* | *OR2J2* | 34,7 | Aggressive behavior | Pappa et al., 2016 |
| rs3116831 | 6 | 29286117 | 1,68E-04 | *-* | *OR2J2* | 35,8 | Aggressive behavior | Pappa et al., 2016 |
| rs3116832 | 6 | 29287124 | 9,78E-05 | *-* | *OR2J2* | 36,8 | Aggressive behavior | Pappa et al., 2016 |
| rs3130826 | 6 | 29289293 | 1,64E-04 | *-* | *OR2J2* | 39,0 | Aggressive behavior | Pappa et al., 2016 |
| rs3116837 | 6 | 29292639 | 1,43E-04 | *-* | *OR2J2* | 42,3 | Aggressive behavior | Pappa et al., 2016 |
| rs12660419 | 6 | 29299527 | 2,94E-04 | *-* | *LOC651503* | 38,9 | Aggressive behavior | Pappa et al., 2016 |
| rs3130815 | 6 | 29309629 | 5,79E-05 | *-* | *LOC651503* | 28,8 | Aggressive behavior | Pappa et al., 2016 |
| rs9257656 | 6 | 29337125 | 6,10E-05 | *-* | *LOC651503* | 1,3 | Aggressive behavior | Pappa et al., 2016 |
| rs9257657 | 6 | 29339151 | 2,01E-05 | *-* | *LOC651503* | intragenic | Aggressive behavior | Pappa et al., 2016 |
| rs9257660 | 6 | 29348258 | 5,82E-05 | *-* | *LOC651503* | 8,4 | Aggressive behavior | Pappa et al., 2016 |
| rs16867628 | 6 | 29374476 | 1,88E-04 | *-* | *OR14J1* | 8,0 | Aggressive behavior | Pappa et al., 2016 |
| rs9357086 | 6 | 29886008 | 2.9E-05 | - | *HLA-G* | 16.7 | Anger | Merjonen et al., 2011 |
| rs9261494 | 6 | 30218503 | 4,29E-04 | *-* | *TRIM40* | intragenic | Aggressive behavior | Pappa et al., 2016 |
| rs210122 | 6 | 33682613 | 4,61E-04 | *-* | *FLJ43752* | 13,5 | Aggressive behavior | Pappa et al., 2016 |
| rs6905353 | 6 | 33921021 | 4,12E-04 | *-* | *MLN* | 41,2 | Aggressive behavior | Pappa et al., 2016 |
| rs6931760 | 6 | 33921949 | 3,71E-04 | *-* | *MLN* | 42,2 | Aggressive behavior | Pappa et al., 2016 |
| rs1577823 | 6 | 63643246 | 4,99E-05 | - | *-* | - | Aggressive behavior | Brevik et al., 2016 |
| rs7739136 | 6 | 63647800 | 4,91E-05 | - | *-* | - | Aggressive behavior | Brevik et al., 2016 |
| rs7773896 | 6 | 63648071 | 4,95E-05 | - | *-* | - | Aggressive behavior | Brevik et al., 2016 |
| rs9343307 | 6 | 63648371 | 4,94E-05 | - | *-* | - | Aggressive behavior | Brevik et al., 2016 |
| rs9343308 | 6 | 63648656 | 4,74E-05 | - | *-* | - | Aggressive behavior | Brevik et al., 2016 |
| rs4579334 | 6 | 63652910 | 4,46E-05 | - | *-* | - | Aggressive behavior | Brevik et al., 2016 |
| rs1538086 | 6 | 63652922 | 4,52E-05 | - | *-* | - | Aggressive behavior | Brevik et al., 2016 |
| rs9359136 | 6 | 63656989 | 3,20E-05 | - | *-* | - | Aggressive behavior | Brevik et al., 2016 |
| rs13202756 | 6 | 67856645 | 1.6E-06 | - | *-* | - | Antisocial behavior | Tielbeek et al., 2012 |
| rs9343557 | 6 | 77845553 | 4.8E-03 | 4.0E-04 | *-* | - | AB+/CU+ | Viding et al., 2010 |
| rs2575211 | 6 | 80222649 | 3,30E-05 | - | *LCA5* | 28,8 | Aggressive behavior | Brevik et al., 2016 |
| rs9448720 | 6 | 80224621 | 2,74E-05 | - | *LCA5* | 26,8 | Aggressive behavior | Brevik et al., 2016 |
| rs9448722 | 6 | 80224754 | 1,55E-05 | - | *LCA5* | 26,7 | Aggressive behavior | Brevik et al., 2016 |
| rs9341778 | 6 | 80225205 | 4,05E-05 | - | *LCA5* | 26,2 | Aggressive behavior | Brevik et al., 2016 |
| rs2437701 | 6 | 80227427 | 3,44E-05 | - | *LCA5* | 24,0 | Aggressive behavior | Brevik et al., 2016 |
| rs2575210 | 6 | 80227521 | 3,43E-05 | - | *LCA5* | 23,9 | Aggressive behavior | Brevik et al., 2016 |
| rs2575209 | 6 | 80228179 | 3,50E-05 | - | *LCA5* | 23,3 | Aggressive behavior | Brevik et al., 2016 |
| rs2575206 | 6 | 80228881 | 2,93E-05 | - | *LCA5* | 22,6 | Aggressive behavior | Brevik et al., 2016 |
| rs2575205 | 6 | 80230471 | 2,00E-05 | - | *LCA5* | 21,0 | Aggressive behavior | Brevik et al., 2016 |
| rs2629469 | 6 | 80244232 | 1,32E-05 | - | *LCA5* | 7,2 | Aggressive behavior | Brevik et al., 2016 |
| rs16890791 | 6 | 80244701 | 1,17E-05 | - | *LCA5* | 6,7 | Aggressive behavior | Brevik et al., 2016 |
| rs35548514 | 6 | 80244877 | 4,98E-05 | - | *LCA5* | 6,5 | Aggressive behavior | Brevik et al., 2016 |
| rs2437700 | 6 | 80245679 | 2,56E-05 | - | *LCA5* | 5,7 | Aggressive behavior | Brevik et al., 2016 |
| rs2655668 | 6 | 80248839 | 3,09E-05 | - | *LCA5* | 2,6 | Aggressive behavior | Brevik et al., 2016 |
| rs2803190 | 6 | 80249025 | 3,23E-05 | - | *LCA5* | 2,4 | Aggressive behavior | Brevik et al., 2016 |
| rs287832 | 6 | 83504077 | 2,63E-04 | *-* | *-* | - | Aggressive behavior | Pappa et al., 2016 |
| rs293507 | 6 | 83700252 | 4,81E-04 | *-* | *UBE2CBP* | intragenic | Aggressive behavior | Pappa et al., 2016 |
| rs75588203 | 6 | 97201585 | 1,03E-05 | - | *FHL5* | 30,4 | Aggressive behavior | Brevik et al., 2016 |
| rs12203702 | 6 | 105284578 | 4.2E-05 | - | *HACE1* | intragenic | Anger | Merjonen et al., 2011 |
| rs9391253 | 6 | 105474309 | 2,14E-04 | *-* | *LIN28B* | 37,3 | Aggressive behavior | Pappa et al., 2016 |
| rs7759938 | 6 | 105485647 | 1,72E-04 | *-* | *LIN28B* | 26,0 | Aggressive behavior | Pappa et al., 2016 |
| rs2095812 | 6 | 105490671 | 2,09E-04 | *-* | *LIN28B* | 20,9 | Aggressive behavior | Pappa et al., 2016 |
| rs314263 | 6 | 105499438 | 1,31E-04 | *-* | *LIN28B* | 12,2 | Aggressive behavior | Pappa et al., 2016 |
| rs395962 | 6 | 105504111 | 1,39E-04 | *-* | *LIN28B* | 7,5 | Aggressive behavior | Pappa et al., 2016 |
| rs314277 | 6 | 105514355 | 4,66E-04 | *-* | *LIN28B* | intragenic | Aggressive behavior | Pappa et al., 2016 |
| rs314276 | 6 | 105514692 | 2,77E-05 | *-* | *LIN28B* | intragenic | Aggressive behavior | Pappa et al., 2016 |
| rs167539 | 6 | 105516741 | 2,43E-05 | *-* | *LIN28B* | intragenic | Aggressive behavior | Pappa et al., 2016 |
| rs314268 | 6 | 105524671 | 2,04E-05 | *-* | *LIN28B* | intragenic | Aggressive behavior | Pappa et al., 2016 |
| rs369065 | 6 | 105550751 | 2,34E-05 | *-* | *LIN28B* | intragenic | Aggressive behavior | Pappa et al., 2016 |
| rs314273 | 6 | 105568575 | 2,53E-05 | *-* | *LIN28B* | intragenic | Aggressive behavior | Pappa et al., 2016 |
| rs4946818 | 6 | 107572411 | 1,86E-04 | *-* | *PDSS2* | 8,0 | Aggressive behavior | Pappa et al., 2016 |
| rs13202332 | 6 | 107573323 | 6,63E-06 | *-* | *PDSS2* | 7,1 | Aggressive behavior | Pappa et al., 2016 |
| rs6568468 | 6 | 107579642 | 2,31E-04 | *-* | *PDSS2* | 0,8 | Aggressive behavior | Pappa et al., 2016 |
| rs9372149 | 6 | 107579911 | 1,19E-06 | *-* | *PDSS2* | 0,5 | Aggressive behavior | Pappa et al., 2016 |
| rs6938393 | 6 | 107591228 | 1,05E-04 | *-* | *PDSS2* | intragenic | Aggressive behavior | Pappa et al., 2016 |
| rs9398117 | 6 | 107592051 | 8,04E-05 | *-* | *PDSS2* | intragenic | Aggressive behavior | Pappa et al., 2016 |
| rs9486548 | 6 | 107604523 | 2,64E-04 | *-* | *PDSS2* | intragenic | Aggressive behavior | Pappa et al., 2016 |
| rs9486549 | 6 | 107604530 | 8,24E-05 | *-* | *PDSS2* | intragenic | Aggressive behavior | Pappa et al., 2016 |
| rs9374291 | 6 | 112221484 | 5.4E-07 | - | *FYN* | intragenic | Anger (temper) | Mick et al., 2014 |
| rs6919306 | 6 | 112226312 | 8.1E-07 | - | *FYN* | intragenic | Anger (temper) | Mick et al., 2014 |
| rs1022649 | 6 | 112226674 | 1.6E-06 | - | *FYN* | intragenic | Anger (temper) | Mick et al., 2014 |
| rs2148710 | 6 | 112228919 | 4.6E-07 | - | *FYN* | intragenic | Anger (temper) | Mick et al., 2014 |
| rs7752620 | 6 | 112244987 | 1.0E-06 | - | *FYN* | intragenic | Anger (temper) | Mick et al., 2014 |
| rs2182644 | 6 | 112245575 | 2.4E-05 | - | *FYN* | intragenic | Anger (temper) | Mick et al., 2014 |
| rs7752077 | 6 | 112253800 | 8.8E-07 | - | *FYN* | intragenic | Anger (temper) | Mick et al., 2014 |
| rs3734457 | 6 | 114287364 | 3,19E-04 | *-* | *MARCKS* | intragenic | Aggressive behavior | Pappa et al., 2016 |
| rs12663908 | 6 | 115189232 | 1,25E-04 | *-* | *-* | - | Aggressive behavior | Pappa et al., 2016 |
| rs12664090 | 6 | 115201602 | 1,36E-04 | *-* | *-* | - | Aggressive behavior | Pappa et al., 2016 |
| rs12661361 | 6 | 115254659 | 1,01E-04 | *-* | *-* | - | Aggressive behavior | Pappa et al., 2016 |
| rs666019 | 6 | 117143096 | 4,57E-04 | *-* | *KPNA5* | intragenic | Aggressive behavior | Pappa et al., 2016 |
| rs665499 | 6 | 117149805 | 3,24E-04 | *-* | *KPNA5* | intragenic | Aggressive behavior | Pappa et al., 2016 |
| rs4538743 | 6 | 118490825 | 4,78E-04 | *-* | *SLC35F1* | intragenic | Aggressive behavior | Pappa et al., 2016 |
| rs7742473 | 6 | 119208433 | 2.3E-05 | - | *ASF1A* | 55.2 | Anger (temper) | Mick et al., 2014 |
| rs9398797 | 6 | 126614266 | 3,98E-04 | *-* | *C6orf173* | 88,7 | Aggressive behavior | Pappa et al., 2016 |
| rs9401868 | 6 | 126616401 | 4,02E-04 | *-* | *C6orf173* | 86,6 | Aggressive behavior | Pappa et al., 2016 |
| rs4895804 | 6 | 126621076 | 3,97E-04 | *-* | *C6orf173* | 81,9 | Aggressive behavior | Pappa et al., 2016 |
| rs4895805 | 6 | 126621837 | 3,34E-04 | *-* | *C6orf173* | 81,1 | Aggressive behavior | Pappa et al., 2016 |
| rs9388476 | 6 | 126623026 | 3,91E-04 | *-* | *C6orf173* | 79,9 | Aggressive behavior | Pappa et al., 2016 |
| rs988490 | 6 | 126624904 | 4,73E-04 | *-* | *C6orf173* | 78,1 | Aggressive behavior | Pappa et al., 2016 |
| rs4895806 | 6 | 126628760 | 3,05E-04 | *-* | *C6orf173* | 74,2 | Aggressive behavior | Pappa et al., 2016 |
| rs9482734 | 6 | 126629661 | 3,17E-04 | *-* | *C6orf173* | 73,3 | Aggressive behavior | Pappa et al., 2016 |
| rs658308 | 6 | 126631631 | 4,94E-04 | *-* | *C6orf173* | 71,3 | Aggressive behavior | Pappa et al., 2016 |
| rs6916556 | 6 | 126633465 | 3,24E-04 | *-* | *C6orf173* | 69,5 | Aggressive behavior | Pappa et al., 2016 |
| rs643394 | 6 | 126635960 | 3,92E-04 | *-* | *C6orf173* | 67,0 | Aggressive behavior | Pappa et al., 2016 |
| rs4897174 | 6 | 126639081 | 3,64E-04 | *-* | *C6orf173* | 63,9 | Aggressive behavior | Pappa et al., 2016 |
| rs608669 | 6 | 126639347 | 4,07E-04 | *-* | *C6orf173* | 63,6 | Aggressive behavior | Pappa et al., 2016 |
| rs632735 | 6 | 126639476 | 4,08E-04 | *-* | *C6orf173* | 63,5 | Aggressive behavior | Pappa et al., 2016 |
| rs613489 | 6 | 126640553 | 3,67E-04 | *-* | *C6orf173* | 62,4 | Aggressive behavior | Pappa et al., 2016 |
| rs1455736 | 6 | 126641451 | 3,43E-04 | *-* | *C6orf173* | 61,5 | Aggressive behavior | Pappa et al., 2016 |
| rs6903939 | 6 | 126642226 | 4,86E-04 | *-* | *C6orf173* | 60,7 | Aggressive behavior | Pappa et al., 2016 |
| rs9388477 | 6 | 126643653 | 4,71E-04 | *-* | *C6orf173* | 59,3 | Aggressive behavior | Pappa et al., 2016 |
| rs636061 | 6 | 126644524 | 4,25E-04 | *-* | *C6orf173* | 58,5 | Aggressive behavior | Pappa et al., 2016 |
| rs593284 | 6 | 126649882 | 4,31E-04 | *-* | *C6orf173* | 53,1 | Aggressive behavior | Pappa et al., 2016 |
| rs13216682 | 6 | 129436356 | 3,26E-04 | *-* | *LAMA2* | intragenic | Aggressive behavior | Pappa et al., 2016 |
| rs73585571 | 6 | 129667050 | 4,13E-05 | - | *LAMA2* | intragenic | Aggressive behavior | Brevik et al., 2016 |
| rs12204350 | 6 | 129667122 | 3,96E-05 | - | *LAMA2* | intragenic | Aggressive behavior | Brevik et al., 2016 |
| rs10457520 | 6 | 129669502 | 4,42E-05 | - | *LAMA2* | intragenic | Aggressive behavior | Brevik et al., 2016 |
| rs3798662 | 6 | 129676119 | 4,42E-05 | - | *LAMA2* | intragenic | Aggressive behavior | Brevik et al., 2016 |
| rs12208367 | 6 | 129683067 | 4,76E-05 | - | *LAMA2* | intragenic | Aggressive behavior | Brevik et al., 2016 |
| rs12201387 | 6 | 129683203 | 4,02E-05 | - | *LAMA2* | intragenic | Aggressive behavior | Brevik et al., 2016 |
| rs12206166 | 6 | 129690509 | 4,49E-05 | - | *LAMA2* | intragenic | Aggressive behavior | Brevik et al., 2016 |
| rs12206081 | 6 | 129690510 | 4,49E-05 | - | *LAMA2* | intragenic | Aggressive behavior | Brevik et al., 2016 |
| rs12208111 | 6 | 129691831 | 4,04E-05 | - | *LAMA2* | intragenic | Aggressive behavior | Brevik et al., 2016 |
| rs9492302 | 6 | 129707479 | 2,80E-05 | - | *LAMA2* | intragenic | Aggressive behavior | Brevik et al., 2016 |
| rs1490390 | 6 | 129710696 | 4,21E-05 | - | *LAMA2* | intragenic | Aggressive behavior | Brevik et al., 2016 |
| rs9492305 | 6 | 129713366 | 2,69E-05 | - | *LAMA2* | intragenic | Aggressive behavior | Brevik et al., 2016 |
| rs9483012 | 6 | 129720216 | 3,74E-05 | - | *LAMA2* | intragenic | Aggressive behavior | Brevik et al., 2016 |
| rs11754670 | 6 | 129743345 | 2,56E-05 | - | *LAMA2* | intragenic | Aggressive behavior | Brevik et al., 2016 |
| rs58558234 | 6 | 129749227 | 2,07E-05 | - | *LAMA2* | intragenic | Aggressive behavior | Brevik et al., 2016 |
| rs9402839 | 6 | 137036903 | 1,68E-04 | *-* | *MAP3K5* | intragenic | Aggressive behavior | Pappa et al., 2016 |
| rs12215384 | 6 | 140047787 | 2,59E-05 | - | *-* | - | Aggressive behavior | Brevik et al., 2016 |
| rs9386227 | 6 | 148809529 | 2.0E-05 | - | *SASH1* | intragenic | Anger (temper) | Mick et al., 2014 |
| rs679249 | 6 | 150741788 | 5.6E-07 | - | *IYD* | intragenic | Anger (temper) | Mick et al., 2014 |
| rs7772011 | 6 | 150744080 | 3.0E-06 | - | *IYD* | intragenic | Anger (temper) | Mick et al., 2014 |
| rs670292 | 6 | 150744540 | 2.2E-07 | - | *IYD* | intragenic | Anger (temper) | Mick et al., 2014 |
| rs633945 | 6 | 150744998 | 5.0E-07 | - | *IYD* | intragenic | Anger (temper) | Mick et al., 2014 |
| rs1339111 | 6 | 150770910 | 7.6E-07 | - | *IYD* | 3.5 | Anger (temper) | Mick et al., 2014 |
| rs590179 | 6 | 150773348 | 1.1E-06 | - | *IYD* | 5.9 | Anger (temper) | Mick et al., 2014 |
| rs2057181 | 6 | 153395904 | 3,89E-04 | *-* | *RGS17* | intragenic | Aggressive behavior | Pappa et al., 2016 |
| rs994777 | 6 | 153405171 | 1,13E-04 | *-* | *RGS17* | intragenic | Aggressive behavior | Pappa et al., 2016 |
| rs9479488 | 6 | 153412876 | 5,64E-05 | *-* | *RGS17* | intragenic | Aggressive behavior | Pappa et al., 2016 |
| rs13217265 | 6 | 158121630 | 3.3E-05 | - | *SNX9* | 42.7 | Anger (reaction) | Mick et al., 2014 |
| rs316002 | 6 | 160563809 | 8.7E-06 | - | *SLC22A2* | intragenic | Anger (reaction) | Mick et al., 2014 |
| rs12111075 | 6 | 162224195 | 2.4E-05 | - | *PARK2* | intragenic | Anger | Merjonen et al., 2011 |
| rs12110765 | 6 | 162224235 | 2.2E-05 | - | *PARK2* | intragenic | Anger | Merjonen et al., 2011 |
| rs139748362 | 6 | 162811819 | 2,99E-05 | - | *PARK2* | intragenic | Aggressive behavior | Brevik et al., 2016 |
| rs73035881 | 6 | 162831120 | 2,10E-05 | - | *PARK2* | intragenic | Aggressive behavior | Brevik et al., 2016 |
| rs2038091 | 6 | 163630631 | 3.9E-05 | - | *PACRG* | intragenic | Anger (temper) | Mick et al., 2014 |
| rs4709094 | 6 | 166423628 | 2,34E-04 | *-* | *T* | 67,5 | Aggressive behavior | Pappa et al., 2016 |
| rs4710005 | 6 | 166425195 | 2,55E-04 | *-* | *T* | 65,9 | Aggressive behavior | Pappa et al., 2016 |
| rs9459565 | 6 | 166425516 | 2,56E-04 | *-* | *T* | 65,6 | Aggressive behavior | Pappa et al., 2016 |
| rs3127389 | 6 | 166430586 | 2,85E-04 | *-* | *T* | 60,5 | Aggressive behavior | Pappa et al., 2016 |
| rs9459601 | 6 | 166512749 | 8,19E-05 | *-* | *T* | 10,6 | Aggressive behavior | Pappa et al., 2016 |
| rs9364245 | 6 | 168499366 | 2,81E-05 | - | *DACT2* | 36,1 | Aggressive behavior | Brevik et al., 2016 |
| rs498475 | 7 | 28222765 | 2.9E-05 | - | *JAZF1* | 35.8 | Antisocial behavior | Tielbeek et al., 2012 |
| rs17158386 | 7 | 29771886 | 2,11E-05 | - | *WIPF3* | 69,0 | Aggressive behavior | Brevik et al., 2016 |
| rs7811079 | 7 | 31909609 | 1,14E-05 | - | *PDE1C* | intragenic | ODD | Aebi et al., 2016 |
| rs340370 | 7 | 35354647 | 2,93E-04 | *-* | *TBX20* | 94,9 | Aggressive behavior | Pappa et al., 2016 |
| rs6954895 | 7 | 35552731 | 4.7E-06 | - | *HERPUD2* | 86.1 | Anger (temper) | Mick et al., 2014 |
| rs28731457 | 7 | 35573561 | 7.9E-06 | - | *HERPUD2* | 65.2 | Anger (temper) | Mick et al., 2014 |
| rs62445316 | 7 | 36477607 | 4,18E-05 | - | *ANLN* | 17,7 | Aggressive behavior | Brevik et al., 2016 |
| rs12534609 | 7 | 48352448 | 2.4E-05 | - | *ABCA13* | intragenic | Anger (reaction) | Mick et al., 2014 |
| rs602148 | 7 | 51845912 | 3.7E-05 | - | *-* | - | Anger | Merjonen et al., 2011 |
| rs641056 | 7 | 51863474 | 4.5E-05 | - | *-* | - | Anger | Merjonen et al., 2011 |
| rs632691 | 7 | 51876141 | 4.4E-05 | - | *-* | - | Anger | Merjonen et al., 2011 |
| rs602319 | 7 | 51880476 | 4.6E-05 | - | *-* | - | Anger | Merjonen et al., 2011 |
| rs1029705 | 7 | 66763317 | 4,02E-04 | *-* | *-* | - | Aggressive behavior | Pappa et al., 2016 |
| rs4718658 | 7 | 67026053 | 3,32E-04 | *-* | *-* | - | Aggressive behavior | Pappa et al., 2016 |
| rs10271531 | 7 | 80951877 | 3.5E-05 | - | *-* | - | Anger (reaction) | Mick et al., 2014 |
| rs6467890 | 7 | 81773149 | 1,01E-04 | *-* | *CACNA2D1* | intragenic | Aggressive behavior | Pappa et al., 2016 |
| rs12670356 | 7 | 81840844 | 2,17E-04 | *-* | *CACNA2D1* | intragenic | Aggressive behavior | Pappa et al., 2016 |
| rs17156440 | 7 | 82035028 | 4,34E-04 | *-* | *-* | - | Aggressive behavior | Pappa et al., 2016 |
| rs13438454 | 7 | 82055535 | 3,59E-05 | *-* | *-* | - | Aggressive behavior | Pappa et al., 2016 |
| rs797820 | 7 | 83446975 | 8.0E-06 | - | *SEMA3A* | intragenic | CBCL-DP | Mick et al., 2011 |
| rs2538909 | 7 | 88109439 | 2,69E-04 | *-* | *-* | - | Aggressive behavior | Pappa et al., 2016 |
| rs1024507 | 7 | 88409364 | 3.3E-05 | - | *ZNF804B* | intragenic | CBCL-DP | Mick et al., 2011 |
| rs10488529 | 7 | 92491270 | 2.1E-05 | - | *SAMD9* | 75.5 | Antisocial behavior | Tielbeek et al., 2012 |
| rs727397 | 7 | 105739130 | 4,68E-04 | *-* | *NAMPT* | 26,3 | Aggressive behavior | Pappa et al., 2016 |
| rs17156981 | 7 | 109057005 | 2.4E-05 | - | *-* |  | Anger (temper) | Mick et al., 2014 |
| rs10244937 | 7 | 116365178 | 4.4E-05 | - | *ST7* | 15.4 | CBCL-DP | Mick et al., 2011 |
| rs1419428 | 7 | 127017512 | 1,03E-04 | *-* | *ARF5* | intragenic | Aggressive behavior | Pappa et al., 2016 |
| rs6975965 | 7 | 129409274 | 4,47E-04 | *-* | *UBE2H* | 29,2 | Aggressive behavior | Pappa et al., 2016 |
| rs13308940 | 7 | 130913621 | 2.9E-05 | - | *PODXL* | 21.7 | Anger (temper) | Mick et al., 2014 |
| rs6961544 | 7 | 130918747 | 3.3E-05 | - | *PODXL* | 26.8 | Anger (temper) | Mick et al., 2014 |
| rs6975650 | 7 | 133743514 | 3,78E-04 | *-* | *AKR1B1* | 34,1 | Aggressive behavior | Pappa et al., 2016 |
| rs1646747 | 7 | 133744565 | 3,02E-04 | *-* | *AKR1B1* | 33,1 | Aggressive behavior | Pappa et al., 2016 |
| rs11547028 | 7 | 142181218 | 1.7E-05 | - | *PRSS2* | intragenic | Antisocial behavior | Tielbeek et al., 2012 |
| rs6961306 | 7 | 148397324 | 4,86E-04 | *-* | *ZNF786* | 0,3 | Aggressive behavior | Pappa et al., 2016 |
| rs17530756 | 7 | 148836781 | 4,50E-04 | *-* | *ZNF746* | 11,1 | Aggressive behavior | Pappa et al., 2016 |
| rs34601482 | 7 | 154861691 | 2,54E-05 | - | *EN2* | 81,9 | Aggressive behavior | Brevik et al., 2016 |
| rs73490168 | 7 | 154862226 | 2,74E-05 | - | *EN2* | 81,4 | Aggressive behavior | Brevik et al., 2016 |
| rs6992825 | 8 | 2337642 | 4,19E-04 | *-* | *-* | - | Aggressive behavior | Pappa et al., 2016 |
| rs7846114 | 8 | 2337779 | 4,98E-04 | *-* | *-* | - | Aggressive behavior | Pappa et al., 2016 |
| rs6558665 | 8 | 2407574 | 3.0E-05 | - | *-* | - | Antisocial behavior | Tielbeek et al., 2012 |
| rs1350332 | 8 | 2409404 | 1.8E-05 | - | *-* | - | Antisocial behavior | Tielbeek et al., 2012 |
| rs6558666 | 8 | 2410854 | 2.9E-05 | - | *-* | - | Antisocial behavior | Tielbeek et al., 2012 |
| rs6558776 | 8 | 3287571 | 6.3E-06 | - | *CSMD1* | intragenic | Antisocial behavior | Tielbeek et al., 2012 |
| rs931200 | 8 | 3443506 | 5,38E-06 | - | *CSMD1* | intragenic | Aggressive behavior | Brevik et al., 2016 |
| rs80113388 | 8 | 3455051 | 2,61E-06 | - | *CSMD1* | intragenic | Aggressive behavior | Brevik et al., 2016 |
| rs60480742 | 8 | 5160645 | 3,32E-05 | - | *-* | - | Aggressive behavior | Brevik et al., 2016 |
| rs57375224 | 8 | 5160907 | 3,21E-05 | - | *-* | - | Aggressive behavior | Brevik et al., 2016 |
| rs11986426 | 8 | 5161250 | 3,66E-05 | - | *-* | - | Aggressive behavior | Brevik et al., 2016 |
| rs66915074 | 8 | 5161425 | 3,68E-05 | - | *-* | - | Aggressive behavior | Brevik et al., 2016 |
| rs66856205 | 8 | 5162613 | 3,97E-05 | - | *-* | - | Aggressive behavior | Brevik et al., 2016 |
| rs7816376 | 8 | 5163181 | 4,15E-05 | - | *-* | - | Aggressive behavior | Brevik et al., 2016 |
| rs6987022 | 8 | 5163772 | 4,46E-05 | - | *-* | - | Aggressive behavior | Brevik et al., 2016 |
| rs2959799 | 8 | 6461123 | 3,85E-05 | - | *MCPH1* | intragenic | ODD | Aebi et al., 2016 |
| rs2959798 | 8 | 6461154 | 3,94E-05 | - | *MCPH1* | intragenic | ODD | Aebi et al., 2016 |
| rs2454517 | 8 | 6461166 | 3,60E-05 | - | *MCPH1* | intragenic | ODD | Aebi et al., 2016 |
| rs2936501 | 8 | 6461544 | 3,85E-05 | - | *MCPH1* | intragenic | ODD | Aebi et al., 2016 |
| rs2013938 | 8 | 6463312 | 1,31E-05 | - | *MCPH1* | intragenic | ODD | Aebi et al., 2016 |
| rs2062002 | 8 | 13748732 | 2.6E-05 | - | *-* | - | Anger | Merjonen et al., 2011 |
| rs17117843 | 8 | 13749851 | 2.6E-05 | - | *-* | - | Anger | Merjonen et al., 2011 |
| rs7013476 | 8 | 13754066 | 1.8E-05 | - | *-* | - | Anger | Merjonen et al., 2011 |
| rs10101844 | 8 | 13759054 | 2.4E-05 | - | *-* | - | Anger | Merjonen et al., 2011 |
| rs1820825 | 8 | 15507610 | 4,99E-04 | *-* | *TUSC3* | intragenic | Aggressive behavior | Pappa et al., 2016 |
| rs2726960 | 8 | 27805313 | 6,87E-05 | *-* | *SCARA5* | intragenic | Aggressive behavior | Pappa et al., 2016 |
| rs2726975 | 8 | 27812173 | 8,34E-05 | *-* | *SCARA5* | intragenic | Aggressive behavior | Pappa et al., 2016 |
| rs1111619 | 8 | 27812359 | 8,23E-05 | *-* | *SCARA5* | intragenic | Aggressive behavior | Pappa et al., 2016 |
| rs10103840 | 8 | 29475632 | 2.5E-03 | 5.1E-04 | *-* | - | AB+/CU+ | Viding et al., 2010 |
| rs2725374 | 8 | 30949231 | 1.9E-05 | - | *PURG* | 23.6 | Anger | Merjonen et al., 2011 |
| rs9694122 | 8 | 30950350 | 9.1E-06 | - | *PURG* | 22.5 | Anger | Merjonen et al., 2011 |
| rs2543615 | 8 | 30957395 | 9.8E-06 | - | *PURG* | 15.5 | Anger | Merjonen et al., 2011 |
| rs11783562 | 8 | 30958088 | 6.4E-06 | - | *PURG* | 14.8 | Anger | Merjonen et al., 2011 |
| rs11778607 | 8 | 30959110 | 9.5E-06 | - | *PURG* | 13.8 | Anger | Merjonen et al., 2011 |
| rs17648656 | 8 | 30973921 | 4.6E-06 | - | *PURG* | intragenic | Anger | Merjonen et al., 2011 |
| rs1362910 | 8 | 30976006 | 1.8E-05 | - | *PURG* | intragenic | Anger | Merjonen et al., 2011 |
| rs11776713 | 8 | 30981149 | 4.6E-06 | - | *PURG* | intragenic | Anger | Merjonen et al., 2011 |
| rs11779521 | 8 | 30983843 | 4.6E-06 | - | *PURG* | intragenic | Anger | Merjonen et al., 2011 |
| rs11775287 | 8 | 30983881 | 4.7E-06 | - | *PURG* | intragenic | Anger | Merjonen et al., 2011 |
| rs2195588 | 8 | 40475857 | 7,88E-06 | - | *ZMAT4* | 31,4 | Aggressive behavior | Brevik et al., 2016 |
| rs11778329 | 8 | 49215718 | 7.0E-06 | - | *UBE2V2* | 78.7 | CBCL-DP | Mick et al., 2011 |
| rs13264368 | 8 | 55433967 | 3,86E-04 | *-* | *SOX17* | 99,1 | Aggressive behavior | Pappa et al., 2016 |
| rs3110145 | 8 | 60204965 | 2.4E-05 | - | *TOX* | 10.6 | Anger (reaction) | Mick et al., 2014 |
| rs10504394 | 8 | 67080797 | 4.9E-05 | - | *DNAJC5B* | 15.6 | Anger (reaction) | Mick et al., 2014 |
| rs1866897 | 8 | 70688850 | 3,99E-04 | *-* | *SULF1* | intragenic | Aggressive behavior | Pappa et al., 2016 |
| rs10808642 | 8 | 92936888 | 1,31E-04 | *-* | *-* | - | Aggressive behavior | Pappa et al., 2016 |
| rs4500123 | 8 | 93039329 | 5,93E-06 | - | *RUNX1T1* | 1,0 | ODD | Aebi et al., 2016 |
| rs4734962 | 8 | 93039798 | 5,93E-06 | - | *RUNX1T1* | 0,5 | ODD | Aebi et al., 2016 |
| rs10091060 | 8 | 95178351 | 2,32E-04 | *-* | *CDH17* | 30,2 | Aggressive behavior | Pappa et al., 2016 |
| rs2340015 | 8 | 95184445 | 2,77E-04 | *-* | *CDH17* | 24,1 | Aggressive behavior | Pappa et al., 2016 |
| rs10087687 | 8 | 95187542 | 2,81E-04 | *-* | *CDH17* | 21,0 | Aggressive behavior | Pappa et al., 2016 |
| rs16916528 | 8 | 95191694 | 2,72E-04 | *-* | *CDH17* | 16,9 | Aggressive behavior | Pappa et al., 2016 |
| rs4421324 | 8 | 95193232 | 2,74E-04 | *-* | *CDH17* | 15,3 | Aggressive behavior | Pappa et al., 2016 |
| rs745173 | 8 | 95194547 | 2,07E-04 | *-* | *CDH17* | 14,0 | Aggressive behavior | Pappa et al., 2016 |
| rs10100656 | 8 | 95198590 | 2,93E-04 | *-* | *CDH17* | 10,0 | Aggressive behavior | Pappa et al., 2016 |
| rs2513794 | 8 | 95199503 | 3,02E-04 | *-* | *CDH17* | 9,1 | Aggressive behavior | Pappa et al., 2016 |
| rs2249967 | 8 | 95200359 | 3,50E-04 | *-* | *CDH17* | 8,2 | Aggressive behavior | Pappa et al., 2016 |
| rs9417 | 8 | 95208942 | 4,85E-04 | *-* | *CDH17* | intragenic | Aggressive behavior | Pappa et al., 2016 |
| rs2514788 | 8 | 95212920 | 5.2E-03 | 1.5E-03 | *CDH17* | intragenic | AB+/CU+ | Viding et al., 2010 |
| rs2514800 | 8 | 95227033 | 4,61E-04 | *-* | *CDH17* | intragenic | Aggressive behavior | Pappa et al., 2016 |
| rs10504933 | 8 | 95228980 | 4,50E-04 | *-* | *CDH17* | intragenic | Aggressive behavior | Pappa et al., 2016 |
| rs17425970 | 8 | 101498872 | 4,96E-04 | *-* | *-* | - | Aggressive behavior | Pappa et al., 2016 |
| rs10505229 | 8 | 115908561 | 1,83E-04 | *-* | *-* | - | Aggressive behavior | Pappa et al., 2016 |
| rs7015522 | 8 | 115915163 | 1,87E-04 | *-* | *-* | - | Aggressive behavior | Pappa et al., 2016 |
| rs10107011 | 8 | 115918007 | 9,66E-05 | *-* | *-* | - | Aggressive behavior | Pappa et al., 2016 |
| rs10090449 | 8 | 117295170 | 2.6E-05 | - | *-* | - | Anger | Merjonen et al., 2011 |
| rs998245 | 8 | 117309055 | 1.1E-05 | - | *-* | - | Anger | Merjonen et al., 2011 |
| rs10505278 | 8 | 117341103 | 1.2E-05 | - | *-* | - | Anger | Merjonen et al., 2011 |
| rs7012323 | 8 | 127651009 | 3.6E-05 | - | *FAM84B* | 11.4 | Anger (reaction) | Mick et al., 2014 |
| rs1441990 | 8 | 130075654 | 5.4E-03 | 3.5E-03 | *-* | - | AB+/CU+ | Viding et al., 2010 |
| rs10123123 | 9 | 1174757 | 3.6E-05 | - | *-* | - | Anger | Merjonen et al., 2011 |
| rs1933041 | 9 | 1186586 | 3.4E-05 | - | *-* | - | Anger | Merjonen et al., 2011 |
| rs2281785 | 9 | 2171854 | 4,22E-04 | *-* | *SMARCA2* | intragenic | Aggressive behavior | Pappa et al., 2016 |
| rs10732343 | 9 | 2380378 | 2,45E-04 | *-* | *-* | - | Aggressive behavior | Pappa et al., 2016 |
| rs10811972 | 9 | 2382170 | 1,62E-04 | *-* | *-* | - | Aggressive behavior | Pappa et al., 2016 |
| rs10811975 | 9 | 2382227 | 1,32E-04 | *-* | *-* | - | Aggressive behavior | Pappa et al., 2016 |
| rs7849851 | 9 | 2382608 | 1,88E-04 | *-* | *-* | - | Aggressive behavior | Pappa et al., 2016 |
| rs10757450 | 9 | 2382703 | 1,55E-04 | *-* | *-* | - | Aggressive behavior | Pappa et al., 2016 |
| rs1331830 | 9 | 2383460 | 1,96E-04 | *-* | *-* | - | Aggressive behavior | Pappa et al., 2016 |
| rs1331832 | 9 | 2383681 | 1,90E-04 | *-* | *-* | - | Aggressive behavior | Pappa et al., 2016 |
| rs7470470 | 9 | 2383775 | 1,87E-04 | *-* | *-* | - | Aggressive behavior | Pappa et al., 2016 |
| rs2150836 | 9 | 2384242 | 1,82E-04 | *-* | *-* | - | Aggressive behavior | Pappa et al., 2016 |
| rs4741694 | 9 | 2384539 | 1,78E-04 | *-* | *-* | - | Aggressive behavior | Pappa et al., 2016 |
| rs959381 | 9 | 2384785 | 1,39E-04 | *-* | *-* | - | Aggressive behavior | Pappa et al., 2016 |
| rs959380 | 9 | 2384850 | 1,37E-04 | *-* | *-* | - | Aggressive behavior | Pappa et al., 2016 |
| rs959379 | 9 | 2384917 | 1,19E-04 | *-* | *-* | - | Aggressive behavior | Pappa et al., 2016 |
| rs959378 | 9 | 2385191 | 1,28E-04 | *-* | *-* | - | Aggressive behavior | Pappa et al., 2016 |
| rs1331835 | 9 | 2385837 | 1,34E-04 | *-* | *-* | - | Aggressive behavior | Pappa et al., 2016 |
| rs1412184 | 9 | 2385968 | 1,01E-04 | *-* | *-* | - | Aggressive behavior | Pappa et al., 2016 |
| rs1412185 | 9 | 2386346 | 1,10E-04 | *-* | *-* | - | Aggressive behavior | Pappa et al., 2016 |
| rs10757457 | 9 | 2386521 | 1,09E-04 | *-* | *-* | - | Aggressive behavior | Pappa et al., 2016 |
| rs7028293 | 9 | 2386546 | 1,14E-04 | *-* | *-* | - | Aggressive behavior | Pappa et al., 2016 |
| rs7025116 | 9 | 2386673 | 1,11E-04 | *-* | *-* | - | Aggressive behavior | Pappa et al., 2016 |
| rs10966115 | 9 | 2386732 | 1,32E-04 | *-* | *-* | - | Aggressive behavior | Pappa et al., 2016 |
| rs11791024 | 9 | 2387050 | 1,16E-04 | *-* | *-* | - | Aggressive behavior | Pappa et al., 2016 |
| rs3901487 | 9 | 2387206 | 1,17E-04 | *-* | *-* | - | Aggressive behavior | Pappa et al., 2016 |
| rs4452847 | 9 | 2387354 | 1,17E-04 | *-* | *-* | - | Aggressive behavior | Pappa et al., 2016 |
| rs2104858 | 9 | 2390622 | 1,19E-04 | *-* | *-* | - | Aggressive behavior | Pappa et al., 2016 |
| rs10811996 | 9 | 2392438 | 1,32E-04 | *-* | *-* | - | Aggressive behavior | Pappa et al., 2016 |
| rs10966137 | 9 | 2392576 | 1,34E-04 | *-* | *-* | - | Aggressive behavior | Pappa et al., 2016 |
| rs1412188 | 9 | 2393730 | 1,62E-04 | *-* | *-* | - | Aggressive behavior | Pappa et al., 2016 |
| rs866991 | 9 | 2393909 | 1,92E-04 | *-* | *-* | - | Aggressive behavior | Pappa et al., 2016 |
| rs4741699 | 9 | 2394459 | 1,79E-04 | *-* | *-* | - | Aggressive behavior | Pappa et al., 2016 |
| rs4741700 | 9 | 2394563 | 1,75E-04 | *-* | *-* | - | Aggressive behavior | Pappa et al., 2016 |
| rs4741701 | 9 | 2394608 | 1,72E-04 | *-* | *-* | - | Aggressive behavior | Pappa et al., 2016 |
| rs4741703 | 9 | 2396139 | 2,67E-04 | *-* | *-* | - | Aggressive behavior | Pappa et al., 2016 |
| rs7019582 | 9 | 2397930 | 3,85E-04 | *-* | *-* | - | Aggressive behavior | Pappa et al., 2016 |
| rs7023637 | 9 | 2398792 | 2,30E-04 | *-* | *-* | - | Aggressive behavior | Pappa et al., 2016 |
| rs7038869 | 9 | 2398944 | 2,90E-04 | *-* | *-* | - | Aggressive behavior | Pappa et al., 2016 |
| rs872039 | 9 | 2409538 | 3,42E-04 | *-* | *-* | - | Aggressive behavior | Pappa et al., 2016 |
| rs1323398 | 9 | 6977663 | 3.8E-05 | - | *JMJD2C* | intragenic | CBCL-DP | Mick et al., 2011 |
| rs111862078 | 9 | 7327253 | 3,19E-05 | - | *-* | - | Aggressive behavior | Brevik et al., 2016 |
| rs1609801 | 9 | 13964667 | 3,29E-04 | *-* | *-* | - | Aggressive behavior | Pappa et al., 2016 |
| rs7036966 | 9 | 15367391 | 4,07E-04 | *-* | *SNAPC3* | 45,4 | Aggressive behavior | Pappa et al., 2016 |
| rs2804982 | 9 | 15368449 | 3,89E-04 | *-* | *SNAPC3* | 44,3 | Aggressive behavior | Pappa et al., 2016 |
| rs6474915 | 9 | 15405461 | 4,05E-04 | *-* | *SNAPC3* | 7,3 | Aggressive behavior | Pappa et al., 2016 |
| rs10756697 | 9 | 15718517 | 4,61E-04 | *-* | *C9orf93* | intragenic | Aggressive behavior | Pappa et al., 2016 |
| rs1341737 | 9 | 15798216 | 3,41E-04 | *-* | *C9orf93* | intragenic | Aggressive behavior | Pappa et al., 2016 |
| rs1341738 | 9 | 15798249 | 3,27E-04 | *-* | *C9orf93* | intragenic | Aggressive behavior | Pappa et al., 2016 |
| rs13296360 | 9 | 15800787 | 3,44E-04 | *-* | *C9orf93* | intragenic | Aggressive behavior | Pappa et al., 2016 |
| rs4146293 | 9 | 15801562 | 3,44E-04 | *-* | *C9orf93* | intragenic | Aggressive behavior | Pappa et al., 2016 |
| rs7849380 | 9 | 15801980 | 3,47E-04 | *-* | *C9orf93* | intragenic | Aggressive behavior | Pappa et al., 2016 |
| rs7866641 | 9 | 15802396 | 3,81E-04 | *-* | *C9orf93* | intragenic | Aggressive behavior | Pappa et al., 2016 |
| rs10962170 | 9 | 15802665 | 4,56E-04 | *-* | *C9orf93* | intragenic | Aggressive behavior | Pappa et al., 2016 |
| rs2382553 | 9 | 15804455 | 4,77E-04 | *-* | *C9orf93* | intragenic | Aggressive behavior | Pappa et al., 2016 |
| rs9406537 | 9 | 15805246 | 4,86E-04 | *-* | *C9orf93* | intragenic | Aggressive behavior | Pappa et al., 2016 |
| rs7019205 | 9 | 15813667 | 3,22E-04 | *-* | *C9orf93* | intragenic | Aggressive behavior | Pappa et al., 2016 |
| rs2891007 | 9 | 15816335 | 1,26E-04 | *-* | *C9orf93* | intragenic | Aggressive behavior | Pappa et al., 2016 |
| rs9407649 | 9 | 15816603 | 3,40E-04 | *-* | *C9orf93* | intragenic | Aggressive behavior | Pappa et al., 2016 |
| rs2185665 | 9 | 15818641 | 4,76E-04 | *-* | *C9orf93* | intragenic | Aggressive behavior | Pappa et al., 2016 |
| rs4741545 | 9 | 15820506 | 1,63E-04 | *-* | *C9orf93* | intragenic | Aggressive behavior | Pappa et al., 2016 |
| rs9298739 | 9 | 15823655 | 3,93E-04 | *-* | *C9orf93* | intragenic | Aggressive behavior | Pappa et al., 2016 |
| rs10756709 | 9 | 15829516 | 2,63E-04 | *-* | *C9orf93* | intragenic | Aggressive behavior | Pappa et al., 2016 |
| rs10756720 | 9 | 15909177 | 1,41E-04 | *-* | *C9orf93* | intragenic | Aggressive behavior | Pappa et al., 2016 |
| rs961117 | 9 | 15928548 | 3,45E-04 | *-* | *C9orf93* | intragenic | Aggressive behavior | Pappa et al., 2016 |
| rs10810485 | 9 | 15931175 | 3,48E-04 | *-* | *C9orf93* | intragenic | Aggressive behavior | Pappa et al., 2016 |
| rs4472620 | 9 | 15931439 | 3,46E-04 | *-* | *C9orf93* | intragenic | Aggressive behavior | Pappa et al., 2016 |
| rs1410444 | 9 | 15932632 | 3,46E-04 | *-* | *C9orf93* | intragenic | Aggressive behavior | Pappa et al., 2016 |
| rs10810506 | 9 | 15945190 | 4,77E-04 | *-* | *C9orf93* | intragenic | Aggressive behavior | Pappa et al., 2016 |
| rs10810509 | 9 | 15945666 | 4,16E-04 | *-* | *C9orf93* | intragenic | Aggressive behavior | Pappa et al., 2016 |
| rs9969791 | 9 | 15946164 | 4,58E-04 | *-* | *C9orf93* | intragenic | Aggressive behavior | Pappa et al., 2016 |
| rs10738419 | 9 | 15946824 | 4,11E-04 | *-* | *C9orf93* | intragenic | Aggressive behavior | Pappa et al., 2016 |
| rs10756731 | 9 | 15965600 | 3,90E-04 | *-* | *C9orf93* | 3,7 | Aggressive behavior | Pappa et al., 2016 |
| rs10733306 | 9 | 16038248 | 2,88E-04 | *-* | *C9orf93* | 76,4 | Aggressive behavior | Pappa et al., 2016 |
| rs10810528 | 9 | 16038844 | 1,92E-04 | *-* | *C9orf93* | 76,9 | Aggressive behavior | Pappa et al., 2016 |
| rs10756742 | 9 | 16039260 | 1,42E-04 | *-* | *C9orf93* | 77,4 | Aggressive behavior | Pappa et al., 2016 |
| rs10733307 | 9 | 16041490 | 2,07E-04 | *-* | *C9orf93* | 79,6 | Aggressive behavior | Pappa et al., 2016 |
| rs10738430 | 9 | 16041759 | 2,27E-04 | *-* | *C9orf93* | 79,9 | Aggressive behavior | Pappa et al., 2016 |
| rs2149233 | 9 | 16043485 | 2,02E-04 | *-* | *C9orf93* | 81,6 | Aggressive behavior | Pappa et al., 2016 |
| rs1014307 | 9 | 16043793 | 2,12E-04 | *-* | *C9orf93* | 81,9 | Aggressive behavior | Pappa et al., 2016 |
| rs10963852 | 9 | 18933550 | 4,05E-05 | *-* | *FAM154A* | intragenic | Aggressive behavior | Pappa et al., 2016 |
| rs72703966 | 9 | 20884409 | 2,38E-05 | - | *KIAA1797* | intragenic | Aggressive behavior | Brevik et al., 2016 |
| rs72703970 | 9 | 20894628 | 4,02E-05 | - | *KIAA1797* | intragenic | Aggressive behavior | Brevik et al., 2016 |
| rs72703975 | 9 | 20911798 | 4,28E-05 | - | *KIAA1797* | intragenic | Aggressive behavior | Brevik et al., 2016 |
| rs7861639 | 9 | 24840192 | 1.7E-05 | - | *-* | - | CBCL-DP | Mick et al., 2011 |
| rs10812110 | 9 | 24843876 | 2.2E-05 | - | *-* | - | CBCL-DP | Mick et al., 2011 |
| rs17701236 | 9 | 25292926 | 3,09E-05 | - | *-* | - | Aggressive behavior | Brevik et al., 2016 |
| rs1058326 | 9 | 27437089 | 1,68E-04 | *-* | *MOBKL2B* | intragenic | Aggressive behavior | Pappa et al., 2016 |
| rs1999285 | 9 | 27442817 | 2,95E-04 | *-* | *MOBKL2B* | intragenic | Aggressive behavior | Pappa et al., 2016 |
| rs10970318 | 9 | 31371619 | 5.9E-06 | - | *-* | - | Anger | Merjonen et al., 2011 |
| rs471951 | 9 | 34773040 | 4,45E-04 | *-* | *LOC100129533* | 47,9 | Aggressive behavior | Pappa et al., 2016 |
| rs277603 | 9 | 34776380 | 4,51E-04 | *-* | *LOC100129533* | 44,5 | Aggressive behavior | Pappa et al., 2016 |
| rs277602 | 9 | 34776673 | 4,47E-04 | *-* | *LOC100129533* | 44,2 | Aggressive behavior | Pappa et al., 2016 |
| rs12684304 | 9 | 34989779 | 4,38E-04 | *-* | *DNAJB5* | 1,4 | Aggressive behavior | Pappa et al., 2016 |
| rs10972300 | 9 | 35058201 | 4,40E-04 | *-* | *VCP* | intragenic | Aggressive behavior | Pappa et al., 2016 |
| rs2299612 | 9 | 35060726 | 4,78E-04 | *-* | *VCP* | intragenic | Aggressive behavior | Pappa et al., 2016 |
| rs11139792 | 9 | 71483331 | 4,61E-05 | - | *APBA1* | 6,3 | Aggressive behavior | Brevik et al., 2016 |
| rs7027513 | 9 | 77646126 | 3,99E-04 | *-* | *PCSK5* | 49,3 | Aggressive behavior | Pappa et al., 2016 |
| rs7031123 | 9 | 77646521 | 4,11E-04 | *-* | *PCSK5* | 48,9 | Aggressive behavior | Pappa et al., 2016 |
| rs1831037 | 9 | 77648755 | 1,91E-04 | *-* | *PCSK5* | 46,7 | Aggressive behavior | Pappa et al., 2016 |
| rs10869660 | 9 | 77652437 | 4,06E-04 | *-* | *PCSK5* | 43,0 | Aggressive behavior | Pappa et al., 2016 |
| rs11138445 | 9 | 81799582 | 2,62E-05 | - | *-* | - | ODD | Aebi et al., 2016 |
| rs11141978 | 9 | 89624121 | 3.4E-05 | - | *CTSL3* | 32.5 | Antisocial behavior | Tielbeek et al., 2012 |
| rs11141984 | 9 | 89640666 | 1.7E-05 | - | *C9orf79* | 46.9 | Antisocial behavior | Tielbeek et al., 2012 |
| rs9314779 | 9 | 89642215 | 1.7E-05 | - | *C9orf79* | 45.4 | Antisocial behavior | Tielbeek et al., 2012 |
| rs7028884 | 9 | 89649205 | 1.7E-05 | - | *C9orf79* | 38.4 | Antisocial behavior | Tielbeek et al., 2012 |
| rs10821391 | 9 | 92201540 | 2,26E-05 | - | *-* | - | ODD | Aebi et al., 2016 |
| rs4744373 | 9 | 92203523 | 2,26E-05 | - | *-* | - | ODD | Aebi et al., 2016 |
| rs883922 | 9 | 92221244 | 3,37E-05 | - | *-* | - | ODD | Aebi et al., 2016 |
| rs4237224 | 9 | 92222892 | 3,37E-05 | - | *-* | - | ODD | Aebi et al., 2016 |
| rs4744418 | 9 | 92223224 | 3,37E-05 | - | *-* | - | ODD | Aebi et al., 2016 |
| rs10821418 | 9 | 92223719 | 3,37E-05 | - | *-* | - | ODD | Aebi et al., 2016 |
| rs10821420 | 9 | 92224104 | 2,07E-05 | - | *-* | - | ODD | Aebi et al., 2016 |
| rs10821422 | 9 | 92224502 | 2,07E-05 | - | *-* | - | ODD | Aebi et al., 2016 |
| rs10821423 | 9 | 92225167 | 2,07E-05 | - | *-* | - | ODD | Aebi et al., 2016 |
| rs10821426 | 9 | 92225627 | 2,24E-05 | - | *-* | - | ODD | Aebi et al., 2016 |
| rs10821427 | 9 | 92225760 | 4,30E-05 | - | *-* | - | ODD | Aebi et al., 2016 |
| rs10821428 | 9 | 92227628 | 9,70E-06 | - | *-* | - | ODD | Aebi et al., 2016 |
| rs12002147 | 9 | 99837875 | 2.4E-05 | - | *ANP32B* | 19.8 | Anger | Merjonen et al., 2011 |
| rs1518392 | 9 | 108872566 | 3,19E-05 | - | *ZNF462* | 59,0 | Aggressive behavior | Brevik et al., 2016 |
| rs56084669 | 9 | 108876223 | 4,07E-05 | - | *ZNF462* | 62,6 | Aggressive behavior | Brevik et al., 2016 |
| rs112514183 | 9 | 108877814 | 3,84E-05 | - | *ZNF462* | 64,2 | Aggressive behavior | Brevik et al., 2016 |
| rs56290016 | 9 | 108878196 | 3,81E-05 | - | *ZNF462* | 64,6 | Aggressive behavior | Brevik et al., 2016 |
| rs7040651 | 9 | 108878231 | 3,80E-05 | - | *ZNF462* | 64,6 | Aggressive behavior | Brevik et al., 2016 |
| rs56322036 | 9 | 108878550 | 3,77E-05 | - | *ZNF462* | 65,0 | Aggressive behavior | Brevik et al., 2016 |
| rs72745608 | 9 | 108879515 | 3,73E-05 | - | *ZNF462* | 65,9 | Aggressive behavior | Brevik et al., 2016 |
| rs72745609 | 9 | 108879954 | 3,75E-05 | - | *ZNF462* | 66,4 | Aggressive behavior | Brevik et al., 2016 |
| rs72745610 | 9 | 108880140 | 3,75E-05 | - | *ZNF462* | 66,5 | Aggressive behavior | Brevik et al., 2016 |
| rs72745611 | 9 | 108880538 | 3,74E-05 | - | *ZNF462* | 66,9 | Aggressive behavior | Brevik et al., 2016 |
| rs72745612 | 9 | 108880565 | 3,74E-05 | - | *ZNF462* | 67,0 | Aggressive behavior | Brevik et al., 2016 |
| rs72745614 | 9 | 108880700 | 3,69E-05 | - | *ZNF462* | 67,1 | Aggressive behavior | Brevik et al., 2016 |
| rs72745619 | 9 | 108882140 | 3,19E-05 | - | *ZNF462* | 68,5 | Aggressive behavior | Brevik et al., 2016 |
| rs67679495 | 9 | 108883105 | 3,22E-05 | - | *ZNF462* | 69,5 | Aggressive behavior | Brevik et al., 2016 |
| rs2030235 | 9 | 108883164 | 3,78E-05 | - | *ZNF462* | 69,6 | Aggressive behavior | Brevik et al., 2016 |
| rs4443716 | 9 | 108883423 | 3,79E-05 | - | *ZNF462* | 69,8 | Aggressive behavior | Brevik et al., 2016 |
| rs2056258 | 9 | 108884105 | 3,25E-05 | - | *ZNF462* | 70,5 | Aggressive behavior | Brevik et al., 2016 |
| rs2056257 | 9 | 108884121 | 3,25E-05 | - | *ZNF462* | 70,5 | Aggressive behavior | Brevik et al., 2016 |
| rs4351470 | 9 | 108885372 | 3,28E-05 | - | *ZNF462* | 71,8 | Aggressive behavior | Brevik et al., 2016 |
| rs112797455 | 9 | 108885844 | 3,30E-05 | - | *ZNF462* | 72,2 | Aggressive behavior | Brevik et al., 2016 |
| rs72745627 | 9 | 108886730 | 3,33E-05 | - | *ZNF462* | 73,1 | Aggressive behavior | Brevik et al., 2016 |
| rs72745629 | 9 | 108886762 | 3,32E-05 | - | *ZNF462* | 73,2 | Aggressive behavior | Brevik et al., 2016 |
| rs7861402 | 9 | 108888630 | 3,38E-05 | - | *ZNF462* | 75,0 | Aggressive behavior | Brevik et al., 2016 |
| rs7861530 | 9 | 108888735 | 2,57E-05 | - | *ZNF462* | 75,1 | Aggressive behavior | Brevik et al., 2016 |
| rs7038796 | 9 | 108890022 | 3,38E-05 | - | *ZNF462* | 76,4 | Aggressive behavior | Brevik et al., 2016 |
| rs72745638 | 9 | 108890418 | 3,38E-05 | - | *ZNF462* | 76,8 | Aggressive behavior | Brevik et al., 2016 |
| rs72745640 | 9 | 108890626 | 3,38E-05 | - | *ZNF462* | 77,0 | Aggressive behavior | Brevik et al., 2016 |
| rs72745702 | 9 | 108893088 | 3,39E-05 | - | *ZNF462* | 79,5 | Aggressive behavior | Brevik et al., 2016 |
| rs76748521 | 9 | 108893437 | 3,38E-05 | - | *ZNF462* | 79,8 | Aggressive behavior | Brevik et al., 2016 |
| rs72747507 | 9 | 108896193 | 3,39E-05 | - | *ZNF462* | 82,6 | Aggressive behavior | Brevik et al., 2016 |
| rs16912204 | 9 | 108898000 | 3,37E-05 | - | *ZNF462* | 84,4 | Aggressive behavior | Brevik et al., 2016 |
| rs55900123 | 9 | 108898507 | 3,37E-05 | - | *ZNF462* | 84,9 | Aggressive behavior | Brevik et al., 2016 |
| rs72747514 | 9 | 108900544 | 3,37E-05 | - | *ZNF462* | 86,9 | Aggressive behavior | Brevik et al., 2016 |
| rs56198639 | 9 | 108902535 | 3,38E-05 | - | *ZNF462* | 88,9 | Aggressive behavior | Brevik et al., 2016 |
| rs16912206 | 9 | 108902805 | 3,38E-05 | - | *ZNF462* | 89,2 | Aggressive behavior | Brevik et al., 2016 |
| rs55686268 | 9 | 108903041 | 3,38E-05 | - | *ZNF462* | 89,4 | Aggressive behavior | Brevik et al., 2016 |
| rs16912209 | 9 | 108904852 | 3,40E-05 | - | *ZNF462* | 91,3 | Aggressive behavior | Brevik et al., 2016 |
| rs72747518 | 9 | 108905034 | 3,40E-05 | - | *ZNF462* | 91,4 | Aggressive behavior | Brevik et al., 2016 |
| rs111656836 | 9 | 108905416 | 3,41E-05 | - | *ZNF462* | 91,8 | Aggressive behavior | Brevik et al., 2016 |
| rs7030936 | 9 | 108905657 | 3,42E-05 | - | *ZNF462* | 92,1 | Aggressive behavior | Brevik et al., 2016 |
| rs72747520 | 9 | 108906854 | 3,46E-05 | - | *ZNF462* | 93,3 | Aggressive behavior | Brevik et al., 2016 |
| rs72747521 | 9 | 108907571 | 3,48E-05 | - | *ZNF462* | 94,0 | Aggressive behavior | Brevik et al., 2016 |
| rs55778444 | 9 | 108907886 | 3,87E-05 | - | *ZNF462* | 94,3 | Aggressive behavior | Brevik et al., 2016 |
| rs72747530 | 9 | 108911482 | 3,91E-05 | - | *ZNF462* | 97,9 | Aggressive behavior | Brevik et al., 2016 |
| rs78254720 | 9 | 108915666 | 3,49E-05 | - | *-* | - | Aggressive behavior | Brevik et al., 2016 |
| rs55802380 | 9 | 108925840 | 3,01E-05 | - | *-* | - | Aggressive behavior | Brevik et al., 2016 |
| rs4979590 | 9 | 117687387 | 4,80E-04 | *-* | *-* | - | Aggressive behavior | Pappa et al., 2016 |
| rs914392 | 9 | 137034230 | 3,19E-04 | *-* | *OLFM1* | 72,7 | Aggressive behavior | Pappa et al., 2016 |
| rs11103849 | 9 | 137407391 | 1.8E-05 | - | *-* | - | CBCL-DP | Mick et al., 2011 |
| rs10858396 | 9 | 137415597 | 6.0E-06 | - | *KIAA0649* | 95.9 | CBCL-DP | Mick et al., 2011 |
| rs10508203 | 10 | 1109203 | 2.7E-05 | - | *WDR37* | intragenic | CD | Anney et al., 2008 |
| rs2764980 | 10 | 3274007 | 8.9E-06 | - | *PITRM1* | 69.0 | CD | Anney et al., 2008 |
| rs7095431 | 10 | 8602354 | 6,47E-05 | *-* | *-* | - | Aggressive behavior | Pappa et al., 2016 |
| rs12249942 | 10 | 14530175 | 2.2E-05 | - | *FAM107B* | 70.4 | Anger | Merjonen et al., 2011 |
| rs10508552 | 10 | 18006567 | 1,73E-06 | *-* | *MRC1* | 13,4 | Aggressive behavior | Pappa et al., 2016 |
| rs11014049 | 10 | 18726458 | 4.8E-05 | - | *CACNB2* | intragenic | CBCL-DP | Mick et al., 2011 |
| rs11014484 | 10 | 18819330 | 2,16E-04 | *-* | *CACNB2* | intragenic | Aggressive behavior | Pappa et al., 2016 |
| rs7098949 | 10 | 19042556 | 5,47E-05 | *-* | *ARL5B* | 35,6 | Aggressive behavior | Pappa et al., 2016 |
| rs10734067 | 10 | 19063666 | 4,99E-04 | *-* | *ARL5B* | 56,7 | Aggressive behavior | Pappa et al., 2016 |
| rs2884402 | 10 | 19078020 | 4,06E-04 | *-* | *ARL5B* | 71,1 | Aggressive behavior | Pappa et al., 2016 |
| rs4748516 | 10 | 19083641 | 3,87E-04 | *-* | *ARL5B* | 76,7 | Aggressive behavior | Pappa et al., 2016 |
| rs1854469 | 10 | 19087407 | 3,88E-04 | *-* | *ARL5B* | 80,5 | Aggressive behavior | Pappa et al., 2016 |
| rs1329219 | 10 | 19090437 | 3,89E-04 | *-* | *ARL5B* | 83,5 | Aggressive behavior | Pappa et al., 2016 |
| rs2094647 | 10 | 19093009 | 4,27E-04 | *-* | *ARL5B* | 86,1 | Aggressive behavior | Pappa et al., 2016 |
| rs1490192 | 10 | 19093996 | 4,26E-04 | *-* | *ARL5B* | 87,1 | Aggressive behavior | Pappa et al., 2016 |
| rs75189967 | 10 | 20965737 | 3,95E-05 | - | *-* | - | Aggressive behavior | Brevik et al., 2016 |
| rs117063229 | 10 | 28795268 | 7,49E-06 | - | *WAC* | 66,4 | Aggressive behavior | Brevik et al., 2016 |
| rs7894571 | 10 | 29249956 | 7,86E-06 | - | *-* | - | Aggressive behavior | Brevik et al., 2016 |
| rs7899136 | 10 | 29251328 | 4,59E-06 | - | *-* | - | Aggressive behavior | Brevik et al., 2016 |
| rs4587639 | 10 | 29253180 | 5,43E-06 | - | *-* | - | Aggressive behavior | Brevik et al., 2016 |
| rs17760150 | 10 | 29253850 | 5,83E-06 | - | *-* | - | Aggressive behavior | Brevik et al., 2016 |
| rs12411424 | 10 | 29254350 | 5,85E-06 | - | *-* | - | Aggressive behavior | Brevik et al., 2016 |
| rs2368966 | 10 | 29254615 | 6,52E-06 | - | *-* | - | Aggressive behavior | Brevik et al., 2016 |
| rs2887379 | 10 | 29254737 | 5,89E-06 | - | *-* | - | Aggressive behavior | Brevik et al., 2016 |
| rs7092115 | 10 | 29260313 | 5,69E-06 | - | *-* | - | Aggressive behavior | Brevik et al., 2016 |
| rs10508740 | 10 | 29260345 | 5,70E-06 | - | *-* | - | Aggressive behavior | Brevik et al., 2016 |
| rs10826542 | 10 | 29260425 | 5,73E-06 | - | *-* | - | Aggressive behavior | Brevik et al., 2016 |
| rs10826543 | 10 | 29260506 | 5,69E-06 | - | *-* | - | Aggressive behavior | Brevik et al., 2016 |
| rs10740790 | 10 | 29260891 | 4,84E-06 | - | *-* | - | Aggressive behavior | Brevik et al., 2016 |
| rs10763677 | 10 | 29260981 | 6,39E-06 | - | *-* | - | Aggressive behavior | Brevik et al., 2016 |
| rs1970507 | 10 | 29263011 | 1,01E-05 | - | *-* | - | Aggressive behavior | Brevik et al., 2016 |
| rs4367855 | 10 | 29263318 | 1,01E-05 | - | *-* | - | Aggressive behavior | Brevik et al., 2016 |
| rs10826544 | 10 | 29274967 | 7,99E-06 | - | *-* | - | Aggressive behavior | Brevik et al., 2016 |
| rs10826545 | 10 | 29276401 | 5,51E-06 | - | *-* | - | Aggressive behavior | Brevik et al., 2016 |
| rs10826546 | 10 | 29277468 | 5,15E-06 | - | *-* | - | Aggressive behavior | Brevik et al., 2016 |
| rs7903633 | 10 | 29279955 | 4,96E-06 | - | *-* | - | Aggressive behavior | Brevik et al., 2016 |
| rs117352156 | 10 | 29286211 | 5,41E-06 | - | *-* | - | Aggressive behavior | Brevik et al., 2016 |
| rs188370812 | 10 | 29286255 | 1,75E-06 | - | *-* | - | Aggressive behavior | Brevik et al., 2016 |
| rs192649950 | 10 | 29286256 | 1,97E-06 | - | *-* | - | Aggressive behavior | Brevik et al., 2016 |
| rs10826548 | 10 | 29295600 | 1,07E-06 | - | *-* | - | Aggressive behavior | Brevik et al., 2016 |
| rs12245995 | 10 | 32027325 | 1,02E-04 | *-* | *-* | - | Aggressive behavior | Pappa et al., 2016 |
| rs2311846 | 10 | 44865947 | 1.2E-03 | 2.6E-03 | *ZNF22* | 45.2 | AB+/CU+ | Viding et al., 2010 |
| rs140265971 | 10 | 58981909 | 6,84E-06 | - | *-* | - | Aggressive behavior | Brevik et al., 2016 |
| rs72796654 | 10 | 59025950 | 6,30E-06 | - | *-* | - | Aggressive behavior | Brevik et al., 2016 |
| rs34674354 | 10 | 59037402 | 6,73E-06 | - | *-* | - | Aggressive behavior | Brevik et al., 2016 |
| rs11815470 | 10 | 59040105 | 9,58E-06 | - | *-* | - | Aggressive behavior | Brevik et al., 2016 |
| rs7894146 | 10 | 59041327 | 4,99E-05 | - | *-* | - | Aggressive behavior | Brevik et al., 2016 |
| rs7073050 | 10 | 59051534 | 2,33E-05 | - | *-* | - | Aggressive behavior | Brevik et al., 2016 |
| rs10826093 | 10 | 59059597 | 2,49E-05 | - | *-* | - | Aggressive behavior | Brevik et al., 2016 |
| rs11005881 | 10 | 59065463 | 3,13E-05 | - | *-* | - | Aggressive behavior | Brevik et al., 2016 |
| rs7920252 | 10 | 59069725 | 3,09E-05 | - | *-* | - | Aggressive behavior | Brevik et al., 2016 |
| rs7904303 | 10 | 59070015 | 3,05E-05 | - | *-* | - | Aggressive behavior | Brevik et al., 2016 |
| rs12253686 | 10 | 59070481 | 3,04E-05 | - | *-* | - | Aggressive behavior | Brevik et al., 2016 |
| rs10159907 | 10 | 59072731 | 3,43E-05 | - | *-* | - | Aggressive behavior | Brevik et al., 2016 |
| rs10159565 | 10 | 59072781 | 3,38E-05 | - | *-* | - | Aggressive behavior | Brevik et al., 2016 |
| rs4387281 | 10 | 59085442 | 9,91E-06 | - | *-* | - | Aggressive behavior | Brevik et al., 2016 |
| rs72799990 | 10 | 59086124 | 2,54E-05 | - | *-* | - | Aggressive behavior | Brevik et al., 2016 |
| rs11005885 | 10 | 59086810 | 9,81E-06 | - | *-* | - | Aggressive behavior | Brevik et al., 2016 |
| rs11818693 | 10 | 59088993 | 1,01E-05 | - | *-* | - | Aggressive behavior | Brevik et al., 2016 |
| rs10128443 | 10 | 59092433 | 7,72E-06 | - | *-* | - | Aggressive behavior | Brevik et al., 2016 |
| rs10822277 | 10 | 65495321 | 3.5E-05 | - | *-* | - | Antisocial behavior | Tielbeek et al., 2012 |
| rs3886687 | 10 | 71271770 | 3,53E-04 | *-* | *COL13A1* | intragenic | Aggressive behavior | Pappa et al., 2016 |
| rs2763345 | 10 | 71273522 | 4,55E-04 | *-* | *COL13A1* | intragenic | Aggressive behavior | Pappa et al., 2016 |
| rs1227779 | 10 | 71357217 | 7,72E-05 | *-* | *COL13A1* | intragenic | Aggressive behavior | Pappa et al., 2016 |
| rs1227780 | 10 | 71357372 | 8,23E-05 | *-* | *COL13A1* | intragenic | Aggressive behavior | Pappa et al., 2016 |
| rs3793819 | 10 | 71371570 | 1,92E-04 | *-* | *COL13A1* | intragenic | Aggressive behavior | Pappa et al., 2016 |
| rs3843585 | 10 | 71374757 | 4,87E-05 | *-* | *COL13A1* | intragenic | Aggressive behavior | Pappa et al., 2016 |
| rs3793815 | 10 | 71377693 | 1,14E-04 | *-* | *COL13A1* | intragenic | Aggressive behavior | Pappa et al., 2016 |
| rs4746013 | 10 | 71380148 | 2,94E-04 | *-* | *COL13A1* | intragenic | Aggressive behavior | Pappa et al., 2016 |
| rs10762324 | 10 | 71380268 | 2,09E-04 | *-* | *COL13A1* | intragenic | Aggressive behavior | Pappa et al., 2016 |
| rs3793814 | 10 | 71380589 | 1,88E-04 | *-* | *COL13A1* | intragenic | Aggressive behavior | Pappa et al., 2016 |
| rs1356091 | 10 | 79036677 | 4,24E-04 | *-* | *KCNMA1* | intragenic | Aggressive behavior | Pappa et al., 2016 |
| rs2670149 | 10 | 79044761 | 3,73E-04 | *-* | *KCNMA1* | intragenic | Aggressive behavior | Pappa et al., 2016 |
| rs12761718 | 10 | 79162976 | 5.2E-03 | 4.8E-05 | *DLG5* | 57.6 | AB+/CU+ | Viding et al., 2010 |
| rs7076131 | 10 | 80155611 | 4,77E-04 | *-* | *-* | - | Aggressive behavior | Pappa et al., 2016 |
| rs7898483 | 10 | 80163704 | 5,00E-04 | *-* | *-* | - | Aggressive behavior | Pappa et al., 2016 |
| rs6480904 | 10 | 80163875 | 4,99E-04 | *-* | *-* | - | Aggressive behavior | Pappa et al., 2016 |
| rs7917285 | 10 | 80164188 | 4,71E-04 | *-* | *-* | - | Aggressive behavior | Pappa et al., 2016 |
| rs12411760 | 10 | 83090553 | 3,38E-05 | - | *-* | - | ODD | Aebi et al., 2016 |
| rs9787444 | 10 | 83161089 | 4,41E-05 | - | *-* | - | ODD | Aebi et al., 2016 |
| rs7082300 | 10 | 83168887 | 4,41E-05 | - | *-* | - | ODD | Aebi et al., 2016 |
| rs11189899 | 10 | 83186938 | 3,97E-05 | - | *-* | - | ODD | Aebi et al., 2016 |
| rs11189904 | 10 | 83187383 | 3,97E-05 | - | *-* | - | ODD | Aebi et al., 2016 |
| rs9971207 | 10 | 86417483 | 4.1E-05 | - | *-* | - | Anger (reaction) | Mick et al., 2014 |
| rs11201163 | 10 | 86420715 | 2.8E-05 | - | *-* | - | Anger (reaction) | Mick et al., 2014 |
| rs4933636 | 10 | 92749404 | 4,92E-04 | *-* | *ANKRD1* | 78,4 | Aggressive behavior | Pappa et al., 2016 |
| rs2016612 | 10 | 92753048 | 2,01E-04 | *-* | *ANKRD1* | 82,0 | Aggressive behavior | Pappa et al., 2016 |
| rs7076190 | 10 | 92753745 | 2,52E-04 | *-* | *ANKRD1* | 82,7 | Aggressive behavior | Pappa et al., 2016 |
| rs7096430 | 10 | 92754440 | 2,39E-04 | *-* | *ANKRD1* | 83,4 | Aggressive behavior | Pappa et al., 2016 |
| rs3852490 | 10 | 92771935 | 1,98E-04 | *-* | *-* | - | Aggressive behavior | Pappa et al., 2016 |
| rs9787573 | 10 | 92773493 | 1,11E-04 | *-* | *-* | - | Aggressive behavior | Pappa et al., 2016 |
| rs3843023 | 10 | 92781365 | 1,37E-04 | *-* | *-* | - | Aggressive behavior | Pappa et al., 2016 |
| rs2486687 | 10 | 94768263 | 4.1E-05 | - | *EXOC6* | intragenic | CBCL-DP | Mick et al., 2011 |
| rs1475366 | 10 | 94771837 | 3.2E-05 | - | *EXOC6* | intragenic | CBCL-DP | Mick et al., 2011 |
| rs12416051 | 10 | 94778815 | 4.4E-05 | - | *EXOC6* | intragenic | CBCL-DP | Mick et al., 2011 |
| rs9419763 | 10 | 94778945 | 4.9E-05 | - | *EXOC6* | intragenic | CBCL-DP | Mick et al., 2011 |
| rs7895700 | 10 | 94781079 | 4.4E-05 | - | *EXOC6* | intragenic | CBCL-DP | Mick et al., 2011 |
| rs11187247 | 10 | 94784299 | 1.8E-05 | - | *EXOC6* | intragenic | CBCL-DP | Mick et al., 2011 |
| rs835275 | 10 | 94786663 | 2.9E-05 | - | *EXOC6* | intragenic | CBCL-DP | Mick et al., 2011 |
| rs9419765 | 10 | 94788151 | 1.4E-05 | - | *EXOC6* | intragenic | CBCL-DP | Mick et al., 2011 |
| rs1750741 | 10 | 94794510 | 1.5E-05 | - | *EXOC6* | intragenic | CBCL-DP | Mick et al., 2011 |
| rs835280 | 10 | 94801776 | 2.2E-05 | - | *EXOC6* | intragenic | CBCL-DP | Mick et al., 2011 |
| rs12414054 | 10 | 111324267 | 4.1E-05 | - | *-* | - | Antisocial behavior | Tielbeek et al., 2012 |
| rs1341051 | 10 | 112604200 | 2,78E-04 | *-* | *PDCD4* | 17,4 | Aggressive behavior | Pappa et al., 2016 |
| rs4918607 | 10 | 112610894 | 2,73E-04 | *-* | *PDCD4* | 10,7 | Aggressive behavior | Pappa et al., 2016 |
| rs1341053 | 10 | 112614407 | 4,59E-04 | *-* | *PDCD4* | 7,2 | Aggressive behavior | Pappa et al., 2016 |
| rs11195360 | 10 | 112623908 | 1,08E-04 | *-* | *PDCD4* | intragenic | Aggressive behavior | Pappa et al., 2016 |
| rs1407696 | 10 | 112628018 | 8,19E-05 | *-* | *PDCD4* | intragenic | Aggressive behavior | Pappa et al., 2016 |
| rs10787289 | 10 | 112647197 | 2,53E-04 | *-* | *PDCD4* | intragenic | Aggressive behavior | Pappa et al., 2016 |
| rs7895872 | 10 | 112668647 | 3,80E-04 | *-* | *PDCD4* | 18,9 | Aggressive behavior | Pappa et al., 2016 |
| rs7079511 | 10 | 112676313 | 1,12E-04 | *-* | *PDCD4* | 26,6 | Aggressive behavior | Pappa et al., 2016 |
| rs7915214 | 10 | 112690877 | 2,63E-04 | *-* | *SHOC2* | 23,0 | Aggressive behavior | Pappa et al., 2016 |
| rs4597005 | 10 | 112702467 | 6,89E-05 | *-* | *SHOC2* | 11,4 | Aggressive behavior | Pappa et al., 2016 |
| rs10885068 | 10 | 112703805 | 9,39E-05 | *-* | *SHOC2* | 10,1 | Aggressive behavior | Pappa et al., 2016 |
| rs10885070 | 10 | 112719365 | 9,12E-05 | *-* | *SHOC2* | intragenic | Aggressive behavior | Pappa et al., 2016 |
| rs11195400 | 10 | 112753375 | 7,77E-05 | *-* | *SHOC2* | intragenic | Aggressive behavior | Pappa et al., 2016 |
| rs1327551 | 10 | 112758186 | 7,54E-05 | *-* | *SHOC2* | intragenic | Aggressive behavior | Pappa et al., 2016 |
| rs12245521 | 10 | 118287023 | 3.2E-05 | - | *PNLIP* | 8.4 | Anger (temper) | Mick et al., 2014 |
| rs12249434 | 10 | 118288696 | 1.2E-05 | - | *PNLIP* | 6.7 | Anger (temper) | Mick et al., 2014 |
| rs7921168 | 10 | 118337660 | 2.4E-05 | - | *PNLIPRP1* | 2.8 | Anger (temper) | Mick et al., 2014 |
| rs12357982 | 10 | 122970734 | 1,16E-04 | *-* | *-* | - | Aggressive behavior | Pappa et al., 2016 |
| rs4980180 | 10 | 124877815 | 2,85E-05 | - | *HMX3* | 7,7 | Aggressive behavior | Brevik et al., 2016 |
| rs2935651 | 10 | 126745920 | 2,45E-04 | *-* | *CTBP2* | intragenic | Aggressive behavior | Pappa et al., 2016 |
| rs4021187 | 10 | 126746073 | 2,35E-04 | *-* | *CTBP2* | intragenic | Aggressive behavior | Pappa et al., 2016 |
| rs7087286 | 10 | 126747241 | 2,81E-04 | *-* | *CTBP2* | intragenic | Aggressive behavior | Pappa et al., 2016 |
| rs3012070 | 10 | 126754403 | 3,85E-04 | *-* | *CTBP2* | intragenic | Aggressive behavior | Pappa et al., 2016 |
| rs11245496 | 10 | 126763736 | 8,71E-05 | *-* | *CTBP2* | intragenic | Aggressive behavior | Pappa et al., 2016 |
| rs1278280 | 10 | 127745534 | 1,68E-05 | - | *ADAM12* | intragenic | ODD | Aebi et al., 2016 |
| rs1152655 | 10 | 127749561 | 4,74E-05 | - | *ADAM12* | intragenic | ODD | Aebi et al., 2016 |
| rs985461 | 10 | 127759535 | 2,42E-05 | - | *ADAM12* | intragenic | ODD | Aebi et al., 2016 |
| rs1278352 | 10 | 127763366 | 1,24E-06 | - | *ADAM12* | intragenic | ODD | Aebi et al., 2016 |
| rs12248471 | 10 | 131938087 | 1,77E-04 | *-* | *GLRX3* | 70,2 | Aggressive behavior | Pappa et al., 2016 |
| rs12242542 | 10 | 131942151 | 4,10E-04 | *-* | *GLRX3* | 74,3 | Aggressive behavior | Pappa et al., 2016 |
| rs11017164 | 10 | 131942430 | 4,21E-04 | *-* | *GLRX3* | 74,6 | Aggressive behavior | Pappa et al., 2016 |
| rs11017168 | 10 | 131944840 | 4,69E-04 | *-* | *GLRX3* | 77,0 | Aggressive behavior | Pappa et al., 2016 |
| rs11017169 | 10 | 131945206 | 4,49E-04 | *-* | *GLRX3* | 77,4 | Aggressive behavior | Pappa et al., 2016 |
| rs12260196 | 10 | 131952632 | 3,43E-04 | *-* | *GLRX3* | 84,8 | Aggressive behavior | Pappa et al., 2016 |
| rs11017175 | 10 | 131959278 | 1,07E-04 | *-* | *GLRX3* | 91,4 | Aggressive behavior | Pappa et al., 2016 |
| rs11017177 | 10 | 131960221 | 1,23E-05 | *-* | *GLRX3* | 92,4 | Aggressive behavior | Pappa et al., 2016 |
| rs12252182 | 10 | 131962527 | 1,50E-04 | *-* | *GLRX3* | 94,7 | Aggressive behavior | Pappa et al., 2016 |
| rs12255434 | 10 | 131969078 | 1,33E-05 | *-* | *-* | - | Aggressive behavior | Pappa et al., 2016 |
| rs12265437 | 10 | 131970739 | 8,61E-05 | *-* | *-* | - | Aggressive behavior | Pappa et al., 2016 |
| rs7080464 | 10 | 131974739 | 2,96E-04 | *-* | *-* | - | Aggressive behavior | Pappa et al., 2016 |
| rs9787715 | 10 | 131986709 | 8,44E-05 | *-* | *-* | - | Aggressive behavior | Pappa et al., 2016 |
| rs11017192 | 10 | 131992673 | 8,15E-05 | *-* | *-* | - | Aggressive behavior | Pappa et al., 2016 |
| rs7079367 | 10 | 131997537 | 7,81E-05 | *-* | *-* | - | Aggressive behavior | Pappa et al., 2016 |
| rs12259325 | 10 | 131997992 | 7,66E-05 | *-* | *-* | - | Aggressive behavior | Pappa et al., 2016 |
| rs7918047 | 10 | 132001099 | 8,82E-05 | *-* | *-* | - | Aggressive behavior | Pappa et al., 2016 |
| rs7918061 | 10 | 132001118 | 7,59E-05 | *-* | *-* | - | Aggressive behavior | Pappa et al., 2016 |
| rs12264728 | 10 | 132029564 | 2,26E-04 | *-* | *-* | - | Aggressive behavior | Pappa et al., 2016 |
| rs652911 | 10 | 132029865 | 3,01E-04 | *-* | *-* | - | Aggressive behavior | Pappa et al., 2016 |
| rs366346 | 10 | 132030831 | 2,89E-04 | *-* | *-* | - | Aggressive behavior | Pappa et al., 2016 |
| rs6560704 | 10 | 133648503 | 4.0E-03 | 5.0E-03 | *BNIP3* | 3.1 | AB+/CU+ | Viding et al., 2010 |
| rs12807478 | 11 | 2118531 | 1,41E-05 | - | *IGF2* | intragenic | Aggressive behavior | Brevik et al., 2016 |
| rs7950669 | 11 | 6723648 | 1,77E-05 | - | *OR2AG2* | 22,2 | ODD | Aebi et al., 2016 |
| rs937854 | 11 | 6725977 | 1,77E-05 | - | *OR2AG2* | 19,8 | ODD | Aebi et al., 2016 |
| rs6578777 | 11 | 6731078 | 1,77E-05 | - | *OR2AG2* | 14,7 | ODD | Aebi et al., 2016 |
| rs4366466 | 11 | 6733700 | 1,54E-05 | - | *OR2AG2* | 12,1 | ODD | Aebi et al., 2016 |
| rs4758132 | 11 | 6739977 | 3,15E-06 | - | *OR2AG2* | 5,8 | ODD | Aebi et al., 2016 |
| rs11042078 | 11 | 8783276 | 2,89E-04 | *-* | *ST5* | intragenic | Aggressive behavior | Pappa et al., 2016 |
| rs11603841 | 11 | 8791256 | 3,30E-04 | *-* | *ST5* | intragenic | Aggressive behavior | Pappa et al., 2016 |
| rs2568090 | 11 | 8806071 | 4,40E-04 | *-* | *ST5* | intragenic | Aggressive behavior | Pappa et al., 2016 |
| rs7945705 | 11 | 8819106 | 4,83E-04 | *-* | *ST5* | intragenic | Aggressive behavior | Pappa et al., 2016 |
| rs2742537 | 11 | 8864965 | 3,05E-04 | *-* | *ST5* | intragenic | Aggressive behavior | Pappa et al., 2016 |
| rs11022174 | 11 | 12079782 | 4,74E-06 | - | *MICAL2* | 8,9 | Aggressive behavior | Brevik et al., 2016 |
| rs10831734 | 11 | 12079797 | 4,47E-06 | - | *MICAL2* | 8,9 | Aggressive behavior | Brevik et al., 2016 |
| rs11022522 | 11 | 12850523 | 3,55E-05 | - | *TEAD1* | intragenic | Aggressive behavior | Brevik et al., 2016 |
| rs16924133 | 11 | 33281952 | 2.1E-05 | - | *HIPK3* | intragenic | Anger (temper) | Mick et al., 2014 |
| rs4638283 | 11 | 35456941 | 3,12E-04 | *-* | *DKFZP586H2123* | intragenic | Aggressive behavior | Pappa et al., 2016 |
| rs2953307 | 11 | 40084439 | 3,42E-04 | *-* | *LRRC4C* | 7,9 | Aggressive behavior | Pappa et al., 2016 |
| rs10838266 | 11 | 44324608 | 3,64E-05 | - | *ALX4* | 36,3 | Aggressive behavior | Brevik et al., 2016 |
| rs4755815 | 11 | 44324957 | 4,81E-05 | - | *ALX4* | 36,7 | Aggressive behavior | Brevik et al., 2016 |
| rs12806498 | 11 | 54828758 | 3.0E-05 | - | *TRIM48* | 33.6 | Antisocial behavior | Tielbeek et al., 2012 |
| rs2521568 | 11 | 61457509 | 3,90E-04 | *-* | *RAB3IL1* | 15,9 | Aggressive behavior | Pappa et al., 2016 |
| rs2727266 | 11 | 61460910 | 3,57E-04 | *-* | *BEST1* | 13,5 | Aggressive behavior | Pappa et al., 2016 |
| rs3824854 | 11 | 63641881 | 1.5E-05 | - | *FLRT1, MACROD1* | intragenic | CBCL-DP | Mick et al., 2011 |
| rs7939784 | 11 | 63666729 | 1.1E-05 | - | *MACROD1* | intragenic | CBCL-DP | Mick et al., 2011 |
| rs2845597 | 11 | 63710946 | 4.1E-05 | - | *STIP1* | intragenic | CBCL-DP | Mick et al., 2011 |
| rs2282490 | 11 | 63718728 | 4.0E-06 | - | *STIP1* | intragenic | CBCL-DP | Mick et al., 2011 |
| rs11607165 | 11 | 63720523 | 4.0E-06 | - | *STIP1* | intragenic | CBCL-DP | Mick et al., 2011 |
| rs882146 | 11 | 63733275 | 3.0E-06 | - | *FERMT3* | intragenic | CBCL-DP | Mick et al., 2011 |
| rs12575642 | 11 | 63736219 | 1.0E-06 | - | *FERMT3* | intragenic | CBCL-DP | Mick et al., 2011 |
| rs1059440 | 11 | 63748377 | 4.0E-06 | - | *TRPT1* | intragenic | CBCL-DP | Mick et al., 2011 |
| rs907848 | 11 | 71956950 | 1,74E-04 | *-* | *PDE2A* | 7,9 | Aggressive behavior | Pappa et al., 2016 |
| rs7117518 | 11 | 71957198 | 1,66E-04 | *-* | *PDE2A* | 7,6 | Aggressive behavior | Pappa et al., 2016 |
| rs7122287 | 11 | 76108177 | 3,70E-04 | *-* | *LRRC32* | 48,7 | Aggressive behavior | Pappa et al., 2016 |
| rs1149578 | 11 | 76224619 | 2,35E-04 | *-* | *PHCA* | 25,0 | Aggressive behavior | Pappa et al., 2016 |
| rs1225138 | 11 | 76231135 | 1,76E-04 | *-* | *PHCA* | 18,4 | Aggressive behavior | Pappa et al., 2016 |
| rs1893815 | 11 | 79536511 | 6.2E-05 | 3.6E-03 | *-* | - | AB+/CU+ | Viding et al., 2010 |
| rs11225932 | 11 | 96666068 | 3,20E-04 | *-* | *-* | - | Aggressive behavior | Pappa et al., 2016 |
| rs512078 | 11 | 100237353 | 2,06E-04 | *-* | *-* | - | Aggressive behavior | Pappa et al., 2016 |
| rs17116334 | 11 | 113466972 | 2,77E-05 | - | *ZBTB16* | intragenic | ODD | Aebi et al., 2016 |
| rs10750165 | 11 | 119143298 | 7,38E-06 | - | *PVRL1* | 38,7 | Aggressive behavior | Brevik et al., 2016 |
| rs34547019 | 11 | 130731446 | 2,67E-06 | - | *HNT* | 14,1 | Aggressive behavior | Brevik et al., 2016 |
| rs71483660 | 11 | 130734096 | 1,47E-05 | - | *HNT* | 11,5 | Aggressive behavior | Brevik et al., 2016 |
| rs35042821 | 11 | 130735991 | 3,20E-06 | - | *HNT* | 9,6 | Aggressive behavior | Brevik et al., 2016 |
| rs35665773 | 11 | 130742012 | 3,25E-06 | - | *HNT* | 3,6 | Aggressive behavior | Brevik et al., 2016 |
| rs12804059 | 11 | 130744026 | 3,81E-06 | - | *HNT* | 1,6 | Aggressive behavior | Brevik et al., 2016 |
| rs7119590 | 11 | 130745014 | 5,89E-06 | - | *HNT* | 0,6 | Aggressive behavior | Brevik et al., 2016 |
| rs34588147 | 11 | 130745428 | 3,59E-06 | - | *HNT* | 0,2 | Aggressive behavior | Brevik et al., 2016 |
| rs34807050 | 11 | 130750266 | 1,84E-06 | - | *HNT* | intragenic | Aggressive behavior | Brevik et al., 2016 |
| rs35974940 | 11 | 130750297 | 1,26E-06 | - | *HNT* | intragenic | Aggressive behavior | Brevik et al., 2016 |
| rs35595674 | 11 | 130754684 | 1,77E-05 | - | *HNT* | intragenic | Aggressive behavior | Brevik et al., 2016 |
| rs35385808 | 11 | 130755453 | 5,28E-06 | - | *HNT* | intragenic | Aggressive behavior | Brevik et al., 2016 |
| rs71483662 | 11 | 130764306 | 1,80E-05 | - | *HNT* | intragenic | Aggressive behavior | Brevik et al., 2016 |
| rs66771462 | 11 | 130765735 | 1,59E-05 | - | *HNT* | intragenic | Aggressive behavior | Brevik et al., 2016 |
| rs11064294 | 12 | 675712 | 2.1E-05 | - | *NINJ2* | 32.7 | CBCL-DP | Mick et al., 2011 |
| rs16933834 | 12 | 5718072 | 4,31E-04 | *-* | *TMEM16B* | intragenic | Aggressive behavior | Pappa et al., 2016 |
| rs17785973 | 12 | 5728059 | 4,99E-04 | *-* | *TMEM16B* | intragenic | Aggressive behavior | Pappa et al., 2016 |
| rs4393375 | 12 | 17778102 | 3,64E-05 | - | *-* | - | ODD | Aebi et al., 2016 |
| rs4290283 | 12 | 17778634 | 2,16E-05 | - | *-* | - | ODD | Aebi et al., 2016 |
| rs4073225 | 12 | 17778777 | 2,16E-05 | - | *-* | - | ODD | Aebi et al., 2016 |
| rs7304699 | 12 | 17778929 | 2,38E-05 | - | *-* | - | ODD | Aebi et al., 2016 |
| rs6486834 | 12 | 17779137 | 2,38E-05 | - | *-* | - | ODD | Aebi et al., 2016 |
| rs4323926 | 12 | 17779336 | 2,38E-05 | - | *-* | - | ODD | Aebi et al., 2016 |
| rs6486835 | 12 | 17779531 | 2,38E-05 | - | *-* | - | ODD | Aebi et al., 2016 |
| rs10840804 | 12 | 17779620 | 2,38E-05 | - | *-* | - | ODD | Aebi et al., 2016 |
| rs4432071 | 12 | 17792126 | 2,38E-05 | - | *-* | - | ODD | Aebi et al., 2016 |
| rs10431206 | 12 | 17794950 | 2,38E-05 | - | *-* | - | ODD | Aebi et al., 2016 |
| rs10431208 | 12 | 17795424 | 2,38E-05 | - | *-* | - | ODD | Aebi et al., 2016 |
| rs10431209 | 12 | 17795456 | 2,38E-05 | - | *-* | - | ODD | Aebi et al., 2016 |
| rs10840847 | 12 | 17795891 | 2,38E-05 | - | *-* | - | ODD | Aebi et al., 2016 |
| rs10770251 | 12 | 17796041 | 2,38E-05 | - | *-* | - | ODD | Aebi et al., 2016 |
| rs17399946 | 12 | 23968776 | 6,95E-06 | - | *SOX5* | intragenic | ODD | Aebi et al., 2016 |
| rs7968499 | 12 | 27302033 | 2,86E-04 | *-* | *STK38L* | intragenic | Aggressive behavior | Pappa et al., 2016 |
| rs7358692 | 12 | 27304159 | 2,46E-04 | *-* | *STK38L* | intragenic | Aggressive behavior | Pappa et al., 2016 |
| rs2035124 | 12 | 27304590 | 3,37E-04 | *-* | *STK38L* | intragenic | Aggressive behavior | Pappa et al., 2016 |
| rs1026599 | 12 | 27306629 | 3,11E-04 | *-* | *STK38L* | intragenic | Aggressive behavior | Pappa et al., 2016 |
| rs1963511 | 12 | 27307549 | 3,11E-04 | *-* | *STK38L* | intragenic | Aggressive behavior | Pappa et al., 2016 |
| rs11048955 | 12 | 27314210 | 8,65E-05 | *-* | *STK38L* | intragenic | Aggressive behavior | Pappa et al., 2016 |
| rs10771328 | 12 | 27316633 | 3,79E-04 | *-* | *STK38L* | intragenic | Aggressive behavior | Pappa et al., 2016 |
| rs11048959 | 12 | 27319223 | 1,49E-04 | *-* | *STK38L* | intragenic | Aggressive behavior | Pappa et al., 2016 |
| rs10842896 | 12 | 27326265 | 1,39E-04 | *-* | *STK38L* | intragenic | Aggressive behavior | Pappa et al., 2016 |
| rs10772205 | 12 | 34643868 | 2.6E-05 | - | *-* | - | Anger | Merjonen et al., 2011 |
| rs7484949 | 12 | 41334788 | 1.8E-05 | - | *PRICKLE1* | 65.0 | Antisocial behavior | Tielbeek et al., 2012 |
| rs11170205 | 12 | 51301638 | 3,77E-04 | *-* | *KRT73* | 3,0 | Aggressive behavior | Pappa et al., 2016 |
| rs1453696 | 12 | 60169684 | 1,16E-05 | - | *-* | - | ODD | Aebi et al., 2016 |
| rs10784227 | 12 | 60178960 | 5,77E-06 | - | *-* | - | ODD | Aebi et al., 2016 |
| rs1453714 | 12 | 60187685 | 4,74E-06 | - | *-* | - | ODD | Aebi et al., 2016 |
| rs12370275 | 12 | 60193289 | 2,41E-06 | - | *-* | - | ODD | Aebi et al., 2016 |
| rs11174055 | 12 | 60195641 | 2,41E-06 | - | *-* | - | ODD | Aebi et al., 2016 |
| rs1453708 | 12 | 60200018 | 2,49E-06 | - | *-* | - | ODD | Aebi et al., 2016 |
| rs1495028 | 12 | 61801281 | 3,52E-04 | *-* | *AVPR1A* | 25,2 | Aggressive behavior | Pappa et al., 2016 |
| rs7964655 | 12 | 61814559 | 4,43E-04 | *-* | *AVPR1A* | 11,9 | Aggressive behavior | Pappa et al., 2016 |
| rs7964874 | 12 | 61814689 | 4,67E-04 | *-* | *AVPR1A* | 11,8 | Aggressive behavior | Pappa et al., 2016 |
| rs10877965 | 12 | 61814976 | 4,66E-05 | *-* | *AVPR1A* | 11,5 | Aggressive behavior | Pappa et al., 2016 |
| rs11174803 | 12 | 61815789 | 2,45E-05 | *-* | *AVPR1A* | 10,7 | Aggressive behavior | Pappa et al., 2016 |
| rs1587097 | 12 | 61816512 | 2,25E-04 | *-* | *AVPR1A* | 10,0 | Aggressive behavior | Pappa et al., 2016 |
| rs7972829 | 12 | 61816874 | 2,59E-05 | *-* | *AVPR1A* | 9,6 | Aggressive behavior | Pappa et al., 2016 |
| rs1587098 | 12 | 61818656 | 8,95E-05 | *-* | *AVPR1A* | 7,8 | Aggressive behavior | Pappa et al., 2016 |
| rs11174805 | 12 | 61819374 | 8,99E-05 | *-* | *AVPR1A* | 7,1 | Aggressive behavior | Pappa et al., 2016 |
| rs11174806 | 12 | 61819391 | 1,15E-04 | *-* | *AVPR1A* | 7,1 | Aggressive behavior | Pappa et al., 2016 |
| rs10747983 | 12 | 61824725 | 4,42E-05 | *-* | *AVPR1A* | 1,8 | Aggressive behavior | Pappa et al., 2016 |
| rs10784339 | 12 | 61824913 | 4,99E-05 | *-* | *AVPR1A* | 1,6 | Aggressive behavior | Pappa et al., 2016 |
| rs11174811 | 12 | 61826743 | 2,52E-04 | *-* | *AVPR1A* | intragenic | Aggressive behavior | Pappa et al., 2016 |
| rs3803107 | 12 | 61827101 | 4,14E-05 | *-* | *AVPR1A* | intragenic | Aggressive behavior | Pappa et al., 2016 |
| rs10877968 | 12 | 61829452 | 3,36E-05 | *-* | *AVPR1A* | intragenic | Aggressive behavior | Pappa et al., 2016 |
| rs3021529 | 12 | 61831947 | 3,32E-04 | *-* | *AVPR1A* | intragenic | Aggressive behavior | Pappa et al., 2016 |
| rs7308008 | 12 | 61844229 | 4,69E-04 | *-* | *AVPR1A* | 11,4 | Aggressive behavior | Pappa et al., 2016 |
| rs7962106 | 12 | 61887586 | 1,64E-04 | *-* | *AVPR1A* | 54,7 | Aggressive behavior | Pappa et al., 2016 |
| rs12317376 | 12 | 63727900 | 1.4E-05 | - | *WIF1* | 2.8 | Anger | Merjonen et al., 2011 |
| rs7134682 | 12 | 64454418 | 1,44E-04 | *-* | *HMGA2* | 50,1 | Aggressive behavior | Pappa et al., 2016 |
| rs2917849 | 12 | 65886130 | 4.8E-06 | - | *CAND1* | 63.2 | Antisocial behavior | Tielbeek et al., 2012 |
| rs11176629 | 12 | 65887449 | 4.8E-06 | - | *CAND1* | 61.9 | Antisocial behavior | Tielbeek et al., 2012 |
| rs1657978 | 12 | 65889948 | 4.8E-06 | - | *CAND1* | 59.4 | Antisocial behavior | Tielbeek et al., 2012 |
| rs775313 | 12 | 65898396 | 4.8E-06 | - | *CAND1* | 50.9 | Antisocial behavior | Tielbeek et al., 2012 |
| rs11176925 | 12 | 66568062 | 1,60E-05 | - | *-* | - | ODD | Aebi et al., 2016 |
| rs789560 | 12 | 68618094 | 7.2E-06 | - | *-* | - | CD | Anney et al., 2008 |
| rs17835503 | 12 | 77371908 | 2,33E-05 | - | *-* | - | Aggressive behavior | Brevik et al., 2016 |
| rs10859716 | 12 | 93435384 | 5.1E-03 | 4.1E-03 | *TMCC3* | 49.7 | AB+/CU+ | Viding et al., 2010 |
| rs10860571 | 12 | 99198057 | 4,44E-04 | *-* | *SCYL2* | intragenic | Aggressive behavior | Pappa et al., 2016 |
| rs10860580 | 12 | 99256458 | 3,29E-04 | *-* | *SCYL2* | intragenic | Aggressive behavior | Pappa et al., 2016 |
| rs11110345 | 12 | 99261379 | 3,35E-04 | *-* | *SCYL2* | 3,3 | Aggressive behavior | Pappa et al., 2016 |
| rs3931028 | 12 | 99263745 | 3,08E-04 | *-* | *SCYL2* | 5,7 | Aggressive behavior | Pappa et al., 2016 |
| rs7298461 | 12 | 99267288 | 3,28E-04 | *-* | *SLC17A8* | 7,8 | Aggressive behavior | Pappa et al., 2016 |
| rs4475991 | 12 | 103120886 | 3,00E-04 | *-* | *TXNRD1* | 12,8 | Aggressive behavior | Pappa et al., 2016 |
| rs7314893 | 12 | 103121899 | 3,00E-04 | *-* | *TXNRD1* | 11,8 | Aggressive behavior | Pappa et al., 2016 |
| rs7313063 | 12 | 103126338 | 2,96E-04 | *-* | *TXNRD1* | 7,4 | Aggressive behavior | Pappa et al., 2016 |
| rs10861169 | 12 | 103134770 | 2,85E-04 | *-* | *TXNRD1* | intragenic | Aggressive behavior | Pappa et al., 2016 |
| rs10861171 | 12 | 103141908 | 1,29E-04 | *-* | *TXNRD1* | intragenic | Aggressive behavior | Pappa et al., 2016 |
| rs7975524 | 12 | 103148669 | 4,40E-04 | *-* | *TXNRD1* | intragenic | Aggressive behavior | Pappa et al., 2016 |
| rs7958262 | 12 | 103151021 | 4,16E-04 | *-* | *TXNRD1* | intragenic | Aggressive behavior | Pappa et al., 2016 |
| rs11111939 | 12 | 103152383 | 3,14E-04 | *-* | *TXNRD1* | intragenic | Aggressive behavior | Pappa et al., 2016 |
| rs11111940 | 12 | 103152961 | 4,17E-04 | *-* | *TXNRD1* | intragenic | Aggressive behavior | Pappa et al., 2016 |
| rs10861180 | 12 | 103175129 | 2,05E-04 | *-* | *TXNRD1* | intragenic | Aggressive behavior | Pappa et al., 2016 |
| rs11611207 | 12 | 103727386 | 1,25E-04 | *-* | *SLC41A2* | intragenic | Aggressive behavior | Pappa et al., 2016 |
| rs1394801 | 12 | 115460230 | 7,29E-05 | *-* | *FLJ42957* | 2,5 | Aggressive behavior | Pappa et al., 2016 |
| rs605265 | 12 | 116620279 | 2,54E-04 | *-* | *KSR2* | intragenic | Aggressive behavior | Pappa et al., 2016 |
| rs61956960 | 12 | 121827996 | 4,45E-05 | - | *CCDC62* | intragenic | Aggressive behavior | Brevik et al., 2016 |
| rs2214958 | 12 | 124798552 | 4,69E-04 | *-* | *TMEM132B* | 89,0 | Aggressive behavior | Pappa et al., 2016 |
| rs7397593 | 12 | 124801091 | 4,40E-04 | *-* | *TMEM132B* | 91,6 | Aggressive behavior | Pappa et al., 2016 |
| rs12424368 | 12 | 124802043 | 4,94E-04 | *-* | *TMEM132B* | 92,5 | Aggressive behavior | Pappa et al., 2016 |
| rs16919463 | 12 | 124802759 | 4,72E-04 | *-* | *TMEM132B* | 93,2 | Aggressive behavior | Pappa et al., 2016 |
| rs4765296 | 12 | 124823502 | 4,57E-04 | *-* | *-* | - | Aggressive behavior | Pappa et al., 2016 |
| rs10846979 | 12 | 124850051 | 4,24E-04 | *-* | *-* | - | Aggressive behavior | Pappa et al., 2016 |
| rs11058488 | 12 | 125117158 | 6,56E-06 | - | *-* | - | Aggressive behavior | Brevik et al., 2016 |
| rs77206607 | 12 | 125120316 | 4,17E-06 | - | *-* | - | Aggressive behavior | Brevik et al., 2016 |
| rs12367682 | 12 | 125126979 | 5,77E-06 | - | *-* | - | Aggressive behavior | Brevik et al., 2016 |
| rs138850252 | 12 | 125133199 | 1,97E-06 | - | *-* | - | Aggressive behavior | Brevik et al., 2016 |
| rs11058510 | 12 | 125136995 | 2,03E-06 | - | *-* | - | Aggressive behavior | Brevik et al., 2016 |
| rs77277634 | 12 | 125138597 | 4,61E-06 | - | *-* | - | Aggressive behavior | Brevik et al., 2016 |
| rs11058518 | 12 | 125148566 | 5,65E-06 | - | *-* | - | Aggressive behavior | Brevik et al., 2016 |
| rs117169431 | 12 | 125153763 | 6,38E-06 | - | *-* | - | Aggressive behavior | Brevik et al., 2016 |
| rs117970560 | 12 | 125154268 | 6,38E-06 | - | *-* | - | Aggressive behavior | Brevik et al., 2016 |
| rs7296736 | 12 | 126811567 | 1,61E-04 | *-* | *-* | - | Aggressive behavior | Pappa et al., 2016 |
| rs4760574 | 12 | 127812921 | 1,23E-04 | *-* | *SLC15A4* | 30,8 | Aggressive behavior | Pappa et al., 2016 |
| rs7958082 | 12 | 127812948 | 1,26E-04 | *-* | *SLC15A4* | 30,8 | Aggressive behavior | Pappa et al., 2016 |
| rs2895117 | 12 | 127814267 | 2,55E-05 | *-* | *SLC15A4* | 29,5 | Aggressive behavior | Pappa et al., 2016 |
| rs7958200 | 12 | 128444994 | 2,87E-04 | *-* | *TMEM132D* | intragenic | Aggressive behavior | Pappa et al., 2016 |
| rs11060369 | 12 | 128526150 | 2.3E-05 | - | *TMEM132D* | intragenic | CBCL-DP | Mick et al., 2011 |
| rs61947646 | 13 | 37832077 | 9,02E-06 | - | *UFM1* | intragenic | Aggressive behavior | Brevik et al., 2016 |
| rs61947649 | 13 | 37846554 | 4,31E-05 | - | *UFM1* | 11,4 | Aggressive behavior | Brevik et al., 2016 |
| rs11289732 | 13 | 38669446 | 3,07E-05 | - | *-* | - | ODD | Aebi et al., 2016 |
| rs7989670 | 13 | 38672309 | 3,07E-05 | - | *-* | - | ODD | Aebi et al., 2016 |
| rs7993057 | 13 | 42197052 | 3.0E-05 | - | *C13orf30* | 56.7 | Anger | Merjonen et al., 2011 |
| rs942869 | 13 | 45201953 | 2.5E-05 | - | *SPERT* | 15.3 | Antisocial behavior | Tielbeek et al., 2012 |
| rs2761946 | 13 | 45958619 | 3,77E-04 | *-* | *LRCH1* | 66,7 | Aggressive behavior | Pappa et al., 2016 |
| rs3116620 | 13 | 50116120 | 3,64E-05 | - | *DLEU7* | 68,6 | Aggressive behavior | Brevik et al., 2016 |
| rs3121212 | 13 | 50116560 | 3,72E-05 | - | *DLEU7* | 68,2 | Aggressive behavior | Brevik et al., 2016 |
| rs6561608 | 13 | 50535097 | 3,83E-04 | *-* | *-* | - | Aggressive behavior | Pappa et al., 2016 |
| rs17410833 | 13 | 52254398 | 1,90E-05 | - | *LECT1* | 42,5 | Aggressive behavior | Brevik et al., 2016 |
| rs41423344 | 13 | 52261165 | 2,71E-05 | - | *LECT1* | 49,2 | Aggressive behavior | Brevik et al., 2016 |
| rs625133 | 13 | 52262940 | 4,81E-05 | - | *LECT1* | 51,0 | Aggressive behavior | Brevik et al., 2016 |
| rs6561985 | 13 | 58290774 | 3,14E-05 | - | *-* | - | ODD | Aebi et al., 2016 |
| rs6561987 | 13 | 58292320 | 3,14E-05 | - | *-* | - | ODD | Aebi et al., 2016 |
| rs722122 | 13 | 58297035 | 4,01E-05 | - | *-* | - | ODD | Aebi et al., 2016 |
| rs7326173 | 13 | 58314409 | 4,20E-05 | - | *-* | - | ODD | Aebi et al., 2016 |
| rs7983390 | 13 | 58319712 | 3,72E-05 | - | *-* | - | ODD | Aebi et al., 2016 |
| rs6561989 | 13 | 58322537 | 3,72E-05 | - | *-* | - | ODD | Aebi et al., 2016 |
| rs1333423 | 13 | 58323112 | 3,72E-05 | - | *-* | - | ODD | Aebi et al., 2016 |
| rs7328849 | 13 | 58324900 | 3,72E-05 | - | *-* | - | ODD | Aebi et al., 2016 |
| rs1537449 | 13 | 58327474 | 3,72E-05 | - | *-* | - | ODD | Aebi et al., 2016 |
| rs9538232 | 13 | 58330286 | 2,27E-05 | - | *-* | - | ODD | Aebi et al., 2016 |
| rs9538235 | 13 | 58330682 | 2,62E-05 | - | *-* | - | ODD | Aebi et al., 2016 |
| rs9527910 | 13 | 58332139 | 2,62E-05 | - | *-* | - | ODD | Aebi et al., 2016 |
| rs9527911 | 13 | 58332190 | 3,87E-05 | - | *-* | - | ODD | Aebi et al., 2016 |
| rs12427552 | 13 | 58333616 | 4,12E-05 | - | *-* | - | ODD | Aebi et al., 2016 |
| rs17682784 | 13 | 58357935 | 3,17E-05 | - | *-* | - | ODD | Aebi et al., 2016 |
| rs746717 | 13 | 58374753 | 3,17E-05 | - | *-* | - | ODD | Aebi et al., 2016 |
| rs7981358 | 13 | 60750031 | 3,11E-05 | *-* | *-* | - | Aggressive behavior | Pappa et al., 2016 |
| rs961573 | 13 | 60758080 | 4,96E-05 | *-* | *-* | - | Aggressive behavior | Pappa et al., 2016 |
| rs1483688 | 13 | 60790773 | 1,04E-04 | *-* | *PCDH20* | 91,1 | Aggressive behavior | Pappa et al., 2016 |
| rs7322115 | 13 | 60804005 | 4,82E-04 | *-* | *PCDH20* | 77,8 | Aggressive behavior | Pappa et al., 2016 |
| rs9317178 | 13 | 60804664 | 4,97E-04 | *-* | *PCDH20* | 77,2 | Aggressive behavior | Pappa et al., 2016 |
| rs9317180 | 13 | 60805002 | 4,97E-04 | *-* | *PCDH20* | 76,8 | Aggressive behavior | Pappa et al., 2016 |
| rs9317182 | 13 | 60823473 | 3,92E-04 | *-* | *PCDH20* | 58,4 | Aggressive behavior | Pappa et al., 2016 |
| rs1330598 | 13 | 60831132 | 2,39E-05 | - | *PCDH20* | 50,7 | ODD | Aebi et al., 2016 |
| rs1999246 | 13 | 60835413 | 2,86E-04 | *-* | *PCDH20* | 46,4 | Aggressive behavior | Pappa et al., 2016 |
| rs9528325 | 13 | 60844474 | 1,04E-04 | *-* | *PCDH20* | 37,3 | Aggressive behavior | Pappa et al., 2016 |
| rs17321299 | 13 | 62658948 | 3.4E-05 | - | *-* | - | CD | Anney et al., 2008 |
| rs9572378 | 13 | 69602679 | 2,85E-04 | *-* | *KLHL1* | 22,3 | Aggressive behavior | Pappa et al., 2016 |
| rs17086441 | 13 | 69603108 | 3,21E-04 | *-* | *KLHL1* | 22,7 | Aggressive behavior | Pappa et al., 2016 |
| rs9572380 | 13 | 69603755 | 3,05E-04 | *-* | *KLHL1* | 23,3 | Aggressive behavior | Pappa et al., 2016 |
| rs9564662 | 13 | 69619062 | 2,53E-04 | *-* | *KLHL1* | 38,6 | Aggressive behavior | Pappa et al., 2016 |
| rs9572385 | 13 | 69619293 | 1,01E-04 | *-* | *KLHL1* | 38,9 | Aggressive behavior | Pappa et al., 2016 |
| rs7322990 | 13 | 69622108 | 1,26E-04 | *-* | *KLHL1* | 41,7 | Aggressive behavior | Pappa et al., 2016 |
| rs2949906 | 13 | 84246135 | 4,43E-05 | - | *-* | - | ODD | Aebi et al., 2016 |
| rs17078644 | 13 | 84325593 | 3.6E-05 | - | *-* | - | CD | Anney et al., 2008 |
| rs1538337 | 13 | 94856458 | 8,19E-05 | *-* | *CLDN10* | 27,4 | Aggressive behavior | Pappa et al., 2016 |
| rs1538335 | 13 | 94856834 | 8,65E-05 | *-* | *CLDN10* | 27,0 | Aggressive behavior | Pappa et al., 2016 |
| rs1538334 | 13 | 94856844 | 8,22E-05 | *-* | *CLDN10* | 27,0 | Aggressive behavior | Pappa et al., 2016 |
| rs2993574 | 13 | 94865495 | 2,68E-05 | *-* | *CLDN10* | 18,4 | Aggressive behavior | Pappa et al., 2016 |
| rs7332286 | 13 | 94874887 | 3,12E-04 | *-* | *CLDN10* | 9,0 | Aggressive behavior | Pappa et al., 2016 |
| rs9556480 | 13 | 94877447 | 2,95E-04 | *-* | *CLDN10* | 6,4 | Aggressive behavior | Pappa et al., 2016 |
| rs9556481 | 13 | 94877466 | 2,59E-04 | *-* | *CLDN10* | 6,4 | Aggressive behavior | Pappa et al., 2016 |
| rs7333503 | 13 | 94884481 | 5,69E-05 | *-* | *CLDN10* | intragenic | Aggressive behavior | Pappa et al., 2016 |
| rs11620473 | 13 | 94884949 | 4,32E-05 | *-* | *CLDN10* | intragenic | Aggressive behavior | Pappa et al., 2016 |
| rs9561879 | 13 | 94885297 | 8,09E-05 | *-* | *CLDN10* | intragenic | Aggressive behavior | Pappa et al., 2016 |
| rs565851 | 13 | 95453269 | 1.6E-05 | - | *UGCGL2* | intragenic | CBCL-DP | Mick et al., 2011 |
| rs16951448 | 13 | 95512279 | 4.1E-05 | - | *UGCGL2* | 8.6 | CBCL-DP | Mick et al., 2011 |
| rs9557847 | 13 | 101723545 | 8,38E-05 | *-* | *FGF14* | intragenic | Aggressive behavior | Pappa et al., 2016 |
| rs7336589 | 13 | 101917220 | 4.6E-05 | - | *FGF14* | 65.1 | CBCL-DP | Mick et al., 2011 |
| rs9300745 | 13 | 101918329 | 2.5E-05 | - | *FGF14* | 66.2 | CBCL-DP | Mick et al., 2011 |
| rs9582594 | 13 | 101920990 | 1.6E-05 | - | *FGF14* | 68.9 | CBCL-DP | Mick et al., 2011 |
| rs17633874 | 13 | 101921316 | 1.3E-05 | - | *FGF14* | 69.2 | CBCL-DP | Mick et al., 2011 |
| rs17690437 | 13 | 101921393 | 1.1E-05 | - | *FGF14* | 69.3 | CBCL-DP | Mick et al., 2011 |
| rs1347331 | 13 | 103942694 | 3,50E-04 | *-* | *-* | - | Aggressive behavior | Pappa et al., 2016 |
| rs9300935 | 13 | 103951075 | 3,38E-04 | *-* | *-* | - | Aggressive behavior | Pappa et al., 2016 |
| rs816976 | 13 | 107368910 | 3,16E-04 | *-* | *FAM155A* | 51,8 | Aggressive behavior | Pappa et al., 2016 |
| rs843839 | 13 | 107389473 | 3,06E-04 | *-* | *FAM155A* | 72,4 | Aggressive behavior | Pappa et al., 2016 |
| rs332641 | 13 | 107406147 | 3,10E-04 | *-* | *FAM155A* | 89,1 | Aggressive behavior | Pappa et al., 2016 |
| rs622486 | 13 | 109646292 | 4,35E-04 | *-* | *COL4A1* | intragenic | Aggressive behavior | Pappa et al., 2016 |
| rs635230 | 13 | 109646918 | 4,57E-04 | *-* | *COL4A1* | intragenic | Aggressive behavior | Pappa et al., 2016 |
| rs4771711 | 13 | 110300660 | 4,10E-04 | *-* | *ANKRD10* | 28,2 | Aggressive behavior | Pappa et al., 2016 |
| rs11619266 | 13 | 110302208 | 3,71E-04 | *-* | *ANKRD10* | 26,7 | Aggressive behavior | Pappa et al., 2016 |
| rs2184283 | 14 | 19976454 | 4,44E-05 | *-* | *KLHL33* | 8,0 | Aggressive behavior | Pappa et al., 2016 |
| rs12895453 | 14 | 21938285 | 2.8E-05 | - | *-* | - | Anger | Merjonen et al., 2011 |
| rs2163411 | 14 | 33632644 | 2,35E-04 | *-* | *-* | - | Aggressive behavior | Pappa et al., 2016 |
| rs12890938 | 14 | 39308308 | 3,02E-04 | *-* | *-* | - | Aggressive behavior | Pappa et al., 2016 |
| rs6572288 | 14 | 44437583 | 2.6E-05 | - | *C14orf28* | intragenic | Anger (reaction) | Mick et al., 2014 |
| rs17831706 | 14 | 51825135 | 3.7E-05 | - | *PTGDR* | 11.9 | Anger (temper) | Mick et al., 2014 |
| rs1458114 | 14 | 52093597 | 1,77E-05 | - | *GPR137C* | intragenic | Aggressive behavior | Brevik et al., 2016 |
| rs242585 | 14 | 55805582 | 3,74E-04 | *-* | *PELI2* | intragenic | Aggressive behavior | Pappa et al., 2016 |
| rs2353779 | 14 | 61634220 | 4.9E-05 | - | *SYT16* | intragenic | CD | Anney et al., 2008 |
| rs35010520 | 14 | 65476815 | 1.9E-05 | - | *-* |  | Anger (temper) | Mick et al., 2014 |
| rs28397045 | 14 | 65476893 | 1.1E-05 | - | *-* |  | Anger (temper) | Mick et al., 2014 |
| rs12050199 | 14 | 69024043 | 4.4E-05 | - | *SLC39A9* | 27.0 | Anger | Merjonen et al., 2011 |
| rs175716 | 14 | 75055136 | 4,94E-05 | - | *BATF* | 3,4 | Aggressive behavior | Brevik et al., 2016 |
| rs175717 | 14 | 75055357 | 4,09E-05 | - | *BATF* | 3,2 | Aggressive behavior | Brevik et al., 2016 |
| rs1884008 | 14 | 85054657 | 2,88E-05 | - | *FLRT2* | 11,6 | Aggressive behavior | Brevik et al., 2016 |
| rs10498613 | 14 | 86787235 | 2,93E-05 | - | *-* | - | ODD | Aebi et al., 2016 |
| rs10498614 | 14 | 86787863 | 2,56E-05 | - | *-* | - | ODD | Aebi et al., 2016 |
| rs942746 | 14 | 90181831 | 3.4E-06 | - | *TTC7B* | intragenic | Antisocial behavior | Tielbeek et al., 2012 |
| rs2277512 | 14 | 90183224 | 8.6E-06 | - | *TTC7B* | intragenic | Antisocial behavior | Tielbeek et al., 2012 |
| rs11844114 | 14 | 93530282 | 4,52E-06 | - | *C14orf48* | 3,1 | ODD | Aebi et al., 2016 |
| rs11848552 | 14 | 93530438 | 1,42E-05 | - | *C14orf48* | 3,0 | ODD | Aebi et al., 2016 |
| rs4905137 | 14 | 93535834 | 7,49E-06 | - | *C14orf48* | intragenic | ODD | Aebi et al., 2016 |
| rs749974 | 14 | 95631786 | 2,48E-04 | *-* | *-* | - | Aggressive behavior | Pappa et al., 2016 |
| rs9285600 | 14 | 96348973 | 2.4E-05 | - | *VRK1* | intragenic | Antisocial behavior | Tielbeek et al., 2012 |
| rs10145383 | 14 | 96353248 | 2.4E-05 | - | *VRK1* | intragenic | Antisocial behavior | Tielbeek et al., 2012 |
| rs8013081 | 14 | 96353439 | 1.8E-05 | - | *VRK1* | intragenic | Antisocial behavior | Tielbeek et al., 2012 |
| rs723810 | 14 | 96357723 | 2.4E-05 | - | *VRK1* | intragenic | Antisocial behavior | Tielbeek et al., 2012 |
| rs4905547 | 14 | 96360043 | 2.4E-05 | - | *VRK1* | intragenic | Antisocial behavior | Tielbeek et al., 2012 |
| rs7156355 | 14 | 96363466 | 2.4E-05 | - | *VRK1* | intragenic | Antisocial behavior | Tielbeek et al., 2012 |
| rs10149107 | 14 | 96375679 | 2.3E-05 | - | *VRK1* | intragenic | Antisocial behavior | Tielbeek et al., 2012 |
| rs1951311 | 14 | 96382395 | 2.2E-05 | - | *VRK1* | intragenic | Antisocial behavior | Tielbeek et al., 2012 |
| rs4270096 | 14 | 96385289 | 2.2E-05 | - | *VRK1* | intragenic | Antisocial behavior | Tielbeek et al., 2012 |
| rs12433112 | 14 | 96387676 | 2.2E-05 | - | *VRK1* | intragenic | Antisocial behavior | Tielbeek et al., 2012 |
| rs12882853 | 14 | 96399903 | 2.1E-05 | - | *VRK1* | intragenic | Antisocial behavior | Tielbeek et al., 2012 |
| rs1951312 | 14 | 96404495 | 2.1E-05 | - | *VRK1* | intragenic | Antisocial behavior | Tielbeek et al., 2012 |
| rs1957137 | 14 | 96405781 | 2.1E-05 | - | *VRK1* | intragenic | Antisocial behavior | Tielbeek et al., 2012 |
| rs10146571 | 14 | 96408530 | 2.1E-05 | - | *VRK1* | intragenic | Antisocial behavior | Tielbeek et al., 2012 |
| rs4905557 | 14 | 96411581 | 2.1E-05 | - | *VRK1* | intragenic | Antisocial behavior | Tielbeek et al., 2012 |
| rs4905558 | 14 | 96415559 | 2.1E-05 | - | *VRK1* | intragenic | Antisocial behavior | Tielbeek et al., 2012 |
| rs8013300 | 14 | 96416809 | 1.7E-05 | - | *VRK1* | intragenic | Antisocial behavior | Tielbeek et al., 2012 |
| rs17191485 | 14 | 96417937 | 1.7E-05 | - | *VRK1* | 0.2 | Antisocial behavior | Tielbeek et al., 2012 |
| rs10131201 | 14 | 96421590 | 2.2E-05 | - | *VRK1* | 3.9 | Antisocial behavior | Tielbeek et al., 2012 |
| rs4900351 | 14 | 96423057 | 2.2E-05 | - | *VRK1* | 5.4 | Antisocial behavior | Tielbeek et al., 2012 |
| rs10873476 | 14 | 96432245 | 2.2E-05 | - | *VRK1* | 14.5 | Antisocial behavior | Tielbeek et al., 2012 |
| rs10873477 | 14 | 96432410 | 2.2E-05 | - | *VRK1* | 14.7 | Antisocial behavior | Tielbeek et al., 2012 |
| rs11160375 | 14 | 96432517 | 2.2E-05 | - | *VRK1* | 14.8 | Antisocial behavior | Tielbeek et al., 2012 |
| rs1385551 | 14 | 96444749 | 9.6E-06 | - | *VRK1* | 27.0 | Antisocial behavior | Tielbeek et al., 2012 |
| rs12587324 | 14 | 96450600 | 3.5E-05 | - | *VRK1* | 32.9 | Antisocial behavior | Tielbeek et al., 2012 |
| rs8018103 | 14 | 96458197 | 4.9E-05 | - | *VRK1* | 40.5 | Antisocial behavior | Tielbeek et al., 2012 |
| rs11626300 | 14 | 97963680 | 1,37E-04 | *-* | *-* | - | Aggressive behavior | Pappa et al., 2016 |
| rs12592624 | 15 | 20477046 | 4,68E-04 | *-* | *CYFIP1* | intragenic | Aggressive behavior | Pappa et al., 2016 |
| rs2120968 | 15 | 20483952 | 1,16E-04 | *-* | *CYFIP1* | intragenic | Aggressive behavior | Pappa et al., 2016 |
| rs4779527 | 15 | 29523383 | 6,61E-05 | *-* | *OTUD7A* | 39,2 | Aggressive behavior | Pappa et al., 2016 |
| rs8028114 | 15 | 31119613 | 3.4E-05 | - | *FMN1* | intragenic | CBCL-DP | Mick et al., 2011 |
| rs11636078 | 15 | 31131690 | 2.5E-05 | - | *FMN1* | intragenic | CBCL-DP | Mick et al., 2011 |
| rs11630176 | 15 | 31458133 | 1,18E-04 | *-* | *RYR3* | intragenic | Aggressive behavior | Pappa et al., 2016 |
| rs7164037 | 15 | 35911992 | 4,51E-04 | *-* | *-* | - | Aggressive behavior | Pappa et al., 2016 |
| rs12441065 | 15 | 35912102 | 4,50E-04 | *-* | *-* | - | Aggressive behavior | Pappa et al., 2016 |
| rs4924595 | 15 | 40074898 | 3.5E-05 | - | *PLA2G4E* | intragenic | Anger | Merjonen et al., 2011 |
| rs11854608 | 15 | 45433381 | 1,60E-04 | *-* | *-* | - | Aggressive behavior | Pappa et al., 2016 |
| rs420095 | 15 | 45445506 | 1,12E-04 | *-* | *-* | - | Aggressive behavior | Pappa et al., 2016 |
| rs8034829 | 15 | 46593319 | 4,26E-04 | *-* | *FBN1* | intragenic | Aggressive behavior | Pappa et al., 2016 |
| rs16961065 | 15 | 46593831 | 3,62E-04 | *-* | *FBN1* | intragenic | Aggressive behavior | Pappa et al., 2016 |
| rs6493327 | 15 | 46596720 | 3,54E-04 | *-* | *FBN1* | intragenic | Aggressive behavior | Pappa et al., 2016 |
| rs11634611 | 15 | 46601589 | 2,98E-04 | *-* | *FBN1* | intragenic | Aggressive behavior | Pappa et al., 2016 |
| rs7170686 | 15 | 46608642 | 2,53E-04 | *-* | *FBN1* | intragenic | Aggressive behavior | Pappa et al., 2016 |
| rs8026752 | 15 | 46611062 | 2,72E-04 | *-* | *FBN1* | intragenic | Aggressive behavior | Pappa et al., 2016 |
| rs595244 | 15 | 46628127 | 2,33E-04 | *-* | *FBN1* | intragenic | Aggressive behavior | Pappa et al., 2016 |
| rs1561207 | 15 | 46646263 | 2,41E-04 | *-* | *FBN1* | intragenic | Aggressive behavior | Pappa et al., 2016 |
| rs10744953 | 15 | 46647258 | 2,36E-04 | *-* | *FBN1* | intragenic | Aggressive behavior | Pappa et al., 2016 |
| rs1848050 | 15 | 46649335 | 2,40E-04 | *-* | *FBN1* | intragenic | Aggressive behavior | Pappa et al., 2016 |
| rs627634 | 15 | 46666583 | 2,57E-04 | *-* | *FBN1* | intragenic | Aggressive behavior | Pappa et al., 2016 |
| rs686861 | 15 | 46672297 | 2,49E-04 | *-* | *FBN1* | intragenic | Aggressive behavior | Pappa et al., 2016 |
| rs2456475 | 15 | 46673169 | 2,41E-04 | *-* | *FBN1* | intragenic | Aggressive behavior | Pappa et al., 2016 |
| rs655015 | 15 | 46678399 | 2,39E-04 | *-* | *FBN1* | intragenic | Aggressive behavior | Pappa et al., 2016 |
| rs682737 | 15 | 46679063 | 2,35E-04 | *-* | *FBN1* | intragenic | Aggressive behavior | Pappa et al., 2016 |
| rs2455925 | 15 | 46680941 | 2,35E-04 | *-* | *FBN1* | intragenic | Aggressive behavior | Pappa et al., 2016 |
| rs591519 | 15 | 46681492 | 2,36E-04 | *-* | *FBN1* | intragenic | Aggressive behavior | Pappa et al., 2016 |
| rs636178 | 15 | 46684574 | 2,48E-04 | *-* | *FBN1* | intragenic | Aggressive behavior | Pappa et al., 2016 |
| rs2466790 | 15 | 46686691 | 2,54E-04 | *-* | *FBN1* | intragenic | Aggressive behavior | Pappa et al., 2016 |
| rs1678981 | 15 | 46687389 | 2,56E-04 | *-* | *FBN1* | intragenic | Aggressive behavior | Pappa et al., 2016 |
| rs1678982 | 15 | 46688712 | 2,28E-04 | *-* | *FBN1* | intragenic | Aggressive behavior | Pappa et al., 2016 |
| rs1018148 | 15 | 46690418 | 2,75E-04 | *-* | *FBN1* | intragenic | Aggressive behavior | Pappa et al., 2016 |
| rs1036476 | 15 | 46702067 | 2,70E-04 | *-* | *FBN1* | intragenic | Aggressive behavior | Pappa et al., 2016 |
| rs689304 | 15 | 46709652 | 2,70E-04 | *-* | *FBN1* | intragenic | Aggressive behavior | Pappa et al., 2016 |
| rs625034 | 15 | 46713494 | 2,66E-04 | *-* | *FBN1* | intragenic | Aggressive behavior | Pappa et al., 2016 |
| rs2440339 | 15 | 50822647 | 3,22E-04 | *-* | *ONECUT1* | 14,0 | Aggressive behavior | Pappa et al., 2016 |
| rs572288 | 15 | 52448312 | 1,82E-04 | *-* | *UNC13C* | intragenic | Aggressive behavior | Pappa et al., 2016 |
| rs8031781 | 15 | 52462977 | 2,05E-04 | *-* | *UNC13C* | intragenic | Aggressive behavior | Pappa et al., 2016 |
| rs573740 | 15 | 52470723 | 1,22E-04 | *-* | *UNC13C* | intragenic | Aggressive behavior | Pappa et al., 2016 |
| rs11637779 | 15 | 55998112 | 4.3E-04 | 3.9E-03 | *ALDH1A2* | 34.8 | AB+/CU+ | Viding et al., 2010 |
| rs76899206 | 15 | 59627740 | 1,69E-05 | - | *-* | - | Aggressive behavior | Brevik et al., 2016 |
| rs11631621 | 15 | 59882136 | 4,16E-04 | *-* | *VPS13C* | 49,7 | Aggressive behavior | Pappa et al., 2016 |
| rs7164017 | 15 | 60675326 | 3,57E-05 | - | *TLN2* | 51,5 | ODD | Aebi et al., 2016 |
| rs12903880 | 15 | 64235179 | 3,47E-04 | *-* | *MEGF11* | intragenic | Aggressive behavior | Pappa et al., 2016 |
| rs7359178 | 15 | 64768383 | 1,55E-05 | *-* | *SMAD6* | 13,3 | Aggressive behavior | Pappa et al., 2016 |
| rs16950152 | 15 | 64777477 | 3,93E-04 | *-* | *SMAD6* | 4,2 | Aggressive behavior | Pappa et al., 2016 |
| rs16971853 | 15 | 78305727 | 2,08E-04 | *-* | *FAH* | 40,0 | Aggressive behavior | Pappa et al., 2016 |
| rs950168 | 15 | 82497201 | 4,07E-05 | - | *ADAMTSL3* | intragenic | ODD | Aebi et al., 2016 |
| rs2132655 | 15 | 84695591 | 4,44E-04 | *-* | *AGBL1* | intragenic | Aggressive behavior | Pappa et al., 2016 |
| rs1845933 | 15 | 84696423 | 3,67E-04 | *-* | *AGBL1* | intragenic | Aggressive behavior | Pappa et al., 2016 |
| rs2346307 | 15 | 84705704 | 3,83E-04 | *-* | *AGBL1* | intragenic | Aggressive behavior | Pappa et al., 2016 |
| rs11073632 | 15 | 84710398 | 2,00E-04 | *-* | *AGBL1* | intragenic | Aggressive behavior | Pappa et al., 2016 |
| rs1897266 | 15 | 84751810 | 1,86E-04 | *-* | *AGBL1* | intragenic | Aggressive behavior | Pappa et al., 2016 |
| rs1431237 | 15 | 84751957 | 1,95E-04 | *-* | *AGBL1* | intragenic | Aggressive behavior | Pappa et al., 2016 |
| rs8029257 | 15 | 84752366 | 1,96E-04 | *-* | *AGBL1* | intragenic | Aggressive behavior | Pappa et al., 2016 |
| rs7168149 | 15 | 84752711 | 2,01E-04 | *-* | *AGBL1* | intragenic | Aggressive behavior | Pappa et al., 2016 |
| rs1431242 | 15 | 84754072 | 1,81E-04 | *-* | *AGBL1* | intragenic | Aggressive behavior | Pappa et al., 2016 |
| rs4493015 | 15 | 84761279 | 3,26E-04 | *-* | *AGBL1* | intragenic | Aggressive behavior | Pappa et al., 2016 |
| rs4513061 | 15 | 84772636 | 1,96E-04 | *-* | *AGBL1* | intragenic | Aggressive behavior | Pappa et al., 2016 |
| rs16977283 | 15 | 84797864 | 3,05E-04 | *-* | *AGBL1* | intragenic | Aggressive behavior | Pappa et al., 2016 |
| rs12441378 | 15 | 88559728 | 1.9E-05 | - | *SEMA4B* | intragenic | Antisocial behavior | Tielbeek et al., 2012 |
| rs8031100 | 15 | 88561178 | 1.9E-05 | - | *SEMA4B* | intragenic | Antisocial behavior | Tielbeek et al., 2012 |
| rs1543115 | 15 | 88562140 | 3.9E-05 | - | *SEMA4B* | intragenic | Antisocial behavior | Tielbeek et al., 2012 |
| rs3751656 | 15 | 88572708 | 2.4E-06 | - | *SEMA4B* | intragenic | Antisocial behavior | Tielbeek et al., 2012 |
| rs3829490 | 15 | 88574063 | 1.8E-06 | - | *SEMA4B* | 0.2 | Antisocial behavior | Tielbeek et al., 2012 |
| rs9920862 | 15 | 88607144 | 1.2E-05 | - | *NGRN* | 2.8 | Antisocial behavior | Tielbeek et al., 2012 |
| rs4932549 | 15 | 89867000 | 1,17E-04 | *-* | *-* | - | Aggressive behavior | Pappa et al., 2016 |
| rs4299128 | 15 | 89869918 | 1,42E-05 | *-* | *-* | - | Aggressive behavior | Pappa et al., 2016 |
| rs7170769 | 15 | 89871209 | 1,93E-05 | *-* | *-* | - | Aggressive behavior | Pappa et al., 2016 |
| rs4932550 | 15 | 89871986 | 1,41E-05 | *-* | *-* | - | Aggressive behavior | Pappa et al., 2016 |
| rs4932551 | 15 | 89872962 | 6,60E-05 | *-* | *-* | - | Aggressive behavior | Pappa et al., 2016 |
| rs6496814 | 15 | 89873848 | 4,12E-05 | *-* | *-* | - | Aggressive behavior | Pappa et al., 2016 |
| rs4536445 | 15 | 89874215 | 4,86E-05 | *-* | *-* | - | Aggressive behavior | Pappa et al., 2016 |
| rs4380038 | 15 | 89874261 | 4,45E-05 | *-* | *-* | - | Aggressive behavior | Pappa et al., 2016 |
| rs4244894 | 15 | 89874662 | 3,98E-05 | *-* | *-* | - | Aggressive behavior | Pappa et al., 2016 |
| rs8034160 | 15 | 89875191 | 4,88E-05 | *-* | *-* | - | Aggressive behavior | Pappa et al., 2016 |
| rs2067566 | 15 | 89877963 | 4,89E-05 | *-* | *-* | - | Aggressive behavior | Pappa et al., 2016 |
| rs7166681 | 15 | 89879467 | 4,92E-05 | *-* | *-* | - | Aggressive behavior | Pappa et al., 2016 |
| rs6496820 | 15 | 89881482 | 4,05E-05 | *-* | *-* | - | Aggressive behavior | Pappa et al., 2016 |
| rs3924397 | 15 | 89881911 | 4,06E-05 | *-* | *-* | - | Aggressive behavior | Pappa et al., 2016 |
| rs12443353 | 15 | 89882084 | 5,77E-05 | *-* | *-* | - | Aggressive behavior | Pappa et al., 2016 |
| rs12898526 | 15 | 89882242 | 9,82E-05 | *-* | *-* | - | Aggressive behavior | Pappa et al., 2016 |
| rs7498047 | 15 | 89882445 | 9,05E-05 | *-* | *-* | - | Aggressive behavior | Pappa et al., 2016 |
| rs6496821 | 15 | 89883765 | 9,55E-05 | *-* | *-* | - | Aggressive behavior | Pappa et al., 2016 |
| rs8038691 | 15 | 89884762 | 9,94E-05 | *-* | *-* | - | Aggressive behavior | Pappa et al., 2016 |
| rs7180531 | 15 | 89886778 | 1,01E-04 | *-* | *-* | - | Aggressive behavior | Pappa et al., 2016 |
| rs6496823 | 15 | 89887245 | 8,75E-05 | *-* | *-* | - | Aggressive behavior | Pappa et al., 2016 |
| rs8027786 | 15 | 89888345 | 3,84E-04 | *-* | *-* | - | Aggressive behavior | Pappa et al., 2016 |
| rs4395051 | 15 | 89888899 | 3,04E-04 | *-* | *-* | - | Aggressive behavior | Pappa et al., 2016 |
| rs4509991 | 15 | 89889045 | 3,59E-04 | *-* | *-* | - | Aggressive behavior | Pappa et al., 2016 |
| rs7496485 | 15 | 89889303 | 3,48E-04 | *-* | *-* | - | Aggressive behavior | Pappa et al., 2016 |
| rs6496826 | 15 | 89890810 | 3,87E-04 | *-* | *-* | - | Aggressive behavior | Pappa et al., 2016 |
| rs2238441 | 16 | 3975408 | 4,88E-05 | - | *ADCY9* | intragenic | Aggressive behavior | Brevik et al., 2016 |
| rs4786651 | 16 | 5221742 | 4,76E-05 | - | *-* | - | ODD | Aebi et al., 2016 |
| rs7204436 | 16 | 5223492 | 1,98E-07 | - | *-* | - | ODD | Aebi et al., 2016 |
| rs8059231 | 16 | 5790794 | 3.3E-03 | 4.9E-03 | *-* | - | AB+/CU+ | Viding et al., 2010 |
| rs8062784 | 16 | 6086635 | 2.3E-05 | - | *A2BP1* | intragenic | Anger | Merjonen et al., 2011 |
| rs809682 | 16 | 6336374 | 2,62E-04 | *-* | *A2BP1* | intragenic | Aggressive behavior | Pappa et al., 2016 |
| rs12922093 | 16 | 6336949 | 3,21E-04 | *-* | *A2BP1* | intragenic | Aggressive behavior | Pappa et al., 2016 |
| rs12373031 | 16 | 6337438 | 4,93E-04 | *-* | *A2BP1* | intragenic | Aggressive behavior | Pappa et al., 2016 |
| rs10521042 | 16 | 6339838 | 4,35E-04 | *-* | *A2BP1* | intragenic | Aggressive behavior | Pappa et al., 2016 |
| rs12921846 | 16 | 6850386 | 1.4E-05 | - | *A2BP1* | intragenic | CD | Anney et al., 2008 |
| rs10153149 | 16 | 6865241 | 3.6E-05 | - | *A2BP1* | intragenic | CD | Anney et al., 2008 |
| rs2360851 | 16 | 8269679 | 2,69E-04 | *-* | *-* | - | Aggressive behavior | Pappa et al., 2016 |
| rs7191090 | 16 | 8273810 | 4,10E-04 | *-* | *-* | - | Aggressive behavior | Pappa et al., 2016 |
| rs11077287 | 16 | 8277002 | 4,84E-04 | *-* | *-* | - | Aggressive behavior | Pappa et al., 2016 |
| rs1299926 | 16 | 8778582 | 2.3E-05 | - | *ABAT* | intragenic | Anger (temper) | Mick et al., 2014 |
| rs1019551 | 16 | 11304608 | 4,88E-04 | *-* | *PRM1* | 21,9 | Aggressive behavior | Pappa et al., 2016 |
| rs404854 | 16 | 11984242 | 3,90E-04 | *-* | *RUNDC2A* | intragenic | Aggressive behavior | Pappa et al., 2016 |
| rs386430 | 16 | 11985678 | 4,12E-04 | *-* | *RUNDC2A* | intragenic | Aggressive behavior | Pappa et al., 2016 |
| rs383972 | 16 | 11986388 | 4,22E-04 | *-* | *RUNDC2A* | intragenic | Aggressive behavior | Pappa et al., 2016 |
| rs398700 | 16 | 11986458 | 4,27E-04 | *-* | *RUNDC2A* | intragenic | Aggressive behavior | Pappa et al., 2016 |
| rs434455 | 16 | 11988043 | 4,21E-04 | *-* | *RUNDC2A* | intragenic | Aggressive behavior | Pappa et al., 2016 |
| rs2214197 | 16 | 20437247 | 1,17E-04 | *-* | *ACSM2B* | 18,3 | Aggressive behavior | Pappa et al., 2016 |
| rs4783392 | 16 | 20437735 | 1,22E-04 | *-* | *ACSM2B* | 17,9 | Aggressive behavior | Pappa et al., 2016 |
| rs143400234 | 16 | 21501790 | 4,04E-05 | - | *METTL9* | 16,6 | Aggressive behavior | Brevik et al., 2016 |
| rs11860713 | 16 | 22922511 | 2,87E-04 | *-* | *USP31* | 57,7 | Aggressive behavior | Pappa et al., 2016 |
| rs4783502 | 16 | 22932368 | 4,89E-04 | *-* | *USP31* | 47,9 | Aggressive behavior | Pappa et al., 2016 |
| rs2269738 | 16 | 23051377 | 3,90E-04 | *-* | *USP31* | intragenic | Aggressive behavior | Pappa et al., 2016 |
| rs7202243 | 16 | 23094518 | 3,46E-04 | *-* | *SCNN1G* | 7,0 | Aggressive behavior | Pappa et al., 2016 |
| rs7200952 | 16 | 23113791 | 2,23E-04 | *-* | *SCNN1G* | intragenic | Aggressive behavior | Pappa et al., 2016 |
| rs4486893 | 16 | 23126711 | 2,63E-04 | *-* | *SCNN1G* | intragenic | Aggressive behavior | Pappa et al., 2016 |
| rs7189187 | 16 | 25414534 | 3.5E-05 | - | *-* | - | Antisocial behavior | Tielbeek et al., 2012 |
| rs152709 | 16 | 47916202 | 1,29E-04 | *-* | *CBLN1* | 43,0 | Aggressive behavior | Pappa et al., 2016 |
| rs27789 | 16 | 47921338 | 1,19E-04 | *-* | *C16orf78* | 44,0 | Aggressive behavior | Pappa et al., 2016 |
| rs27790 | 16 | 47921645 | 1,18E-04 | *-* | *C16orf78* | 43,7 | Aggressive behavior | Pappa et al., 2016 |
| rs27792 | 16 | 47922435 | 1,44E-04 | *-* | *C16orf78* | 42,9 | Aggressive behavior | Pappa et al., 2016 |
| rs9937180 | 16 | 48530108 | 3.1E-05 | - | *TMEM188* | 86.6 | CBCL-DP | Mick et al., 2011 |
| rs4785371 | 16 | 48538252 | 3.8E-05 | - | *TMEM188* | 78.4 | CBCL-DP | Mick et al., 2011 |
| rs12600304 | 16 | 56579637 | 2,88E-06 | - | *TEPP* | 0,1 | Aggressive behavior | Brevik et al., 2016 |
| rs116910725 | 16 | 60641528 | 1,61E-05 | - | *CDH8* | 14,0 | Aggressive behavior | Brevik et al., 2016 |
| rs16973500 | 16 | 70522697 | 7.4E-06 | - | *KIAA0174* | 2.3 | CD | Anney et al., 2008 |
| rs11149776 | 16 | 73484021 | 4,22E-04 | *-* | *WDR59* | intragenic | Aggressive behavior | Pappa et al., 2016 |
| rs10514386 | 16 | 73484306 | 1,83E-04 | *-* | *WDR59* | intragenic | Aggressive behavior | Pappa et al., 2016 |
| rs11149777 | 16 | 73484448 | 4,46E-04 | *-* | *WDR59* | intragenic | Aggressive behavior | Pappa et al., 2016 |
| rs4888307 | 16 | 73484559 | 1,84E-04 | *-* | *WDR59* | intragenic | Aggressive behavior | Pappa et al., 2016 |
| rs11865599 | 16 | 73485702 | 4,72E-04 | *-* | *WDR59* | intragenic | Aggressive behavior | Pappa et al., 2016 |
| rs11864349 | 16 | 73485809 | 2,37E-04 | *-* | *WDR59* | intragenic | Aggressive behavior | Pappa et al., 2016 |
| rs7194094 | 16 | 79925389 | 3,76E-04 | *-* | *GAN* | intragenic | Aggressive behavior | Pappa et al., 2016 |
| rs16955063 | 16 | 79926014 | 4,85E-04 | *-* | *GAN* | intragenic | Aggressive behavior | Pappa et al., 2016 |
| rs1820260 | 16 | 79932005 | 3,41E-04 | *-* | *GAN* | intragenic | Aggressive behavior | Pappa et al., 2016 |
| rs12921222 | 16 | 79937908 | 4,24E-04 | *-* | *GAN* | intragenic | Aggressive behavior | Pappa et al., 2016 |
| rs12933277 | 16 | 79940357 | 4,81E-04 | *-* | *GAN* | intragenic | Aggressive behavior | Pappa et al., 2016 |
| rs455617 | 16 | 82366257 | 1,90E-05 | - | *CDH13* | intragenic | Aggressive behavior | Brevik et al., 2016 |
| rs244849 | 16 | 82859375 | 3.3E-05 | - | *WFDC1* | 26.5 | CBCL-DP | Mick et al., 2011 |
| rs2362399 | 16 | 84361180 | 1.9E-05 | - | *COX4NB* | 8.6 | Antisocial behavior | Tielbeek et al., 2012 |
| rs301152 | 16 | 84363395 | 2.0E-05 | - | *COX4NB* | 6.3 | Antisocial behavior | Tielbeek et al., 2012 |
| rs11860152 | 16 | 84374825 | 1.1E-05 | - | *COX4NB* | intragenic | Antisocial behavior | Tielbeek et al., 2012 |
| rs11866179 | 16 | 84375919 | 1.1E-05 | - | *COX4NB* | intragenic | Antisocial behavior | Tielbeek et al., 2012 |
| rs896253 | 16 | 84377703 | 1.1E-05 | - | *COX4NB* | intragenic | Antisocial behavior | Tielbeek et al., 2012 |
| rs11644720 | 16 | 84384581 | 3.1E-05 | - | *COX4NB* | intragenic | Antisocial behavior | Tielbeek et al., 2012 |
| rs11647655 | 16 | 84384600 | 3.1E-05 | - | *COX4NB* | intragenic | Antisocial behavior | Tielbeek et al., 2012 |
| rs16939756 | 16 | 84384901 | 4.5E-05 | - | *COX4NB* | intragenic | Antisocial behavior | Tielbeek et al., 2012 |
| rs16939766 | 16 | 84385854 | 4.5E-05 | - | *COX4NB* | intragenic | Antisocial behavior | Tielbeek et al., 2012 |
| rs11642887 | 16 | 84839194 | 4,11E-04 | *-* | *-* | - | Aggressive behavior | Pappa et al., 2016 |
| rs2047396 | 16 | 84840332 | 3,26E-04 | *-* | *-* | - | Aggressive behavior | Pappa et al., 2016 |
| rs3922878 | 16 | 85412205 | 3.5E-05 | - | *-* | - | Anger (reaction) | Mick et al., 2014 |
| rs62072697 | 17 | 5294578 | 4,31E-05 | - | *DHX33* | intragenic | Aggressive behavior | Brevik et al., 2016 |
| rs11656526 | 17 | 11289530 | 2.0E-05 | - | *-* | - | Anger | Merjonen et al., 2011 |
| rs7207585 | 17 | 13242886 | 3,52E-05 | *-* | *HS3ST3A1* | 96,8 | Aggressive behavior | Pappa et al., 2016 |
| rs1561426 | 17 | 14575303 | 3,87E-04 | *-* | *FLJ45831* | 36,3 | Aggressive behavior | Pappa et al., 2016 |
| rs1000724 | 17 | 14579993 | 2,33E-04 | *-* | *FLJ45831* | 31,6 | Aggressive behavior | Pappa et al., 2016 |
| rs4627401 | 17 | 40599817 | 3,41E-04 | *-* | *HEXIM2* | intragenic | Aggressive behavior | Pappa et al., 2016 |
| rs6504605 | 17 | 44757715 | 4,38E-04 | *-* | *ZNF652* | intragenic | Aggressive behavior | Pappa et al., 2016 |
| rs2676795 | 17 | 45076014 | 4.6E-05 | - | *SPOP* | intragenic | Anger | Merjonen et al., 2011 |
| rs4541132 | 17 | 51518635 | 4,98E-04 | *-* | *ANKFN1* | 67,2 | Aggressive behavior | Pappa et al., 2016 |
| rs2079514 | 17 | 66425059 | 5,73E-06 | *-* | *-* | - | Aggressive behavior | Pappa et al., 2016 |
| rs2079515 | 17 | 66425101 | 4,29E-06 | *-* | *-* | - | Aggressive behavior | Pappa et al., 2016 |
| rs12950250 | 17 | 66438235 | 5,76E-05 | *-* | *-* | - | Aggressive behavior | Pappa et al., 2016 |
| rs2041054 | 17 | 66440761 | 6,52E-05 | *-* | *-* | - | Aggressive behavior | Pappa et al., 2016 |
| rs6501425 | 17 | 66447427 | 4,69E-05 | *-* | *-* | - | Aggressive behavior | Pappa et al., 2016 |
| rs590040 | 17 | 74030083 | 4,02E-05 | - | *PGS1* | 97,9 | ODD | Aebi et al., 2016 |
| rs618588 | 18 | 2153315 | 3,91E-04 | *-* | *-* | - | Aggressive behavior | Pappa et al., 2016 |
| rs885564 | 18 | 2329141 | 3,76E-04 | *-* | *-* | - | Aggressive behavior | Pappa et al., 2016 |
| rs578839 | 18 | 7448215 | 4,93E-04 | *-* | *-* | - | Aggressive behavior | Pappa et al., 2016 |
| rs679231 | 18 | 9685356 | 4.6E-05 | - | *RAB31* | 12.9 | Anger (temper) | Mick et al., 2014 |
| rs114148114 | 18 | 19576319 | 3,53E-05 | - | *LAMA3* | intragenic | Aggressive behavior | Brevik et al., 2016 |
| rs3934832 | 18 | 20965968 | 1,68E-04 | *-* | *ZNF521* | intragenic | Aggressive behavior | Pappa et al., 2016 |
| rs4459629 | 18 | 20975513 | 3,01E-04 | *-* | *ZNF521* | intragenic | Aggressive behavior | Pappa et al., 2016 |
| rs9646558 | 18 | 20977078 | 2,44E-04 | *-* | *ZNF521* | intragenic | Aggressive behavior | Pappa et al., 2016 |
| rs4393651 | 18 | 20978532 | 2,95E-04 | *-* | *ZNF521* | intragenic | Aggressive behavior | Pappa et al., 2016 |
| rs10775514 | 18 | 20979671 | 3,33E-04 | *-* | *ZNF521* | intragenic | Aggressive behavior | Pappa et al., 2016 |
| rs12968618 | 18 | 20982975 | 4,69E-04 | *-* | *ZNF521* | intragenic | Aggressive behavior | Pappa et al., 2016 |
| rs9967300 | 18 | 20983647 | 4,63E-04 | *-* | *ZNF521* | intragenic | Aggressive behavior | Pappa et al., 2016 |
| rs4377227 | 18 | 20987826 | 4,83E-04 | *-* | *ZNF521* | intragenic | Aggressive behavior | Pappa et al., 2016 |
| rs7229946 | 18 | 20992999 | 4,29E-04 | *-* | *ZNF521* | intragenic | Aggressive behavior | Pappa et al., 2016 |
| rs4528662 | 18 | 20994956 | 1,96E-04 | *-* | *ZNF521* | intragenic | Aggressive behavior | Pappa et al., 2016 |
| rs4629056 | 18 | 21000545 | 1,48E-04 | *-* | *ZNF521* | intragenic | Aggressive behavior | Pappa et al., 2016 |
| rs2339102 | 18 | 22051851 | 3,31E-04 | *-* | *TAF4B* | 8,6 | Aggressive behavior | Pappa et al., 2016 |
| rs74492028 | 18 | 31925003 | 4,99E-05 | - | *SLC39A6* | 17,5 | Aggressive behavior | Brevik et al., 2016 |
| rs111340016 | 18 | 31925089 | 4,99E-05 | - | *SLC39A6* | 17,4 | Aggressive behavior | Brevik et al., 2016 |
| rs75247851 | 18 | 31927716 | 4,99E-05 | - | *SLC39A6* | 14,8 | Aggressive behavior | Brevik et al., 2016 |
| rs11659339 | 18 | 36212896 | 1,81E-04 | *-* | *-* | - | Aggressive behavior | Pappa et al., 2016 |
| rs17688770 | 18 | 36904325 | 3,53E-04 | *-* | *-* | - | Aggressive behavior | Pappa et al., 2016 |
| rs1791314 | 18 | 55658599 | 3,27E-04 | *-* | *PMAIP1* | 59,6 | Aggressive behavior | Pappa et al., 2016 |
| rs692916 | 18 | 58676372 | 8,50E-06 | - | *PHLPP* | intragenic | Aggressive behavior | Brevik et al., 2016 |
| rs645525 | 18 | 58677882 | 1,89E-05 | - | *PHLPP* | intragenic | Aggressive behavior | Brevik et al., 2016 |
| rs540128 | 18 | 58695331 | 1,67E-05 | - | *PHLPP* | intragenic | Aggressive behavior | Brevik et al., 2016 |
| rs7230742 | 18 | 74307634 | 4,18E-04 | *-* | *-* | - | Aggressive behavior | Pappa et al., 2016 |
| rs7230768 | 18 | 74307686 | 4,15E-04 | *-* | *-* | - | Aggressive behavior | Pappa et al., 2016 |
| rs3746130 | 19 | 5191392 | 2.0E-05 | - | *PTPRS* | intragenic | Anger | Merjonen et al., 2011 |
| rs17515517 | 19 | 8905534 | 2.1E-05 | - | *MUC16* | intragenic | Anger | Merjonen et al., 2011 |
| rs17682497 | 19 | 15657542 | 5,96E-05 | *-* | *CYP4F12* | intragenic | Aggressive behavior | Pappa et al., 2016 |
| rs3096092 | 19 | 20830470 | 2,24E-05 | - | *ZNF85* | 67,5 | Aggressive behavior | Brevik et al., 2016 |
| rs2301735 | 19 | 41269390 | 4,16E-04 | *-* | *WDR62* | intragenic | Aggressive behavior | Pappa et al., 2016 |
| rs4806270 | 19 | 41273817 | 4,88E-05 | - | *WDR62* | intragenic | Aggressive behavior | Brevik et al., 2016 |
| rs749818 | 19 | 51188633 | 1,61E-04 | *-* | *CCDC61* | 1,5 | Aggressive behavior | Pappa et al., 2016 |
| rs892217 | 19 | 51190193 | 2,78E-04 | *-* | *CCDC61* | intragenic | Aggressive behavior | Pappa et al., 2016 |
| rs12463001 | 19 | 51190879 | 3,17E-04 | *-* | *CCDC61* | intragenic | Aggressive behavior | Pappa et al., 2016 |
| rs11878454 | 19 | 51191921 | 4,25E-04 | *-* | *CCDC61* | intragenic | Aggressive behavior | Pappa et al., 2016 |
| rs4801843 | 19 | 55851001 | 1,94E-04 | *-* | *SHANK1* | 5,9 | Aggressive behavior | Pappa et al., 2016 |
| rs73062800 | 19 | 61970190 | 4,67E-05 | - | *ZIM2* | 7,5 | Aggressive behavior | Brevik et al., 2016 |
| rs17724512 | 20 | 6612177 | 1.9E-05 | - | *BMP2* | 84.6 | Anger | Merjonen et al., 2011 |
| rs6074574 | 20 | 13183157 | 3,97E-04 | *-* | *C20orf82* | intragenic | Aggressive behavior | Pappa et al., 2016 |
| rs11697805 | 20 | 15977530 | 5,21E-05 | *-* | *MACROD2* | intragenic | Aggressive behavior | Pappa et al., 2016 |
| rs6080106 | 20 | 15977837 | 4,53E-04 | *-* | *MACROD2* | intragenic | Aggressive behavior | Pappa et al., 2016 |
| rs16997014 | 20 | 15979369 | 4,21E-04 | *-* | *MACROD2* | intragenic | Aggressive behavior | Pappa et al., 2016 |
| rs1225891 | 20 | 15981185 | 3,66E-04 | *-* | *MACROD2* | intragenic | Aggressive behavior | Pappa et al., 2016 |
| rs912185 | 20 | 15984149 | 1,53E-04 | *-* | *MACROD2* | 2,3 | Aggressive behavior | Pappa et al., 2016 |
| rs1225893 | 20 | 15985325 | 1,70E-04 | *-* | *MACROD2* | 3,3 | Aggressive behavior | Pappa et al., 2016 |
| rs1225895 | 20 | 15985834 | 1,75E-04 | *-* | *MACROD2* | 4,3 | Aggressive behavior | Pappa et al., 2016 |
| rs6043707 | 20 | 15989628 | 1,93E-04 | *-* | *MACROD2* | 7,3 | Aggressive behavior | Pappa et al., 2016 |
| rs6105975 | 20 | 18779315 | 4,20E-04 | *-* | *C20orf79* | 36,3 | Aggressive behavior | Pappa et al., 2016 |
| rs6088857 | 20 | 29690425 | 9,88E-06 | - | *COX4I2* | intragenic | ODD | Aebi et al., 2016 |
| rs6088864 | 20 | 29691818 | 9,88E-06 | - | *COX4I2* | intragenic | ODD | Aebi et al., 2016 |
| rs6060446 | 20 | 29695298 | 9,88E-06 | - | *COX4I2* | intragenic | ODD | Aebi et al., 2016 |
| rs6120970 | 20 | 29696113 | 6,42E-06 | - | *COX4I2* | intragenic | ODD | Aebi et al., 2016 |
| rs7272062 | 20 | 29722806 | 4,44E-06 | - | *BCL2L1* | intragenic | ODD | Aebi et al., 2016 |
| rs6119651 | 20 | 29722907 | 4,44E-06 | - | *BCL2L1* | intragenic | ODD | Aebi et al., 2016 |
| rs6060763 | 20 | 29745884 | 3,82E-06 | - | *BCL2L1* | intragenic | ODD | Aebi et al., 2016 |
| rs1994251 | 20 | 29750989 | 3,82E-06 | - | *BCL2L1* | intragenic | ODD | Aebi et al., 2016 |
| rs1994250 | 20 | 29751155 | 3,82E-06 | - | *BCL2L1* | intragenic | ODD | Aebi et al., 2016 |
| rs6060793 | 20 | 29752491 | 3,82E-06 | - | *BCL2L1* | intragenic | ODD | Aebi et al., 2016 |
| rs6060812 | 20 | 29756464 | 3,82E-06 | - | *BCL2L1* | intragenic | ODD | Aebi et al., 2016 |
| rs6060821 | 20 | 29757695 | 3,82E-06 | - | *BCL2L1* | intragenic | ODD | Aebi et al., 2016 |
| rs6058421 | 20 | 29758674 | 3,82E-06 | - | *BCL2L1* | intragenic | ODD | Aebi et al., 2016 |
| rs7354225 | 20 | 29760542 | 3,82E-06 | - | *BCL2L1* | intragenic | ODD | Aebi et al., 2016 |
| rs6060870 | 20 | 29766960 | 3,82E-06 | - | *BCL2L1* | intragenic | ODD | Aebi et al., 2016 |
| rs3181073 | 20 | 29770860 | 3,82E-06 | - | *BCL2L1* | intragenic | ODD | Aebi et al., 2016 |
| rs2376996 | 20 | 29780328 | 3,63E-06 | - | *BCL2L1* | 6,0 | ODD | Aebi et al., 2016 |
| rs6089055 | 20 | 29787706 | 3,63E-06 | - | *TPX2* | 2,9 | ODD | Aebi et al., 2016 |
| rs6060912 | 20 | 29789416 | 6,61E-06 | - | *TPX2* | 1,1 | ODD | Aebi et al., 2016 |
| rs6089058 | 20 | 29789999 | 6,87E-06 | - | *TPX2* | 0,6 | ODD | Aebi et al., 2016 |
| rs6058448 | 20 | 29796057 | 9,87E-06 | - | *TPX2* | intragenic | ODD | Aebi et al., 2016 |
| rs6060923 | 20 | 29800316 | 6,87E-06 | - | *TPX2* | intragenic | ODD | Aebi et al., 2016 |
| rs6058450 | 20 | 29807572 | 6,87E-06 | - | *TPX2* | intragenic | ODD | Aebi et al., 2016 |
| rs6089070 | 20 | 29841434 | 6,87E-06 | - | *TPX2* | intragenic | ODD | Aebi et al., 2016 |
| rs6089071 | 20 | 29842045 | 6,87E-06 | - | *TPX2* | intragenic | ODD | Aebi et al., 2016 |
| rs3203770 | 20 | 29845976 | 4,86E-06 | - | *TPX2* | intragenic | ODD | Aebi et al., 2016 |
| rs6058463 | 20 | 29848853 | 4,08E-06 | - | *TPX2* | intragenic | ODD | Aebi et al., 2016 |
| rs6119725 | 20 | 29849218 | 4,08E-06 | - | *TPX2* | intragenic | ODD | Aebi et al., 2016 |
| rs6060960 | 20 | 29858178 | 3,00E-06 | - | *TPX2* | 4,9 | ODD | Aebi et al., 2016 |
| rs6121242 | 20 | 29874538 | 9,79E-06 | - | *MYLK2* | intragenic | ODD | Aebi et al., 2016 |
| rs6119729 | 20 | 29876824 | 9,79E-06 | - | *MYLK2* | intragenic | ODD | Aebi et al., 2016 |
| rs6060979 | 20 | 29881183 | 8,07E-06 | - | *MYLK2* | intragenic | ODD | Aebi et al., 2016 |
| rs4518038 | 20 | 29882712 | 8,07E-06 | - | *MYLK2* | intragenic | ODD | Aebi et al., 2016 |
| rs6089093 | 20 | 29885120 | 4,44E-05 | - | *MYLK2* | intragenic | ODD | Aebi et al., 2016 |
| rs17093657 | 20 | 29892555 | 8,07E-06 | - | *FOXS1* | 3,2 | ODD | Aebi et al., 2016 |
| rs6060989 | 20 | 29893424 | 8,07E-06 | - | *FOXS1* | 2,3 | ODD | Aebi et al., 2016 |
| rs6060992 | 20 | 29894916 | 6,26E-06 | - | *FOXS1* | 0,8 | ODD | Aebi et al., 2016 |
| rs6089094 | 20 | 29895432 | 6,26E-06 | - | *FOXS1* | 0,3 | ODD | Aebi et al., 2016 |
| rs6121246 | 20 | 29897013 | 1,47E-05 | - | *FOXS1* | intragenic | ODD | Aebi et al., 2016 |
| rs2424879 | 20 | 30423365 | 2,94E-04 | *-* | *ASXL1* | intragenic | Aggressive behavior | Pappa et al., 2016 |
| rs6119363 | 20 | 31340171 | 1.1E-05 | - | *C20orf114* | intragenic | Antisocial behavior | Tielbeek et al., 2012 |
| rs6028516 | 20 | 37624020 | 2,66E-05 | - | *-* | - | Aggressive behavior | Brevik et al., 2016 |
| rs6101610 | 20 | 37625366 | 2,60E-05 | - | *-* | - | Aggressive behavior | Brevik et al., 2016 |
| rs6028520 | 20 | 37627786 | 2,92E-05 | - | *-* | - | Aggressive behavior | Brevik et al., 2016 |
| rs6028521 | 20 | 37627787 | 2,94E-05 | - | *-* | - | Aggressive behavior | Brevik et al., 2016 |
| rs6028522 | 20 | 37627867 | 3,50E-05 | - | *-* | - | Aggressive behavior | Brevik et al., 2016 |
| rs6016111 | 20 | 37634119 | 4,21E-05 | - | *-* | - | Aggressive behavior | Brevik et al., 2016 |
| rs6124910 | 20 | 45042456 | 2,19E-05 | - | *EYA2* | intragenic | Aggressive behavior | Brevik et al., 2016 |
| rs6063215 | 20 | 46210921 | 1,64E-04 | *-* | *-* | - | Aggressive behavior | Pappa et al., 2016 |
| rs6066909 | 20 | 46963320 | 1.2E-05 | - | *ARFGEF2* | 8.4 | Anger (temper) | Mick et al., 2014 |
| rs4810896 | 20 | 46968705 | 8.0E-06 | - | *ARFGEF2* | 3.0 | Anger (temper) | Mick et al., 2014 |
| rs6012564 | 20 | 46975007 | 1.1E-05 | - | *ARFGEF2* | intragenic | Anger (temper) | Mick et al., 2014 |
| rs1883881 | 20 | 46999186 | 1.6E-05 | - | *ARFGEF2* | intragenic | Anger (temper) | Mick et al., 2014 |
| rs3092374 | 20 | 47097351 | 2.4E-05 | - | *CSE1L* | intragenic | Anger (temper) | Mick et al., 2014 |
| rs3092039 | 20 | 47112177 | 1.9E-05 | - | *CSE1L* | intragenic | Anger (temper) | Mick et al., 2014 |
| rs2075679 | 20 | 47134996 | 3.6E-05 | - | *CSE1L* | intragenic | Anger (temper) | Mick et al., 2014 |
| rs6125539 | 20 | 47137217 | 3.8E-05 | - | *CSE1L* | intragenic | Anger (temper) | Mick et al., 2014 |
| rs6125551 | 20 | 47153793 | 1.9E-05 | - | *CSE1L* | 6.9 | Anger (temper) | Mick et al., 2014 |
| rs2295714 | 20 | 47173221 | 3.5E-05 | - | *STAU1* | intragenic | Anger (temper) | Mick et al., 2014 |
| rs6066975 | 20 | 47183784 | 3.3E-05 | - | *STAU1* | intragenic | Anger (temper) | Mick et al., 2014 |
| rs2273653 | 20 | 47204163 | 3.0E-05 | - | *STAU1* | intragenic | Anger (temper) | Mick et al., 2014 |
| rs6019687 | 20 | 47265311 | 2.6E-05 | - | *DDX27* | 4.0 | Anger (temper) | Mick et al., 2014 |
| rs6512575 | 20 | 47266825 | 2.2E-05 | - | *DDX27* | 2.5 | Anger (temper) | Mick et al., 2014 |
| rs6067024 | 20 | 47276553 | 4.6E-05 | - | *DDX27* | intragenic | Anger (temper) | Mick et al., 2014 |
| rs238203 | 20 | 47292329 | 2.1E-05 | - | *DDX27* | intragenic | Anger (temper) | Mick et al., 2014 |
| rs238204 | 20 | 47292422 | 2.7E-05 | - | *DDX27* | intragenic | Anger (temper) | Mick et al., 2014 |
| rs238215 | 20 | 47303913 | 6.4E-06 | - | *ZNFX1* | intragenic | Anger (temper) | Mick et al., 2014 |
| rs237744 | 20 | 47336183 | 2.7E-05 | - | *ZNFX1* | 8.0 | Anger (temper) | Mick et al., 2014 |
| rs119716 | 20 | 47355241 | 4.4E-05 | - | *ZNFX1* | 27.1 | Anger (temper) | Mick et al., 2014 |
| rs6127751 | 20 | 54440295 | 3,38E-04 | *-* | *CASS4* | intragenic | Aggressive behavior | Pappa et al., 2016 |
| rs10854207 | 20 | 54818492 | 2,07E-05 | - | *-* | - | Aggressive behavior | Brevik et al., 2016 |
| rs6069889 | 20 | 54818573 | 3,70E-05 | - | *-* | - | Aggressive behavior | Brevik et al., 2016 |
| rs2235826 | 20 | 55576575 | 4.1E-05 | - | *PCK1* | 1.7 | Anger | Merjonen et al., 2011 |
| rs2277758 | 20 | 57756351 | 4,01E-04 | *-* | *PHACTR3* | intragenic | Aggressive behavior | Pappa et al., 2016 |
| rs6027079 | 20 | 57756748 | 2,17E-04 | *-* | *PHACTR3* | intragenic | Aggressive behavior | Pappa et al., 2016 |
| rs6061345 | 20 | 59762911 | 4,04E-05 | - | *CDH4* | intragenic | ODD | Aebi et al., 2016 |
| rs7264220 | 20 | 62023877 | 4,01E-04 | *-* | *DNAJC5* | intragenic | Aggressive behavior | Pappa et al., 2016 |
| rs10482853 | 21 | 14618186 | 4,88E-04 | *-* | *HSPA13* | 47,1 | Aggressive behavior | Pappa et al., 2016 |
| rs6516992 | 21 | 15060596 | 3,91E-05 | - | *-* | - | Aggressive behavior | Brevik et al., 2016 |
| rs11088618 | 21 | 17420297 | 4.2E-03 | 2.7E-03 | *-* | - | AB+/CU+ | Viding et al., 2010 |
| rs461221 | 21 | 27163871 | 2,72E-04 | *-* | *ADAMTS1* | 24,3 | Aggressive behavior | Pappa et al., 2016 |
| rs468396 | 21 | 27189803 | 3,99E-04 | *-* | *ADAMTS5* | 22,3 | Aggressive behavior | Pappa et al., 2016 |
| rs467691 | 21 | 27208461 | 2,91E-04 | *-* | *ADAMTS5* | 3,7 | Aggressive behavior | Pappa et al., 2016 |
| rs151065 | 21 | 27218195 | 3,51E-04 | *-* | *ADAMTS5* | intragenic | Aggressive behavior | Pappa et al., 2016 |
| rs226794 | 21 | 27224226 | 3,09E-04 | *-* | *ADAMTS5* | intragenic | Aggressive behavior | Pappa et al., 2016 |
| rs2249333 | 21 | 27232045 | 3,85E-04 | *-* | *ADAMTS5* | intragenic | Aggressive behavior | Pappa et al., 2016 |
| rs2249350 | 21 | 27244377 | 1,56E-04 | *-* | *ADAMTS5* | intragenic | Aggressive behavior | Pappa et al., 2016 |
| rs2833145 | 21 | 31179148 | 3.5E-05 | - | *KRTAP11-1* | 3.4 | CBCL-DP | Mick et al., 2011 |
| rs2835702 | 21 | 37646181 | 5.8E-07 | - | *DYRK1A* | 15.6 | Antisocial behavior | Tielbeek et al., 2012 |
| rs12106331 | 21 | 37649147 | 5.7E-07 | - | *DYRK1A* | 12.6 | Antisocial behavior | Tielbeek et al., 2012 |
| rs2835706 | 21 | 37667833 | 4.5E-05 | - | *DYRK1A* | intragenic | Antisocial behavior | Tielbeek et al., 2012 |
| rs2835708 | 21 | 37673734 | 4.9E-05 | - | *DYRK1A* | intragenic | Antisocial behavior | Tielbeek et al., 2012 |
| rs1060578 | 21 | 37675388 | 4.9E-05 | - | *DYRK1A* | intragenic | Antisocial behavior | Tielbeek et al., 2012 |
| rs2835709 | 21 | 37681971 | 4.9E-05 | - | *DYRK1A* | intragenic | Antisocial behavior | Tielbeek et al., 2012 |
| rs2835710 | 21 | 37682106 | 4.8E-05 | - | *DYRK1A* | intragenic | Antisocial behavior | Tielbeek et al., 2012 |
| rs2835719 | 21 | 37706121 | 4.3E-05 | - | *DYRK1A* | intragenic | Antisocial behavior | Tielbeek et al., 2012 |
| rs9976328 | 21 | 37707076 | 2.6E-05 | - | *DYRK1A* | intragenic | Antisocial behavior | Tielbeek et al., 2012 |
| rs2835721 | 21 | 37707169 | 4.3E-05 | - | *DYRK1A* | intragenic | Antisocial behavior | Tielbeek et al., 2012 |
| rs2835725 | 21 | 37713026 | 4.3E-05 | - | *DYRK1A* | intragenic | Antisocial behavior | Tielbeek et al., 2012 |
| rs2835728 | 21 | 37713633 | 4.3E-05 | - | *DYRK1A* | intragenic | Antisocial behavior | Tielbeek et al., 2012 |
| rs2298329 | 21 | 37717143 | 4.3E-05 | - | *DYRK1A* | intragenic | Antisocial behavior | Tielbeek et al., 2012 |
| rs2835739 | 21 | 37727512 | 4.3E-05 | - | *DYRK1A* | intragenic | Antisocial behavior | Tielbeek et al., 2012 |
| rs2835740 | 21 | 37730943 | 4.3E-05 | - | *DYRK1A* | intragenic | Antisocial behavior | Tielbeek et al., 2012 |
| rs2835747 | 21 | 37738200 | 3.3E-05 | - | *DYRK1A* | intragenic | Antisocial behavior | Tielbeek et al., 2012 |
| rs7283089 | 21 | 37739231 | 3.2E-05 | - | *DYRK1A* | intragenic | Antisocial behavior | Tielbeek et al., 2012 |
| rs2835763 | 21 | 37771610 | 2.8E-05 | - | *DYRK1A* | intragenic | Antisocial behavior | Tielbeek et al., 2012 |
| rs2248244 | 21 | 37774231 | 2.8E-05 | - | *DYRK1A* | intragenic | Antisocial behavior | Tielbeek et al., 2012 |
| rs2835771 | 21 | 37783703 | 1.0E-05 | - | *DYRK1A* | intragenic | Antisocial behavior | Tielbeek et al., 2012 |
| rs2835776 | 21 | 37811845 | 4.9E-05 | - | *DYRK1A* | 2.3 | Antisocial behavior | Tielbeek et al., 2012 |
| rs9983361 | 21 | 38215643 | 3,74E-04 | *-* | *KCNJ6* | 5,1 | Aggressive behavior | Pappa et al., 2016 |
| rs2836525 | 21 | 38867719 | 2,99E-04 | *-* | *ERG* | intragenic | Aggressive behavior | Pappa et al., 2016 |
| rs2836527 | 21 | 38869617 | 3,33E-04 | *-* | *ERG* | intragenic | Aggressive behavior | Pappa et al., 2016 |
| rs2836534 | 21 | 38871421 | 4,50E-04 | *-* | *ERG* | intragenic | Aggressive behavior | Pappa et al., 2016 |
| rs2839391 | 21 | 42128428 | 2,72E-05 | *-* | *PRDM15* | intragenic | Aggressive behavior | Pappa et al., 2016 |
| rs2839392 | 21 | 42128578 | 1,77E-04 | *-* | *PRDM15* | intragenic | Aggressive behavior | Pappa et al., 2016 |
| rs16985013 | 22 | 26274404 | 1.4E-05 | - | *-* | - | Anger (reaction) | Mick et al., 2014 |
| rs84510 | 22 | 26368608 | 3.7E-05 | - | *-* | - | Anger | Merjonen et al., 2011 |
| rs2873857 | 22 | 47971162 | 3,84E-05 | - | *-* | - | ODD | Aebi et al., 2016 |
| rs4564035 | 23 | 34321711 | 4.4E-05 | - | *-* | - | Anger | Merjonen et al., 2011 |
| rs6528097 | X | 22139449 | 4.2E-05 | - | *-* | - | Anger (reaction) | Mick et al., 2014 |
| rs5904512 | X | 22140677 | 6.2E-06 | - | *-* | - | Anger (reaction) | Mick et al., 2014 |
| rs5904513 | X | 22140715 | 5.7E-06 | - | *-* | - | Anger (reaction) | Mick et al., 2014 |
| rs5951727 | X | 22141379 | 1.3E-05 | - | *-* | - | Anger (reaction) | Mick et al., 2014 |
| rs2301325 | X | 22143166 | 1.7E-05 | - | *-* | - | Anger (reaction) | Mick et al., 2014 |
| rs3752433 | X | 22149641 | 1.6E-07 | - | *-* | - | Anger (reaction) | Mick et al., 2014 |
| rs2285075 | X | 22151389 | 2.8E-05 | - | *-* | - | Anger (reaction) | Mick et al., 2014 |
| rs2285079 | X | 22153599 | 1.7E-05 | - | *-* | - | Anger (reaction) | Mick et al., 2014 |
| rs5951522 | X | 22180900 | 2.1E-05 | - | *-* | - | Anger (reaction) | Mick et al., 2014 |
| rs543042 | X | 146406079 | 1.5E-05 | - | *-* |  | Anger (temper) | Mick et al., 2014 |
|  |  |  |  |  |  |  |  |  |

SNP: Single Nucleotide Polymorphism; Chr: Chromosome; GWAS: Genome Wide Association Study; CD: Conduct Disorder; ODD: Oppositional Defiand Disorder; AB+/CU+: Antisocial behavior and Callous Unemotional; CBCL-DP; Children Behavior Checklist Dysregulation Profile.

*in a window of 100 kb
