# Supplementary material for: An integrated analysis of genes and functional pathways for aggression in human and rodent models
Source: Mol Psychiatry. 2018 Jun 1;24(11):1655–67. doi: 10.1038/s41380-018-0068-7 (PMC6274606; doi:10.1038/s41380-018-0068-7)
Supplement: Supplementary file 3 — Supplementary Table 2 [file 41380_2018_68_MOESM3_ESM.doc]

**Supplementary Table 2:** SNPs associated in 14 selected GWAS assessing phenotypes not related to the nervous system, used for the control GWAS gene list.

| SNP | Chr | bp | P-value | Closest gene* | Phenotype | GWAS study | PubMed ID |
| --- | --- | --- | --- | --- | --- | --- | --- |
|  |  |  |  |  |  |  |  |
| rs3765350 | 1 | 22319903 | 7,00E-10 | *WNT4* | Bone mineral density (paediatric) | Kemp et al., 2014 | 24945404 |
| rs2235529 | 1 | 22323074 | 1,00E-08 | *WNT4* | Bone mineral density (paediatric) | Kemp et al., 2014 | 24945404 |
| rs3820282 | 1 | 22340802 | 2,00E-08 | *WNT4* | Epithelial ovarian cancer | Kuchenbaecker et al., 2015 | 25581431 |
| rs3920498 | 1 | 22365474 | 1,00E-10 | *WNT4* | Bone mineral density (paediatric) | Kemp et al., 2014 | 24945404 |
| rs72647484 | 1 | 22460315 | 1,00E-08 | *-* | Colorectal cancer | Al-Tassan et al., 2015 | 25990418 |
| rs34920465 | 1 | 22572938 | 3,00E-13 | *ZBTB40* | Bone mineral density | Zhang et al., 2014 | 24249740 |
| rs12039431 | 1 | 37854709 | 1,00E-11 | *RSPO1* | Epithelial ovarian cancer | Kuchenbaecker et al., 2015 | 25581431 |
| rs1430740 | 1 | 68430098 | 1,00E-11 | *GPR177* | Bone mineral density | Zhang et al., 2014 | 24249740 |
| rs2153977 | 1 | 113881594 | 4,00E-07 | *MAGI3* | Type 1 diabetes | Tomer et al., 2015 | 25936594 |
| rs2476601 | 1 | 114179091 | 7,00E-20 | *PTPN22* | Type 1 diabetes | Tomer et al., 2015 | 25936594 |
| rs2358994 | 1 | 114230984 | 2,00E-11 | *BCL2L15* | Type 1 diabetes | Tomer et al., 2015 | 25936594 |
| rs3001089 | 1 | 161767335 | 5,00E-07 | *-* | Chronic obstructive pulmonary disease | Cho et al., 2014 | 24621683 |
| rs4524 | 1 | 167778379 | 3,00E-11 | *F5* | Venous thromboembolism | Germain et al., 2015 | 25772935 |
| rs6025 | 1 | 167785673 | 1,00E-96 | *F5* | Venous thromboembolism | Germain et al., 2015 | 25772935 |
| rs10914144 | 1 | 170216373 | 2,00E-08 | *DNM3* | Platelet count or volume | Shameer et al., 2014 | 24026423 |
| rs9660992 | 1 | 203516073 | 3,00E-13 | *TMCC2* | Platelet count or volume | Shameer et al., 2014 | 24026423 |
| rs4846480 | 1 | 216665092 | 8,00E-09 | *TGFB2* | Chronic obstructive pulmonary disease | Cho et al., 2014 | 24621683 |
| rs11118883 | 1 | 220127645 | 5,00E-06 | *-* | Colorectal cancer | Al-Tassan et al., 2015 | 25990418 |
| rs7569716 | 2 | 22422218 | 1,00E-07 | *-* | Chronic obstructive pulmonary disease | Cho et al., 2014 | 24621683 |
| rs649729 | 2 | 31317889 | 4,00E-06 | *EHD3* | Platelet count or volume | Shameer et al., 2014 | 24026423 |
| rs2041840 | 2 | 37320768 | 9,00E-06 | *C2orf56* | Chronic lymphocytic leukemia | Speedy et al., 2013 | 24292274 |
| rs2430386 | 2 | 63031615 | 9,00E-12 | *EHBP1* | Prostate cancer | Berndt et al., 2015 | 25939597 |
| rs11884776 | 2 | 73600431 | 6,00E-17 | *ALMS1* | Urinary metabolites (H-NMR features) | Rueedi et al., 2014 | 24586186 |
| rs6546847 | 2 | 73638866 | 5,00E-161 | *ALMS1* | Urinary metabolites (H-NMR features) | Rueedi et al., 2014 | 24586186 |
| rs1439287 | 2 | 111588368 | 5,00E-15 | *BCL2L11* | Chronic lymphocytic leukemia | Speedy et al., 2013 | 24292274 |
| rs6726821 | 2 | 166286360 | 4,00E-10 | *GALNT3* | Bone mineral density (paediatric) | Kemp et al., 2014 | 24945404 |
| rs1346004 | 2 | 166309292 | 5,00E-07 | *GALNT3* | Bone mineral density (paediatric) | Kemp et al., 2014 | 24945404 |
| rs2072590 | 2 | 176750879 | 9,00E-14 | *HOXD3* | Epithelial ovarian cancer | Kuchenbaecker et al., 2015 | 25581431 |
| rs1509569 | 2 | 210840660 | 3,00E-18 | *MYL1* | Urinary metabolites (H-NMR features) | Rueedi et al., 2014 | 24586186 |
| rs13397985 | 2 | 230799467 | 5,00E-13 | *SP140* | Chronic lymphocytic leukemia | Speedy et al., 2013 | 24292274 |
| rs1354034 | 3 | 56824789 | 9,00E-34 | *ARHGEF3* | Platelet count or volume | Shameer et al., 2014 | 24026423 |
| rs2687201 | 3 | 71011620 | 5,00E-09 | *FOXP1* | Digestive system disease (Barrett's esophagus and/or esophageal adenocarcinoma) | Levine et al., 2013 | 24121790 |
| rs17023900 | 3 | 87217490 | 1,00E-11 | *VGLL3* | Prostate cancer | Berndt et al., 2015 | 25939597 |
| rs2681416 | 3 | 123300303 | 2,00E-07 | *CD86* | Follicular lymphoma | Skibola et al., 2014 | 25279986 |
| rs7651446 | 3 | 157889691 | 6,00E-51 | *TIPARP* | Epithelial ovarian cancer | Kuchenbaecker et al., 2015 | 25581431 |
| rs7632500 | 3 | 169208043 | 1,00E-06 | *GOLIM4* | Digestive system disease (Barrett's esophagus and/or esophageal adenocarcinoma) | Levine et al., 2013 | 24121790 |
| rs10936599 | 3 | 170974795 | 2,00E-09 | *MYNN* | Chronic lymphocytic leukemia | Speedy et al., 2013 | 24292274 |
| rs71277158 | 3 | 171481910 | 8,00E-14 | *PRKCI* | Prostate cancer | Berndt et al., 2015 | 25939597 |
| rs78943174 | 3 | 176735430 | 4,00E-08 | *NAALADL2* | Prostate cancer | Berndt et al., 2015 | 25939597 |
| rs6444305 | 3 | 189782596 | 1,00E-10 | *LPP* | Follicular lymphoma | Skibola et al., 2014 | 25279986 |
| rs6827815 | 4 | 989445 | 5,00E-12 | *IDUA* | Bone mineral density | Zhang et al., 2014 | 24249740 |
| rs11734132 | 4 | 6942420 | 6,00E-07 | *KIAA0232* | Platelet count or volume | Shameer et al., 2014 | 24026423 |
| rs10034692 | 4 | 75638651 | 2,00E-10 | *AREG* | Mammographic density | Lindstrom et al., 2014 | 25342443 |
| rs12642133 | 4 | 75762313 | 8,00E-10 | *AREG* | Mammographic density | Lindstrom et al., 2014 | 25342443 |
| rs4610302 | 4 | 88619134 | 4,00E-06 | *SPARCL1* | Digestive system disease (Barrett's esophagus and/or esophageal adenocarcinoma) | Levine et al., 2013 | 24121790 |
| rs6532023 | 4 | 88992873 | 3,00E-06 | *MEPE* | Bone mineral density (paediatric) | Kemp et al., 2014 | 24945404 |
| rs1463104 | 4 | 89018734 | 2,00E-09 | *MEPE* | Bone mineral density | Zhang et al., 2014 | 24249740 |
| rs4416442 | 4 | 90085736 | 9,00E-15 | *FAM13A1* | Chronic obstructive pulmonary disease | Cho et al., 2014 | 24621683 |
| rs7679673 | 4 | 106280983 | 4,00E-09 | *TET2* | Prostate cancer | Berndt et al., 2015 | 25939597 |
| rs6858698 | 4 | 114903293 | 3,00E-09 | *CAMK2D* | Chronic lymphocytic leukemia | Speedy et al., 2013 | 24292274 |
| rs17329882 | 4 | 120169408 | 2,00E-08 | *SYNPO2* | Epithelial ovarian cancer | Kuchenbaecker et al., 2015 | 25581431 |
| rs7679475 | 4 | 122533490 | 4,00E-10 | *GPR103* | Type 1 diabetes | Tomer et al., 2015 | 25936594 |
| rs7655841 | 4 | 130110240 | 7,00E-07 | *SCLT1* | Chronic obstructive pulmonary disease | Cho et al., 2014 | 24621683 |
| rs13141641 | 4 | 145725906 | 4,00E-15 | *HHIP* | Chronic obstructive pulmonary disease | Cho et al., 2014 | 24621683 |
| rs2066865 | 4 | 155744726 | 1,00E-16 | *FGG* | Venous thromboembolism | Germain et al., 2015 | 25772935 |
| rs4253417 | 4 | 187435999 | 1,00E-23 | *F11* | Venous thromboembolism | Germain et al., 2015 | 25772935 |
| rs10069690 | 5 | 1332790 | 9,00E-09 | *TERT* | Epithelial ovarian cancer | Kuchenbaecker et al., 2015 | 25581431 |
| rs7725218 | 5 | 1335414 | 3,00E-11 | *TERT* | Prostate cancer | Berndt et al., 2015 | 25939597 |
| rs31490 | 5 | 1397458 | 2,00E-07 | *CLPTM1L* | Chronic lymphocytic leukemia | Speedy et al., 2013 | 24292274 |
| rs7717823 | 5 | 35036552 | 3,00E-29 | *AGXT2* | Urinary metabolites (H-NMR features) | Rueedi et al., 2014 | 24586186 |
| rs468327 | 5 | 35038869 | 4,00E-15 | *AGXT2* | Urinary metabolites (H-NMR features) | Rueedi et al., 2014 | 24586186 |
| rs37369 | 5 | 35072872 | 1,00E-63 | *AGXT2* | Urinary metabolites (H-NMR features) | Rueedi et al., 2014 | 24586186 |
| rs40200 | 5 | 35081502 | 9,00E-37 | *AGXT2* | Urinary metabolites (H-NMR features) | Rueedi et al., 2014 | 24586186 |
| rs6449586 | 5 | 50670635 | 1,00E-06 | *ISL1* | Digestive system disease (Barrett's esophagus and/or esophageal adenocarcinoma) | Levine et al., 2013 | 24121790 |
| rs35148638 | 5 | 86646745 | 6,00E-09 | *RASA1* | Prostate cancer | Berndt et al., 2015 | 25939597 |
| rs6894139 | 5 | 88363538 | 7,00E-18 | *-* | Bone mineral density | Zhang et al., 2014 | 24249740 |
| rs202110856 | 5 | 96155629 | 7,00E-08 | *ERAP1* | Colorectal cancer | Al-Tassan et al., 2015 | 25990418 |
| rs186749 | 5 | 122482204 | 3,00E-09 | *PPIC* | Mammographic density | Lindstrom et al., 2014 | 25342443 |
| rs872071 | 6 | 356064 | 3,00E-16 | *IRF4* | Chronic lymphocytic leukemia | Speedy et al., 2013 | 24292274 |
| rs1294438 | 6 | 6697058 | 1,00E-06 | *LY86* | Mammographic density | Lindstrom et al., 2014 | 25342443 |
| rs12198220 | 6 | 21438272 | 2,00E-07 | *CDKAL1* | Prostate cancer | Berndt et al., 2015 | 25939597 |
| rs115344852 | 6 | 28594077 | 3,00E-08 | *GPX6* | Epithelial ovarian cancer | Kuchenbaecker et al., 2015 | 25581431 |
| rs2523989 | 6 | 30186254 | 2,00E-08 | *TRIM31* | Type 1 diabetes | Tomer et al., 2015 | 25936594 |
| rs886424 | 6 | 30889981 | 3,00E-14 | *IER3* | Type 1 diabetes | Tomer et al., 2015 | 25936594 |
| rs2251396 | 6 | 31472686 | 1,00E-22 | *MICA* | Type 1 diabetes | Tomer et al., 2015 | 25936594 |
| rs2857595 | 6 | 31676448 | 2,00E-23 | *NCR3* | Type 1 diabetes | Tomer et al., 2015 | 25936594 |
| rs1270942 | 6 | 32026839 | 5,00E-25 | *CFB* | Type 1 diabetes | Tomer et al., 2015 | 25936594 |
| rs926070 | 6 | 32365544 | 4,00E-08 | *C6orf10* | Chronic lymphocytic leukemia | Speedy et al., 2013 | 24292274 |
| rs1980493 | 6 | 32471193 | 2,00E-20 | *BTNL2* | Type 1 diabetes | Tomer et al., 2015 | 25936594 |
| rs12195582 | 6 | 32552522 | 5,00E-100 | *HLA-DRA* | Follicular lymphoma | Skibola et al., 2014 | 25279986 |
| rs1015166 | 6 | 32906709 | 4,00E-22 | *TAP2* | Type 1 diabetes | Tomer et al., 2015 | 25936594 |
| rs210134 | 6 | 33648187 | 3,00E-06 | *BAK1* | Chronic lymphocytic leukemia | Speedy et al., 2013 | 24292274 |
| rs210134 | 6 | 33648187 | 6,00E-08 | *BAK1* | Platelet count or volume | Shameer et al., 2014 | 24026423 |
| rs2342002 | 6 | 62009819 | 9,00E-07 | *-* | Digestive system disease (Barrett's esophagus and/or esophageal adenocarcinoma) | Levine et al., 2013 | 24121790 |
| rs4418209 | 6 | 126861392 | 1,00E-06 | *-* | Bone mineral density (paediatric) | Kemp et al., 2014 | 24945404 |
| rs2130604 | 6 | 126862254 | 3,00E-11 | *-* | Bone mineral density (paediatric) | Kemp et al., 2014 | 24945404 |
| rs1262476 | 6 | 127028689 | 3,00E-09 | *-* | Bone mineral density (paediatric) | Kemp et al., 2014 | 24945404 |
| rs13204965 | 6 | 127208765 | 2,00E-06 | *-* | Bone mineral density (paediatric) | Kemp et al., 2014 | 24945404 |
| rs3012465 | 6 | 133392629 | 8,00E-17 | *-* | Bone mineral density (paediatric) | Kemp et al., 2014 | 24945404 |
| rs9399137 | 6 | 135460711 | 8,00E-10 | *HBS1L* | Platelet count or volume | Shameer et al., 2014 | 24026423 |
| rs1871859 | 6 | 151940199 | 9,00E-13 | *C6orf97* | Bone mineral density | Zhang et al., 2014 | 24249740 |
| rs12665607 | 6 | 151988322 | 2,00E-08 | *C6orf97* | Mammographic density | Lindstrom et al., 2014 | 25342443 |
| rs2236256 | 6 | 154520132 | 2,00E-10 | *OPRM1* | Chronic lymphocytic leukemia | Speedy et al., 2013 | 24292274 |
| rs7758229 | 6 | 160760242 | 4,00E-12 | *SLC22A3* | Prostate cancer | Berndt et al., 2015 | 25939597 |
| rs17172185 | 7 | 43253364 | 5,00E-06 | *HECW1* | Digestive system disease (Barrett's esophagus and/or esophageal adenocarcinoma) | Levine et al., 2013 | 24121790 |
| rs2190016 | 7 | 46911332 | 3,00E-06 | *-* | Mammographic density | Lindstrom et al., 2014 | 25342443 |
| rs798336 | 7 | 77752279 | 2,00E-06 | *MAGI2* | Mammographic density | Lindstrom et al., 2014 | 25342443 |
| rs10429035 | 7 | 95957417 | 4,00E-12 | *-* | Bone mineral density | Zhang et al., 2014 | 24249740 |
| rs342293 | 7 | 106159455 | 5,00E-22 | *FLJ36031* | Platelet count or volume | Shameer et al., 2014 | 24026423 |
| rs13223036 | 7 | 120534544 | 2,00E-28 | *C7orf58* | Bone mineral density (paediatric) | Kemp et al., 2014 | 24945404 |
| rs798943 | 7 | 120546135 | 1,00E-37 | *C7orf58* | Bone mineral density (paediatric) | Kemp et al., 2014 | 24945404 |
| rs2908004 | 7 | 120757005 | 3,00E-11 | *WNT16* | Bone mineral density (paediatric) | Kemp et al., 2014 | 24945404 |
| rs10242100 | 7 | 120770579 | 2,00E-10 | *WNT16* | Bone mineral density | Zhang et al., 2014 | 24249740 |
| rs7776725 | 7 | 120820357 | 2,00E-15 | *FAM3C* | Bone mineral density (paediatric) | Kemp et al., 2014 | 24945404 |
| rs17246404 | 7 | 124249897 | 3,00E-08 | *POT1* | Chronic lymphocytic leukemia | Speedy et al., 2013 | 24292274 |
| rs11771429 | 7 | 152902810 | 7,00E-06 | *-* | Digestive system disease (Barrett's esophagus and/or esophageal adenocarcinoma) | Levine et al., 2013 | 24121790 |
| rs4921914 | 8 | 18316718 | 4,00E-32 | *NAT2* | Urinary metabolites (H-NMR features) | Rueedi et al., 2014 | 24586186 |
| rs7816345 | 8 | 36965267 | 2,00E-23 | *KCNU1* | Mammographic density | Lindstrom et al., 2014 | 25342443 |
| rs11782652 | 8 | 82816199 | 3,00E-10 | *CHMP4C* | Epithelial ovarian cancer | Kuchenbaecker et al., 2015 | 25581431 |
| rs4602861 | 8 | 106659882 | 5,00E-07 | *ZFPM2* | Venous thromboembolism | Germain et al., 2015 | 25772935 |
| rs76316943 | 8 | 117917488 | 2,00E-11 | *RAD21* | Colorectal cancer | Al-Tassan et al., 2015 | 25990418 |
| rs4424296 | 8 | 120082457 | 9,00E-14 | *TNFRSF11B* | Bone mineral density | Zhang et al., 2014 | 24249740 |
| rs2450083 | 8 | 120132723 | 2,00E-11 | *COLEC10* | Bone mineral density (paediatric) | Kemp et al., 2014 | 24945404 |
| rs16901979 | 8 | 128194098 | 5,00E-09 | *-* | Prostate cancer | Berndt et al., 2015 | 25939597 |
| rs2466024 | 8 | 128257201 | 3,00E-06 | *-* | Chronic lymphocytic leukemia | Speedy et al., 2013 | 24292274 |
| rs6983267 | 8 | 128482487 | 4,00E-10 | *-* | Prostate cancer | Berndt et al., 2015 | 25939597 |
| rs7014346 | 8 | 128493974 | 6,00E-15 | *-* | Colorectal cancer | Al-Tassan et al., 2015 | 25990418 |
| rs4242382 | 8 | 128586755 | 1,00E-34 | *-* | Prostate cancer | Berndt et al., 2015 | 25939597 |
| rs13254990 | 8 | 129145633 | 1,00E-08 | *-* | Follicular lymphoma | Skibola et al., 2014 | 25279986 |
| rs10088218 | 8 | 129613131 | 1,00E-20 | *-* | Epithelial ovarian cancer | Kuchenbaecker et al., 2015 | 25581431 |
| rs423955 | 9 | 4782339 | 1,00E-09 | *RCL1* | Platelet count or volume | Shameer et al., 2014 | 24026423 |
| rs3814113 | 9 | 16905021 | 6,00E-50 | *BNC2* | Epithelial ovarian cancer | Kuchenbaecker et al., 2015 | 25581431 |
| rs10814275 | 9 | 35748578 | 6,00E-07 | *PC-3* | Mammographic density | Lindstrom et al., 2014 | 25342443 |
| rs11789015 | 9 | 95755849 | 1,00E-09 | *BARX1* | Digestive system disease (Barrett's esophagus and/or esophageal adenocarcinoma) | Levine et al., 2013 | 24121790 |
| rs6479527 | 9 | 95898232 | 2,00E-06 | *PTPDC1* | Digestive system disease (Barrett's esophagus and/or esophageal adenocarcinoma) | Levine et al., 2013 | 24121790 |
| rs1110403 | 9 | 109423404 | 7,00E-06 | *-* | Mammographic density | Lindstrom et al., 2014 | 25342443 |
| rs7466269 | 9 | 132453905 | 2,00E-08 | *FUBP3* | Bone mineral density (paediatric) | Kemp et al., 2014 | 24945404 |
| rs8176749 | 9 | 135121009 | 4,00E-12 | *ABO* | Urinary metabolites (H-NMR features) | Rueedi et al., 2014 | 24586186 |
| rs579459 | 9 | 135143989 | 2,00E-32 | *ABO* | Urinary metabolites (H-NMR features) | Rueedi et al., 2014 | 24586186 |
| rs11255841 | 10 | 8779586 | 4,00E-13 | *-* | Colorectal cancer | Al-Tassan et al., 2015 | 25990418 |
| rs10904849 | 10 | 17037272 | 7,00E-08 | *CUBN* | Colorectal cancer | Al-Tassan et al., 2015 | 25990418 |
| rs1243180 | 10 | 21955625 | 1,00E-09 | *MLLT10* | Epithelial ovarian cancer | Kuchenbaecker et al., 2015 | 25581431 |
| rs3905706 | 10 | 28519948 | 3,00E-06 | *MPP7* | Bone mineral density (paediatric) | Kemp et al., 2014 | 24945404 |
| rs3905706 | 10 | 28519948 | 8,00E-06 | *MPP7* | Bone mineral density | Zhang et al., 2014 | 24249740 |
| rs10993994 | 10 | 51219502 | 1,00E-15 | *MSMB* | Prostate cancer | Berndt et al., 2015 | 25939597 |
| rs14168 | 10 | 53125568 | 3,00E-06 | *CSTF2T* | Mammographic density | Lindstrom et al., 2014 | 25342443 |
| rs12220488 | 10 | 63889006 | 8,00E-07 | *ZNF365* | Mammographic density | Lindstrom et al., 2014 | 25342443 |
| rs10509168 | 10 | 63927834 | 1,00E-09 | *ZNF365* | Mammographic density | Lindstrom et al., 2014 | 25342443 |
| rs10995190 | 10 | 63948688 | 1,00E-16 | *ZNF365* | Mammographic density | Lindstrom et al., 2014 | 25342443 |
| rs10761731 | 10 | 64697616 | 2,00E-06 | *JMJD1C* | Platelet count or volume | Shameer et al., 2014 | 24026423 |
| rs7075195 | 10 | 64720665 | 3,00E-18 | *JMJD1C* | Platelet count or volume | Shameer et al., 2014 | 24026423 |
| rs78707713 | 10 | 70915282 | 2,00E-16 | *TSPAN15* | Venous thromboembolism | Germain et al., 2015 | 25772935 |
| rs7904985 | 10 | 88106459 | 6,00E-06 | *GRID1* | Digestive system disease (Barrett's esophagus and/or esophageal adenocarcinoma) | Levine et al., 2013 | 24121790 |
| rs1800682 | 10 | 90739943 | 2,00E-08 | *FAS* | Chronic lymphocytic leukemia | Speedy et al., 2013 | 24292274 |
| rs4345897 | 10 | 100137050 | 2,00E-19 | *C10orf33* | Urinary metabolites (H-NMR features) | Rueedi et al., 2014 | 24586186 |
| rs4539242 | 10 | 100138048 | 2,00E-20 | *C10orf33* | Urinary metabolites (H-NMR features) | Rueedi et al., 2014 | 24586186 |
| rs2147896 | 10 | 100138166 | 3,00E-164 | *C10orf33* | Urinary metabolites (H-NMR features) | Rueedi et al., 2014 | 24586186 |
| rs17455577 | 10 | 100155987 | 3,00E-22 | *C10orf33* | Urinary metabolites (H-NMR features) | Rueedi et al., 2014 | 24586186 |
| rs11190164 | 10 | 101341694 | 8,00E-07 | *SLC25A28* | Colorectal cancer | Al-Tassan et al., 2015 | 25990418 |
| rs10884482 | 10 | 109370254 | 6,00E-08 | *-* | Mammographic density | Lindstrom et al., 2014 | 25342443 |
| rs11602954 | 11 | 192856 | 5,00E-12 | *BET1L* | Platelet count or volume | Shameer et al., 2014 | 24026423 |
| rs3817198 | 11 | 1865582 | 1,00E-10 | *LSP1* | Mammographic density | Lindstrom et al., 2014 | 25342443 |
| rs11022157 | 11 | 2279405 | 3,00E-06 | *TSPAN32* | Chronic lymphocytic leukemia | Speedy et al., 2013 | 24292274 |
| rs7108738 | 11 | 15666660 | 1,00E-15 | *-* | Bone mineral density | Zhang et al., 2014 | 24249740 |
| rs10160456 | 11 | 27293280 | 8,00E-06 | *CCDC34* | Bone mineral density (paediatric) | Kemp et al., 2014 | 24945404 |
| rs10835187 | 11 | 27462253 | 2,00E-17 | *LIN7C* | Bone mineral density (paediatric) | Kemp et al., 2014 | 24945404 |
| rs1799963 | 11 | 46717631 | 2,00E-09 | *F2* | Venous thromboembolism | Germain et al., 2015 | 25772935 |
| rs525592 | 11 | 67951680 | 3,00E-11 | *LRP5* | Bone mineral density | Zhang et al., 2014 | 24249740 |
| rs12272917 | 11 | 68019946 | 1,00E-10 | *SAPS3* | Bone mineral density (paediatric) | Kemp et al., 2014 | 24945404 |
| rs7929962 | 11 | 68742159 | 2,00E-13 | *MYEOV* | Prostate cancer | Berndt et al., 2015 | 25939597 |
| rs3824999 | 11 | 74023198 | 8,00E-11 | *POLD3* | Colorectal cancer | Al-Tassan et al., 2015 | 25990418 |
| rs626750 | 11 | 102226155 | 3,00E-09 | *MMP3* | Chronic obstructive pulmonary disease | Cho et al., 2014 | 24621683 |
| rs3802842 | 11 | 110676919 | 2,00E-06 | *FLJ45803* | Colorectal cancer | Al-Tassan et al., 2015 | 25990418 |
| rs4938573 | 11 | 118247052 | 6,00E-20 | *CXCR5* | Follicular lymphoma | Skibola et al., 2014 | 25279986 |
| rs735665 | 11 | 122866607 | 4,00E-24 | *GRAMD1B* | Chronic lymphocytic leukemia | Speedy et al., 2013 | 24292274 |
| rs4937362 | 11 | 127997949 | 7,00E-11 | *FLI1* | Follicular lymphoma | Skibola et al., 2014 | 25279986 |
| rs11836164 | 12 | 26337892 | 1,00E-07 | *ITPR2* | Mammographic density | Lindstrom et al., 2014 | 25342443 |
| rs4420311 | 12 | 27875457 | 3,00E-08 | *KLHDC5* | Bone mineral density (paediatric) | Kemp et al., 2014 | 24945404 |
| rs4768903 | 12 | 49331716 | 4,00E-08 | *DIP2B* | Colorectal cancer | Al-Tassan et al., 2015 | 25990418 |
| rs10506328 | 12 | 52973499 | 2,00E-09 | *NFE2* | Platelet count or volume | Shameer et al., 2014 | 24026423 |
| rs11174267 | 12 | 60672867 | 8,00E-07 | *FAM19A2* | Chronic obstructive pulmonary disease | Cho et al., 2014 | 24621683 |
| rs703556 | 12 | 101536024 | 4,00E-10 | *-* | Mammographic density | Lindstrom et al., 2014 | 25342443 |
| rs3184504 | 12 | 110368991 | 5,00E-11 | *SH2B3* | Platelet count or volume | Shameer et al., 2014 | 24026423 |
| rs10774740 | 12 | 113150585 | 3,00E-10 | *-* | Prostate cancer | Berndt et al., 2015 | 25939597 |
| rs3916 | 12 | 119661655 | 2,00E-22 | *ACADS* | Urinary metabolites (H-NMR features) | Rueedi et al., 2014 | 24586186 |
| rs7314056 | 12 | 120827347 | 4,00E-16 | *PSMD9* | Urinary metabolites (H-NMR features) | Rueedi et al., 2014 | 24586186 |
| rs7961894 | 12 | 120849966 | 6,00E-38 | *WDR66* | Platelet count or volume | Shameer et al., 2014 | 24026423 |
| rs9533090 | 13 | 41849449 | 3,00E-07 | *AKAP11* | Bone mineral density | Zhang et al., 2014 | 24249740 |
| rs9533095 | 13 | 41867049 | 2,00E-15 | *AKAP11* | Bone mineral density | Zhang et al., 2014 | 24249740 |
| rs9525638 | 13 | 42026577 | 3,00E-09 | *TNFSF11* | Bone mineral density (paediatric) | Kemp et al., 2014 | 24945404 |
| rs17536328 | 13 | 42041029 | 8,00E-09 | *TNFSF11* | Bone mineral density (paediatric) | Kemp et al., 2014 | 24945404 |
| rs2148072 | 13 | 42060063 | 2,00E-06 | *TNFSF11* | Bone mineral density (paediatric) | Kemp et al., 2014 | 24945404 |
| rs1546939 | 14 | 25740765 | 9,00E-06 | *-* | Mammographic density | Lindstrom et al., 2014 | 25342443 |
| rs35107139 | 14 | 53488856 | 2,00E-06 | *BMP4* | Colorectal cancer | Al-Tassan et al., 2015 | 25990418 |
| rs227425 | 14 | 69526452 | 4,00E-13 | *SMOC1* | Bone mineral density | Zhang et al., 2014 | 24249740 |
| rs754388 | 14 | 92185163 | 1,00E-10 | *RIN3* | Bone mineral density (paediatric) | Kemp et al., 2014 | 24945404 |
| rs754388 | 14 | 92185163 | 5,00E-09 | *RIN3* | Chronic obstructive pulmonary disease | Cho et al., 2014 | 24621683 |
| rs2293582 | 15 | 30797704 | 3,00E-11 | *GREM1* | Colorectal cancer | Al-Tassan et al., 2015 | 25990418 |
| rs8023845 | 15 | 38201408 | 2,00E-06 | *BMF* | Chronic lymphocytic leukemia | Speedy et al., 2013 | 24292274 |
| rs16976734 | 15 | 54169587 | 4,00E-07 | *RFXDC2* | Chronic lymphocytic leukemia | Speedy et al., 2013 | 24292274 |
| rs3784262 | 15 | 56040398 | 4,00E-07 | *ALDH1A2* | Digestive system disease (Barrett's esophagus and/or esophageal adenocarcinoma) | Levine et al., 2013 | 24121790 |
| rs7176508 | 15 | 67806044 | 8,00E-18 | *-* | Chronic lymphocytic leukemia | Speedy et al., 2013 | 24292274 |
| rs12914385 | 15 | 76685778 | 3,00E-16 | *CHRNA3* | Chronic obstructive pulmonary disease | Cho et al., 2014 | 24621683 |
| rs79900961 | 16 | 9205313 | 5,00E-08 | *C16orf72* | Colorectal cancer | Al-Tassan et al., 2015 | 25990418 |
| rs8044477 | 16 | 57534092 | 1,00E-08 | *-* | Epithelial ovarian cancer | Kuchenbaecker et al., 2015 | 25581431 |
| rs12597458 | 16 | 70588471 | 1,00E-07 | *DHODH* | Prostate cancer | Berndt et al., 2015 | 25939597 |
| rs1044873 | 16 | 84513172 | 1,00E-09 | *IRF8* | Chronic lymphocytic leukemia | Speedy et al., 2013 | 24292274 |
| rs2178146 | 16 | 85021196 | 1,00E-06 | *FOXF1* | Digestive system disease (Barrett's esophagus and/or esophageal adenocarcinoma) | Levine et al., 2013 | 24121790 |
| rs16941835 | 16 | 85253221 | 5,00E-08 | *FOXL1* | Colorectal cancer | Al-Tassan et al., 2015 | 25990418 |
| rs71390846 | 16 | 85272216 | 2,00E-10 | *FOXL1* | Bone mineral density | Zhang et al., 2014 | 24249740 |
| rs9900280 | 17 | 24793724 | 1,00E-10 | *TAOK1* | Platelet count or volume | Shameer et al., 2014 | 24026423 |
| rs757210 | 17 | 33170628 | 2,00E-08 | *HNF1B* | Epithelial ovarian cancer | Kuchenbaecker et al., 2015 | 25581431 |
| rs8064454 | 17 | 33175699 | 8,00E-29 | *HNF1B* | Prostate cancer | Berndt et al., 2015 | 25939597 |
| rs183211 | 17 | 42143493 | 2,00E-13 | *NSF* | Epithelial ovarian cancer | Kuchenbaecker et al., 2015 | 25581431 |
| rs9303542 | 17 | 43766499 | 5,00E-15 | *SKAP1* | Epithelial ovarian cancer | Kuchenbaecker et al., 2015 | 25581431 |
| rs17765344 | 17 | 66618469 | 4,00E-21 | *-* | Prostate cancer | Berndt et al., 2015 | 25939597 |
| rs3751913 | 17 | 77998841 | 2,00E-07 | *C17orf62* | Follicular lymphoma | Skibola et al., 2014 | 25279986 |
| rs4800353 | 18 | 17908135 | 3,00E-07 | *GATA6* | Digestive system disease (Barrett's esophagus and/or esophageal adenocarcinoma) | Levine et al., 2013 | 24121790 |
| rs11082304 | 18 | 18974971 | 5,00E-06 | *CABLES1* | Platelet count or volume | Shameer et al., 2014 | 24026423 |
| rs11082438 | 18 | 41119208 | 4,00E-07 | *-* | Follicular lymphoma | Skibola et al., 2014 | 25279986 |
| rs7226855 | 18 | 44708046 | 4,00E-23 | *SMAD7* | Colorectal cancer | Al-Tassan et al., 2015 | 25990418 |
| rs884205 | 18 | 58205837 | 2,00E-08 | *TNFRSF11A* | Bone mineral density (paediatric) | Kemp et al., 2014 | 24945404 |
| rs17749561 | 18 | 58934191 | 8,00E-10 | *BCL2* | Follicular lymphoma | Skibola et al., 2014 | 25279986 |
| rs2288904 | 19 | 10603170 | 3,00E-15 | *SLC44A2* | Venous thromboembolism | Germain et al., 2015 | 25772935 |
| rs8170 | 19 | 17250704 | 5,00E-14 | *C19orf62* | Epithelial ovarian cancer | Kuchenbaecker et al., 2015 | 25581431 |
| rs10419226 | 19 | 18664172 | 4,00E-10 | *CRTC1* | Digestive system disease (Barrett's esophagus and/or esophageal adenocarcinoma) | Levine et al., 2013 | 24121790 |
| rs8101881 | 19 | 38056468 | 1,00E-33 | *SLC7A9* | Urinary metabolites (H-NMR features) | Rueedi et al., 2014 | 24586186 |
| rs2927438 | 19 | 49933947 | 2,00E-06 | *BCL3* | Digestive system disease (Barrett's esophagus and/or esophageal adenocarcinoma) | Levine et al., 2013 | 24121790 |
| rs2287921 | 19 | 53920084 | 3,00E-36 | *RASIP1* | Urinary metabolites (H-NMR features) | Rueedi et al., 2014 | 24586186 |
| rs281408 | 19 | 53925218 | 4,00E-32 | *RASIP1* | Urinary metabolites (H-NMR features) | Rueedi et al., 2014 | 24586186 |
| rs62113212 | 19 | 56052652 | 6,00E-09 | *KLK3* | Prostate cancer | Berndt et al., 2015 | 25939597 |
| rs57046232 | 20 | 6328344 | 5,00E-07 | *-* | Colorectal cancer | Al-Tassan et al., 2015 | 25990418 |
| rs1015563 | 20 | 6638101 | 2,00E-06 | *BMP2* | Colorectal cancer | Al-Tassan et al., 2015 | 25990418 |
| rs6104691 | 20 | 10944911 | 1,00E-06 | *-* | Mammographic density | Lindstrom et al., 2014 | 25342443 |
| rs6087685 | 20 | 33241273 | 2,00E-08 | *PROCR* | Venous thromboembolism | Germain et al., 2015 | 25772935 |
| rs6099314 | 20 | 54888066 | 4,00E-07 | *-* | Chronic obstructive pulmonary disease | Cho et al., 2014 | 24621683 |
| rs4812048 | 20 | 57021166 | 7,00E-06 | *CTSZ* | Platelet count or volume | Shameer et al., 2014 | 24026423 |
| rs2427308 | 20 | 60402846 | 3,00E-11 | *CABLES2* | Colorectal cancer | Al-Tassan et al., 2015 | 25990418 |
| rs170183 | 21 | 36770204 | 4,00E-09 | *CLDN14* | Bone mineral density | Zhang et al., 2014 | 24249740 |
| rs7289126 | 22 | 36958252 | 5,00E-09 | *TMEM184B* | Mammographic density | Lindstrom et al., 2014 | 25342443 |
| rs17001868 | 22 | 39108177 | 2,00E-13 | *SGSM3* | Mammographic density | Lindstrom et al., 2014 | 25342443 |
| rs5759167 | 22 | 41830156 | 1,00E-16 | *BIK* | Prostate cancer | Berndt et al., 2015 | 25939597 |
| rs2807031 | X | 52913674 | 4,00E-06 | *-* | Prostate cancer | Berndt et al., 2015 | 25939597 |
|  |  |  |  |  |  |  |  |

SNP: Single Nucleotide Polymorphism; Chr: Chromosome; GWAS: Genome Wide Association Study.

*in a window of 100 kb
