# Supplementary material for: An integrated analysis of genes and functional pathways for aggression in human and rodent models
Source: Mol Psychiatry. 2018 Jun 1;24(11):1655–67. doi: 10.1038/s41380-018-0068-7 (PMC6274606; doi:10.1038/s41380-018-0068-7)
Supplement: Supplementary file 6 — Supplementary Table 5 [file 41380_2018_68_MOESM6_ESM.docx]

**Supplementary Table 2. Sample sizes and sources of six psychiatric disorders used in LDSC analysis.**

| Disorder | Cases | Controls | Reference |
| --- | --- | --- | --- |
| Schizophrenia (SCZ) | 35476 | 46839 | Schizophrenia Working Group of the Psychiatric Genomics Consortium, et al. 2014 (PMID: 25056061) |
| Bipolar disorder (BIP) | 9784 | 30471 | Hou L, et al. 2016 (PMID: 27329760) |
| Major depression (MDD) | 9240 | 9519 | Major Depressive Disorder Working Group of the Psychiatric GWAS Consortium, et al. 2013 (PMID: 22472876) |
| Attention-Deficit/Hyperactivity Disorder (ADHD) | 20183 | 35191 | Demontis D, et al (2017 Preprint) https://www.biorxiv.org/content/early/2017/06/03/145581 |
| Autism (ASD) | 7387 | 8567 | Autism Spectrum Disorders Working Group of The Psychiatric Genomics Consortium, et al. 2017 (PMID: 28540026) |
| Posttraumatic Stress Disorder (PTSD) | 5131 | 15092 | Duncan LE, et al. 2017 (PMID: 28439101) |
