# Supplementary figures and images for: An integrated analysis of genes and functional pathways for aggression in human and rodent models
Source: Mol Psychiatry. 2018 Jun 1;24(11):1655–67. doi: 10.1038/s41380-018-0068-7 (PMC6274606; doi:10.1038/s41380-018-0068-7)

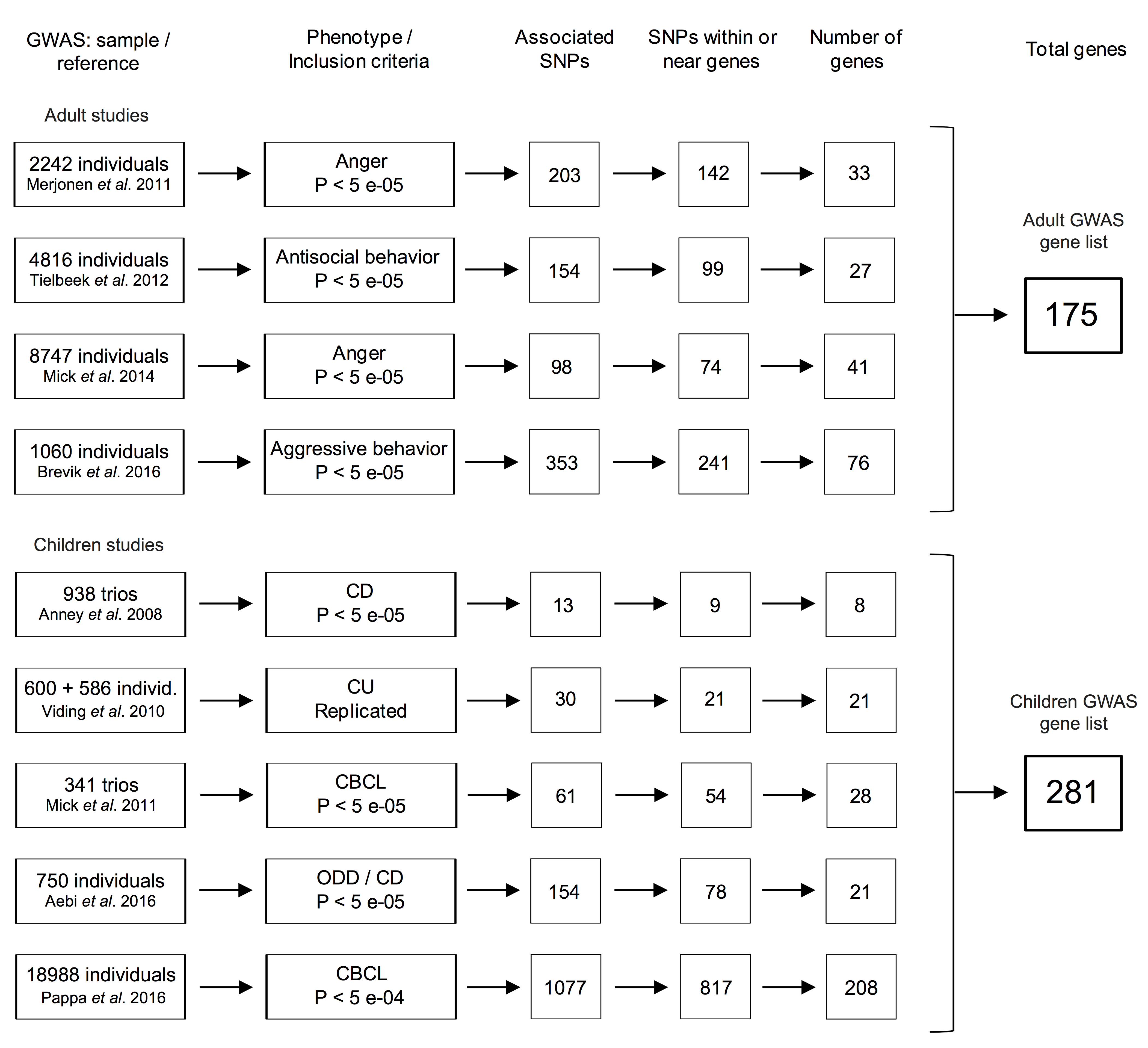

Supplement: Supplementary file 9 — Supplementary Figure 1 [file 41380_2018_68_MOESM9_ESM.tif]

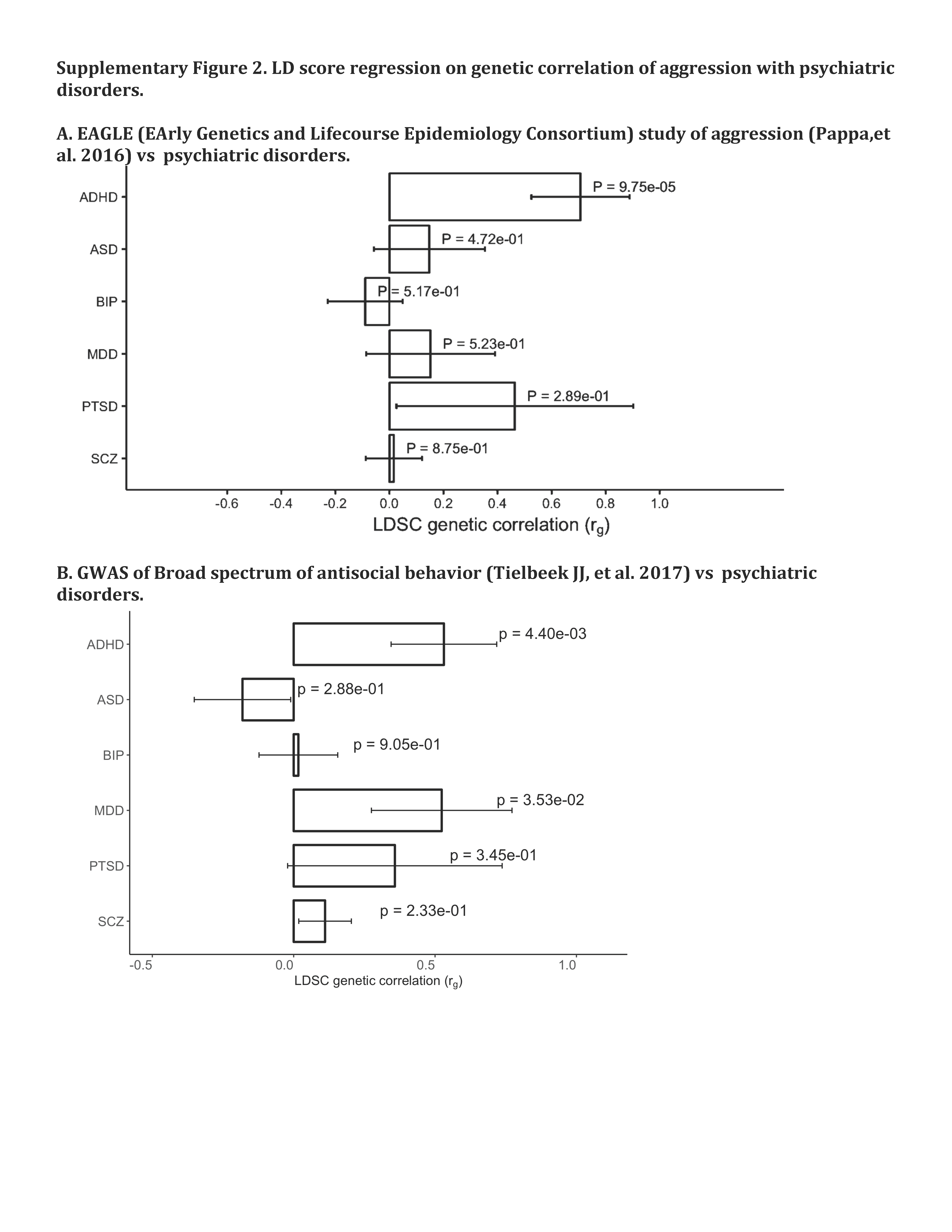

Supplement: Supplementary file 10 — Supplementary Figure 2 [file 41380_2018_68_MOESM10_ESM.tif]

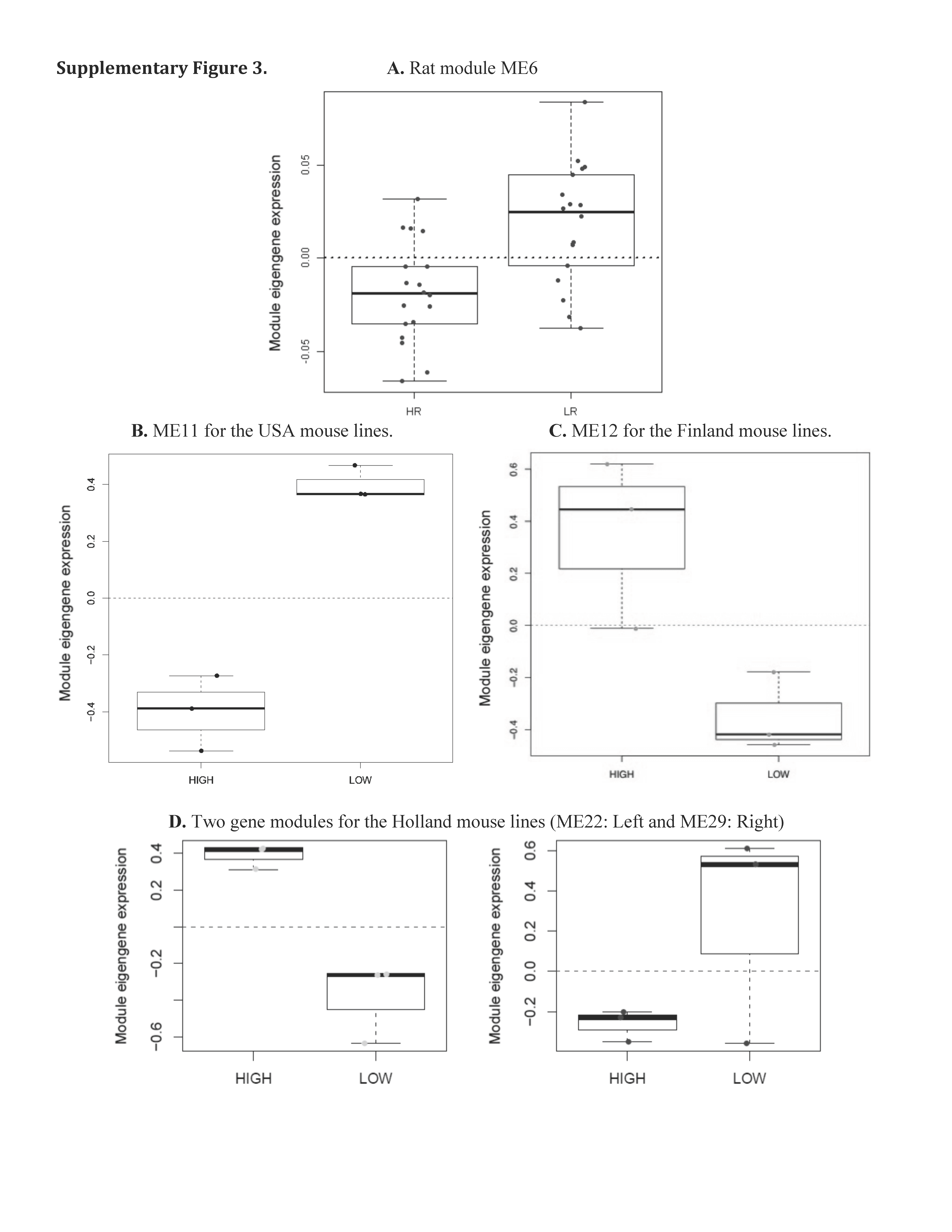

Supplement: Supplementary file 11 — Supplementary Figure 3 [file 41380_2018_68_MOESM11_ESM.tif]

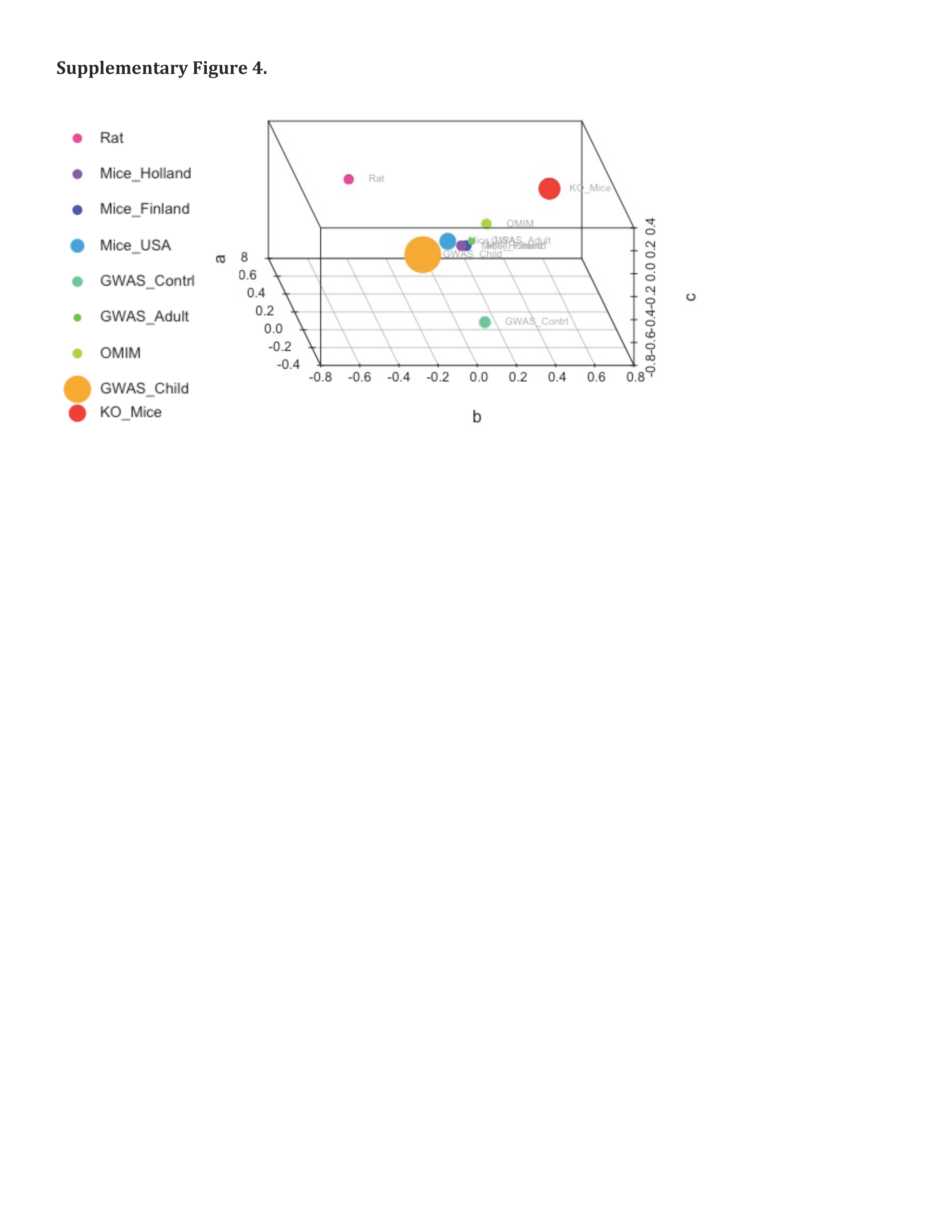

Supplement: Supplementary file 12 — Supplementary Figure 4 [file 41380_2018_68_MOESM12_ESM.tif]

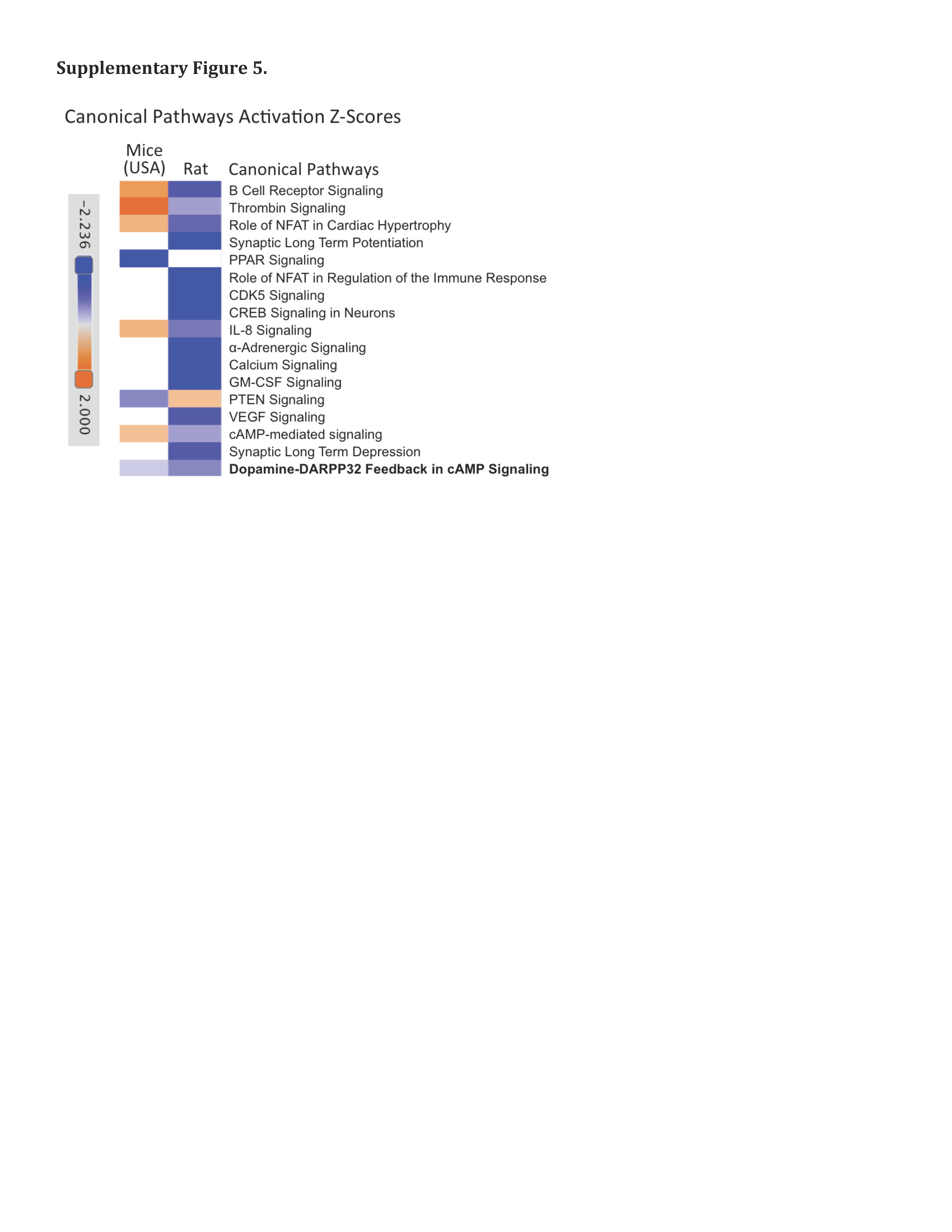

Supplement: Supplementary file 13 — Supplementary Figure 5 [file 41380_2018_68_MOESM13_ESM.tif]

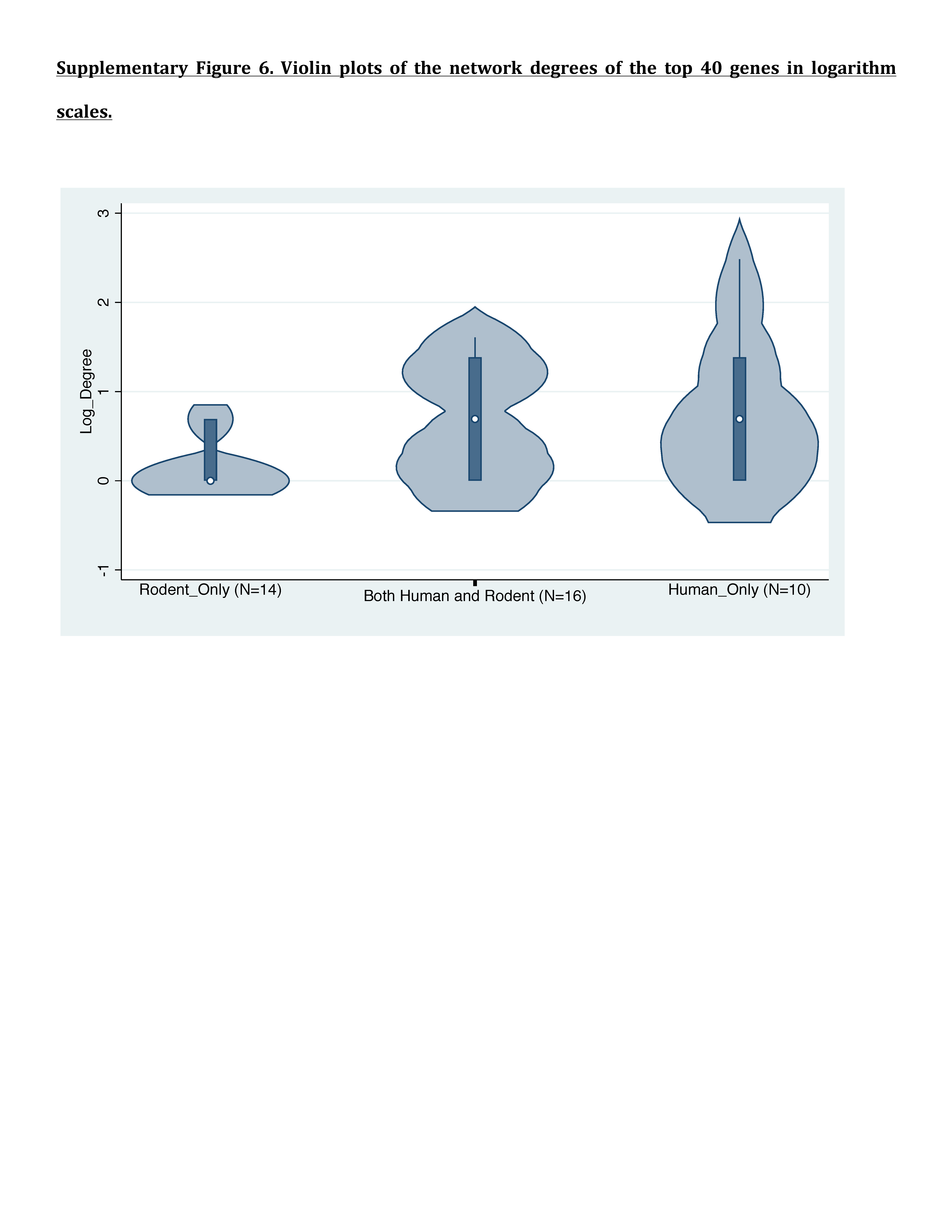

Supplement: Supplementary file 14 — Supplementary Figure 6 [file 41380_2018_68_MOESM14_ESM.tif]
