# Supplementary material for: An integrated analysis of genes and functional pathways for aggression in human and rodent models
Source: Mol Psychiatry. 2018 Jun 1;24(11):1655–67. doi: 10.1038/s41380-018-0068-7 (PMC6274606; doi:10.1038/s41380-018-0068-7)
Supplement: Supplementary file 15 — Supplementary File 1 [file 41380_2018_68_MOESM15_ESM.docx]

**Supplementary File**

***1. Weighted Gene Co-expression Network Analysis* (WGCNA) of rodent models and identification of aggression-specific modules and genes**

In total, 18 mouse (prefrontal cortex) and 36 rat (hippocampus-only) arrays were used. The raw data were imported into R for automated GC-adjusted RMA background correction, quantile normalization, and log2 transformation of microarray signal intensities using *affy* ^1^ and *gcrma* packages ^2^. Manufacturer probes were annotated with mouse or rat gene symbols using the *biomaRt* Bioconductor package ^3, 4^. We simplified the microarray expression matrices by calculating the median expression value for probe clusters that mapped to the same gene, then assigning this value to the corresponding gene, and discarding probes that did not map to known genes. The final gene count was 21,446 for mouse samples and 4,898 for rat samples (the rat array has a limited numbers of probes). We also implemented Surrogate Variable Analysis (**SVA**) using default algorithm (“be”) in R to identify and adjust for latent sources of variation in the microarray datasets orthogonal to factors of interest (*i.e.,* aggression level, age, strain source) ^5^. For the main analysis we used *Weighted Gene Co-expression Network Analysis* (**WGCNA**) ^6^ to generate separate unsupervised gene networks using normalized mouse and rat microarray data. Networks were generated using the one-step procedure *blockwiseModules*, which were tuned with soft-threshold power settings and various parameters. A soft-threshold power value (abbreviated as β) was used to raise all pair-wise correlations calculated across samples (*i.e.,* cor(x,y)^β^); a power value of 3 and 6 was applied to rat and mouse samples, respectively. Additionally, the following parameters were specified during gene network construction: *deepSplit* = 2, *minModuleSize* = 30, *minCoreKMESize* = 10, *minCoreKME* = 0.5, *reassignThreshold* = 1e-6, *mergecutHeight* = 0.2, and *detectCutHeight* = 0.9. *WGCNA* performs singular value decomposition and summarizes the variances of a network by calculating module eigengenes, which is equal to the first principal component derived from the expression matrix of genes belonging to a single module.

Linear regression models with Benjamini-Hochberg (BH) corrections tested for an association between aggression-status and eigengene expression levels. We covaried regressions for the rat data with animal’s age and seven surrogate variables identified from SVA. Mouse models were analyzed separately for each line. Their WGCNA network eigengenes were subjected to regression models to test for an association of aggression status with one significant surrogate variable identified from SVA for each strain. Only modules significant for aggression status were retained for the gene set comparison and pathway analysis.

**2. Overlap of our gene sets with other studies that were not included in the current study**

Non-genetic models, such as stress-induced aggression, were not included in our studies. However, it is worth mentioning some correspondences with our findings because genetic background can influence susceptibility to stress and modify stress-responsive aggressive behaviors. One transcriptomic study examined differences in the stress-prone BALB/c and stress-resistant C57BL/c mice lines upon mild chronic stress-exposure and compared them with the three aforementioned genetic mouse lines ^7^. There were a number of genes shared by the stress-induced and the genetic models, especially in the protein ubiquitination and p38 MAPK pathways. One of the shared genes was in our adult GWAS gene set, *HIBCH*. Another one, *FAM160B1*, was found in the bHR and bLR rat model. Another study used intruder-induced social-stress in mice and also highlighted the MAPK, phospholipase C and phosphoinositide 3-kinase (PI3K) pathways ^8^. Several of their reported genes are present in the lists of human GWAS or OMIM genes produced by us, and in KO mouse studies. These studies confirmed some shared genetic mechanisms underlying different types of human aggression and animal models, highlighting mechanisms that may underlie gene by environment interactions. On the contrary, there was no genes shared between our aggression gene sets and a rat model selectively bred by tameness and aggression towards humans ^9^, a type of aggression that are not within the species context. This suggests that animal models based on tameness may not be suitable for modeling human aggression.

Studies of other model organisms that we did not include in the current study, such as *Drosophila*, Zebrafish, chicken and dogs, also support the idea of a genetic contribution to aggression. A brain transcriptome study of hyper-aggressive *Drosophila prolongata* found 21 differentially expressed genes ^10^, three of which have low or moderate homologues in our human or mouse aggression gene lists: *MME* (Finland and KO mice), *OVGP1* (Finland) and *ST6GAL2* (USA mouse)/ *ST3GAL5* (KO mouse)/ *ST8SIA2* (KO mouse). A cross-species study of mouse and Zebrafish models found seven differentially expressed genes shared in both species ^11^. Four of them were also present in our non-mouse gene lists: *HDAC4* (child GWAS and OMIM), *DUSP1* (child GWAS), *BNDF* (KO mouse) and *BTG2*(rat). A GWAS of hundreds of dog breeds reported genes associated with fear and aggression. Some of them had also been identified in human GWAS, such as *FSTL4* (adult) and *HMGA2* (child) and in rodent models, like *IGF1*(Finland) ^12^. All these studies support the utility of a cross-species approach like ours in the identification of important genetic mechanisms that are evolutionary conserved.

1. Gautier L, Cope L, Bolstad BM, Irizarry RA. affy--analysis of Affymetrix GeneChip data at the probe level. *Bioinformatics* 2004; **20**(3)**:** 307-315.

2. Wu J, Gentry R. gcrma: Background Adjustment Using Sequence Information. *R package version* 2016; (2.44.0.).

3. Durinck S, Spellman PT, Birney E, Huber W. Mapping identifiers for the integration of genomic datasets with the R/Bioconductor package biomaRt. *Nat Protoc* 2009; **4**(8)**:** 1184-1191.

4. Durinck S, Moreau Y, Kasprzyk A, Davis S, De Moor B, Brazma A *et al.* BioMart and Bioconductor: a powerful link between biological databases and microarray data analysis. *Bioinformatics* 2005; **21**(16)**:** 3439-3440.

5. Leek JT, Johnson WE, Parker HS, Jaffe AE, Storey JD. The sva package for removing batch effects and other unwanted variation in high-throughput experiments. *Bioinformatics* 2012; **28**(6)**:** 882-883.

6. Langfelder P, Horvath S. WGCNA: an R package for weighted correlation network analysis. *BMC bioinformatics* 2008; **9:** 559.

7. Malki K, Tosto MG, Pain O, Sluyter F, Mineur YS, Crusio WE *et al.* Comparative mRNA analysis of behavioral and genetic mouse models of aggression. *Am J Med Genet B Neuropsychiatr Genet* 2016; **171B**(3)**:** 427-436.

8. Muhie S, Gautam A, Meyerhoff J, Chakraborty N, Hammamieh R, Jett M. Brain transcriptome profiles in mouse model simulating features of post-traumatic stress disorder. *Mol Brain* 2015; **8:** 14.

9. Heyne HO, Lautenschlager S, Nelson R, Besnier F, Rotival M, Cagan A *et al.* Genetic influences on brain gene expression in rats selected for tameness and aggression. *Genetics* 2014; **198**(3)**:** 1277-1290.

10. Kudo A, Shigenobu S, Kadota K, Nozawa M, Shibata TF, Ishikawa Y *et al.* Comparative analysis of the brain transcriptome in a hyper-aggressive fruit fly, Drosophila prolongata. *Insect Biochem Mol Biol* 2017; **82:** 11-20.

11. Malki K, Du Rietz E, Crusio WE, Pain O, Paya-Cano J, Karadaghi RL *et al.* Transcriptome analysis of genes and gene networks involved in aggressive behavior in mouse and zebrafish. *Am J Med Genet B Neuropsychiatr Genet* 2016; **171**(6)**:** 827-838.

12. Zapata I, Serpell JA, Alvarez CE. Genetic mapping of canine fear and aggression. *BMC genomics* 2016; **17:** 572.
